# The reprogramming and function of H4K20me1 during early embryo development

**DOI:** 10.1038/s44319-026-00780-x

**Published:** 2026-04-22

**Authors:** Xiangrui Meng, Zhen He, Dong Fang, Wenbo Li, Jia Guo, Jiacheng Lu, Fen Yan, Xin Dong, Lingling Chen, Sarmir Khan, Chunling Wang, Jiawei Xu

**Affiliations:** 1 The First Affiliated Hospital of Zhengzhou University & Institute of Reproductive Health, Henan Academy of Innovations in Medical Science, Zhengzhou, China; 2 NHC Key Laboratory of Birth Defects Prevention, Zhengzhou, China; 3 Institute of Reproductive Health, Henan Academy of Innovations in Medical Science, Zhengzhou, China; 4 Luoyang Maternal and Child Health Hospital, Luoyang, China

**Keywords:** Chromatin, Transcription & Genomics, Development

## Abstract

Histone modifications play critical roles in regulating chromatin dynamics and embryonic development. Among these, histone H4 lysine 20 mono-methylation (H4K20me1) is an essential epigenetic mark associated with gene expression and genome stability. However, the reprogramming and functional roles of H4K20me1 in early embryogenesis remain unclear. Here, we map genome-wide distributions of H4K20me1 in mouse, human, and zebrafish early embryos, revealing a broad distribution pattern along with species-specific features. H4K20me1 is predominantly enriched in gene bodies and undergoes dynamic erasure and reestablishment following fertilization. Functional perturbation of SET8, the only known H4K20me1 methyltransferase, results in developmental arrest, highlighting its necessity for embryogenesis. Mechanistically, H4K20me1 is crucial for zygotic genome activation (ZGA), where it regulates RNA synthesis and transcription, and promotes chromatin accessibility. Our findings provide insights into the dynamic reprogramming and regulatory functions of H4K20me1 in early developmental processes.

## Synopsis

H4K20me1 is dynamically reprogrammed in preimplantation embryos, playing an essential role in zygotic genome activation by promoting chromatin accessibility and transcription.

- H4K20me1 exhibits a broad distribution pattern with species-specific features in mouse, human, and zebrafish embryos.
- H4K20me1 undergoes erasure-reestablishment at promoters and gene bodies during early mouse embryo development.
- Loss of SET8-mediated H4K20me1 in mouse embryos leads to developmental arrest, reduced chromatin accessibility and aberrant gene expression.

## Abstract (web summary)

H4K20me1 is dynamically reprogrammed in preimplantation embryos, playing an essential role in zygotic genome activation by promoting chromatin accessibility and transcription.

## Graphical abstract

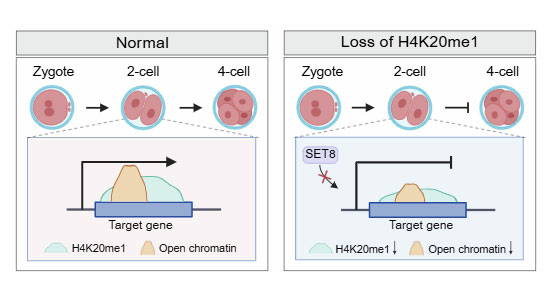

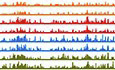

## Introduction

Precise gene regulation is essential for the development of multicellular organisms. During early development, a series of key biological processes, such as maternal-to-zygotic transition (MZT) (Vastenhouw et al, 2019; Sha et al, 2020) and among which zygotic genome activation (ZGA) (Schulz and Harrison, 2019; Chen et al, 2023; Yuan et al, 2023), mutually propel each other to complete the whole process. These processes are regulated by various genetic and epigenetic modifications that coordinate to ensure the completion of development in a mammalian individual (Burton and Torres-Padilla, 2025). Cell division and differentiation are extremely active throughout early embryonic development, with epigenetic marks playing a particularly prominent role (Xu and Xie, 2018; Zhang et al, 2018b). Epigenetic modifications, such as DNA methylation (Greenberg and Bourc’his, 2019), histone modifications (Sinha et al, 2023), and chromatin remodeling (Clapier et al, 2017; Alexanian, 2022), are crucial for the establishment and maintenance of a dynamic chromatin structure (Clapier et al, 2017; Bourguet et al, 2021; Li et al, 2022). Alterations in epigenetic modifications can trigger either gene activation or gene silencing. Furthermore, gene expression can be regulated by modulating the genome into autosomal active regions, where chromatin is more accessible for transcription, or into inactive heterochromatin regions, where chromatin is more compact and less easily transcribed (Bourguet et al, 2021; Gaspar-Maia et al, 2011; Liu et al, 2022; Bartosovic and Castelo-Branco, 2023). These regulatory processes affect embryonic development, and in severe cases, they can even cause miscarriage, stillbirth, and fetal malformations (Wei et al, 2023). Therefore, cell state transitions in development and disease are influenced by the epigenetic states of these factors (Wilkinson et al, 2023).

Histone post-translational modifications, as key regulatory events throughout preimplantation embryogenesis, serve as an important class of epigenetic regulators that regulate gene expression and various essential cellular and biological processes. They are vital for the dynamic reprogramming of chromatin structure and function, as well as for changes in gene expression (Peterson and Laniel, 2004; Suganuma and Workman, 2011). Diverse histone marks are localized to specific regions of the genome and perform biological functions. For example, the transcriptional activation marker H3K4me3 is primarily found at promoters (Ruthenburg et al, 2007), and the transcriptional repression marker H3K27me3 is typically located at CpG-rich promoters and intergenic regions (Frapporti et al, 2019). These histone modifications undergo a reprogramming process of build-up, erasure, and rebuild during gametogenesis and embryonic development (Liu et al, 2016; Zheng et al, 2016). Although several histone modifications have been extensively investigated, there are still many modifications whose functions and regulatory mechanisms in early embryonic development remain unclear.

Histone H4 lysine 20 (H4K20) methylation is observed in various eukaryotes and is evolutionarily conserved across species from yeast to humans (Jørgensen et al, 2013). This modification exists in three states as me1, me2, and me3, each of which has distinct biological activities (Balakrishnan and Milavetz, 2010; Corvalan and Coller, 2020). Unlike H4K20me3, which is preferentially enriched in repetitive elements, H4K20me1 is mainly enriched in gene bodies and is catalyzed by the histone methyltransferase SET8 (also known as KMT5A or PR-SET7/9) (Congdon et al, 2010). H4K20me1 regulates numerous nuclear functions, such as transcription, chromosome cohesion, and cell cycle control, and is a key regulator of the overall life process (Balakrishnan and Milavetz, 2010). It has been demonstrated that SET8-mediated H4K20me1 directly promotes chromatin accessibility in U2OS cells by disrupting chromatin folding (Dorigo et al, 2003; Sakaguchi et al, 2012; Shoaib et al, 2018), and also affects gene activation and repression, as well as Pol II promoter-proximal arrest (Perner et al, 2014; Nikolaou et al, 2017). In addition, studies have revealed that H4K20me1 is necessary for mouse development (Oda et al, 2009; Shikata et al, 2020). It functions in maintaining genome integrity by repairing DNA damage induced during embryonic development (Shikata et al, 2020). These interesting findings led us to try to find changes in the dynamic distribution pattern of H4K20me1 in oocytes and early embryos, for chromatin accessibility and potential regulation by ZGA.

In this study, we explored the genome-wide dynamic distribution of H4K20me1 from mouse oocytes to blastocysts, as well as in early embryos of human and zebrafish. Using the CUT&RUN technique with inputs as low as 100 cells, we found that H4K20me1 exhibits a broad distribution pattern enriched in gene bodies, with additional species-specific localization to promoters and distal intergenic regions. Notably, in the mouse, H4K20me1 undergoes extensive reprogramming during the maternal-to-zygotic transition. The decline of H4K20me1 in the embryos leads to the blockage of embryonic development mostly at the 2-cell stage, as well as the failure of zygotic genome activation, with a massive reduction in chromatin accessibility and disruption of normal ZGA gene expression. Therefore, our study reveals the dynamic distribution changes and regulatory functions of H4K20me1 in early development.

## Results

### H4K20me1 exhibits a broad pattern in the oocytes and preimplantation embryos

**Figure EV1:**
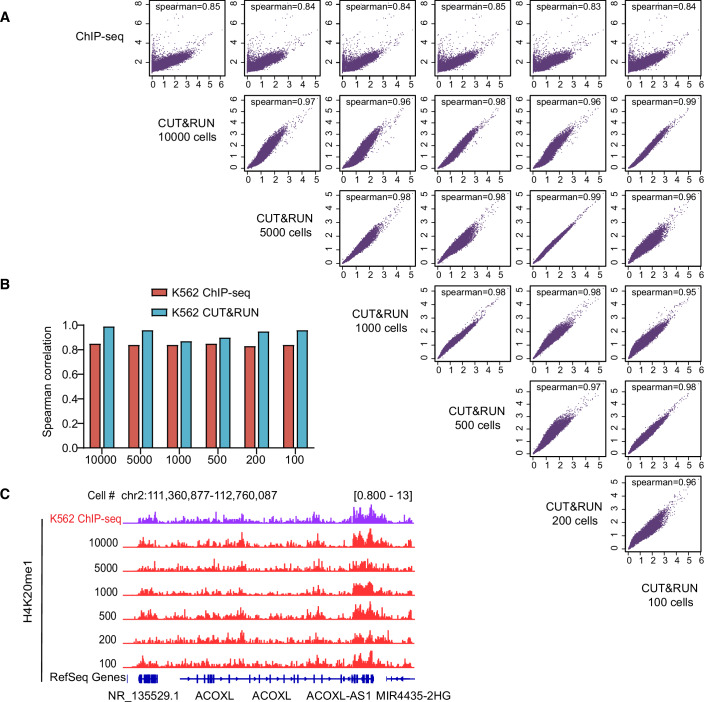
Validation of the low-input CUT&RUN approach. (**A**) Spearman correlation analysis showing that high correlations between different cell inputs in K562 cells of CUT&RUN data and ChIP-seq data (obtained from GSE29611) of H4K20me1. The axes in the plots represent log2(normalized signal + 1) values. (**B**) Correlations were assessed between: (i) H4K20me1 CUT&RUN data (from the indicated K562 cell numbers) and the ChIP-seq data (blue bars), and (ii) two biological replicates of the CUT&RUN data at the same cell number (red bars). (**C**) Landscapes of different K562 cell input amounts of H4K20me1 detected with CUT&RUN and a large number of K562 cell amounts of H4K20me1 detected by ChIP-seq.

**Figure EV2:**
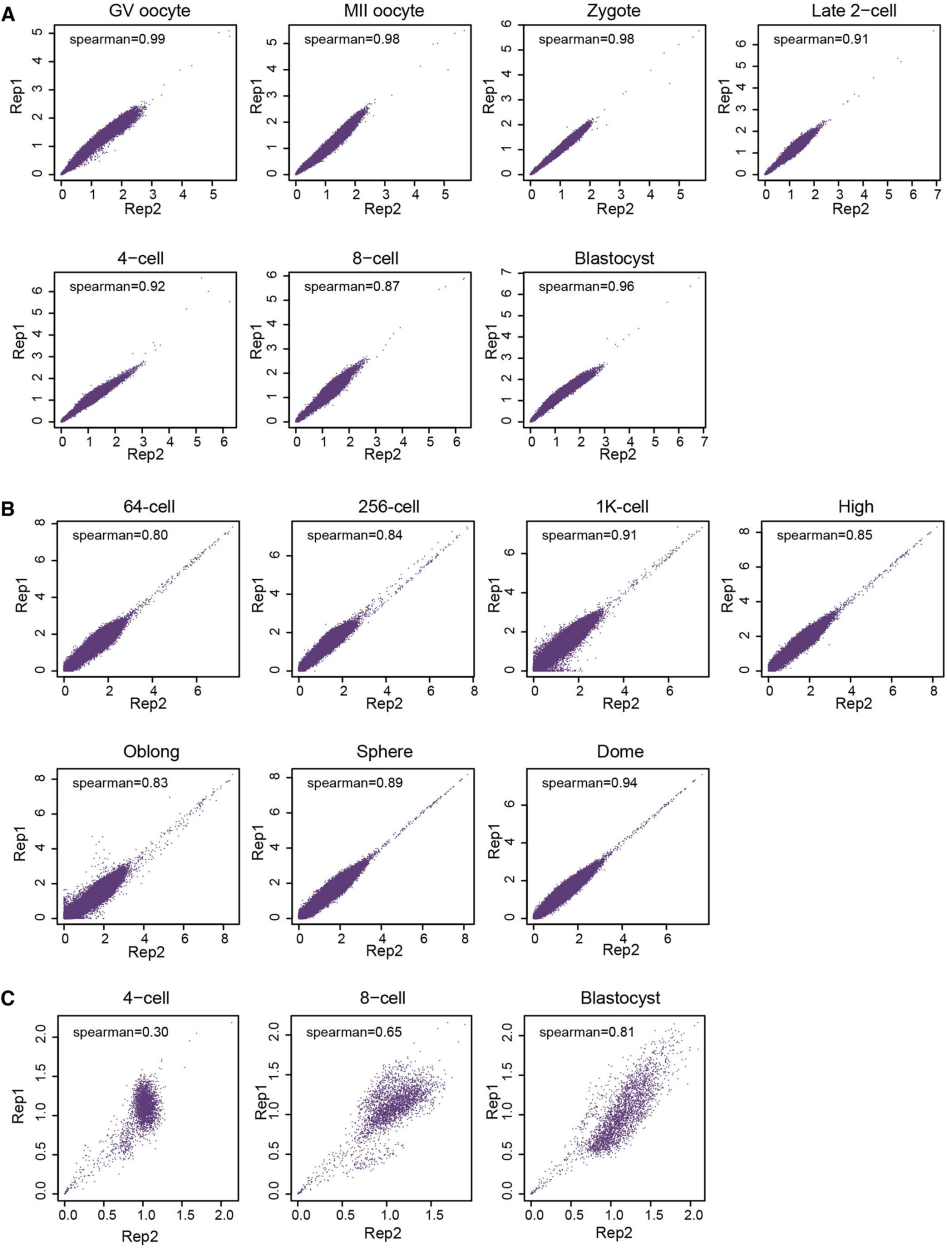
Correlations between biological replicates of CUT&RUN. (**A**) The strong correlations between biological replicates at each stage from GV oocyte to blastocyst in mice demonstrate the high quality of the CUT&RUN data. (**B**) Spearman correlation analysis of CUT&RUN data across zebrafish embryonic stages, demonstrating the high reproducibility between biological replicates. (**C**) Spearman correlations between biological replicates are shown for the 4-cell, 8-cell, and blastocyst stages in human. The lower correlations observed at the earlier stages (particularly the 4-cell stage) are likely attributable to the extremely limited sample availability and the resulting technical variability, including differences in sequencing depth between these rare specimens. The axes in the plots represent log2(normalized signal + 1) values.

To investigate the genome-wide reprogramming pattern and regulatory mechanism of H4K20me1 in oocytes and preimplantation embryos, it is essential to unfold the localization and distribution of the histone modification H4K20me1. We used a highly sensitive micro-scale cleavage under targets and release using nuclease (CUT&RUN) analysis (Skene and Henikoff, 2017; Skene et al, 2018; Xia et al, 2019) and validated CUT&RUN for H4K20me1. Notably, our detection of H4K20me1 peaks from as few as 100 cells has a correlation of 0.84 with peaks detected for ChIP-seq (Data ref: ENCODE Project Consortium, 2011) and a correlation of 0.99 with peaks detected from 10,000 cells (Fig. EV1A). The H4K20me1 enrichment was highly correlated and similar to conventional ChIP-seq, as well as other mapping of cell inputs (Fig. EV1B,C). After validating the method, we performed CUT&RUN to generate genome-wide H4K20me1 maps for three vertebrate species, mouse, human, and zebrafish (Table EV1). For mouse, we analyzed germinal vesicle (GV) oocytes, metaphase II (MII) oocytes, zygotes, and preimplantation embryos from late 2-cell to blastocyst stages. Notably, the fraction of reads in peak values was relatively low in some stages, which may reflect the broad distribution of H4K20me1, or the lower signal-to-noise ratio typical of low-input samples (Table EV2). In addition, human embryos at the 4-cell, 8-cell, and blastocyst stages, and zebrafish embryos from 64-cell to dome stages were collected. The experiments were performed with two biological replicates (Fig. EV2), and the biological replicates were merged for subsequent analyses.

**Figure 1:**
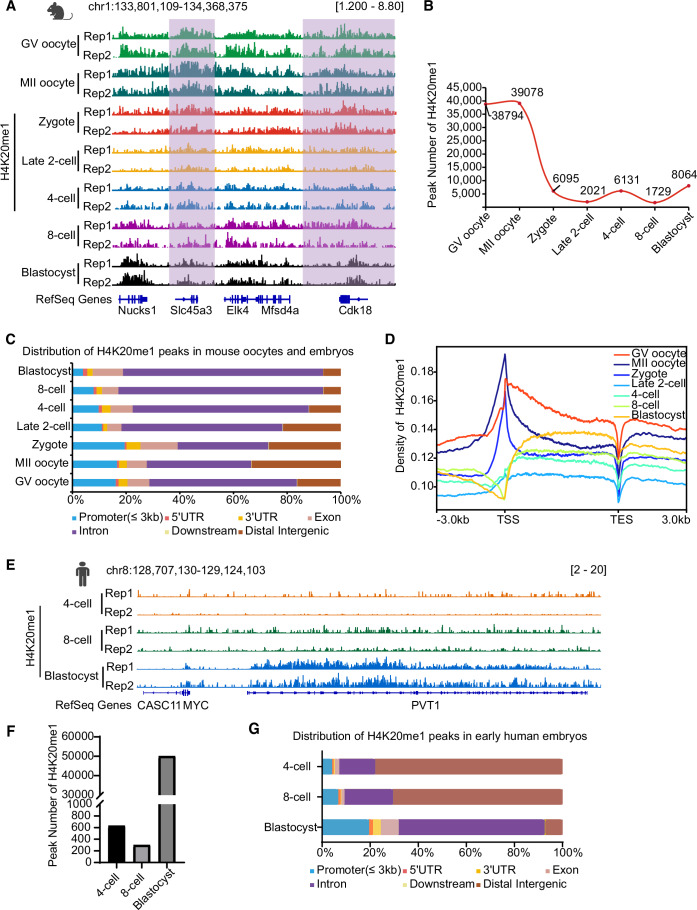
Genome-wide mapping of H4K20me1 in oocytes and early embryos. (**A**) IGV Browser showing enrichment of H4K20me1 CUT&RUN signal in mouse oocytes and early embryos (two biological replicates at each stage). The purple shading indicates the representative regions shown in the tracks. (**B**) Peak number of H4K20me1 at different developmental stages in mice. (**C**) The proportion of mouse H4K20me1 peaks in different genomic features at various developmental stages, with peaks assigned to promoter ( ≤ 3 kb), 5’UTR, 3’UTR, exon, intron, downstream, and distal intergenic regions. (**D**) Metaplot shows genomic enrichment of H4K20me1 signal during different developmental periods in mice. The y‑axis represents the average H4K20me1 signal density. (**E**) IGV Browser showing enrichment of H4K20me1 CUT&RUN signal in early human embryos. (**F**) Peak number of H4K20me1 at different developmental stages in humans. (**G**) The proportion of human H4K20me1 peaks in different genomic features at various developmental stages, with peaks assigned to promoter (≤ 3 kb), 5’UTR, 3’UTR, exon, intron, downstream, and distal intergenic regions. Source data are available online for this figure.

**Figure EV3:**
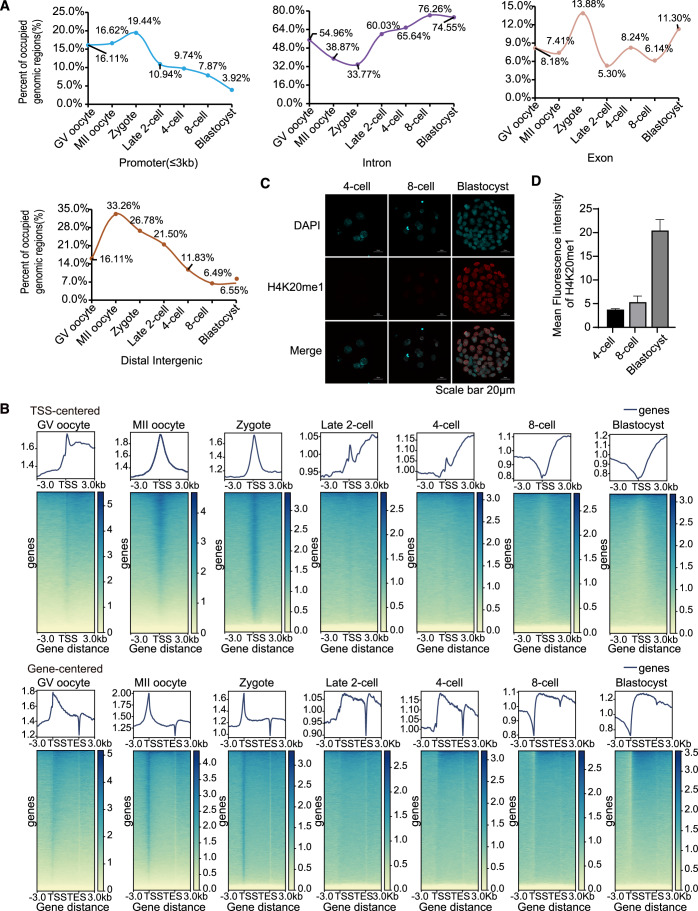
The dynamics of H4K20me1 in mice and humans. (**A**) Percentage of H4K20me1 peaks assigned to promoters, introns, exons, and intergenic regions at different developmental stages of the mouse. (**B**) Heatmap analysis showing TSS-centered and gene-centered distribution of H4K20me1 CUT&RUN signals in mouse oocytes and early embryos. The *y* axis represents the average H4K20me1 signal density across all genes. (**C**) Immunofluorescence images showing H4K20me1 (red), DAPI-stained nuclei (cyan), and the merged signals in human embryos. Scale bar: 20 µm. (**D**) Mean fluorescence values of H4K20me1 in human embryos. The total number of embryos analyzed per developmental stage was as follows: 4-cell (4), 8-cell (4), and blastocyst (7). Data are presented as mean ± SEM. Source data are available online for this figure.

We observed a broad peak distribution pattern of H4K20me1 in mouse oocytes and preimplantation embryos (Fig. 1A). Using model-based analysis of ChIP-seq (MACS) (Zhang et al, 2008) for peak calling, we found that H4K20me1 peaks were more abundant in oocytes, while the number of peaks was substantially reduced at the late 2-cell stage, a change coinciding with the timing of major ZGA (Fig. 1A,B). We also performed an analysis of the distribution of H4K20me1 peaks in genomic elements at different developmental stages. The results showed that H4K20me1 peaks were present in the promoters, gene body regions (introns and exons), and distal intergenic regions, especially enriched in the gene body regions, consistent with previously reported results in human-derived cells (Congdon et al, 2010) (Fig. 1C). At the oocyte-to-zygote stage, H4K20me1 peaks were mainly enriched in promoters (16.11%–19.44%), gene bodies (63.14%–47.65%), and distal intergenic regions (16.11%–26.78%). In late 2-cell embryos, there was a notable enrichment of H4K20me1 peaks in gene bodies (65.33%), especially within intron regions (60.03%), and the percentage of coverage increased as development progressed (Figs. 1C and EV3A). Then, we focused on the distribution pattern of H4K20me1 signal in the TSS-TES profile, where H4K20me1 was enriched in the transcription start site (TSS) of oocytes and zygotes in a single-peak pattern, and the distribution of H4K20me1 shifted from the TSS to the flanking regions at the late 2-cell stage, resulting in a bimodal pattern (Figs. 1D and EV3B). In contrast, the enrichment of H4K20me1 in gene bodies and transcription termination sites (TES) declined continuously from oocyte to the late 2-cell embryos, and rebuilt in both regions after the late 2-cell stage (Figs. 1D and EV3B).

However, in early human embryos, H4K20me1 peaks remained relatively low at both the 4-cell (pre-ZGA) and 8-cell (peri-ZGA) stages (Fig. 1E,F), with predominant enrichment in distal intergenic regions (Fig. 1G). After the major ZGA in human, H4K20me1 peaks at the blastocyst stage showed a marked increase, with enrichment mainly in promoters and gene bodies (Fig. 1E–G), similar to that observed in mice. Immunofluorescence staining also confirmed the dynamic changes of H4K20me1 during early human development (Fig. EV3C,D). These results suggest that broad domains of H4K20me1 do not necessarily occur at the same developmental stage in different mammalian species.

**Figure 2:**
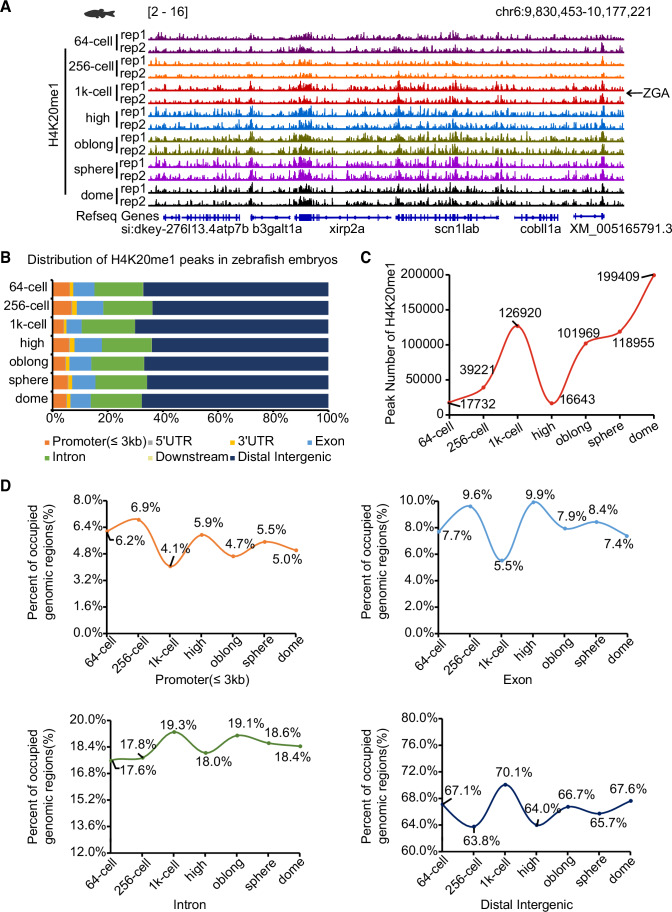
Genome-wide mapping of H4K20me1 in early zebrafish embryos. (**A**) IGV Browser showing enrichment of H4K20me1 CUT&RUN signal in early zebrafish embryos (two biological replicates at each stage). The timing of zygotic genome activation (ZGA) in zebrafish is indicated. (**B**) The proportion of H4K20me1 peaks in different genomic features at various developmental stages, with peaks assigned to promoter (≤ 3 kb), 5’UTR, 3’UTR, exon, intron, downstream, and distal intergenic regions. (**C**) Peak number of H4K20me1 at different developmental stages in zebrafish. (**D**) Dynamic changes in the proportion of H4K20me1 peaks coverage in different genomic features, with peaks assigned to the promoter, exon, intron, and distal intergenic region. Source data are available online for this figure.

We next asked whether the distribution pattern of H4K20me1 is unique to mammals, and equally focused on genome-wide mapping of H4K20me1 in zebrafish. Consistent with findings in mice and humans, H4K20me1 also showed a broad distribution pattern in early zebrafish embryos (Fig. 2A), with peaks predominantly observed in introns and distal intergenic regions (Fig. 2B). Notably, unlike in mice, H4K20me1 peaks increased continuously from the 64-cell to the 1k-cell, reaching a high level at the major ZGA stage in zebrafish (Fig. 2C). The enrichment proportion of H4K20me1 peaks in different genomic elements changed notably at the major ZGA stage (Fig. 2D), with the coverage proportion in promoters and exons decreasing substantially, while the coverage proportion in introns and distal intergenic regions increased. These data indicate that the broad-domain distribution pattern of H4K20me1 in early development is a common feature of embryos in mice, zebrafish, and humans. However, the timing of its redistribution relative to ZGA, as well as its enrichment levels and fine-scale genomic localization, exhibits notable species-specific differences.

### H4K20me1 undergoes erasure-reprogramming in preimplantation embryos

**Figure 3:**
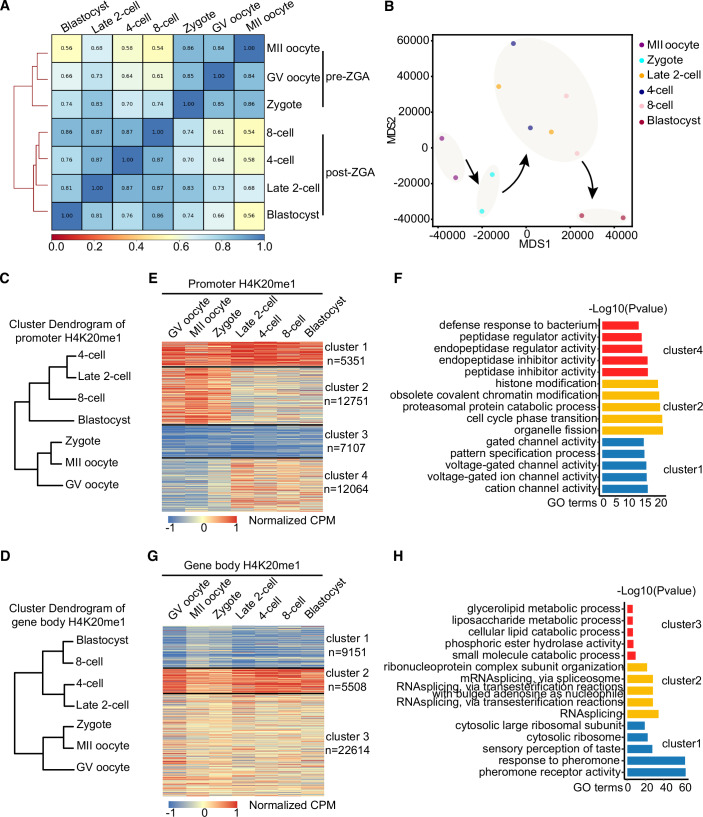
Reprogramming of promoters and gene bodies by H4K20me1 in mouse oocytes and early embryos. (**A**) Correlation clustering heatmap of H4K20me1 signal in mouse oocytes and early embryos. (**B**) MDS analysis of H4K20me1 profiles, with different colors representing different developmental periods. (**C**, **D**) Hierarchical clustering of the promoter (**C**) and gene body (**D**) H4K20me1 enrichment in mouse oocytes and early embryos. (**E**) Dynamics of H4K20me1 enrichment at gene promoters in oocytes and early embryos. Genes are clustered into four groups, and the number of genes in each cluster is shown. Data represent Z-score normalized signal intensity (based on CPM). CPM, counts per million. (**F**) The enriched Gene Ontology (GO) terms for gene cluster 1–2 and cluster 4 in (**E**). Statistical significance was determined using the hypergeometric test. (**G**) Dynamics of H4K20me1 enrichment at gene bodies in oocytes and early embryos. Genes are clustered into three groups, and the number of genes in each cluster is shown. Data represent Z-score normalized signal intensity (based on CPM). (**H**) GO term enrichment results for clusters 1–3 shown in (**G**). Statistical significance was determined using the hypergeometric test.

**Figure EV4:**
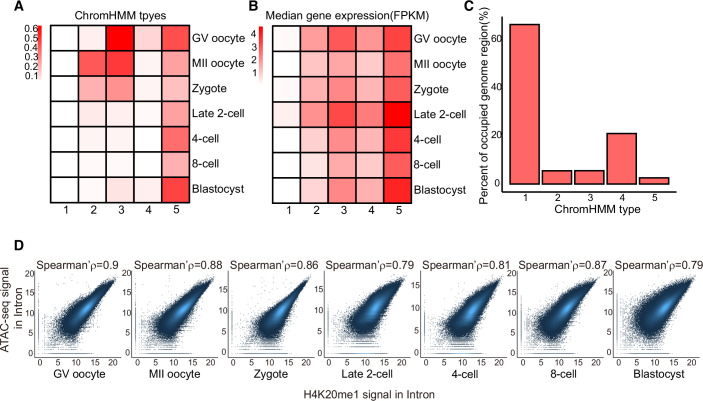
Reprogramming of H4K20me1 in mouse oocytes and early embryos. (**A**) Heatmaps of H4K20me1 dynamics for each ChromHMM type in oocytes and early embryos. Red color intensity reflects the level of H4K20me1 signal for each state at respective developmental stages. (**B**) Heatmap of the dynamics of each H4K20me1 ChromHMM type gene at different developmental stages. The intensity in red in each cell indicates the median number of fragments per kb per million reads (FPKM). (**C**) Genomic coverage proportions for each ChromHMM state. (**D**) Scatter plots comparing H4K20me1 signals with ATAC-seq signals in introns across developmental stages. Spearman correlation coefficients are shown. All *P* values < 2.2e^–16^.

To investigate the reprogramming of H4K20me1 during early development, we performed hierarchical clustering and multiple-dimensional scaling (MDS) analyses based on global H4K20me1 in mice. Mouse oocytes and zygotes were distinctly clustered apart from two-cell embryos and later stages, as revealed by hierarchical clustering analysis (Fig. 3A). In addition, MDS analysis separated the late 2-cell embryos from zygotes, showing that H4K20me1 was substantially altered specifically at the late 2-cell stage (Fig. 3B). Then, we classified genomic regions into 5 types based on H4K20me1 dynamics in early development using the ChromHMM method (Ernst and Kellis, 2012) (Fig. EV4A). Specifically, type 5 “stable” represents that H4K20me1 accumulates at various developmental stages, but there are dynamic changes in H4K20me1 levels (Fig. EV4B,C).

H4K20me1 was mainly distributed in promoters and gene bodies in early mouse embryos. Our hierarchical cluster analysis showed that the accumulation of promoters H4K20me1 in GV oocyte-zygote embryos was different from that in late 2-cell embryos to blastocysts (Fig. 3C), and that the accumulation of H4K20me1 in gene bodies was also divided into before and after late 2-cell embryos (Fig. 3D). By using the k-means algorithm, the accumulation of H4K20me1 at promoters in oocytes and early embryos was dynamically reprogrammed and classified into four clusters: accumulation at all stages (cluster 1), accumulation before zygotic genome activation (cluster 2), no accumulation at any stage (cluster 3), and obtained after zygotic genome activation (cluster 4) (Fig. 3E). Gene Ontology (GO) analyses showed that genes deposited by promoters H4K20me1 in cluster 1 were involved in physiological functions of the basis-voltage-gated channel activity; and genes deposited in cluster 2 were involved in histone modification, obsolete covalent chromatin modification, and cell cycle (Fig. 3F). In contrast, there was differential reprogramming of H4K20me1 accumulation in the gene bodies: All three clusters shared a similar pattern, differing primarily in their H4K20me1 signal levels (Fig. 3G). These genes highly enriched in H4K20me1 in the gene bodies (cluster 2) were involved in ribonucleoprotein complex subunit organization and mRNA splicing process (Fig. 3H). Taken together, these data suggest that H4K20me1 is reprogrammed in mouse oocytes and preimplantation embryos and is associated with regulating various biological functions in early development.

### H4K20me1 is strongly associated with chromatin opening in oocytes and early embryos

Chromatin accessibility describes the continuum of chromatin in different states of compaction, which extends from compact parthenogenetic and constitutive heterochromatin to more open and accessible euchromatin. In mammals, dynamic changes in chromatin structure are essential for the correct regulation of chromosome function (Venkatesh and Workman, 2015). The basic components of chromatin are nucleosomes around which DNA is encapsulated, and for genomes, the degree of chromatin densification is determined by the accumulation of nucleosomes and chromatin-binding proteins (Bartosovic and Castelo-Branco, 2023). H4K20 methylation has been implicated in the maintenance of a compact chromatin state (Shoaib et al, 2018). Specifically, H4K20me1 facilitates an open and accessible chromatin environment by disrupting chromatin folding (Shoaib et al, 2021). This relationship was further validated through a joint analysis of our H4K20me1 data with published chromatin accessibility datasets (Data ref: Jung et al, 2019; Gou et al, 2020; Wu et al, 2016) in early mouse embryos.

**Figure 4:**
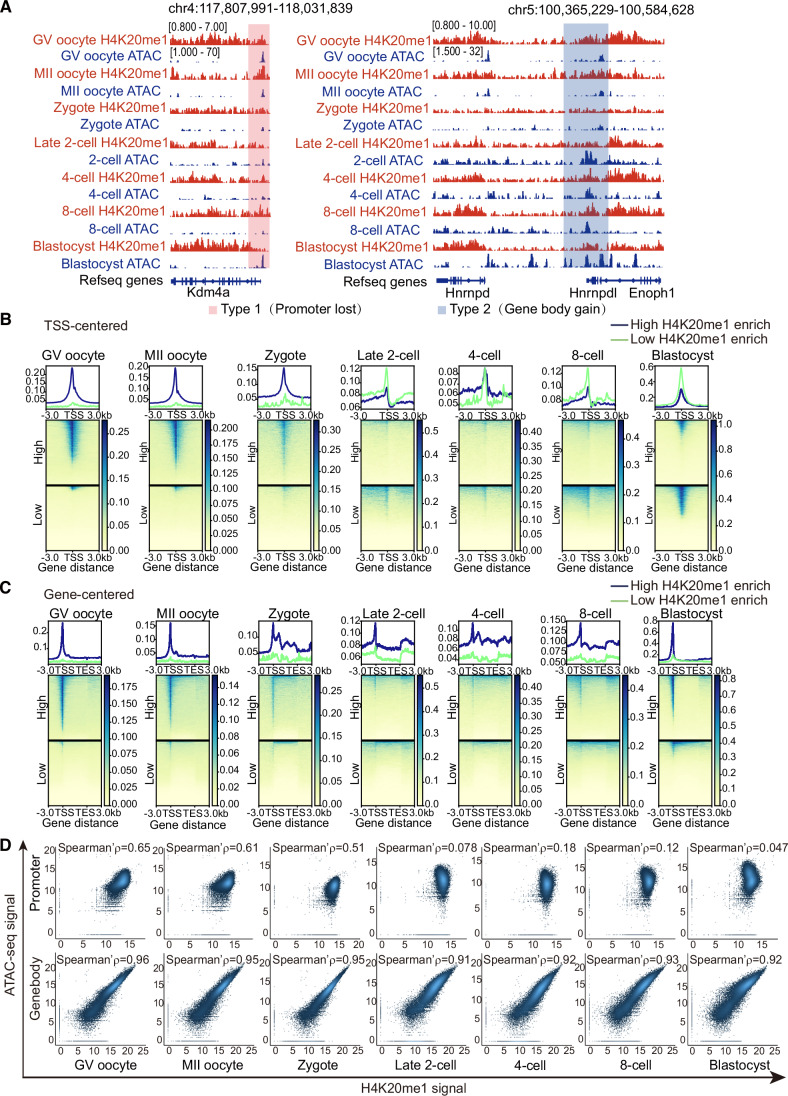
Relationship of H4K20me1 and chromatin accessibility in mouse oocytes and early embryos. (**A**) IGV Browser showing enrichment of ATAC-seq data (GEO data) and H4K20me1 CUT&RUN data at different developmental stages in mice. (**B**, **C**) Metagene heatmap shows the distribution of TSS-centered (**B**) or gene-centered (**C**) ATAC-seq signals in the H4K20me1 high-enriched region as well as in the H4K20me1 low-enriched region. Peaks in the top 25% of H4K20me1 signal intensity were classified as high-enrichment, while those in the bottom 25% were defined as low-enrichment. The *y* axis represents the average signal density. (**D**) Correlation between H4K20me1 and ATAC-seq signals in promoter (3 kb upstream of TSS) and gene body (TSS to TES) regions of mouse oocytes and early embryos. The axes in the plots represent log2(FPKM + 1) values. Spearman correlation coefficients are shown. All *P* values < 2.2e^−16^.

As H4K20me1 is mainly enriched in promoters and gene bodies, we focused on ATAC-seq and CUT&RUN levels after annotating genes in these two regions. We found that, from mouse oocytes to the zygote stage, higher chromatin accessibility in promoters correlated with higher H4K20me1 signals. In contrast, at the late 2-cell stage, chromatin accessibility in these promoter regions was markedly reduced, which then converted to higher chromatin accessibility but with lower H4K20me1 signal enrichment (Fig. 4A,B). We classified this as a type 1 “Promoter lost” region (Fig. 4A). Remarkably, the enrichment of H4K20me1 in the promoters is also rapidly reduced from late 2-cell embryos.

Unlike the case of promoters, the gene body regions of H4K20me1 were consistently and strongly positively correlated with chromatin accessibility, and genes with higher levels of H4K20me1 had higher chromatin accessibility (Fig. 4C,D). This relationship was retained throughout the period from oocytes to preimplantation embryos. However, the relationship between high-opening chromatin status and genes with higher H4K20me1 enrichment levels was more pronounced in the zygote to 8-cell embryos (Fig. 4C). This is consistent with the shifting of H4K20me1 enrichment towards gene body regions in the genome starting from late 2-cell embryos, which we classify as a type 2 “Gene body gain” region (Fig. 4A). Further analysis specifically focusing on intronic regions confirmed that this positive correlation remains highly robust (Fig. EV4D). With these results, we showed a significant positive correlation between retained chromatin accessibility and H4K20me1 in mouse oocytes and preimplantation embryos.

### H4K20me1 is required in early embryonic development

**Figure 5:**
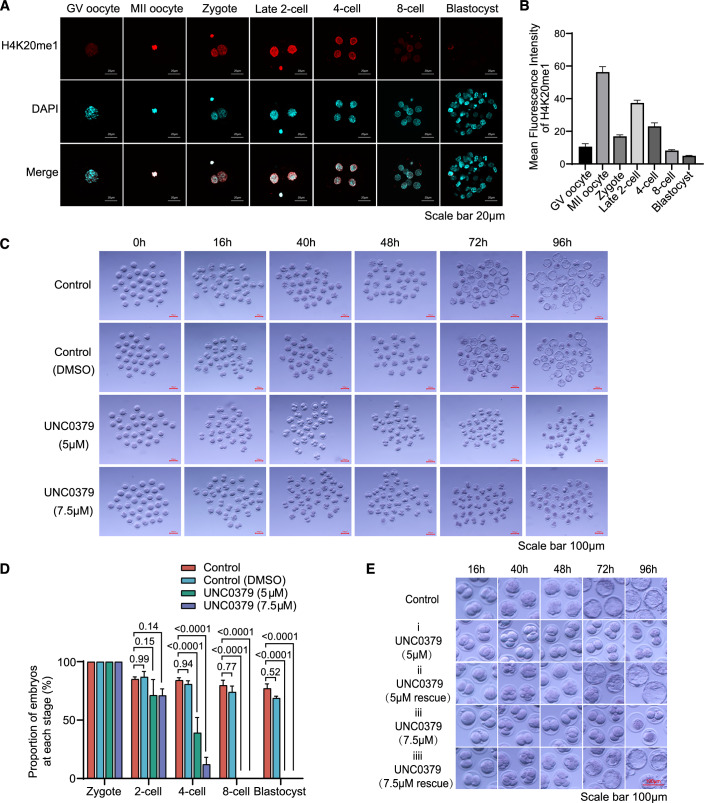
Inhibition of H4K20me1 in early embryos leads to arrest of embryonic developmental processes. (**A**) Immunofluorescence images showing H4K20me1 (red), DAPI-stained nuclei (cyan), and the merged signals in mouse oocytes and early embryos. Scale bar: 20 µm. (**B**) Mean fluorescence values of H4K20me1 levels in mouse oocytes and early embryos. The experiments were independently repeated three times, and the total number of embryos analyzed per developmental stage was as follows: GV oocyte (23), MII oocyte (29), zygote (32), 2-cell (26), 4-cell (36), 8-cell (47), and blastocyst (30). Data are presented as mean ± SEM. (**C**) Representative images of embryos from the Control, DMSO control, 5 μM UNC0379, and 7.5 μM UNC0379 groups are shown at the indicated time points after treatment. Scale bar: 100 µm. (**D**) The proportions of embryos at each stage in the standard medium control, DMSO control, 5 µM UNC0379, and 7.5 µM UNC0379 groups. Embryos were cultured in vitro and assessed at the indicated time points: zygote (0 h), 2-cell (16 h), 4-cell (40 h), 8-cell (48 h), and blastocyst (72–96 h). The experiment was independently repeated three times. Total embryo numbers per group: Control, *n* = 89; DMSO, *n* = 72; 5 µM UNC0379, *n* = 88; 7.5 µM UNC0379, *n* = 90. Data are shown as mean ± SEM. Statistical analysis was performed using two-way ANOVA with a multiple comparisons test. (**E**) Representative images of embryos from the Control, 5 μM UNC0379, 7.5 μM UNC0379, and rescue groups are shown at the indicated time points after treatment. Scale bar: 100 µm. Source data are available online for this figure.

**Figure EV5:**
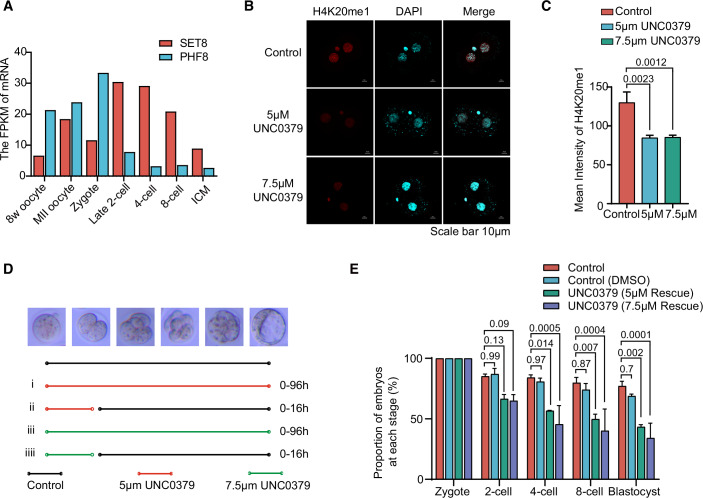
Inhibition of H4K20me1 in early embryos leads to arrest of embryonic developmental processes. (**A**) Expression of the H4K20me1-specific methylase SET8 as well as the demethylase PHF8 in mouse oocytes and early embryos. RNA-seq data from GSE71434. (**B**) Representative immunofluorescence images of 2-cell embryos under different conditions (Control, 5 µM UNC0379, 7.5 µM UNC0379). H4K20me1 (red) and DNA stained with DAPI (cyan) are shown individually and in a merge. Scale bar: 10 µm. (**C**) Mean fluorescence values of H4K20me1 levels in 2-cell embryos under different conditions (*n* = 7 embryos per group). Statistical analysis was performed using the Mann–Whitney *U* test. (**D**) Overview of in vitro experimental design. (**E**) The proportions of embryos at each stage in control, DMSO control, 5 µM UNC0379 rescue, and 7.5 µM UNC0379 rescue groups. Embryos were cultured in vitro and assessed at zygote (0 h), 2-cell (16 h), 4-cell (40 h), 8-cell (48 h), and blastocyst (72–96 h). The experiment was independently repeated three times. Total embryos per group: Control, *n* = 89; DMSO, *n* = 72; 5 µM UNC0379 rescue, *n* = 91; 7.5 µM UNC0379 rescue, *n* = 85. Data are shown as mean ± SEM. Statistical analysis was performed using two-way ANOVA with a multiple comparisons test. Source data are available online for this figure.

By immunofluorescence, we observed that H4K20me1 was highest in MII oocytes, decreased at the zygote, and increased at the late 2-cell stage (Fig. 5A,B). The expression of SET8, the only known lysine methyltransferase capable of mediating H4K20me1 according to current studies (Fang et al, 2002; Pan et al, 2022; Xu et al, 2022), showed a consistent trend in both oocytes and preimplantation embryos, with markedly higher expression in late 2-cell embryos (Fig. EV5A). Meanwhile, the expression of the H4K20me1 demethylase PHF8 was lower in preimplantation embryos and showed an opposite trend to that of the methyltransferase SET8 (Fig. EV5A), suggesting that SET8 is the primary regulator of H4K20me1 in preimplantation embryos. Therefore, the SET8 substrate competitive inhibitor UNC0379 was used to intervene in the embryos to reduce the H4K20me1 levels.

Treatment with two effective concentrations of UNC0379 led to inhibition of H4K20me1 and developmental arrest at the 2-cell stage (Figs. 5C,D and EV5B,C). Embryos cultured with UNC0379 failed to develop into blastocysts after 96 h of in vitro culture and either broke apart or died (Fig. 5C,D). To further determine that the 2-cell arrest was due to a decrease in H4K20me1, we set up a 16-h culture with the addition of UNC0379 and then removed the competitive inhibitor from the culture medium (Fig EV5D). We found that there existed embryos that were able to develop to the blastocyst stage, although the developmental process was delayed compared to the control group (Figs. 5E and EV5E). These results suggest that H4K20me1 may play a role in ZGA during embryonic development and confirm that it is essential for preimplantation embryonic development in mice.

### H4K20me1 promotes chromatin opening in early embryonic development

**Figure 6:**
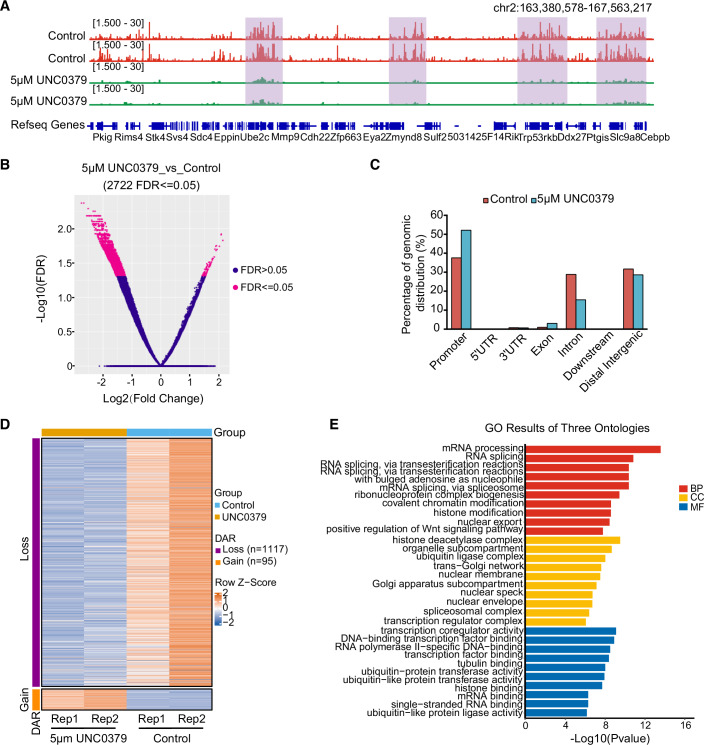
Loss of H4K20me1 in early embryos results in a reduction of chromatin-accessible regions. (**A**) IGV browser showing chromatin accessibility signals of the 5 μM UNC0379 group and the control group. The purple shading indicates the representative regions shown in the tracks. (**B**) ATAC-seq analysis comparing 5 μM UNC0379-treated embryos to controls. The volcano plot displays 2722 differentially accessible regions (DARs). (**C**) Percentage of chromatin-accessible regions in different genomic features covered by the 5 μM UNC0379 group and the control group. ATAC-seq peaks assigned to promoter (≤ 3 kb), 5’UTR, 3’UTR, exon, intron, downstream, and distal intergenic region. (**D**) Heatmap depicting the clustering of genes associated with DARs, with rows representing individual genes and columns representing samples from the control and UNC0379-treated groups. DARs were identified using DiffBind with DESeq2 (FDR < 0.05, FC > 2). Signal intensity is Z-score normalized. Genes are separated into two clusters: those with lost chromatin accessibility (*n* = 1117) and those with gained chromatin accessibility (*n* = 95). (**E**) GO enrichment analysis for genes associated with chromatin accessibility loss shown in (**D**). Statistical significance was determined using the hypergeometric test. Source data are available online for this figure.

**Figure EV6:**
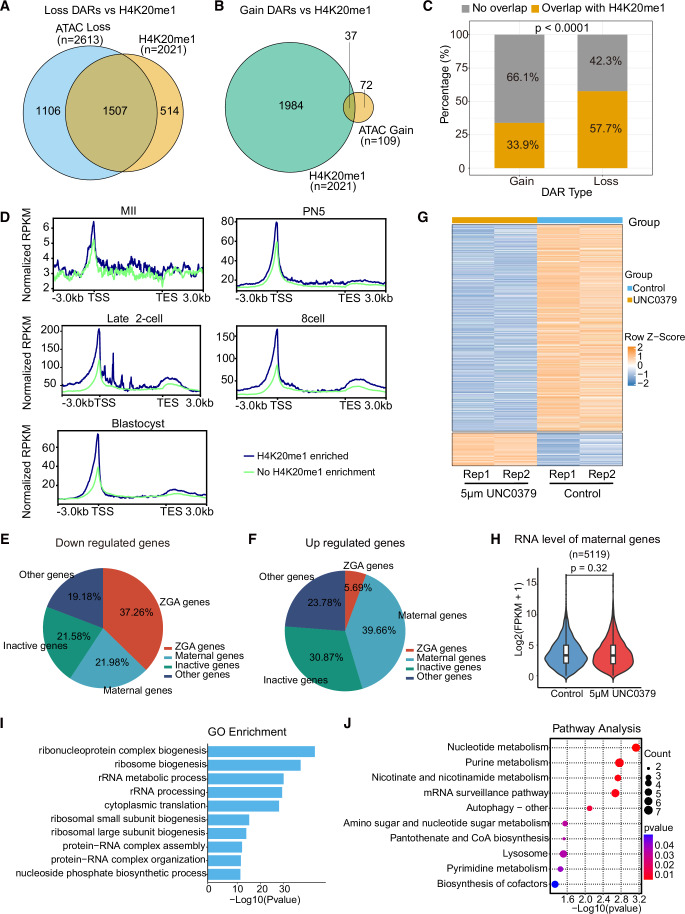
Loss of H4K20me1 in early embryos affects the expression of ZGA genes. (**A**, **B**) Venn diagrams showing the overlap between H4K20me1 peaks in control 2‑cell embryos and DARs with (**A**) lost accessibility or (**B**) gained accessibility upon H4K20me1 inhibition. Numbers indicate the counts of peaks or regions in each category and their intersection. (**C**) Stacked bar plots showing the proportion of Gain DARs (left) and Loss DARs (right) that overlap or do not overlap with H4K20me1 peaks. (**D**) Metaplot shows that Pol II is highly loaded at the locus where H4K20me1 is highly enriched. The data used to assess Pol II loading were obtained from the publicly available dataset GSE135457. The *y* axis represents the average signal density. (**E**, **F**) Pie charts showing the percentage composition of downregulated (**E**) and upregulated (**F**) genes, categorized as ZGA genes, maternal genes, inactive genes, and other gene types. (**G**) Heatmap showing expression levels of differentially expressed ZGA genes (*n* = 568) in control and UNC0379-treated samples. Signal intensity is Z-score normalized, with rows representing individual genes and columns representing samples (DESeq2, FDR < 0.05, FC > 2). (**H**) Violin plot showing no significant change in maternal gene expression between the control and 5 μM UNC0379 group. *n* denotes the number of genes included in each group. Box plots indicate the median (center line), 25th and 75th percentiles (box bounds), and whiskers extending to the minimum and maximum values. Statistical significance was determined using the Wilcoxon rank-sum test. (**I**) GO terms of ZGA genes in downregulated genes. (**J**) Kyoto Encyclopedia of Genes and Genomes (KEGG) analysis for downregulated genes. Source data are available online for this figure.

The establishment of a chromatin regulatory environment in preimplantation embryos has been one of the key concerns in the field of epigenetics. It is critically influenced by histone modifications, which alter chromatin accessibility and modulate transcription factor occupancy at genomic sites. Our data suggest that H4K20me1 in oocytes and early embryos may contribute to maintaining a more accessible chromatin state. To further investigate whether H4K20me1 promotes chromatin accessibility in early embryos, we inhibited H4K20me1 with UNC0379 at the pronuclear stage 5 (PN5) zygotes of mouse development during in vitro culture experiments. We obtained sufficient cell numbers of 2-cell embryos to perform ATAC-seq 16 h after the intervention (Fig. 6A). By comparing the ATAC-seq peaks between experimental and control-treated embryos, we identified 2722 differential accessible regions (DARs), of which 2613 DARs showed a loss of accessibility (Fig. 6B). These DARs were distributed across the genome, and regions with reduced accessibility were mainly enriched in introns (Fig. 6C). This finding is consistent with the observation that the genome coverage of H4K20me1 peaks in 2-cell stage embryos was shifted towards introns. Genome-wide analysis revealed that a majority (57.7%) of these loss-of-accessibility DARs overlapped with H4K20me1 peaks, which constituted 74.6% of all H4K20me1 peaks in controls (Fig. EV6A–C), supporting a specific role for H4K20me1 in maintaining chromatin accessibility. Taken together, our results suggest that the enrichment of H4K20me1 promotes chromatin opening in these regions.

Previous studies have shown that SET8 and H4K20me1 are more directly involved in promoting transcription by relieving promoter proximal pauses in RNA polymerase subunit 2 (Kapoor-Vazirani and Vertino, 2014; Nikolaou et al, 2017). We classified the genes of DARs into two groups: chromatin open region of loss and chromatin open region of gain. GO analysis of the “Chromatin open region of loss” group showed that the genes reduced chromatin accessibility after the suppression of H4K20me1 are involved in RNA splicing and processing, histone modification, and transcription processes (Fig. 6D,E). Furthermore, genes enriched with H4K20me1 in late 2-cell embryos also showed higher loading of Pol II (Data ref: Liu et al, 2020) in the TSS and TES regions (Fig. EV6D). These data suggest a function for H4K20me1 in establishing an accessible chromatin environment that promotes RNA synthesis and transcription.

### Reduction of genomic H4K20me1 in embryos after fertilization leads to failure of zygotic genome activation and abnormal gene expression

To understand the mechanisms behind developmental arrest in embryos with inhibited H4K20me1, we dissected the developmental stages of embryonic arrest. The majority of embryos cultured with UNC0379 were arrested at the 2-cell and 4-cell stages. This distribution suggests that reduced H4K20me1 affects the early developmental stages at which epigenetic reprogramming occurs. From the results of CUT&RUN, H4K20me1 was strongly associated with the ZGA process in early embryos. Following SET8 suppression, H4K20me1 was lost in the genome, and mouse development was arrested at the ZGA stage. To further explore the mechanism by which H4K20me1 affects early development, we performed RNA-seq analysis on embryos arrested at the 2-cell stage. The ZGA genes were screened based on RNA-seq data from mouse germ cells and preimplantation embryos in the GEO database (Data ref: Zhang et al, 2016). The screening principle defined ZGA genes as those with high expression at the late 2-cell stage (FPKM > 5) but low expression in MII oocytes (FPKM < 5), while maternal genes are defined as those with high expression in MII oocytes (FPKM > 5) (Liu et al, 2020).

**Figure 7:**
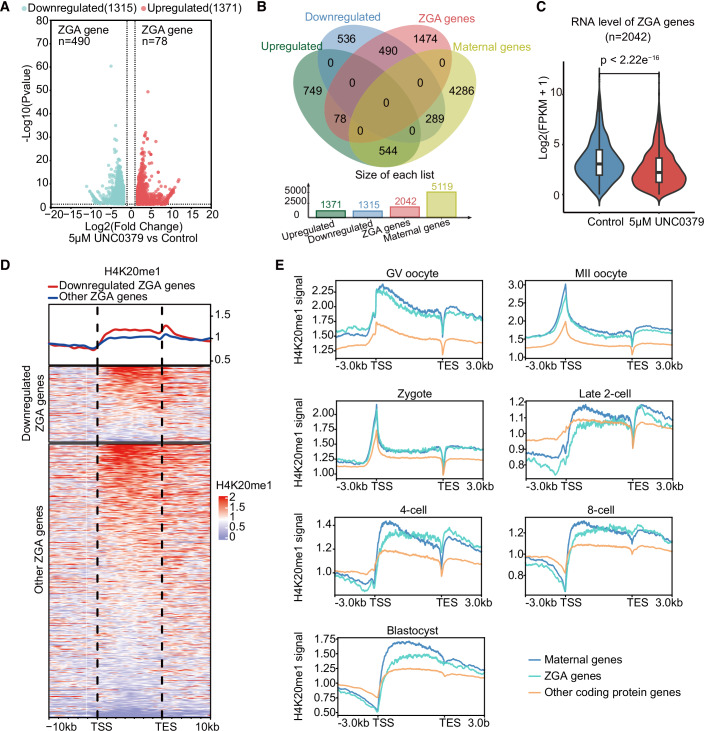
Loss of H4K20me1 in early embryos affects the expression of ZGA genes. (**A**) RNA-seq analysis of 2-cell embryos from the control and 5 μM UNC0379-treated group. Volcano plot displays changes in gene expression. Red and blue dots indicate significantly upregulated and downregulated genes, respectively (DESeq2, FDR < 0.05, FC > 2). (**B**) Venn diagram illustrating overlaps among ZGA genes, maternal genes, upregulated genes, and downregulated genes following H4K20me1 inhibition. The total number of genes in each category is indicated. (**C**) Violin plot showing significantly downregulated expression of ZGA genes in the 5 μM UNC0379 group compared to the control. *n* denotes the number of genes included in each group. Box plots indicate the median (center line), 25th and 75th percentiles (box bounds), and whiskers extending to the minimum and maximum values. Statistical significance was determined using the Wilcoxon signed-rank test. (**D**) Metaplot shows that downregulated ZGA genes of (**A**) occupy a higher H4K20me1 enrichment than other ZGA genes. The *y* axis represents the average H4K20me1 signal density across genes. (**E**) Metaplot shows that maternal genes and ZGA genes exhibit higher H4K20me1 enrichment than other protein-coding genes in mouse oocytes and early embryos. The *y* axis represents the average signal density across genes. Source data are available online for this figure.

Differential gene expression analysis between the H4K20me1-inhibited group and the control group (fold change >2, FDR < 0.05) revealed that loss of H4K20me1 resulted in the upregulation of 1371 genes and the downregulation of 1315 genes (Fig. 7A). Among the upregulated genes upon H4K20me1 inhibition, we identified 78 ZGA genes (5.7%) and 544 maternal genes (39.7%). Among the downregulated genes, there are 490 ZGA genes (37.3%) and 289 maternal genes (22.0%) (Figs. 7B and EV6E,F). ZGA genes are significantly downregulated, indicating that H4K20me1 affects ZGA gene expression (Figs. 7C and EV6G). The remaining differentially expressed genes consist of constantly inactive genes (FPKM < 5 in all stages) and stage-specific genes that are normally activated at other developmental time points. Among the upregulated genes, maternal genes and inactive genes constitute primary categories, accounting for 39.7% and 30.9%, respectively (Fig. EV6E,F). However, the overall expression level of maternal genes is not significantly altered (Fig. EV6H).

We focused on the H4K20me1 enrichment patterns of these affected ZGA genes in 2-cell embryos and found that the downregulated ZGA genes, following the reduction of H4K20me1, exhibited higher H4K20me1 enrichment levels (Fig. 7D). It was further found that there was also higher H4K20me1 enrichment in ZGA genes among embryos from the oocyte to zygote stage (Fig. 7E). GO analysis of the downregulated ZGA genes showed that their functions were mainly enriched in ribosome biogenesis and mRNA processing (Fig. EV6I). Kyoto Encyclopedia of Genes and Genomes (KEGG) analysis showed that downregulated genes were associated with pathways such as nucleotide metabolism, purine metabolism, and mRNA surveillance (Fig. EV6J). In addition, H4K20me1-enriched genes in the embryos also showed higher Pol II loads in the TSS and TES regions (Fig. EV6D). Therefore, embryos can be arrested or even die during development due to H4K20me1 abnormality, leading to disruption of transcription and translation processes. In contrast, H4K20me1 promotes ZGA gene expression in early mouse embryonic development by facilitating chromatin opening while regulating the transition of RNA Pol II from the initiation or pause state to the active elongation stage.

## Discussion

In addition to DNA, the main genetic material, chromatin carries a large amount of epigenetic information that is transmitted from parent to offspring. Along with the progression of early embryonic development, maternal mRNAs and proteins are gradually degraded (Vastenhouw et al, 2019; Sha et al, 2020; Jiang and Fan, 2022), the zygotic genome is activated (Schulz and Harrison, 2019; Chen et al, 2023), and the totipotent embryo develops into an individual. During this process, epigenetic information is essential for the precise regulation of gene expression and normal embryonic development (Liu et al, 2016; Zheng et al, 2016; Shen et al, 2015). Understanding how epigenetic information of parental origin is retained and erased in the zygote, and how the zygote accomplishes epigenetic reconstruction, has been a pressing question in the field. In addition, identifying the factors that regulate zygotic genome activation has been a key concern.

As an important epigenetic marker, the genome-wide distribution and reprogramming of H4K20me1 during preimplantation development remain largely unknown. Our study identified both conserved and divergent patterns of H4K20me1 distribution in mouse oocytes and early embryos, as well as in human and zebrafish early embryos. We found that H4K20me1 of all three model organisms is distributed across the genome in a broad peak pattern.

H4K20me1 is highly enriched in mouse oocytes, with its levels undergoing dynamic changes after fertilization: the majority is erased, while a portion is retained or reestablished either post-fertilization or during the late 2-cell stage. H4K20me1 at promoters is predominantly erased starting from the late 2-cell stage, whereas its enrichment in gene bodies begins to be reestablished during the same period, coinciding with the timing of ZGA. Notably, the apparent nuclear H4K20me1 signal detected by immunofluorescence at the 2-cell stage indicates its retention during this key period. The decrease in locus-specific peaks observed by CUT&RUN may thus reflect not a loss of function, but a transition in its regulatory mode—a spatial redistribution from a broad pattern to a more precisely targeted configuration at select genomic sites, potentially to execute stage-specific functions. These findings suggest that, in preimplantation embryos, H4K20me1 is functionally linked to gene bodies and likely contributes to the regulation of ZGA.

In contrast to the dynamic changes observed in early mouse development, human embryos maintain relatively low H4K20me1 levels at the 4-cell and 8-cell stages, with a substantial increase at the blastocyst stage, suggesting its potential role in lineage differentiation during early development. We note that the relatively lower correlation between replicates at these stages is likely attributable to limited sample availability and technical variability, which is also reflected in the smaller number of peaks called. Nonetheless, the overall trend of low H4K20me1 signal prior to the blastocyst stage remains robust and is further supported by immunofluorescence data.

H4K20me1 peaks in zebrafish exhibit a gradual increase in genomic enrichment starting from the 64-cell stage, peaking at the 1k-cell stage, and subsequently declining. Notably, ZGA occurs relatively late in human and zebrafish, at around the 8-cell stage (Burton and Torres-Padilla, 2025) and 1k-cell stage (Zhang et al, 2018a), respectively. Therefore, H4K20me1 may also play a potential regulatory role during ZGA in these species. In addition, unlike its distribution in early mouse embryos, H4K20me1 peaks in zebrafish are primarily enriched in distal intergenic regions rather than gene bodies. In humans, the enrichment of H4K20me1 peaks shifts from distal intergenic regions to promoters and gene bodies from the 4-cell embryos to the blastocysts. These distinct distribution patterns highlight differences in the regulatory mechanisms and functional conservation of H4K20me1 during embryonic development, emphasizing its species-specific roles.

Previous studies have reported that SET8-mediated H4K20me1 is involved in maintaining genomic integrity and is essential for preimplantation development in mice (Shikata et al, 2020). While this essential role is established, the precise regulatory mechanisms, particularly the dynamics of H4K20me1 and its role in transcriptional and chromatin regulatory programs during the maternal-to-zygotic transition, remain to be fully elucidated. In this study, we inhibited the specific methyltransferase SET8 by treating early mouse embryos with its substrate competitive inhibitor UNC0379, which significantly reduced H4K20me1 levels. The loss of H4K20me1 led to developmental arrest at the 2-cell and 4-cell stage, accompanied by the downregulation of numerous ZGA genes and the upregulation of maternal genes. Furthermore, the reduction of H4K20me1 resulted in abnormal ribosome function, dysregulation of various RNA processing pathways, and aberrant expression of ZGA-associated genes, ultimately causing developmental arrest in early embryos. These findings indicate that, although the number of H4K20me1 peaks decreases after fertilization in mouse oocytes, the remaining H4K20me1 still plays a critical role in regulating ZGA.

It is important to note that SET8 is the sole methyltransferase for H4K20me1, which is a prerequisite for the formation of H4K20me2/3. Thus, the developmental arrest and transcriptional defects we observe upon SET8 inhibition could theoretically stem from the loss of H4K20me1, a secondary decrease in H4K20me2/3, or both. However, a previous study showed that H4K20me2/3 levels are remarkably low or stage-specific during the early cleavage stages in mouse embryos, with H4K20me2 absent at the one- and two-cell stages and H4K20me3 only detectable in the maternal pronucleus of one-cell embryos (Wongtawan et al, 2011). Therefore, while a contribution from diminished H4K20me2/3 cannot be formally excluded, the severe phenotype we observed at the early cleavage stages, coincident with major ZGA, strongly suggests that the presence of H4K20me1 itself is critical for this developmental transition.

H4K20me1 is important for establishing proper chromatin structure during normal embryonic stem cell maintenance and embryonic development (Oda et al, 2009). It has been shown to directly promote chromatin openness by disrupting chromatin folding in U2OS cells, thereby facilitating the transcription of housekeeping genes (Shoaib et al, 2021). H4K20me1 is also recognized for its essential function in chromatin compaction during X chromosome inactivation (Tjalsma et al, 2021). To further investigate the relationship between H4K20me1 and chromatin accessibility, we analyzed its distribution in early mouse embryos. Our observations revealed that as H4K20me1 levels on promoters decreased, the high correlation with chromatin accessibility diminished, and the phenomenon of higher chromatin accessibility in genes with high H4K20me1 enrichment disappeared. In contrast, gene bodies with high H4K20me1 coverage consistently maintained a strong correlation with chromatin accessibility. Reduction of H4K20me1 levels in embryos resulted in a significant loss of chromatin openness, primarily in gene bodies enriched with H4K20me1. These findings suggest that H4K20me1 plays a role in promoting or maintaining chromatin openness during early development. Furthermore, the biological functions of genes affected by this chromatin accessibility are closely associated with transcriptional co-regulator activity, transcription factor binding, and RNA polymerase II-specific DNA-binding transcription factors. Additionally, H4K20me1 is essential for the release of Pol II into active elongation, and our data further confirmed that genes with high H4K20me1 enrichment exhibit higher Pol II occupancy. Therefore, H4K20me1 likely facilitates transcriptional regulation during early development by promoting chromatin accessibility, enabling the recruitment of transcriptional regulators to the genome. Abnormal levels of H4K20me1 in early embryos may lead to reduced chromatin accessibility, impaired recruitment of transcriptional regulators, and disrupted de novo RNA synthesis, ultimately resulting in the failure of ZGA, developmental arrest, and even embryonic lethality. However, some questions remain, such as how chromatin openness influences the deposition or erasure of H4K20me1, and the mechanisms underlying the interaction between H4K20me1 and Pol II. The interplay between H4K20me1, chromatin accessibility, and ZGA in early embryos requires further elucidation.

In summary, our study revealed the distribution and dynamic changes of H4K20me1 during oocyte maturation and preimplantation embryonic development, and uncovered its potential roles in regulating chromatin accessibility and ZGA. These findings enhance our understanding of the mechanisms by which histone modifications influence early embryonic development and provide new insights into the epigenetic regulation of ZGA.

## Methods

**Table:** Reagents and tools table

Reagent/resource	Reference or source	Identifier or catalog number
**Experimental models**		
C57BL/6 N (*Mus musculus*)	Beijing Vital River Laboratory Animal Technology Co., Ltd., Beijing, China	
AB strain (*Danio rerio*)	Jiawei Xu Lab	
K562 cells (*Homo sapiens*)	Procell Life Science & Technology Co., Ltd., Wuhan, China	CL-0130
Human embryos (*Homo sapiens*)	Luoyang Maternal and Child Health Hospital, Luoyang, China	
**Recombinant DNA**		
N/A		
**Antibodies**		
Rabbit anti-H4K20me1 pAb	Abcam	Cat # Ab9051
Alexa FluorTM 568 donkey anti-rabbit IgG (H + L) pAb	Invitrogen	Cat # A10042
**Oligonucleotides and other sequence-based reagents**		
Adapters	New England Biolab	Cat # E7645S
Primers	Vazyme	Cat # TD502, N712
**Chemicals, enzymes, and other reagents**		
4% Paraformaldehyde	Biosharp	Cat # BL539A
Bovine serum albumin	Sigma-Aldrich	Cat # 126615
Triton X-100	Sigma-Aldrich	Cat # T8787
DAPI	Invitrogen	Cat # S36968
UNC0379	Proteintech	Cat # CM04584
VAHTS DNA Clean Beads	Vazyme	Cat # N411
M2 culture medium	Sigma-Aldrich	Cat # M7167
G1 culture medium	Vitrolife	Cat # 10128
G2 culture medium	Vitrolife	Cat # 10132
Paraffin oil	Vitrolife	Cat # 10029
Pregnant Mouse Serum Gonadotropin	Solarbio	Cat # P9970
Human Chorionic Gonadotropin	Shanghai Livzon Pharmaceutical	Cat # H20033905
RPMI Medium 1640	Solarbio	Cat # 31800
Glutathione	Solarbio	Cat # G8180
DMSO (Dimethyl Sulfoxide)	Solarbio	Cat # D8371
Phosphate Buffered Saline	Solarbio	Cat # P1020
EDTA-free protease inhibitor	Roche Diagnostics	Cat # 04693132001
**Software**		
Bowtie (v2.2.2)	https://bowtie-bio.sourceforge.net/bowtie2/index.shtml	
Fastqc	https://www.bioinformatics.babraham.ac.uk/projects/fastqc/	
Trim-Galore	https://www.bioinformatics.babraham.ac.uk/projects/trim_galore/	
Picrad	https://broadinstitute.github.io/picard/	
Deeptools	https://github.com/deeptools	
Bedtools	https://github.com/arq5x/bedtools2	
Samtools	https://www.htslib.org/	
MACS3	https://pypi.org/project/MACS3/	
KEGG	https://www.genome.jp/kegg/	
Integrative Genomics Viewer (v2.6.2)	https://igv.org/	
Graphpad Prism 9	https://www.graphpad.com	
ImageJ 1.54p	https://imagej.net/ij/index.html	
**Other**		
NEBNext Ultra II DNA Library Prep Kit for Illumina	New England Biolab	Cat # E7645S
TruePrep DNA Library Prep Kit V2 for Illumina	Vazyme	Cat # TD502
Single Cell Full-Length mRNA-Amplification Kit	Vazyme	Cat # N712
Illumina NovaSeq 6000	Illumina	

### Ethical statement

Following the Measures of the People’s Republic of China on the Administration of Human Assisted Reproductive Technology and the Helsinki Declaration, this project was approved by the Institutional Review Boards (IRB) of The First Affiliated Hospital of Zhengzhou University (2024-KY-2104-003) and Luoyang Maternal and Child Health Hospital (SZSL2024071101), China. The study followed the Principles of Human Embryonic Stem Cell Ethics issued by the Ministry of Science and Technology and the Ministry of Health. It was regularly reviewed by the Ethics Committee for Scientific Research and Clinical Trials of the First Affiliated Hospital of Zhengzhou University, as well as the Luoyang Maternal and Child Health Hospital. All human embryos were obtained from the Centre for Reproductive Medicine, Luoyang Maternal and Child Health Hospital, and collected after informed consent was signed by volunteer couples. Couples are counseled that their gametes will be used to investigate histone modification dynamics in developing embryos, with the assurance that donation does not interfere with their in vitro fertilization-embryo transfer (IVF-ET) treatment.

### Mouse oocyte and embryo collection

Specific pathogen-free mice were housed in the Centre for Laboratory Animals of Zhengzhou University, China. All animal husbandry procedures followed the guidelines of the First Affiliated Hospital of Zhengzhou University Animal Care and Use Committee (Approval no. 2024-KY-2104-003). Male and female C57BL/6 N mice were purchased from Beijing Vital River Laboratory Animal Technology. In order to collect oocytes and early embryos, 4–5 weeks old females were subjected to superovulation by injection of 10 IU Pregnant Mouse Serum Gonadotropin (PMSG; Solarbio), followed 48 h later by 5 IU Human Chorionic Gonadotropin (hCG; Shanghai Livzon Pharmaceutical). GV oocytes were isolated from ovaries at 46–48 h after PMSG injection, and MII oocytes were collected at 12–14 h after hCG injection. Mouse embryos were obtained from superovulated females mated with C57BL/6 N males. The time points for in vivo collection of embryos after hCG injection were as follows: zygotes at 28 h, late 2-cell embryos at 45 h, 4-cell embryos at 54 h, 8-cell embryos at 60 h, and blastocysts at 90 h.

### Human embryo collection

All embryos were obtained from volunteers (aged 25–30) with normal karyotypes and no history of hereditary or infectious diseases or smoking. Triploid embryos (3PN), identified by the presence of three pronuclei at 18 h post-fertilization, were subsequently cultured to the 4-cell and 8-cell stages. Only morphologically high-grade 3PN embryos without developmental arrest were used in this study. The 3PN embryos were then vitrified and preserved in liquid nitrogen. Frozen embryos were thawed using a glass solution freezing device and cultured under the following conditions: in G-1 (Vitrolife) human embryo medium prior to the 8-cell stage and subsequently in G-2 (Vitrolife) medium until the blastocyst stage, at 37 °C with 6% CO_2_ and 5% O_2_.

### Zebrafish embryo collection

The wild-type zebrafish strain AB was used in this study. Wild-type zebrafish females and males (> 90 dpf) were reared under the following conditions: water temperature (24–30 °C), zebrafish room temperature (25–27 °C), pH (7–8), conductivity (200–1700 uS/cm), and photoperiod (14 h light/10 h dark). For embryo collection, healthy, non-lean females and males were selected and fed with halophilic saline for 30 min. They were then placed in a 1:1 ratio in a set-up tank separated by a divider. The next morning, between 8:00 and 9:00 a.m., the dividers was removed, and embryos were collected after waiting 15–30 min for completion of fertilization. The embryos were cultured in culture water containing methylene blue and placed in an incubator at 28.5 °C. After development to the 64-cell, 256-cell, 1K-cell, high, oblong, sphere, and dome stages, the embryos were washed 2–3 times with Hanks buffer and collected. All zebrafish rearing and experimental procedures followed the approval of the Animal Welfare and Ethical Review Body of the First Affiliated Hospital of Zhengzhou University (2023-KY-1419).

### Cell culture

Human chronic myeloid leukemia K562 cells were maintained in RPMI-1640 medium supplemented with 10% fetal bovine serum under standard culture conditions (37 °C, 5% CO_2_, saturated humidity) until the cell line was stably passaged for use. After 2–3 stable passages, cells were centrifuged at 1000 rpm for 4 min and the supernatant was removed. Subsequently, cells were washed and resuspended in PBS, and then transferred to sterile, enzyme-free EP tubes for counting. Gradient suspensions containing 100, 200, 500, 1000, 5000, and 10,000 cells were obtained. The cell line was validated using short tandem repeat sequences (STRs) and the routine mycoplasma assay.

### Immunofluorescence

The oocytes and embryos were fixed in 4% paraformaldehyde (PFA; Biosharp) for 10 min, and then repeatedly washed with 1% bovine serum albumin (BSA; Sigma) to remove the residual PFA on the surface of the cells. Samples were transferred into the 0.5% Triton X-100 (Sigma) for 30 min and subsequently washed five times with 1% BSA. Then, the samples were directly transferred into 1% BSA and blocked for 1 h at room temperature. After blocking, the samples were transferred into primary antibody (Abcam, ab9051; 1:500 dilution) and incubated overnight (>12 h) at 4 °C. The secondary antibody incubation solution was configured: Alexa FluorTM 568 donkey anti-rabbit IgG (H + L) (Invitrogen) was diluted 1:300 in 1% BSA and avoided the light. The samples were washed with 1% BSA 5 times and then incubated with the secondary antibody for 1 h at room temperature in the dark. After incubation, the samples were washed 5 times for 3 min, to completely wash off the residual secondary antibody on the cell surface. Finally, the cells were blocked with an antifade mounting medium containing DAPI (Invitrogen, S36968) and stored at 4 °C. Immunofluorescence images were obtained using a Zeiss 700 confocal microscope, maintaining consistent imaging parameters across all samples. A Z-stack consisting of five slices with a fixed step size was collected for each embryo. For visual presentation, these Z-stacks were merged into maximum intensity projections, and signals were pseudo-colored for enhanced visibility. Quantification was performed on representative single sections using ImageJ: images were converted to 8-bit grayscale and uniformly thresholded with the default algorithm to define regions of interest, and the mean intensity was measured.

### In vitro mouse embryo culture

Mouse embryos at the PN5 stage were obtained and washed 2–3 times with G-1 culture medium. The SET8 inhibitor buffer containing 5 μM or 7.5 μM UNC0379 was prepared by diluting a 10 mM UNC0379 working solution with G1/G2 culture medium. Morphologically normal and well-developed embryos were randomly transferred into the following culture conditions: G1 control group, DMSO control group, 5 μM UNC0379 group, or 7.5 μM UNC0379 group. The petri dishes were sealed with paraffin oil (Vitrolife) and incubated at 37 °C with 5% CO_2_. The culture medium was changed to G-2 medium when embryos developed to the 8-cell stage. Microscopic photographs were taken at 0, 16, 40, 48, 72, and 96 h of culture. For miniATAC and RNA-seq library construction after SET8 inhibitor intervention, embryos from the control and experimental groups (5 μM UNC0379) were cultured to the 2-cell stage and collected for library construction.

### CUT&RUN library preparation

CUT&RUN library construction was performed according to the published method (Skene and Henikoff, 2017; Skene et al, 2018; Hainer and Fazzio, 2019; Meers et al, 2019), with modifications and refinements for cell permeabilization, DNA purification, and DNA library construction. In brief, oocytes and early embryos were obtained, placed in M2 culture medium containing glutathione (GSH), and the zona pellucida was removed. Samples were washed in PBS and then transferred to 200 µL PCR tubes. ConA beads (BioMag Plus ConA Particles, Polysciences) were pre-washed four times with 1× Binding buffer and resuspended for use. Washed samples were incubated with 60 µL 1× Washing buffer (supplemented with protease inhibitor), then bound to 10 µL pre-cleaned ConA beads for 20 min at 25 °C with rotation (450 rpm). Bead-bound complexes were immobilized magnetically, and the supernatant was removed. Immunobinding was performed overnight at 4 °C using anti-H4K20me1 (Abcam, ab9051) diluted in antibody binding buffer (1× Washing buffer, 0.005% digitonin, 2 mM EDTA). Beads were washed with dig-wash buffer and then incubated with pA-MNase (Novoprotein, 1:500 in dig-wash buffer) for 1 h at 25 °C. After additional washes, added CaCl_2_ to 2 mM and incubated on ice for 30 min. The reaction was stopped with 2× STOR buffer containing digitonin, RNase A, and glycogen. Digestion was terminated at 37 °C for 25 min, and proteins were digested with Proteinase K at 56 °C for 45 min and 72 °C for 20 min after adding SDS and carrier RNA. Then, DNA was extracted using phase-lock gel tubes with phenol-chloroform-isoamyl alcohol, followed by chloroform cleanup. The aqueous phase was ethanol-precipitated with glycogen and sodium acetate, and the DNA pellet was stored at −20 °C or −80 °C. Finally, libraries were constructed using the NEBNext Ultra II DNA Library Prep Kit for Illumina (NEB, E7645S). The obtained fragments were purified and enriched with VAHTS DNA Clean Beads (Vazyme, N411) to sort out the fragments of the appropriate size for next-generation sequencing (GENEWIZ, NovaSeq 6000).

### miniATAC-seq library preparation

miniATAC-seq was carried out as previously described (Wu et al, 2018). Briefly, embryos at the 2-cell stage were removed from the zona pellucida using GSH (Solarbio), washed with PBS, and transferred to PCR tubes. Cells were lysed on ice with 6 µL ATAC lysis buffer (10 mM pH 7.4 Tris-HCl, 10 mM NaCl, 3 mM MgCl_2_, 0.5% NP-40) for 3 min. This step was repeated 3–4 times to ensure complete lysis. Next, transposon labeling and DNA fragmentation were performed by adding 5× TTBL and TTE Mix V5, followed by vortexing and incubation at 37 °C for 30 min. Subsequently, fragmentation was terminated with 5× TS for 5 min at room temperature. The obtained DNA fragments were purified by alcohol precipitation and stored at −20 °C or −80 °C. Libraries were constructed using the TruePrep DNA Library Prep Kit V2 for Illumina (Vazyme, TD502). Purified DNA was combined with 5× TAB, dual-index primers, and TAE. PCR amplification was performed under the following program: 72 °C for 3 min, 98 °C for 15 s, 60 °C for 30 s, 72 °C for 3 min (second to fourth steps of the cycle), 72 °C for 5 min. Amplified libraries were purified using VAHTS DNA Clean Beads (Vazyme, N411) for next-generation sequencing (GENEWIZ, NovaSeq 6000).

### RNA-seq library generation

In vitro treated experimental (5 μM UNC0379) and control 2-cell embryos were collected and washed 2–3 times with PBS. The embryos were then transferred to PCR tubes. Total RNA was lysed using RNA Lysis Buffer on ice for 30–40 min. The cDNA product was generated using the Single Cell Full-Length mRNA-Amplification Kit (Vazyme, N712) as follows: added Oligo(dT) VN Primer and dNTP Mix to the sample, and incubated at 72 °C for 3 min after initial ice incubation. Reverse transcription was performed with 1st Strand Buffer, DTT, RNase Inhibitor, 5’TS Oligo Primer, and Sc Reverse Transcriptase at 42 °C for 90 min, followed by inactivation at 70 °C for 15 min. Next, added PCR primers, 2× amplification mix, and purified the DNA fragments using VAHTS Clean Beads after the amplification of DNA fragments. Libraries were constructed from the amplified products with the TruePrep DNA Library Prep Kit V2 for Illumina (Vazyme, TD502) and sequenced on an Illumina NovaSeq 6000 platform (GENEWIZ) following standard protocols.

### CUT&RUN data processing

To remove the noise from the sequencing data, SOAPnuke (version 2.2.4) was used to filter the data with the following parameters: SOAPnuke filter -l 15 -q 0.5 -n 0.01 -Q 2 -c 21. After quality control, clean reads were compared with the reference genome (mm9, hg19 or danrer10) by Bowtie2 (version 2.3.4.3), and all reads matching to random chromosomes, unmatched reads, non-uniquely matched reads, poorly matched reads, and PCR repetitive sequences were removed, with the following parameters: --end-to-end --very- sensitive --no-mixed --no-discordant --phred33 -I 10 -X 700 -p 8. Duplicates due to PCR amplification were removed by Picard (v2.23.8), and to ensure the reliability of the analyses, only reads with a MapQ greater than 20 were used for subsequent analysis of the information. Peak Calling was performed with MACS3 (v3.0.0b1) with the following parameters: -p 1e-5 -f BEDPE --keep-dup all -B --SPMR -g 2.47e9 --broad --broad-cutoff 1e-5 -nolambda. Peak annotations were performed with the R package ChIPseeker (v1.30.3) and default parameters, annotating to gene bodies and functions. The bamCoverage tool in deepTools software was used to merge biological duplicate sample BAM files to calculate Counts Per Million mapped reads (CPM) and normalize them. The H4K20me1 CUT&RUN data were visualized with the Integrative Genomics Viewer (IGV, v2.6.2). Peaks in the promoter (−3 kb to TSS) and gene body (TSS to TES) regions were separately ranked by H4K20me1 signal intensity, and the top and bottom 25% within each region were defined as high- and low-enrichment peaks, respectively.

### RNA-seq data processing

The raw data obtained from RNA-seq sequencing was likewise first subjected to quality control to remove junctions and low-quality base sequences by FastQC (v0.11.9) and Fastp with the parameters -I -o -O -q 20 -w -h -j -D --detect_adapter_for_pe, and the resulting clean reads were aligned to the mm9 reference fundset by HISAT2 (v2.1.0). Gene-level read counts were quantified with FeatureCounts, and differential expression analysis was performed using DESeq2 (v1.43.0) with thresholds corrected for *p*-value < 0.05, FPKM > 1, FC > 2. Gene ontology analysis of differentially expressed genes was carried out with the “enrichGO” function from the ClusterProfiler (v4.2.2) package.

### ATAC-seq data processing

The high-throughput sequencing results of ATAC-seq libraries were quality filtered using Trimgalore (v0.6.6) with parameters: -q 20 -e 0.1 -paired. After quality control, the filtered reads were aligned to the reference genome using Bowtie2 (v2.4.4). The following types of reads were removed: reads matching random chromosomes, unmapped reads, non-uniquely mapped reads, poorly matched reads, and PCR repetitive sequences with parameters: -t -q -N 1 -L 25 -X 2000 --no-mixed --no-discordant. Downstream data were processed using Picard (v2.23.8) to remove duplicates due to PCR amplification. The read counts were then normalized by calculating the number of RPKM for 100 bp boxes of the genome. Further homogenization or normalization of RPKM values across the genome minimizes the effects of different experimental batches, sequencing depths, and gene lengths to get a true comparison. The experimental and control peaks were analyzed for differences using DiffBind (v3.0.14) software, and peaks with FC > 2 and FDR < 0.05 were considered significantly different chromatin accessibility peaks. The obtained differential peaks were annotated to gene bodies and functions by ChIPseeker (v1.30.3) and ClusterProfiler (v4.2.2). ATAC-seq data were visualized with the IGV Genome Browser. Chromatin accessibility distribution analysis using computeMatrix tool in deepTool software.

### Statistical analysis

Immunofluorescence intensity from in vitro cultured samples was quantified using ImageJ. Differences between two groups were evaluated using Student’s *t* test or Mann–Whitney *U* test for independent samples, and the Wilcoxon signed-rank test for paired comparisons. Embryo development rates under in vitro culture conditions were compared using two-way ANOVA with a multiple comparisons test. Statistical data were expressed as mean ± SEM in bar graphs. Data were analyzed using GraphPad Prism 9 (GraphPad Software, USA). All experiments were performed with at least three biological replicates, except for the omics sequencing, which was repeated twice.

### Graphics

The synopsis image was created in BioRender. Zhen H (2026) https://BioRender.com/htpk8yl.

## Supplementary information

Table EV1

Table EV2

Peer Review File

Source data Fig. 1

Source data Fig. 2

Source data Fig. 5

Source data Fig. 6

Source data Fig. 7

Figure EV3 Source Data

Figure EV5 Source Data

Figure EV6 Source Data

Expanded View Figures

## Data Availability

The raw sequence data generated in this study are available in the Genome Sequence Archive (Chen et al, 2021) in the National Genomics Data Center (CNCB-NGDC Members and Partners, 2022), China National Center for Bioinformation/Beijing Institute of Genomics, Chinese Academy of Sciences. The accession numbers are as follows: GSA: CRA026994 (https://ngdc.cncb.ac.cn/gsa/browse/CRA026994). GSA: CRA014047 (https://ngdc.cncb.ac.cn/gsa/browse/CRA014047). GSA-Human: HRA012004 (https://ngdc.cncb.ac.cn/gsa-human/browse/HRA012004). GSA-Human: HRA012044 (https://ngdc.cncb.ac.cn/gsa-human/browse/HRA012044). The source data of this paper are collected in the following database record: biostudies:S-SCDT-10_1038-S44319-026-00780-x.
