# Supplementary figures and images for: The reprogramming and function of H4K20me1 during early embryo development
Source: EMBO Rep. 2026 Apr 22;27(11):3050–77. doi: 10.1038/s44319-026-00780-x (PMC13261155; doi:10.1038/s44319-026-00780-x)

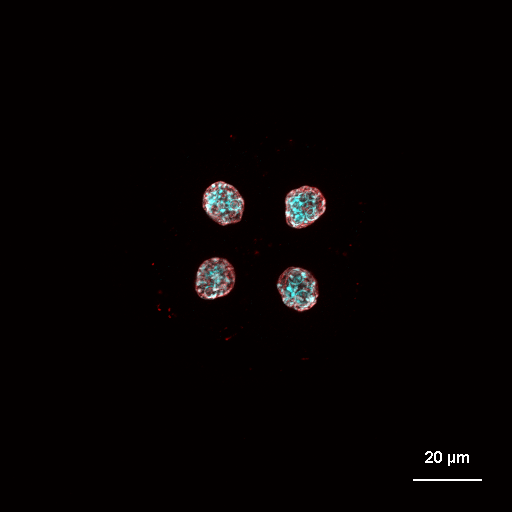

Supplement: Supplementary file 6 — Source data Fig. 5 [file 44319_2026_780_MOESM6_ESM.zip › Figure 5/Fig5A/4-cell/1017-MaxIP_hp_RGB.tif]

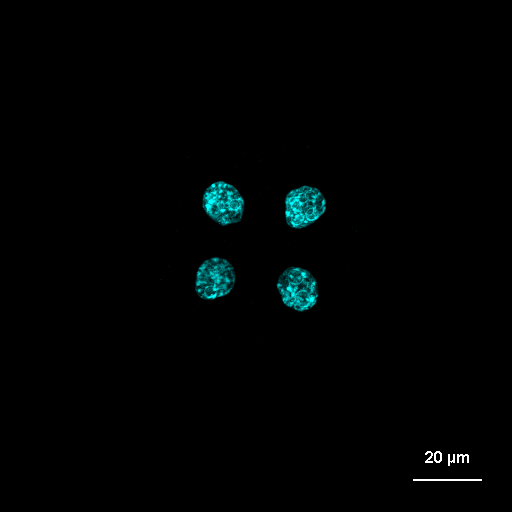

Supplement: Supplementary file 6 — Source data Fig. 5 [file 44319_2026_780_MOESM6_ESM.zip › Figure 5/Fig5A/4-cell/1017-MaxIP_hp_RGB_DAPI.tif]

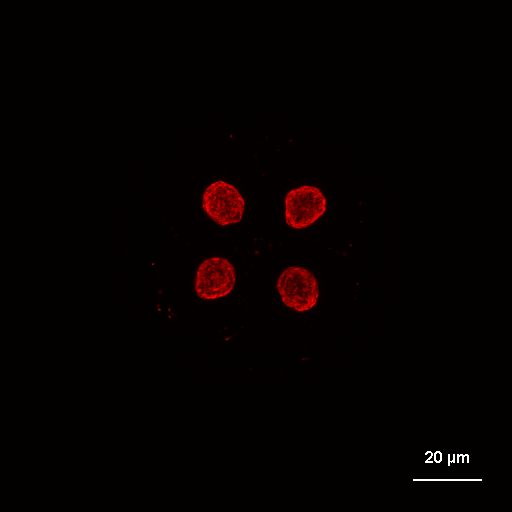

Supplement: Supplementary file 6 — Source data Fig. 5 [file 44319_2026_780_MOESM6_ESM.zip › Figure 5/Fig5A/4-cell/1017-MaxIP_hp_RGB_H4k20M1.tif]

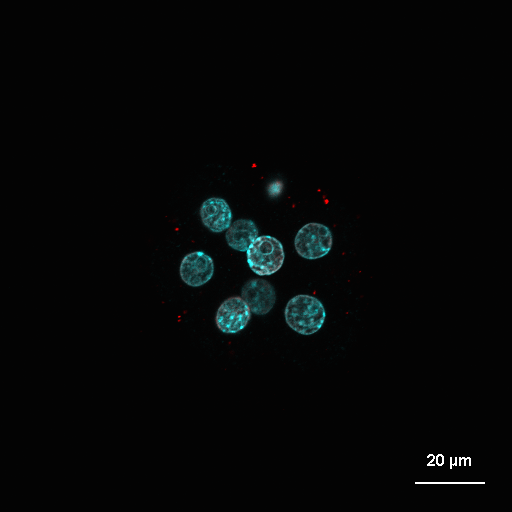

Supplement: Supplementary file 6 — Source data Fig. 5 [file 44319_2026_780_MOESM6_ESM.zip › Figure 5/Fig5A/8-cell/1029-MaxIP_hp_RGB.tif]

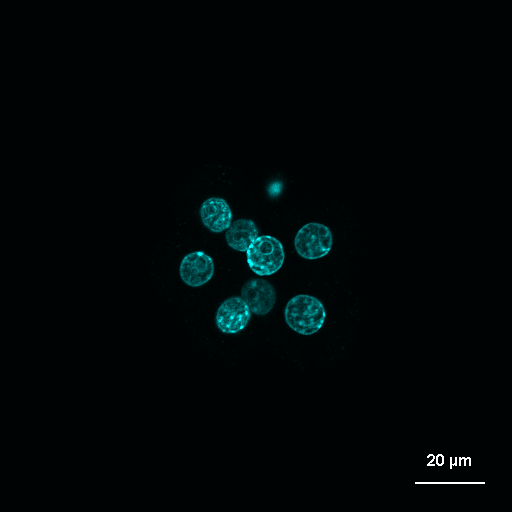

Supplement: Supplementary file 6 — Source data Fig. 5 [file 44319_2026_780_MOESM6_ESM.zip › Figure 5/Fig5A/8-cell/1029-MaxIP_hp_RGB_DAPI.tif]

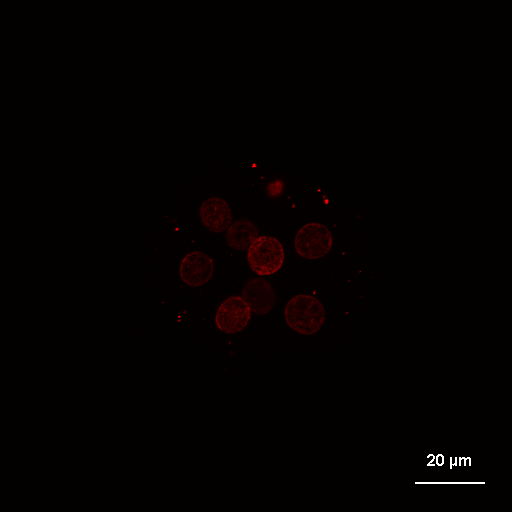

Supplement: Supplementary file 6 — Source data Fig. 5 [file 44319_2026_780_MOESM6_ESM.zip › Figure 5/Fig5A/8-cell/1029-MaxIP_hp_RGB_H4k20M1.tif]

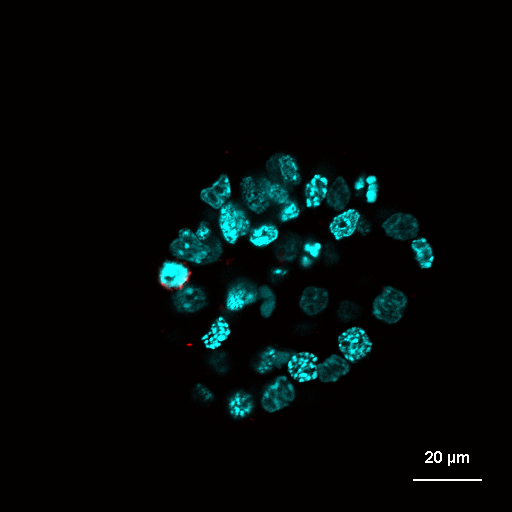

Supplement: Supplementary file 6 — Source data Fig. 5 [file 44319_2026_780_MOESM6_ESM.zip › Figure 5/Fig5A/Blastocyst/1009-MaxIP_hp_RGB.tif]

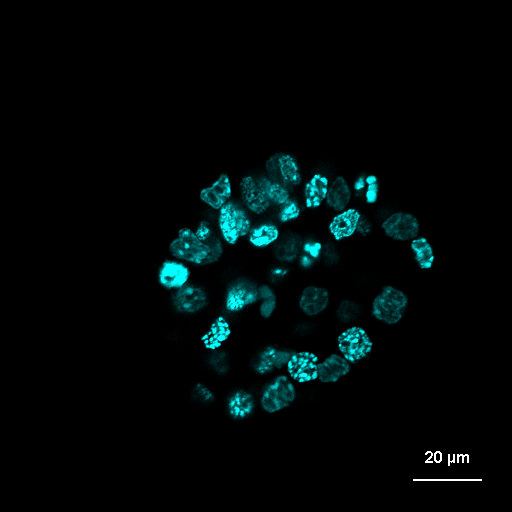

Supplement: Supplementary file 6 — Source data Fig. 5 [file 44319_2026_780_MOESM6_ESM.zip › Figure 5/Fig5A/Blastocyst/1009-MaxIP_hp_RGB_DAPI.tif]

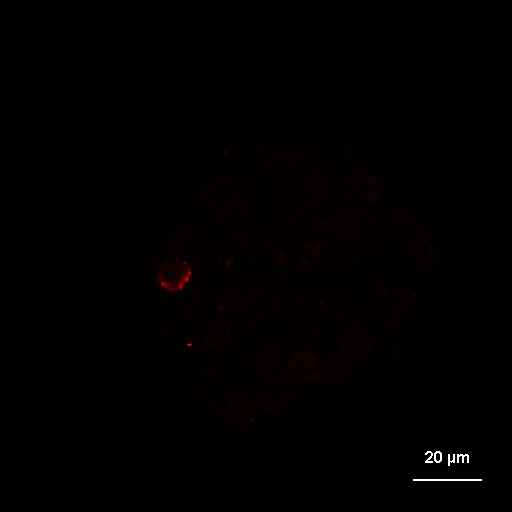

Supplement: Supplementary file 6 — Source data Fig. 5 [file 44319_2026_780_MOESM6_ESM.zip › Figure 5/Fig5A/Blastocyst/1009-MaxIP_hp_RGB_H4k20M1.tif]

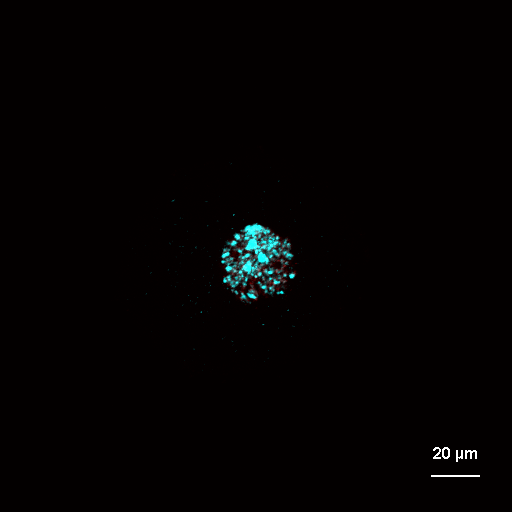

Supplement: Supplementary file 6 — Source data Fig. 5 [file 44319_2026_780_MOESM6_ESM.zip › Figure 5/Fig5A/GV oocyte/1001-MaxIP_hp_RGB.tif]

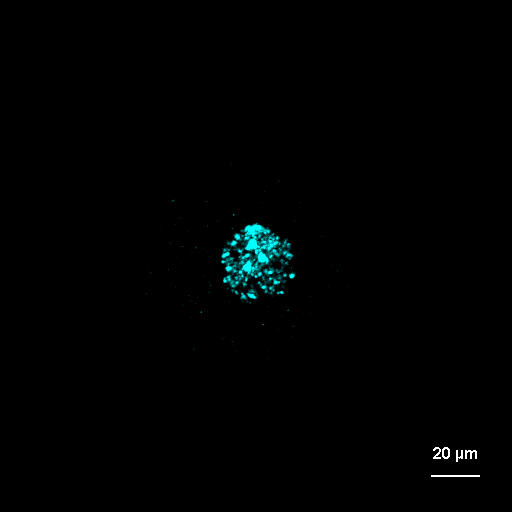

Supplement: Supplementary file 6 — Source data Fig. 5 [file 44319_2026_780_MOESM6_ESM.zip › Figure 5/Fig5A/GV oocyte/1001-MaxIP_hp_RGB_DAPI.tif]

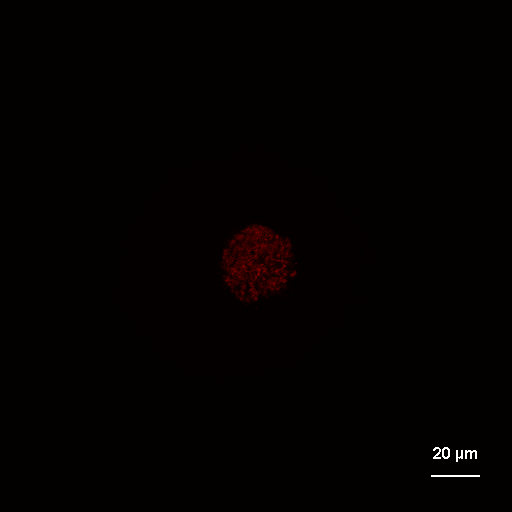

Supplement: Supplementary file 6 — Source data Fig. 5 [file 44319_2026_780_MOESM6_ESM.zip › Figure 5/Fig5A/GV oocyte/1001-MaxIP_hp_RGB_H4k20M1.tif]

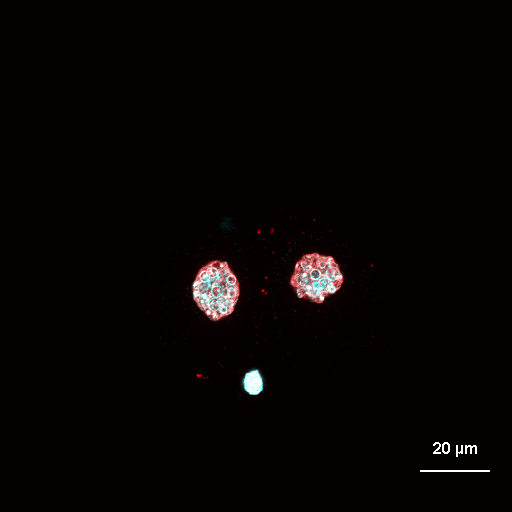

Supplement: Supplementary file 6 — Source data Fig. 5 [file 44319_2026_780_MOESM6_ESM.zip › Figure 5/Fig5A/Late 2-cell/1047-MaxIP_hp_RGB.tif]

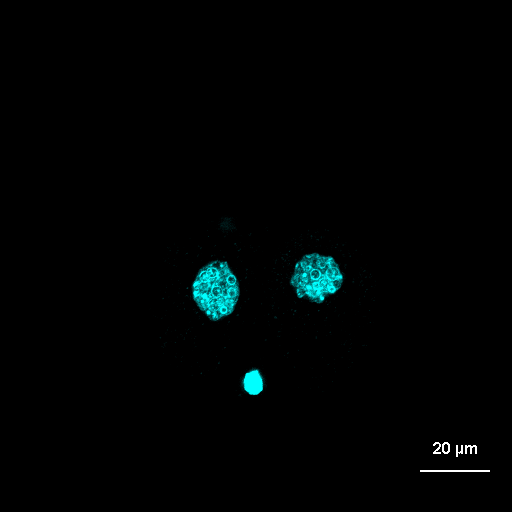

Supplement: Supplementary file 6 — Source data Fig. 5 [file 44319_2026_780_MOESM6_ESM.zip › Figure 5/Fig5A/Late 2-cell/1047-MaxIP_hp_RGB_DAPI.tif]

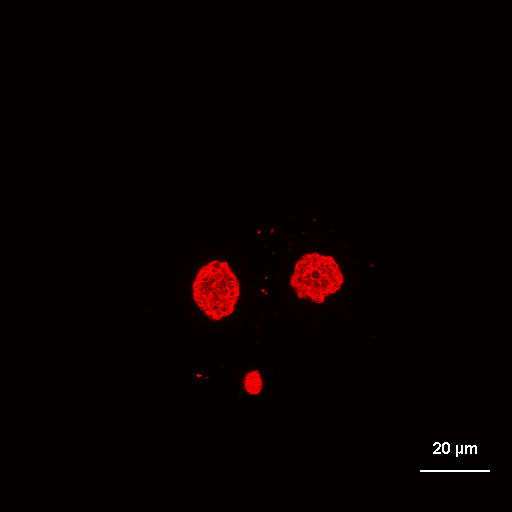

Supplement: Supplementary file 6 — Source data Fig. 5 [file 44319_2026_780_MOESM6_ESM.zip › Figure 5/Fig5A/Late 2-cell/1047-MaxIP_hp_RGB_H4k20M1.tif]

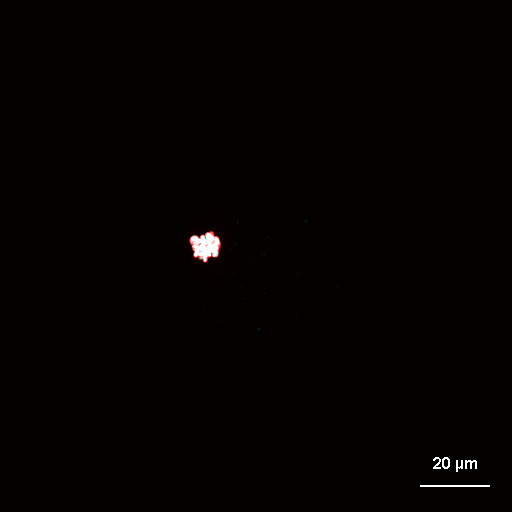

Supplement: Supplementary file 6 — Source data Fig. 5 [file 44319_2026_780_MOESM6_ESM.zip › Figure 5/Fig5A/MII oocyte/mii1017-MaxIP_hp_RGB.tif]

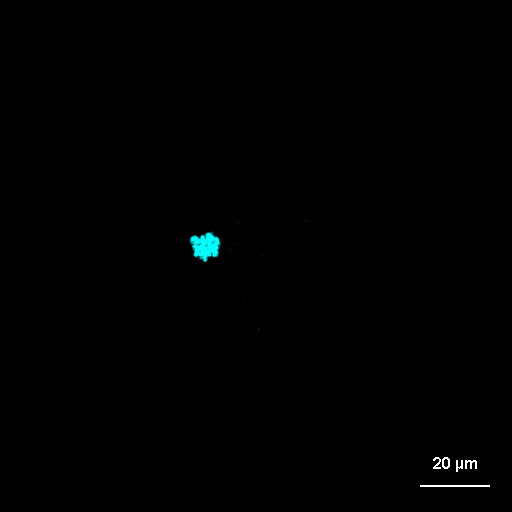

Supplement: Supplementary file 6 — Source data Fig. 5 [file 44319_2026_780_MOESM6_ESM.zip › Figure 5/Fig5A/MII oocyte/mii1017-MaxIP_hp_RGB_DAPI.tif]

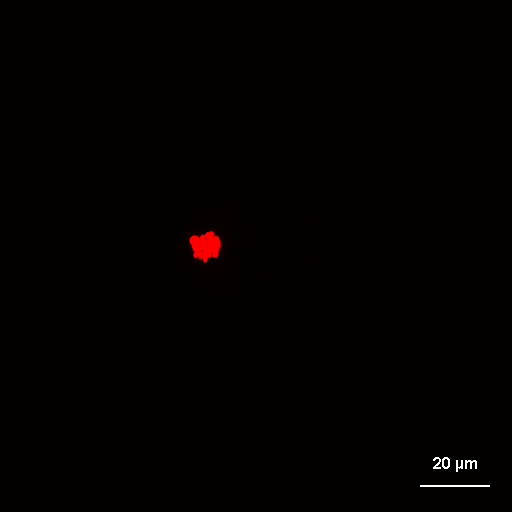

Supplement: Supplementary file 6 — Source data Fig. 5 [file 44319_2026_780_MOESM6_ESM.zip › Figure 5/Fig5A/MII oocyte/mii1017-MaxIP_hp_RGB_H4k20M1.tif]

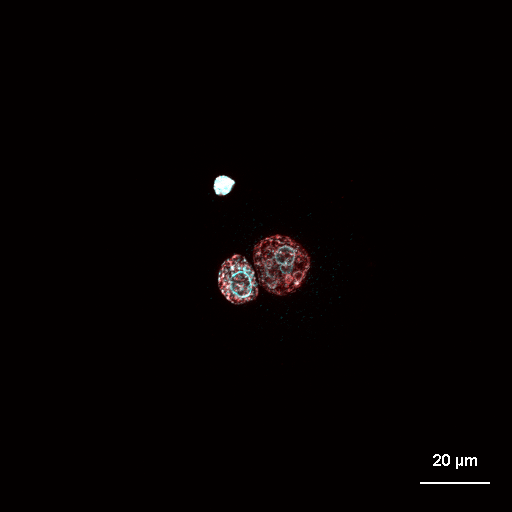

Supplement: Supplementary file 6 — Source data Fig. 5 [file 44319_2026_780_MOESM6_ESM.zip › Figure 5/Fig5A/Zygote/1039-MaxIP_hp_RGB.tif]

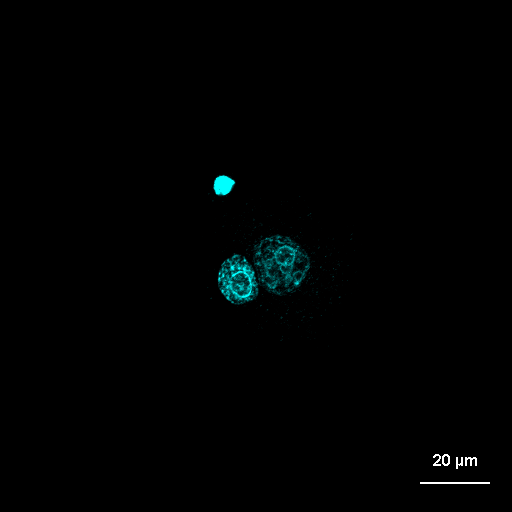

Supplement: Supplementary file 6 — Source data Fig. 5 [file 44319_2026_780_MOESM6_ESM.zip › Figure 5/Fig5A/Zygote/1039-MaxIP_hp_RGB_DAPI.tif]

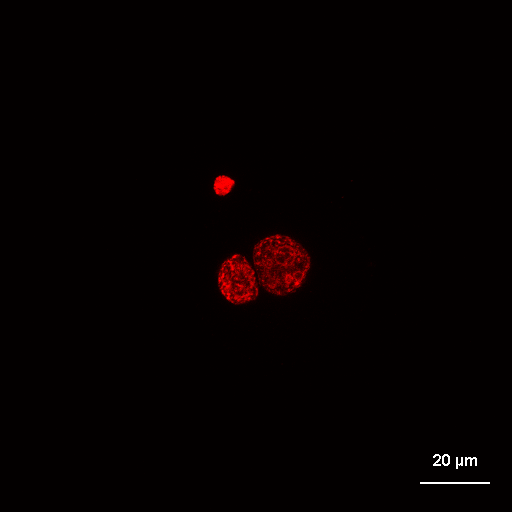

Supplement: Supplementary file 6 — Source data Fig. 5 [file 44319_2026_780_MOESM6_ESM.zip › Figure 5/Fig5A/Zygote/1039-MaxIP_hp_RGB_H4k20M1.tif]

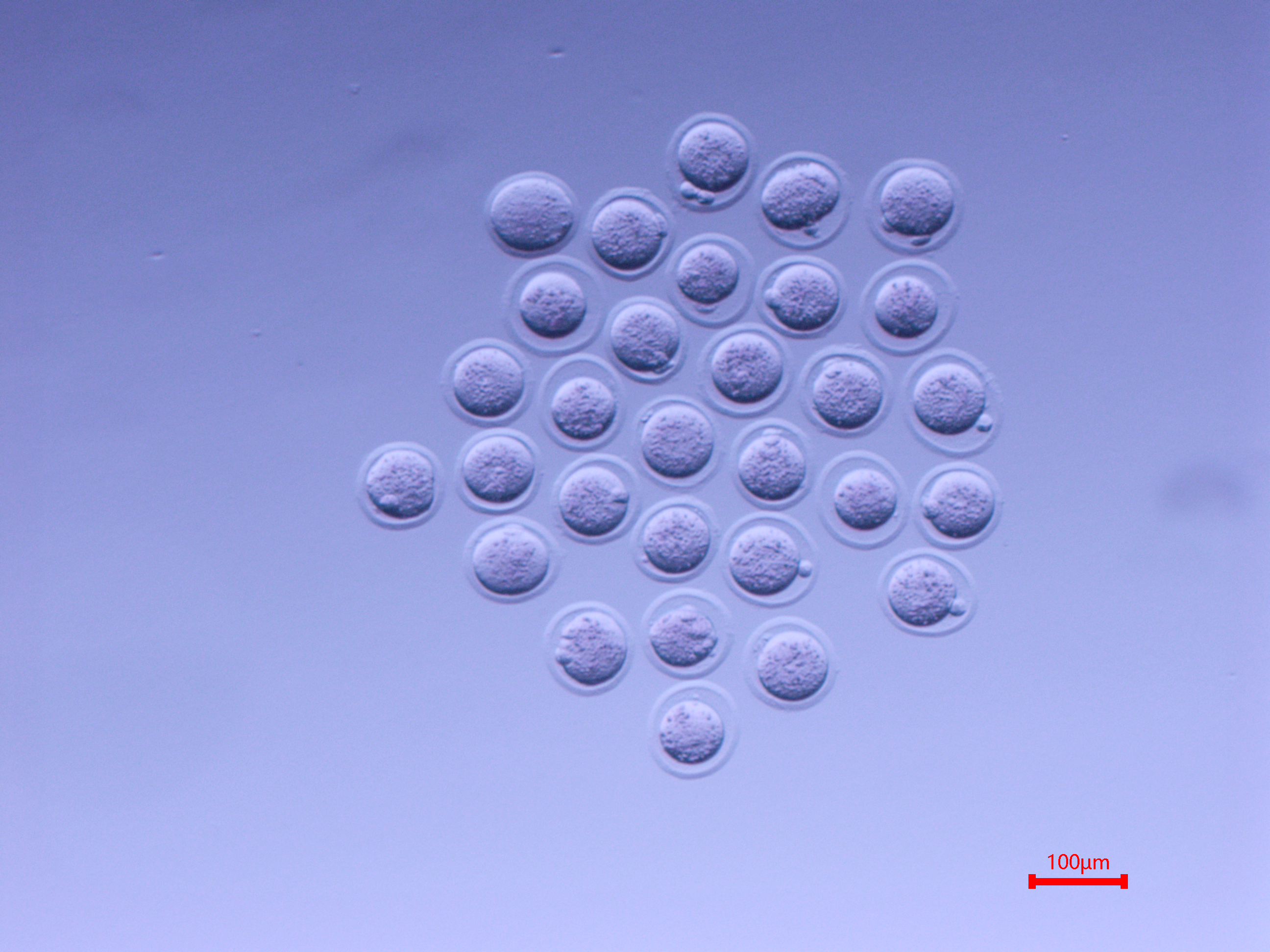

Supplement: Supplementary file 6 — Source data Fig. 5 [file 44319_2026_780_MOESM6_ESM.zip › Figure 5/Fig5C, E/0/5uM-Rescue.tif]

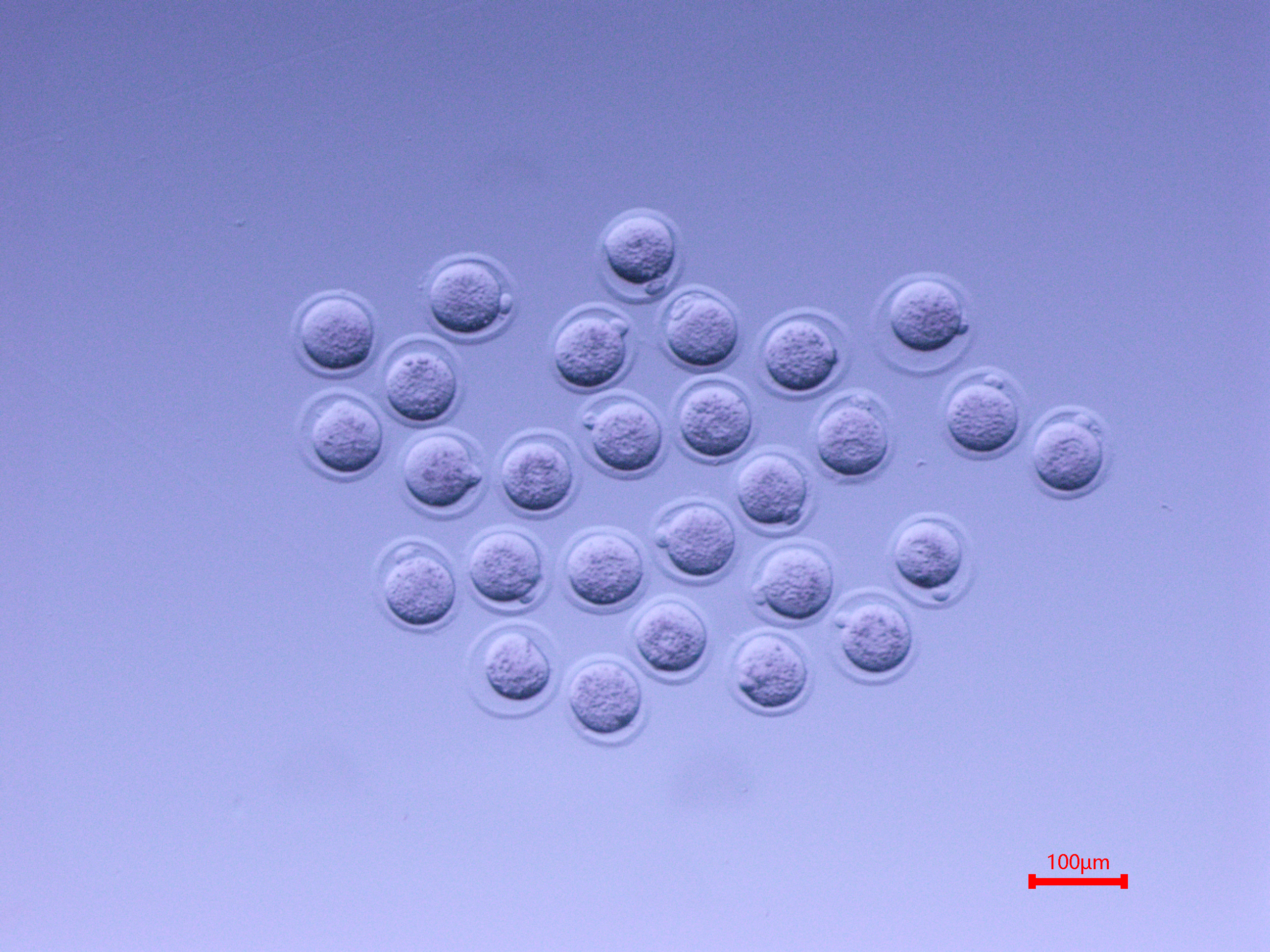

Supplement: Supplementary file 6 — Source data Fig. 5 [file 44319_2026_780_MOESM6_ESM.zip › Figure 5/Fig5C, E/0/5uM.tif]

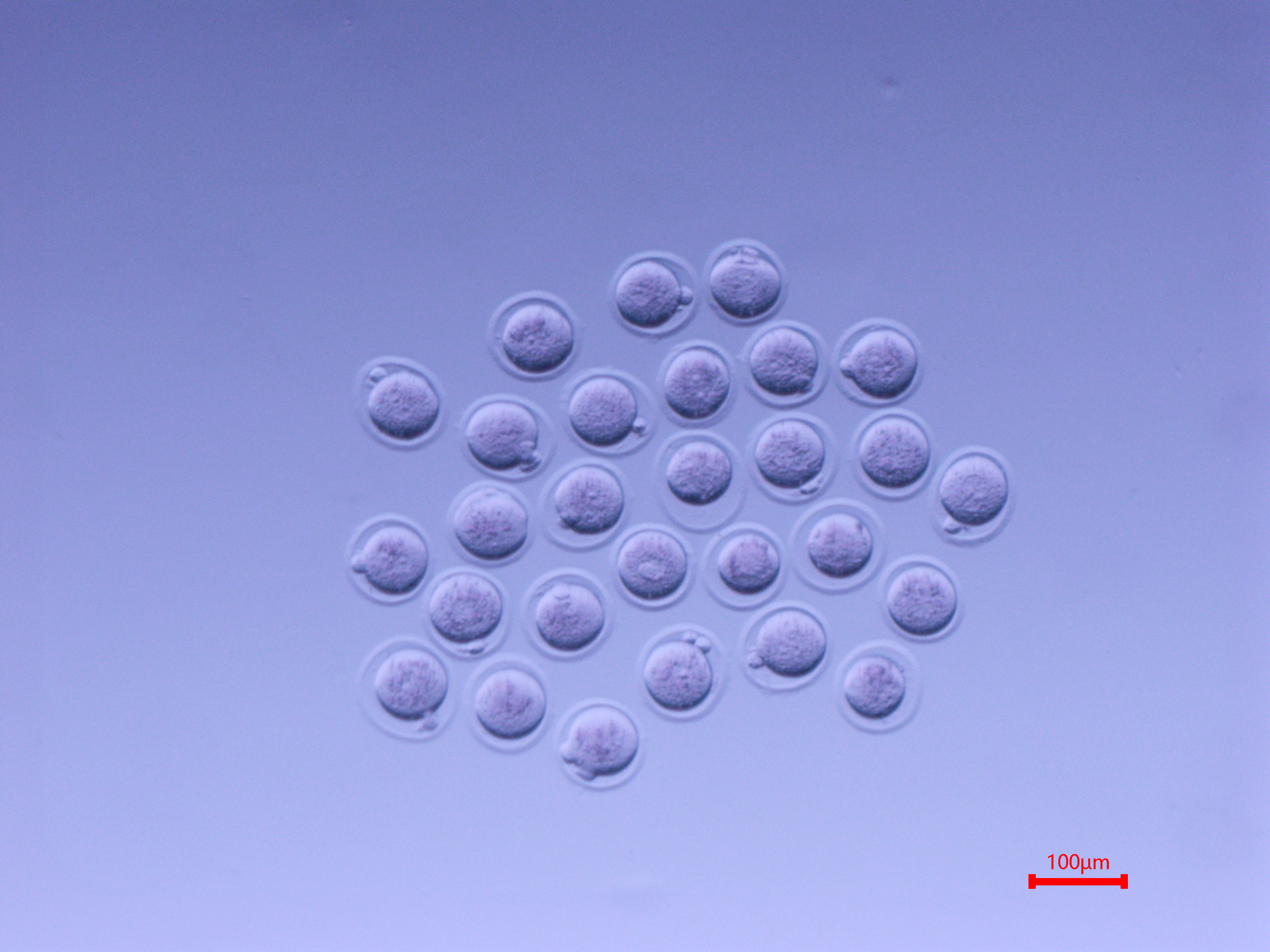

Supplement: Supplementary file 6 — Source data Fig. 5 [file 44319_2026_780_MOESM6_ESM.zip › Figure 5/Fig5C, E/0/7.5uM-Rescue.tif]

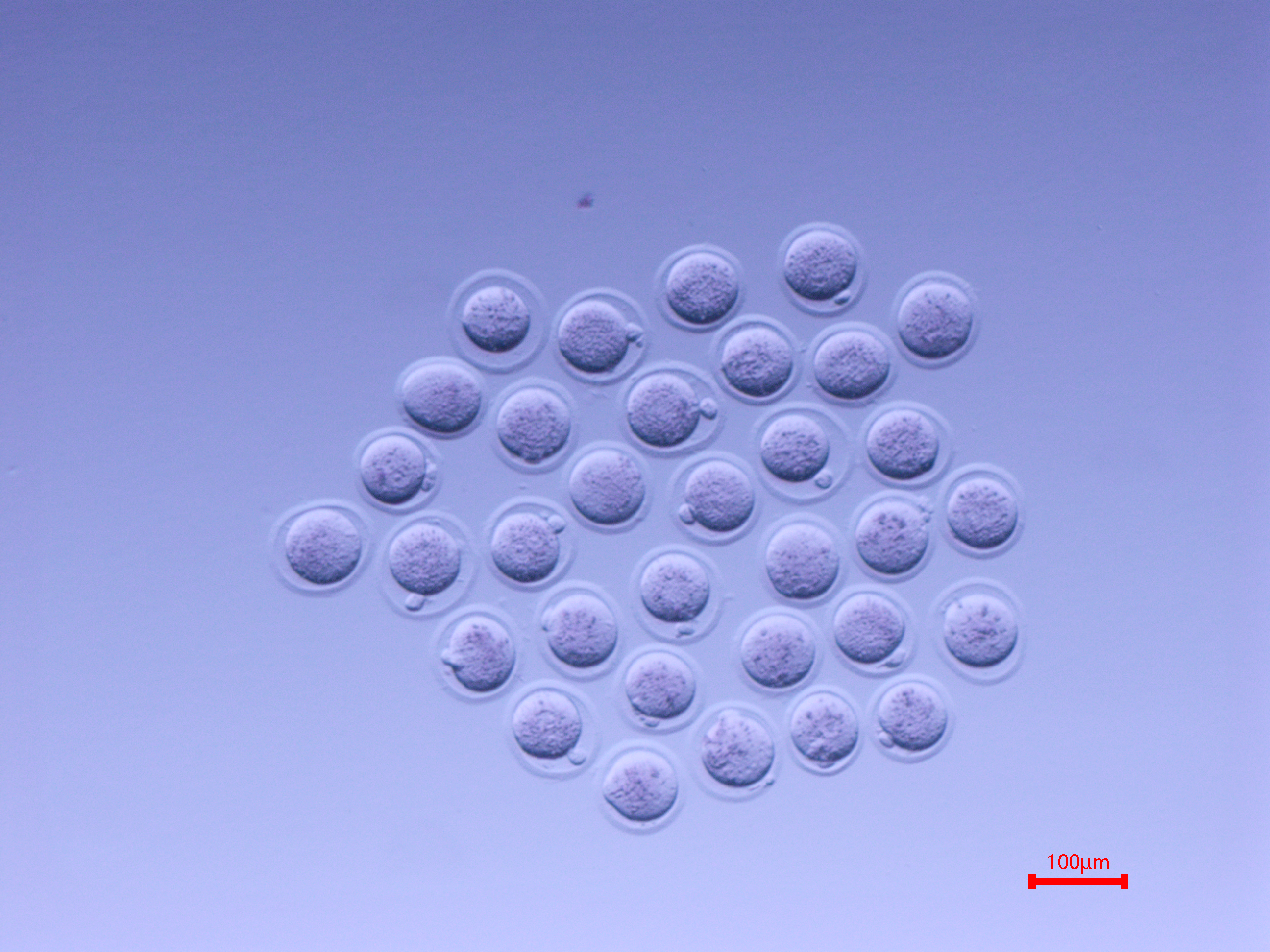

Supplement: Supplementary file 6 — Source data Fig. 5 [file 44319_2026_780_MOESM6_ESM.zip › Figure 5/Fig5C, E/0/7.5uM.tif]

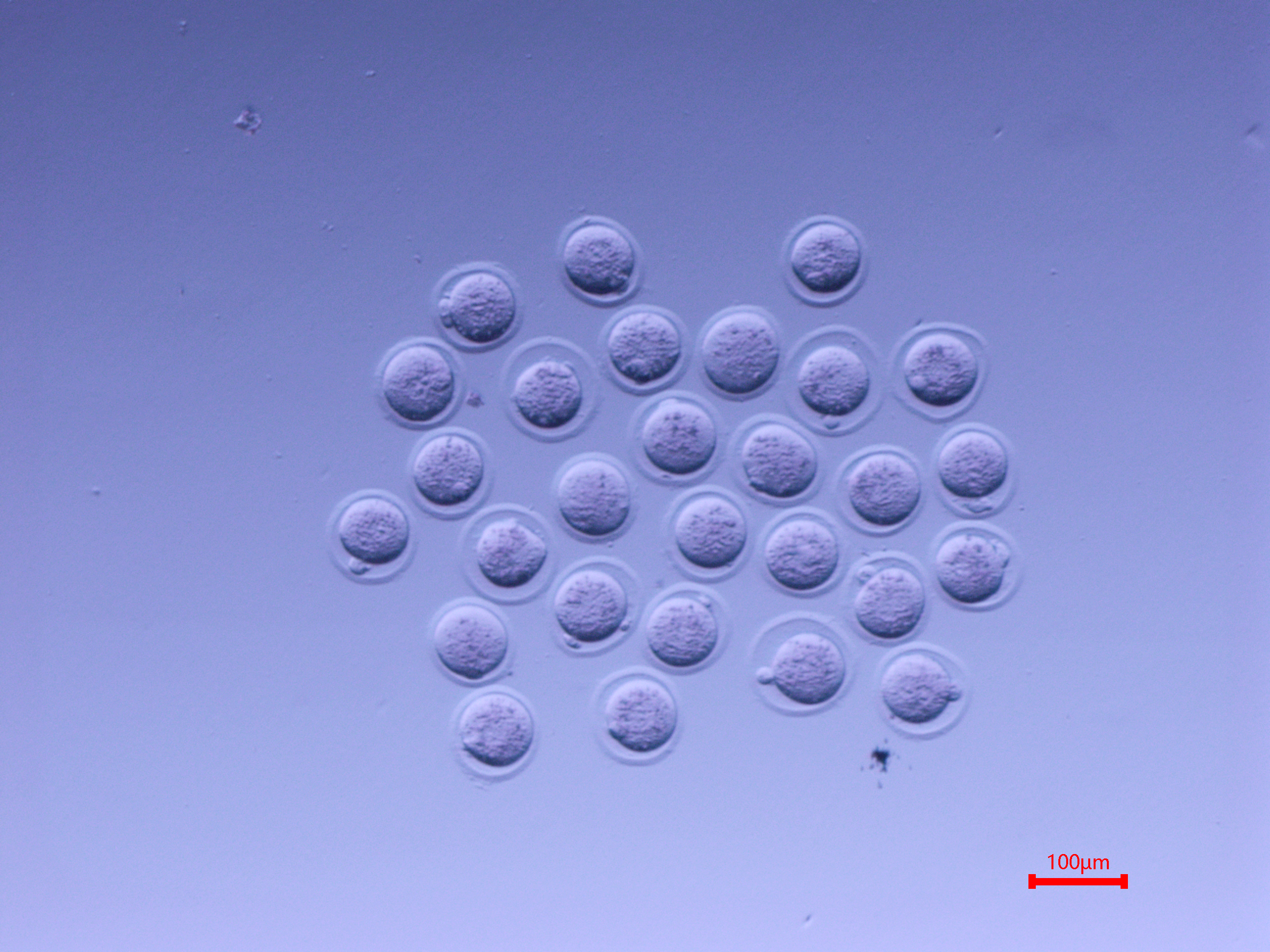

Supplement: Supplementary file 6 — Source data Fig. 5 [file 44319_2026_780_MOESM6_ESM.zip › Figure 5/Fig5C, E/0/Control.tif]

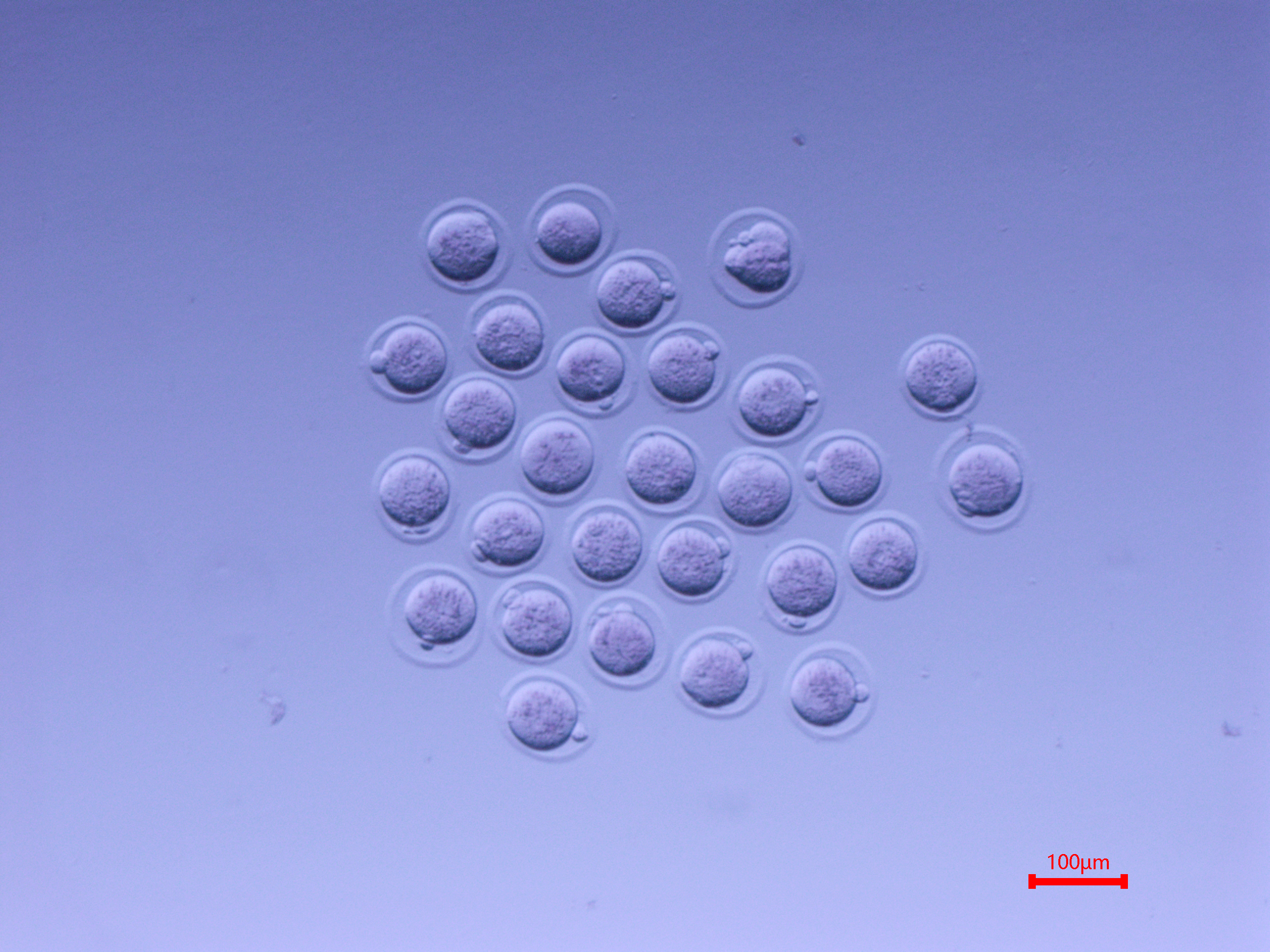

Supplement: Supplementary file 6 — Source data Fig. 5 [file 44319_2026_780_MOESM6_ESM.zip › Figure 5/Fig5C, E/0/DMSO.tif]

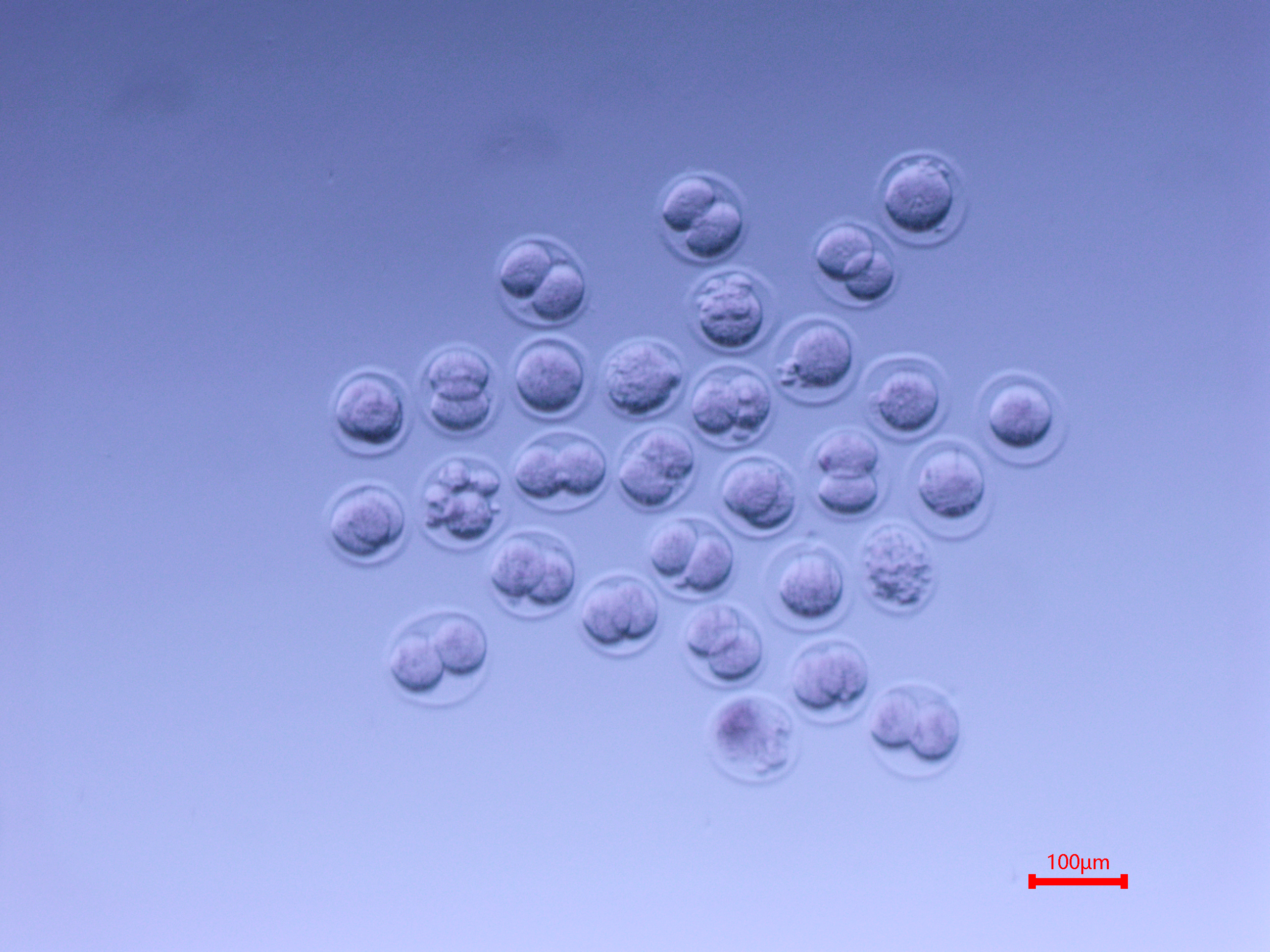

Supplement: Supplementary file 6 — Source data Fig. 5 [file 44319_2026_780_MOESM6_ESM.zip › Figure 5/Fig5C, E/16h/5uM-Rescue.tif]

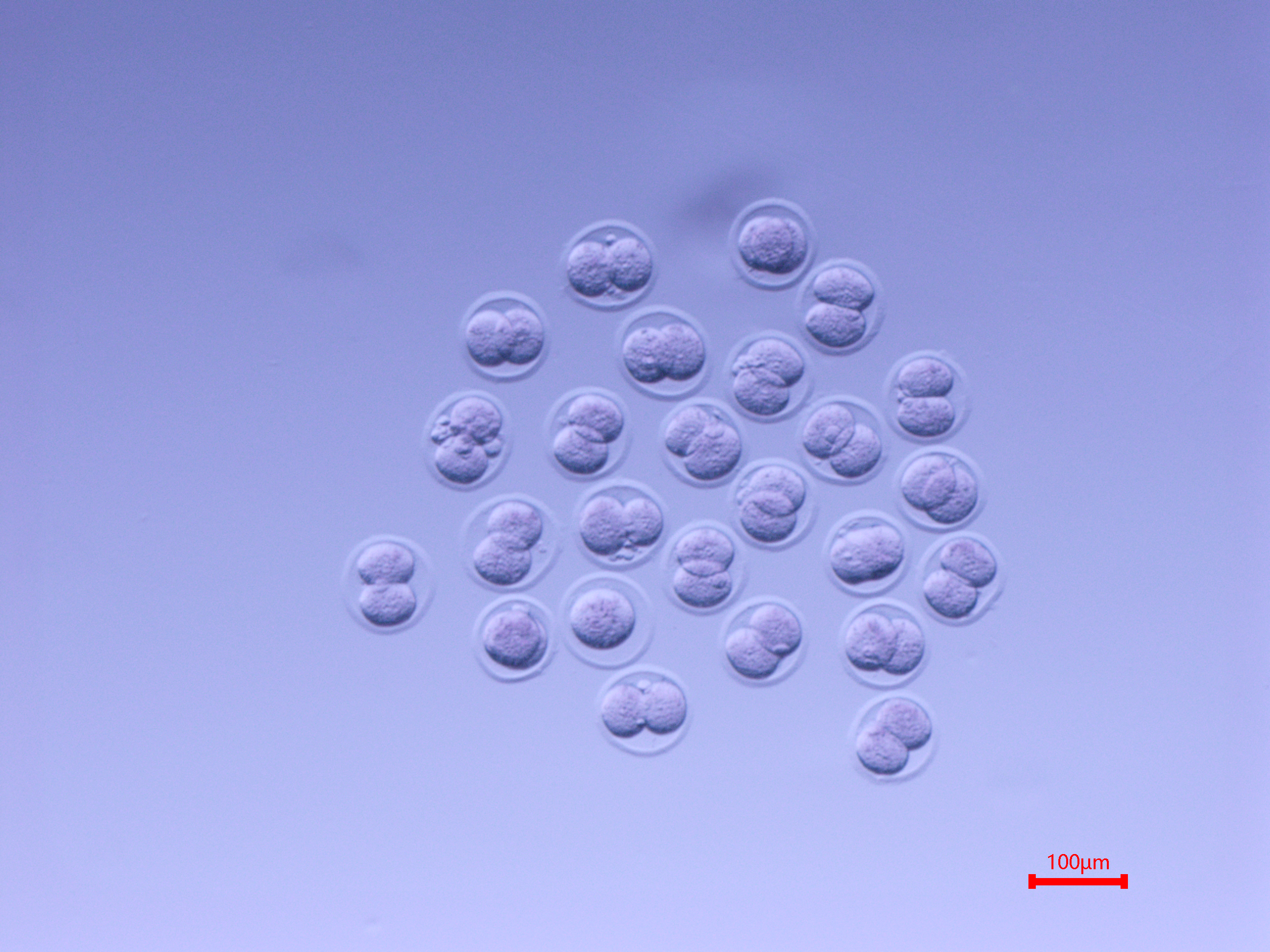

Supplement: Supplementary file 6 — Source data Fig. 5 [file 44319_2026_780_MOESM6_ESM.zip › Figure 5/Fig5C, E/16h/5uM.tif]

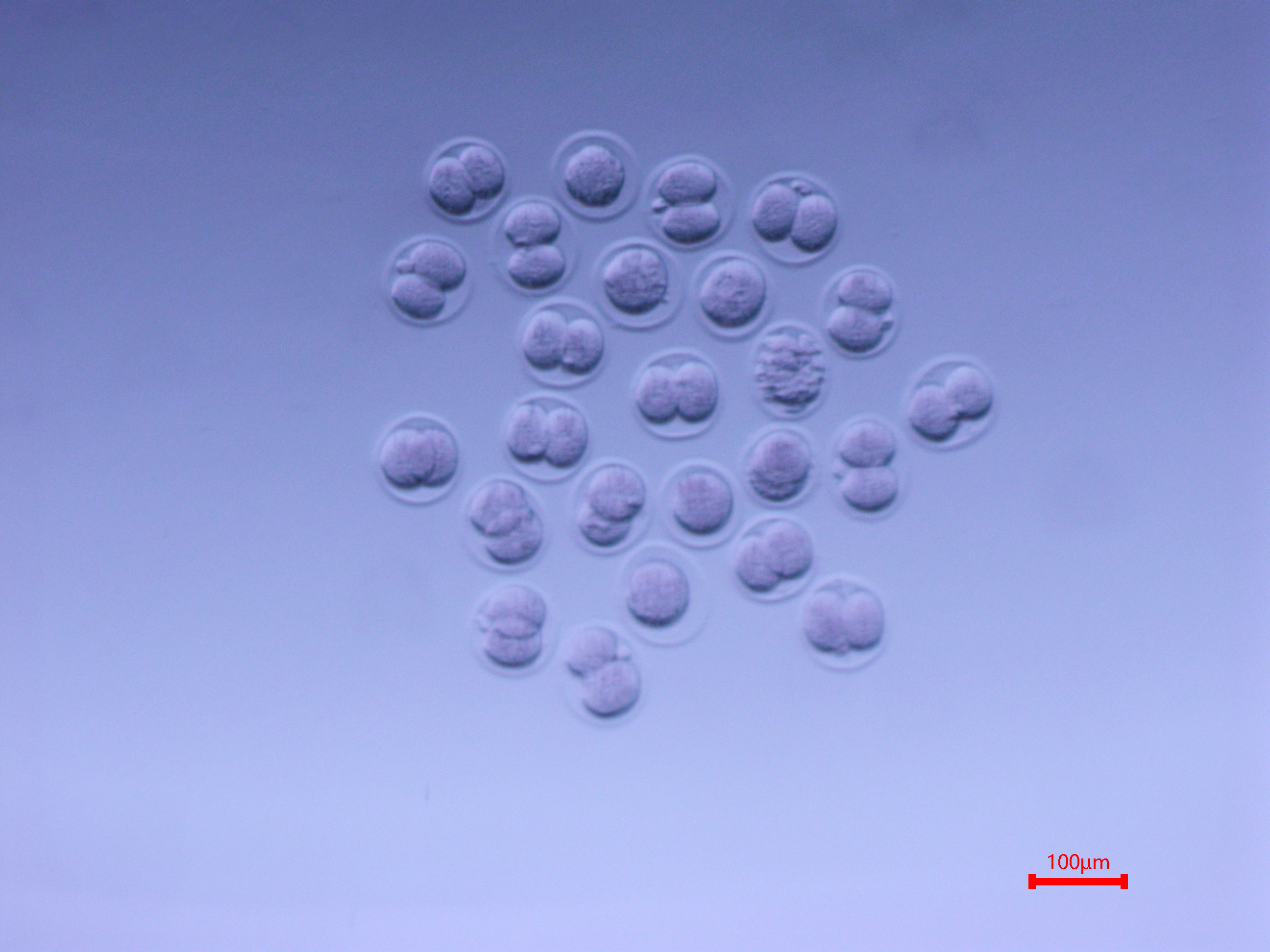

Supplement: Supplementary file 6 — Source data Fig. 5 [file 44319_2026_780_MOESM6_ESM.zip › Figure 5/Fig5C, E/16h/7.5uM-Rescue.tif]

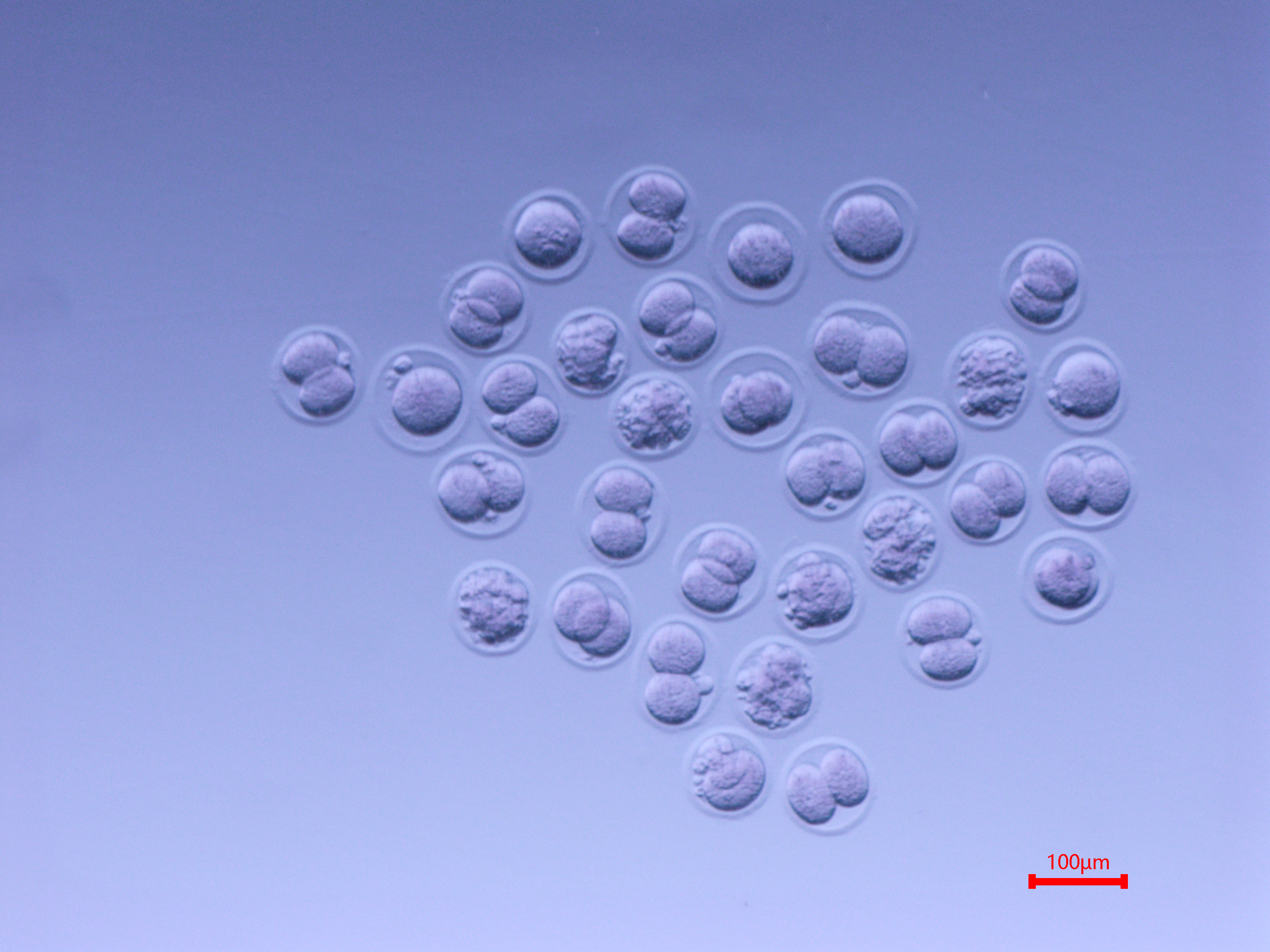

Supplement: Supplementary file 6 — Source data Fig. 5 [file 44319_2026_780_MOESM6_ESM.zip › Figure 5/Fig5C, E/16h/7.5uM.tif]

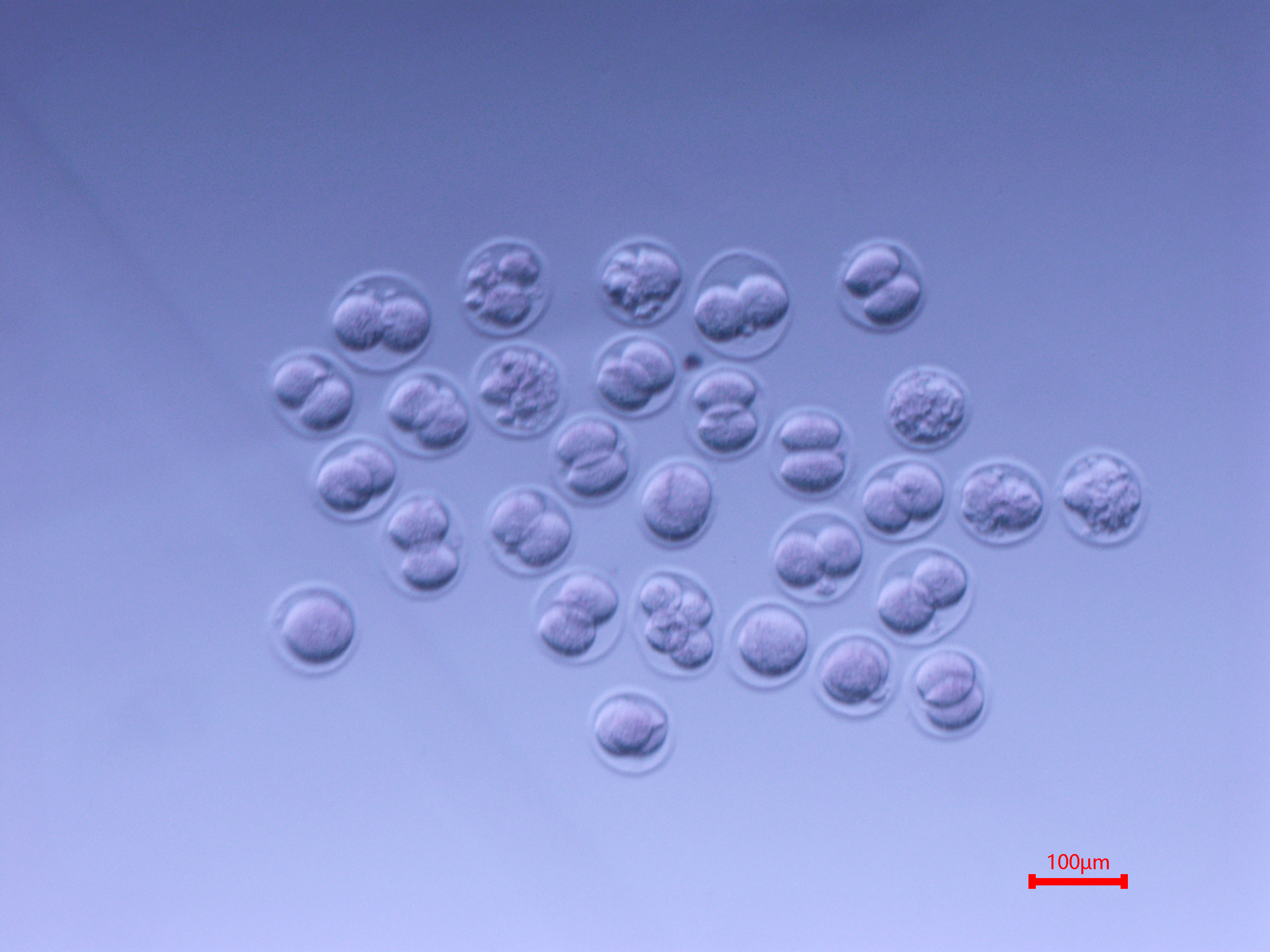

Supplement: Supplementary file 6 — Source data Fig. 5 [file 44319_2026_780_MOESM6_ESM.zip › Figure 5/Fig5C, E/16h/Control.tif]

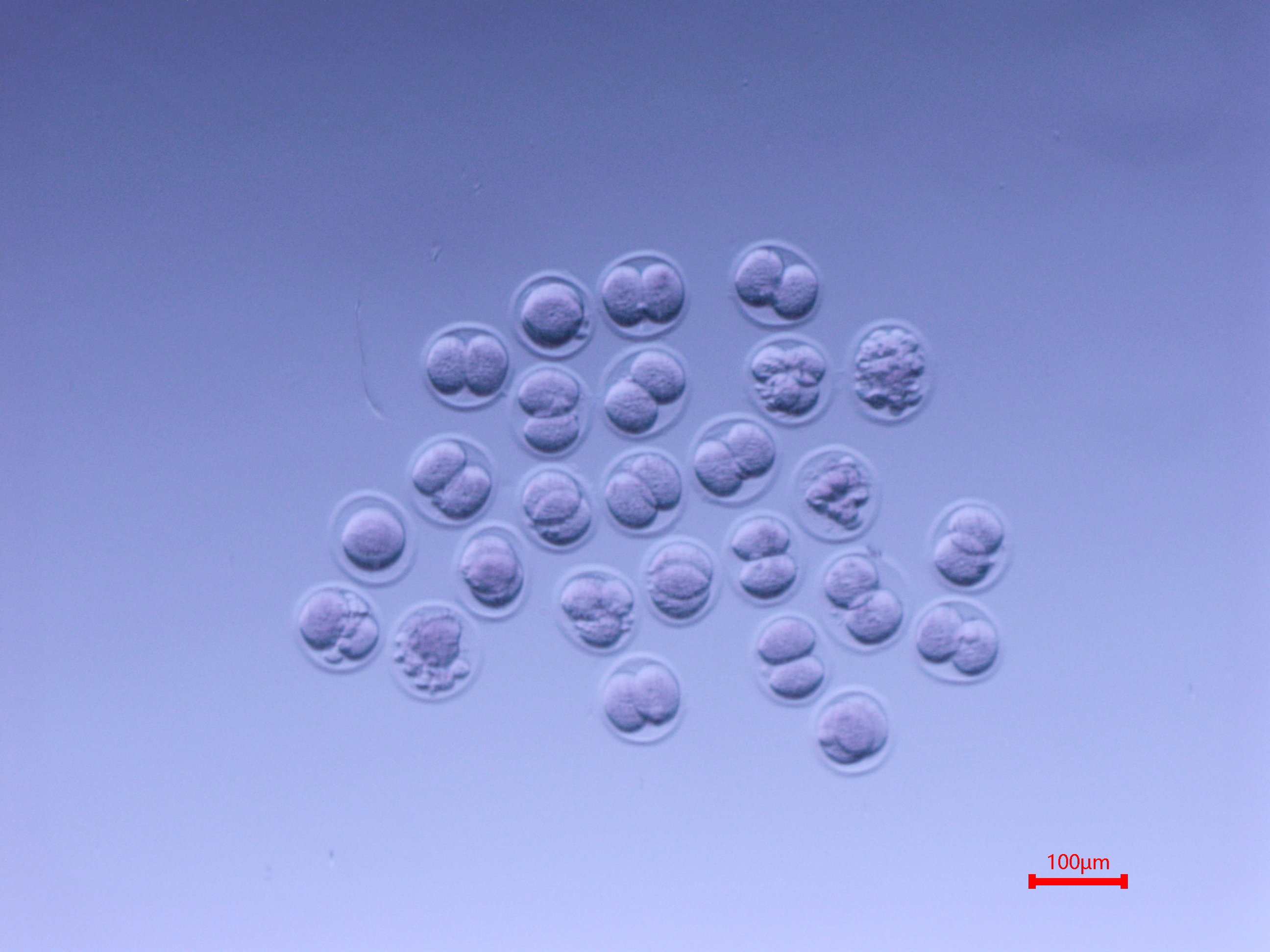

Supplement: Supplementary file 6 — Source data Fig. 5 [file 44319_2026_780_MOESM6_ESM.zip › Figure 5/Fig5C, E/16h/DMSO.tif]

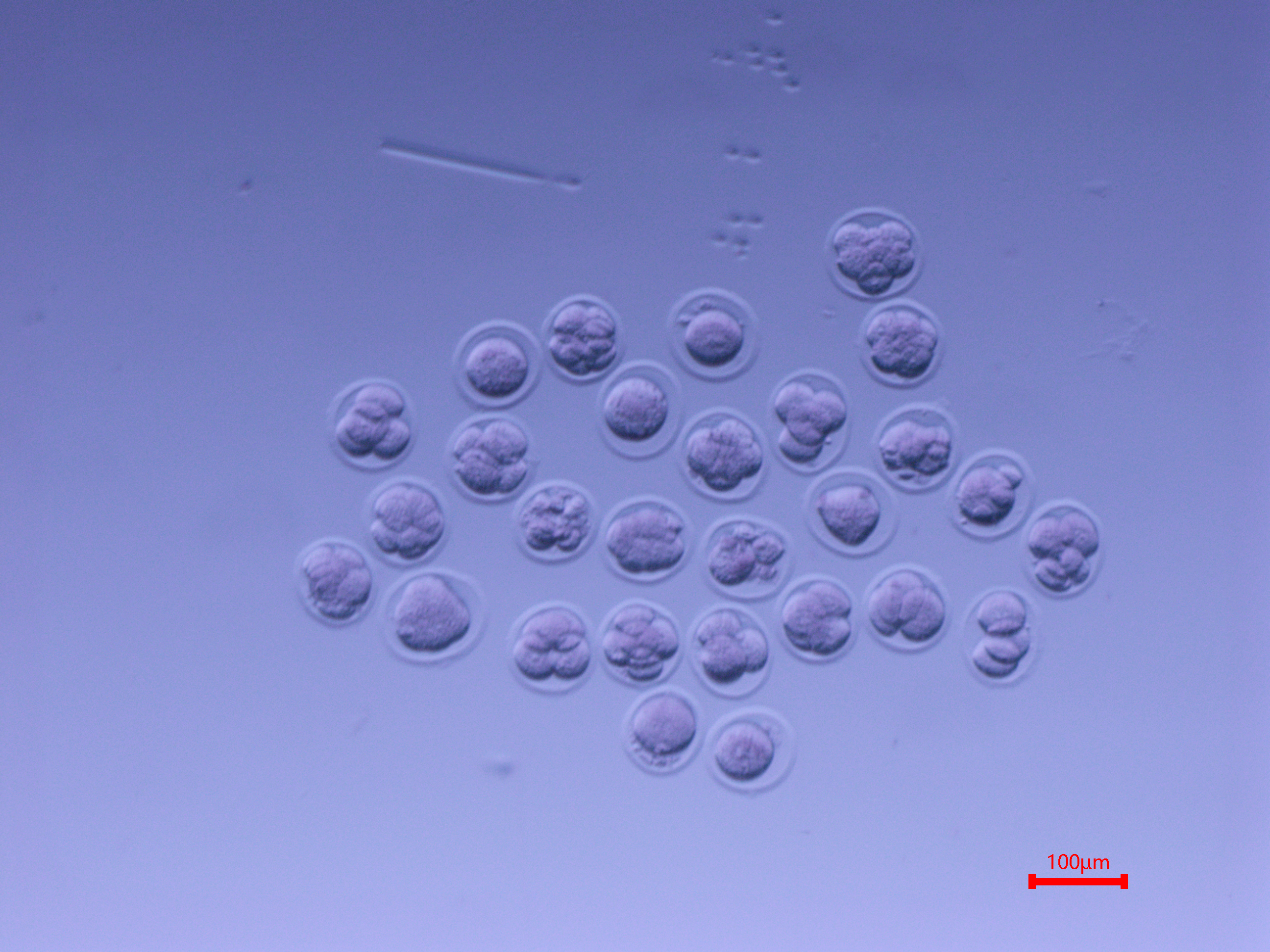

Supplement: Supplementary file 6 — Source data Fig. 5 [file 44319_2026_780_MOESM6_ESM.zip › Figure 5/Fig5C, E/40h/5uM-Rescue.tif]

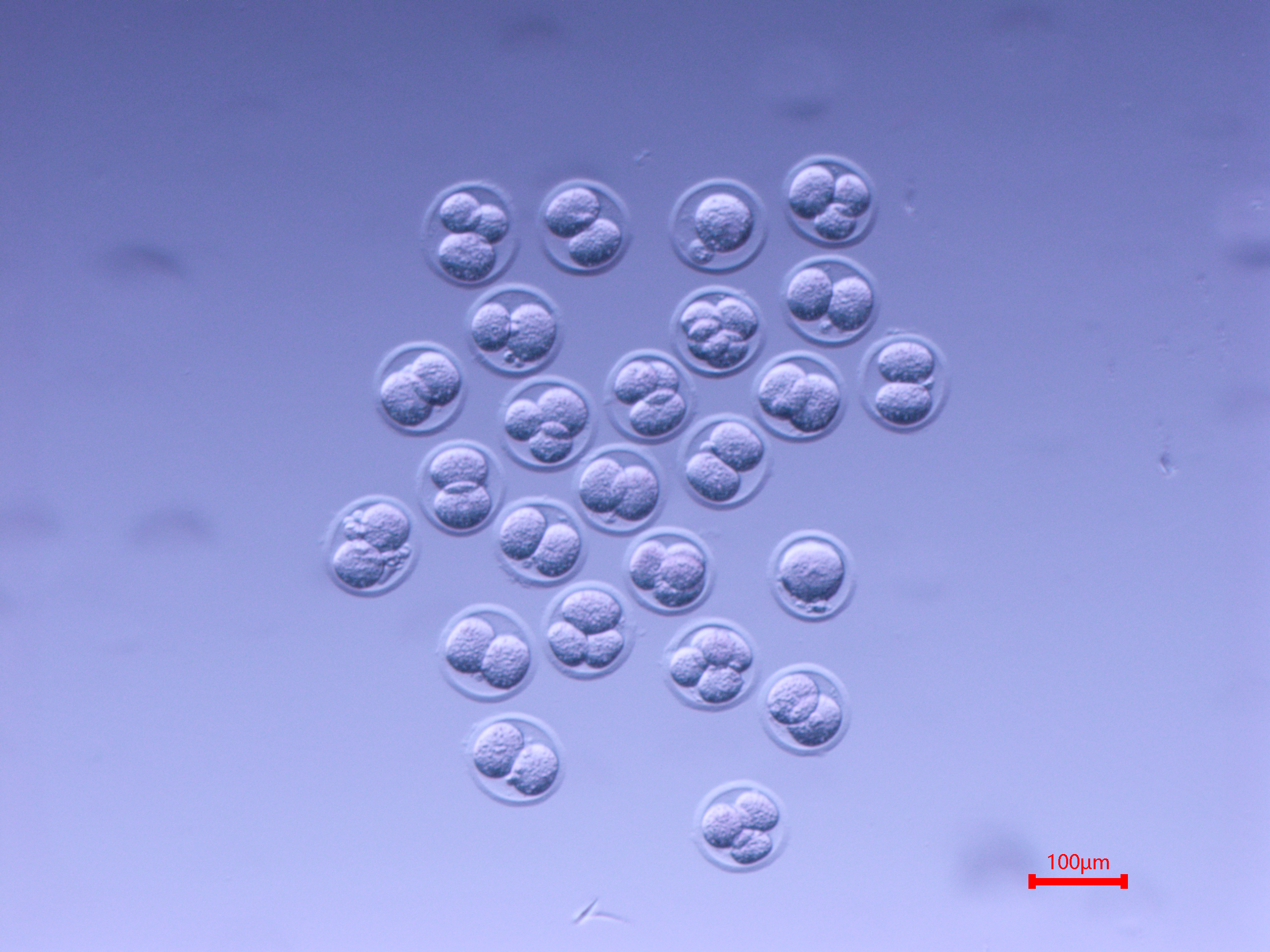

Supplement: Supplementary file 6 — Source data Fig. 5 [file 44319_2026_780_MOESM6_ESM.zip › Figure 5/Fig5C, E/40h/5uM.tif]

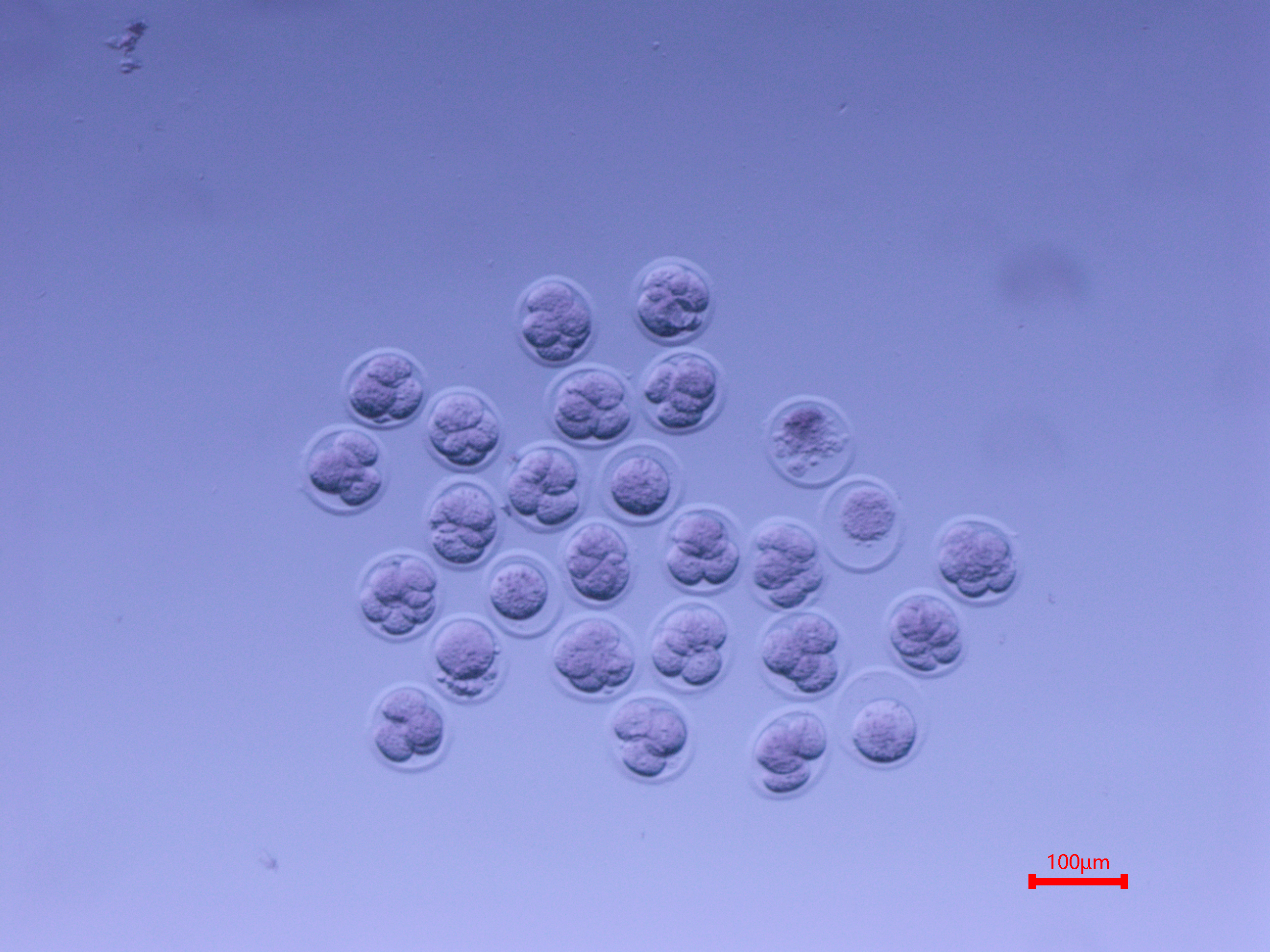

Supplement: Supplementary file 6 — Source data Fig. 5 [file 44319_2026_780_MOESM6_ESM.zip › Figure 5/Fig5C, E/40h/7.5uM-Rescue.tif]

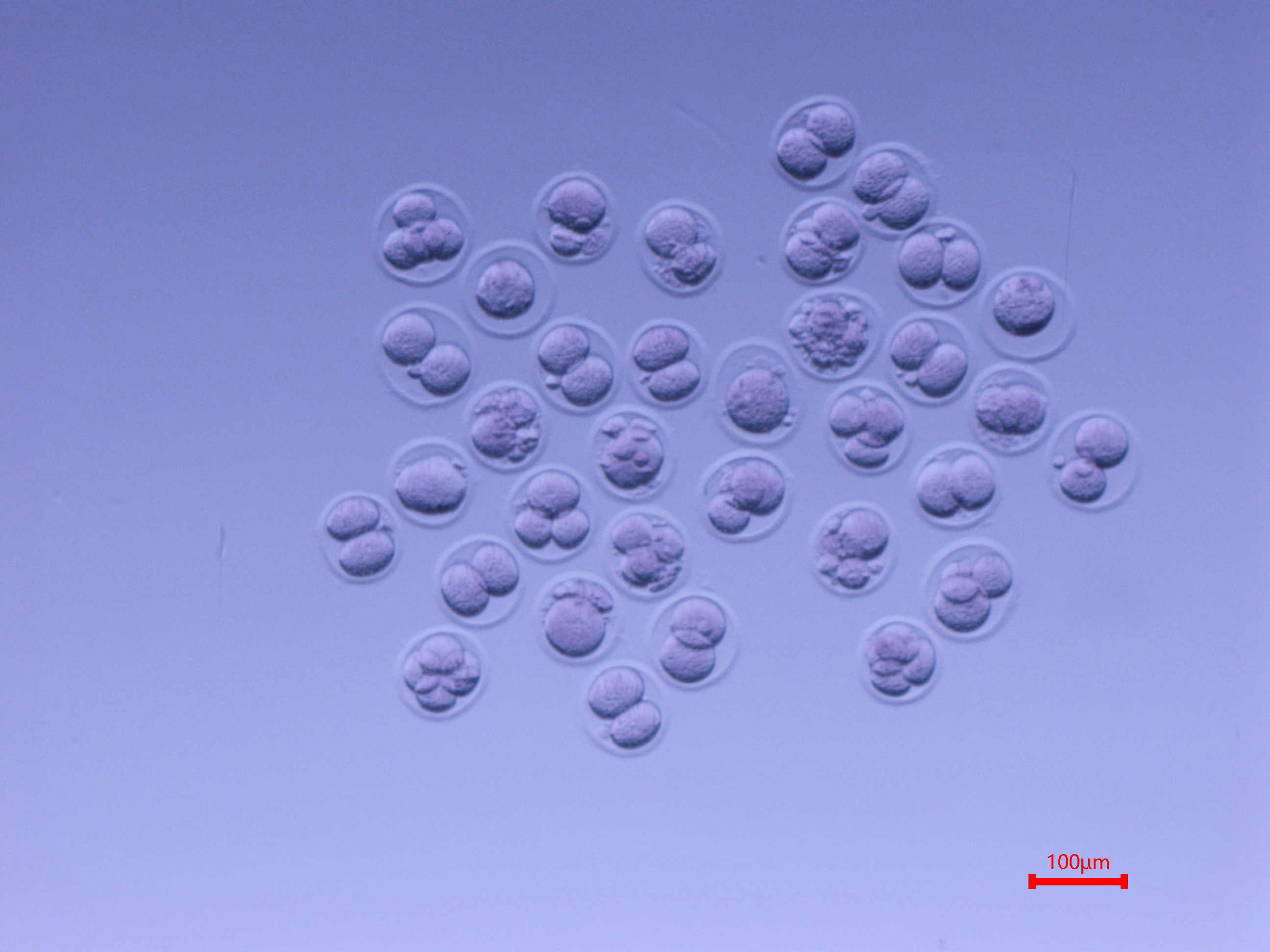

Supplement: Supplementary file 6 — Source data Fig. 5 [file 44319_2026_780_MOESM6_ESM.zip › Figure 5/Fig5C, E/40h/7.5uM.tif]

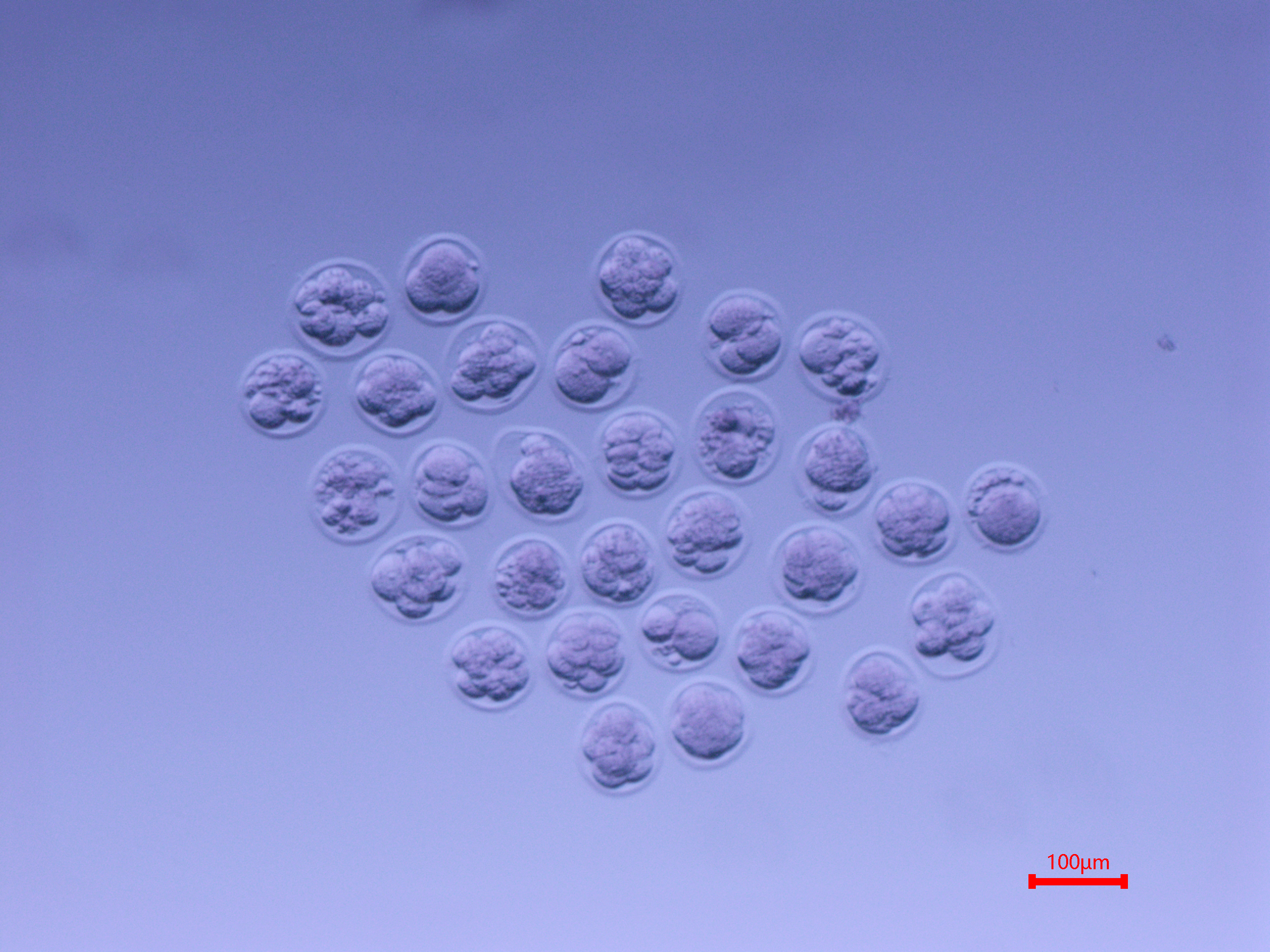

Supplement: Supplementary file 6 — Source data Fig. 5 [file 44319_2026_780_MOESM6_ESM.zip › Figure 5/Fig5C, E/40h/Control.tif]

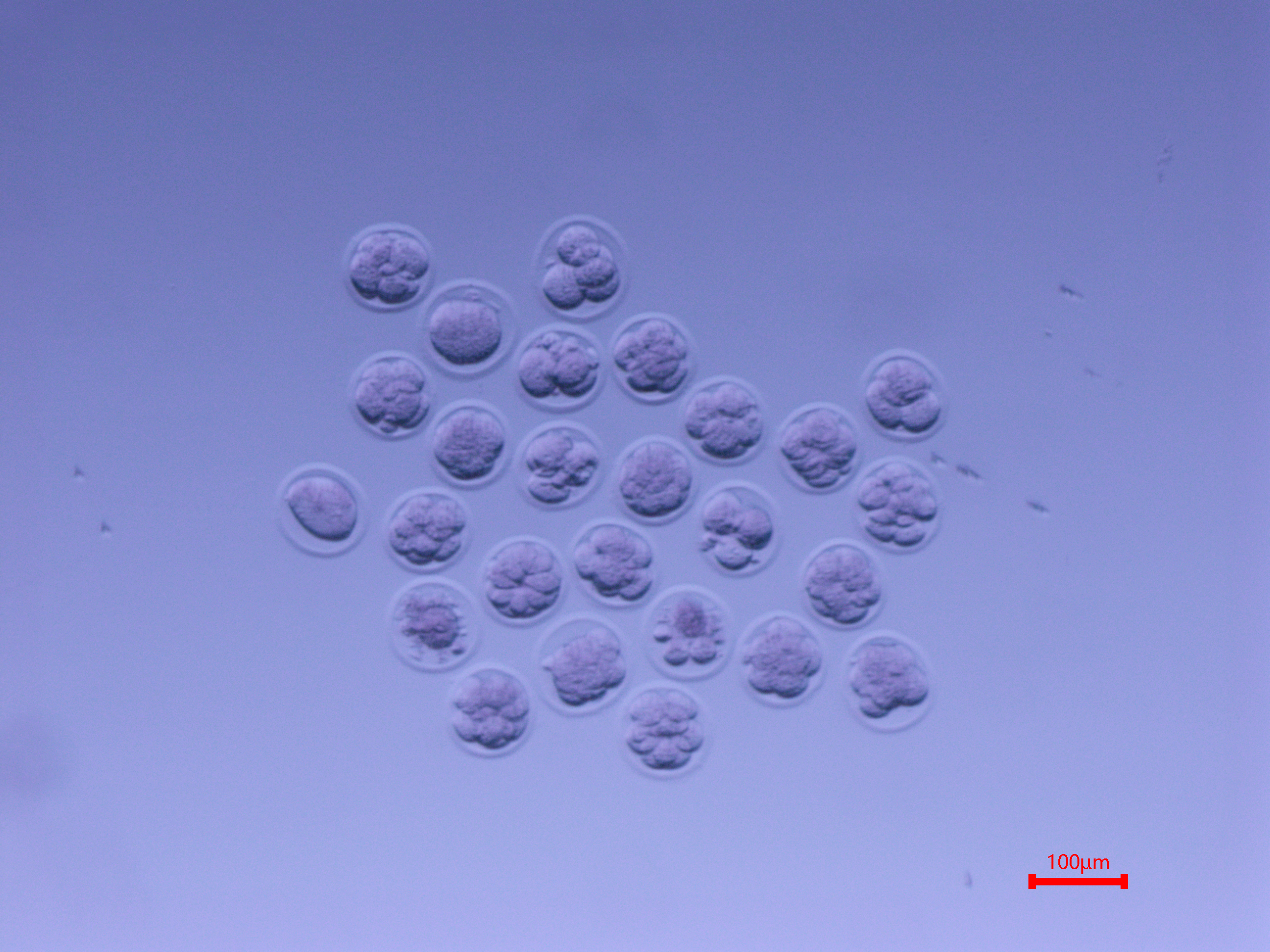

Supplement: Supplementary file 6 — Source data Fig. 5 [file 44319_2026_780_MOESM6_ESM.zip › Figure 5/Fig5C, E/40h/DMSO.tif]

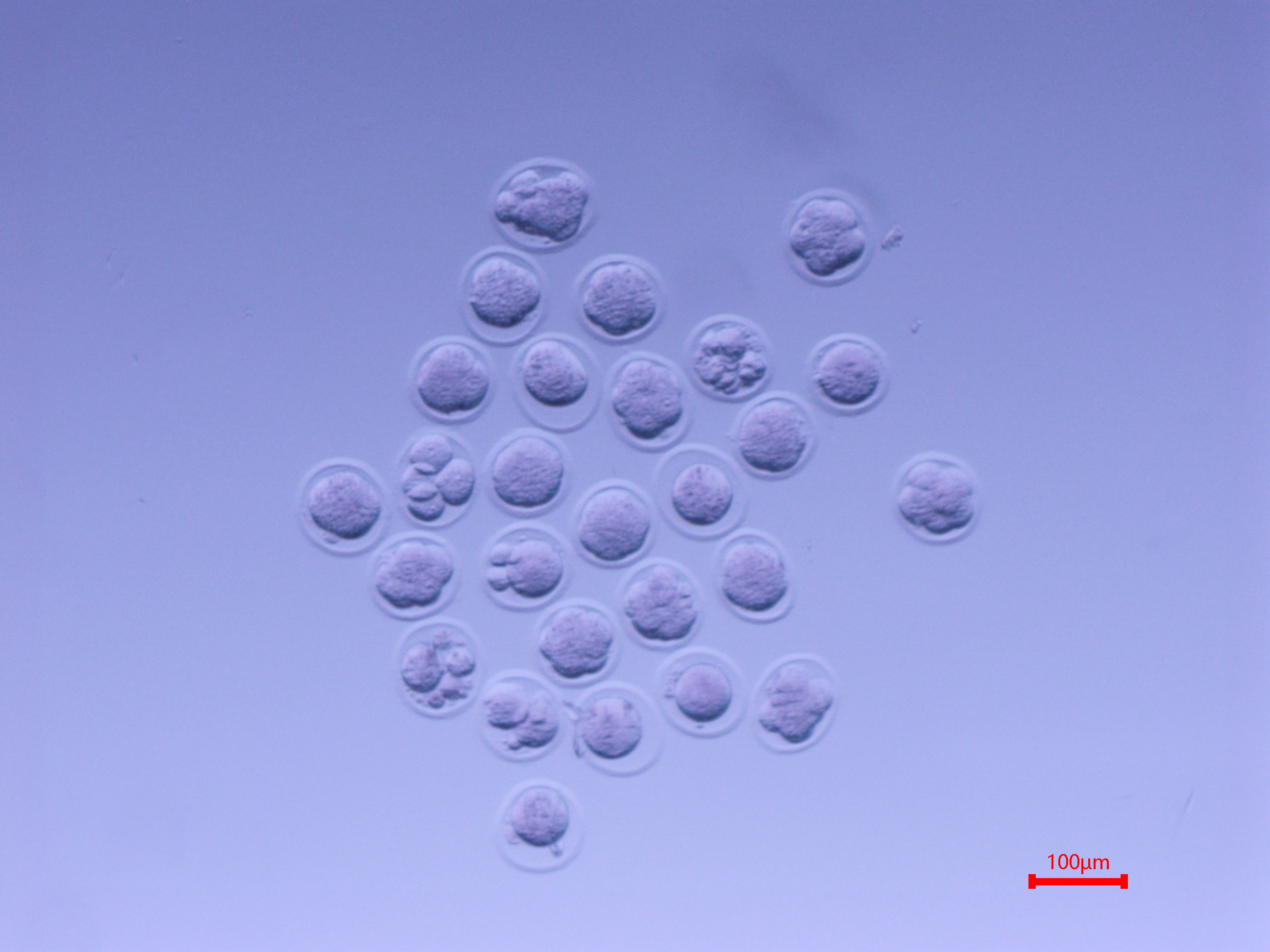

Supplement: Supplementary file 6 — Source data Fig. 5 [file 44319_2026_780_MOESM6_ESM.zip › Figure 5/Fig5C, E/48h/5uM-Rescue.tif]

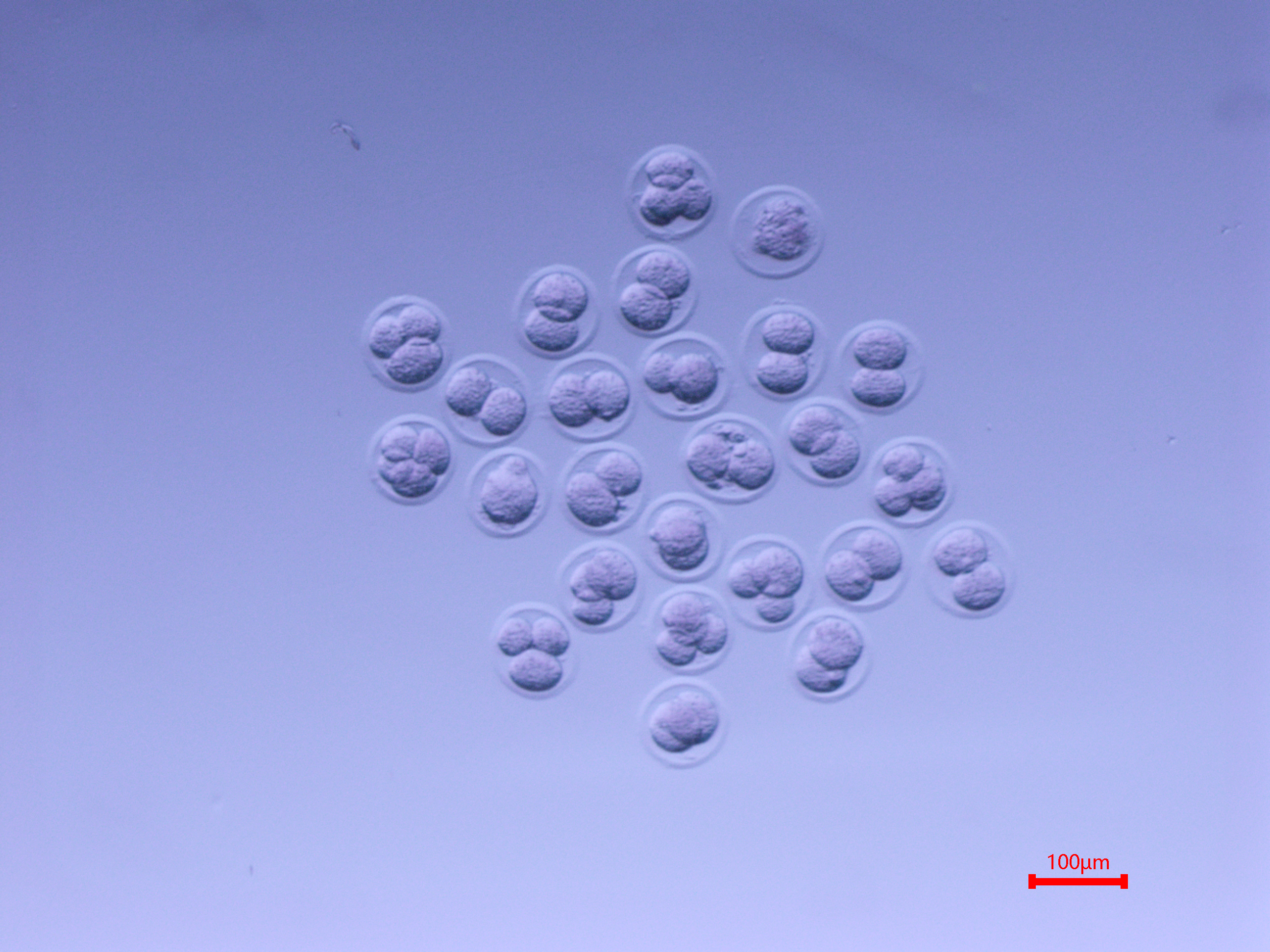

Supplement: Supplementary file 6 — Source data Fig. 5 [file 44319_2026_780_MOESM6_ESM.zip › Figure 5/Fig5C, E/48h/5uM.tif]

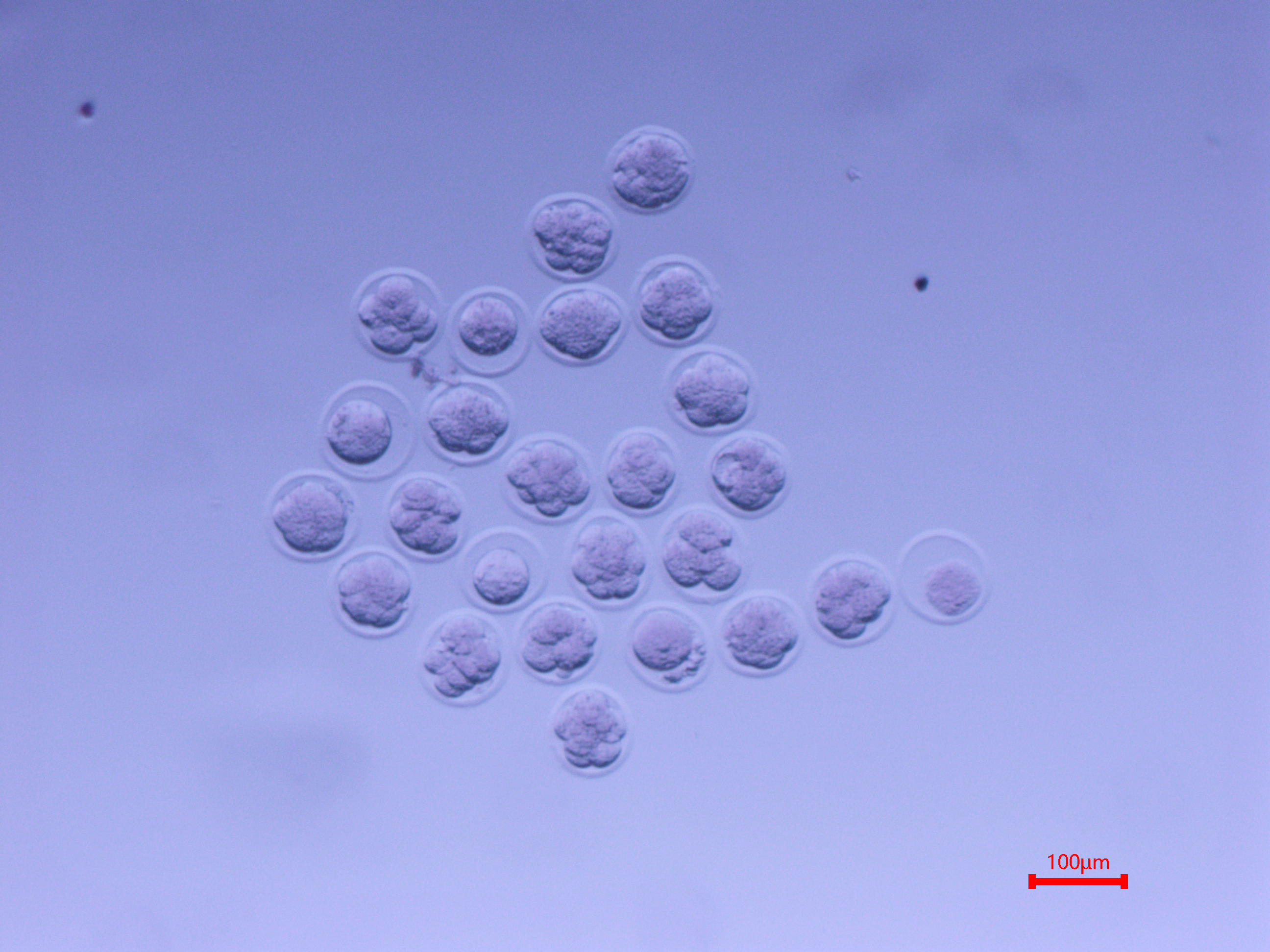

Supplement: Supplementary file 6 — Source data Fig. 5 [file 44319_2026_780_MOESM6_ESM.zip › Figure 5/Fig5C, E/48h/7.5uM-Rescue.tif]

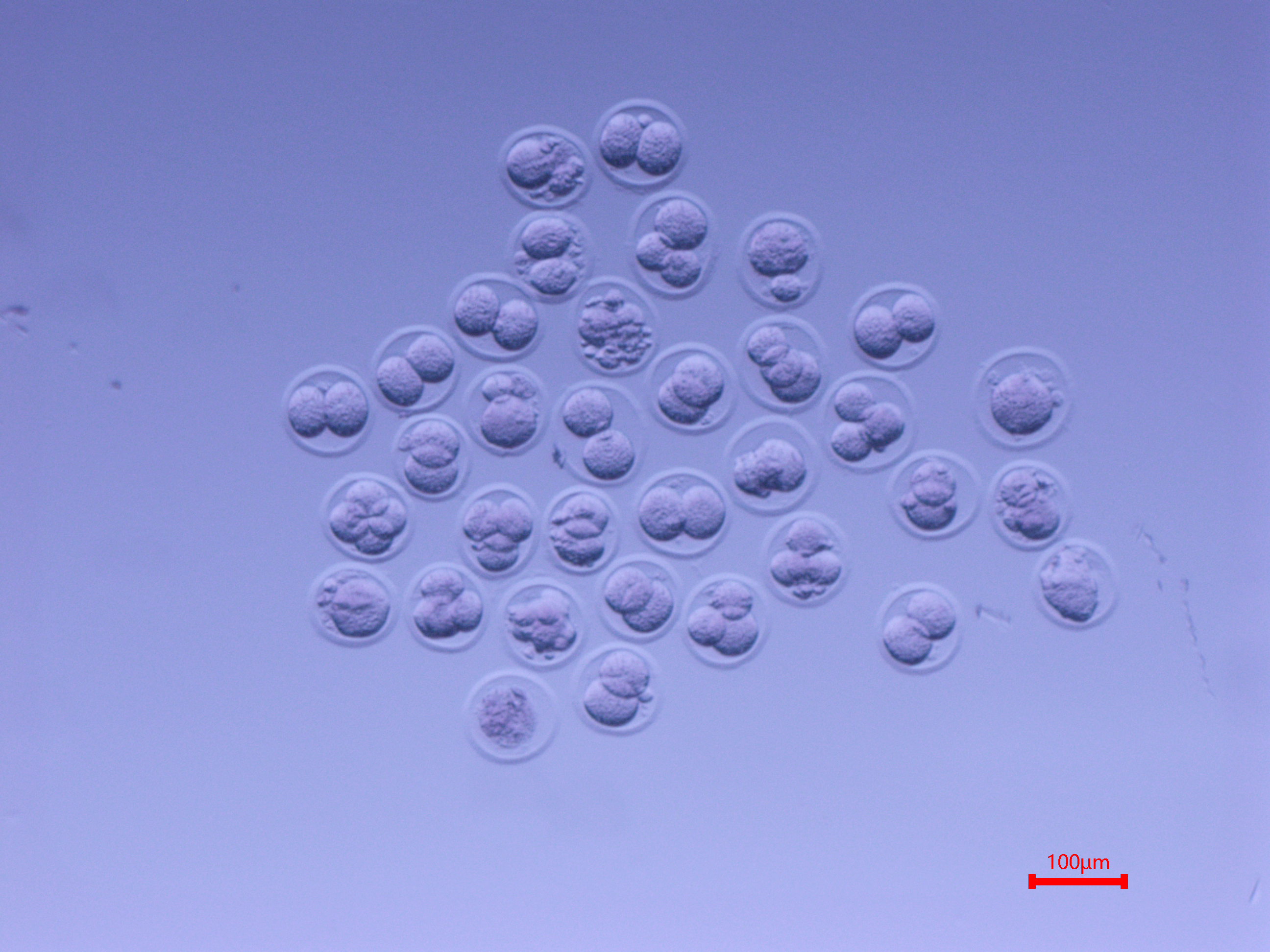

Supplement: Supplementary file 6 — Source data Fig. 5 [file 44319_2026_780_MOESM6_ESM.zip › Figure 5/Fig5C, E/48h/7.5uM.tif]

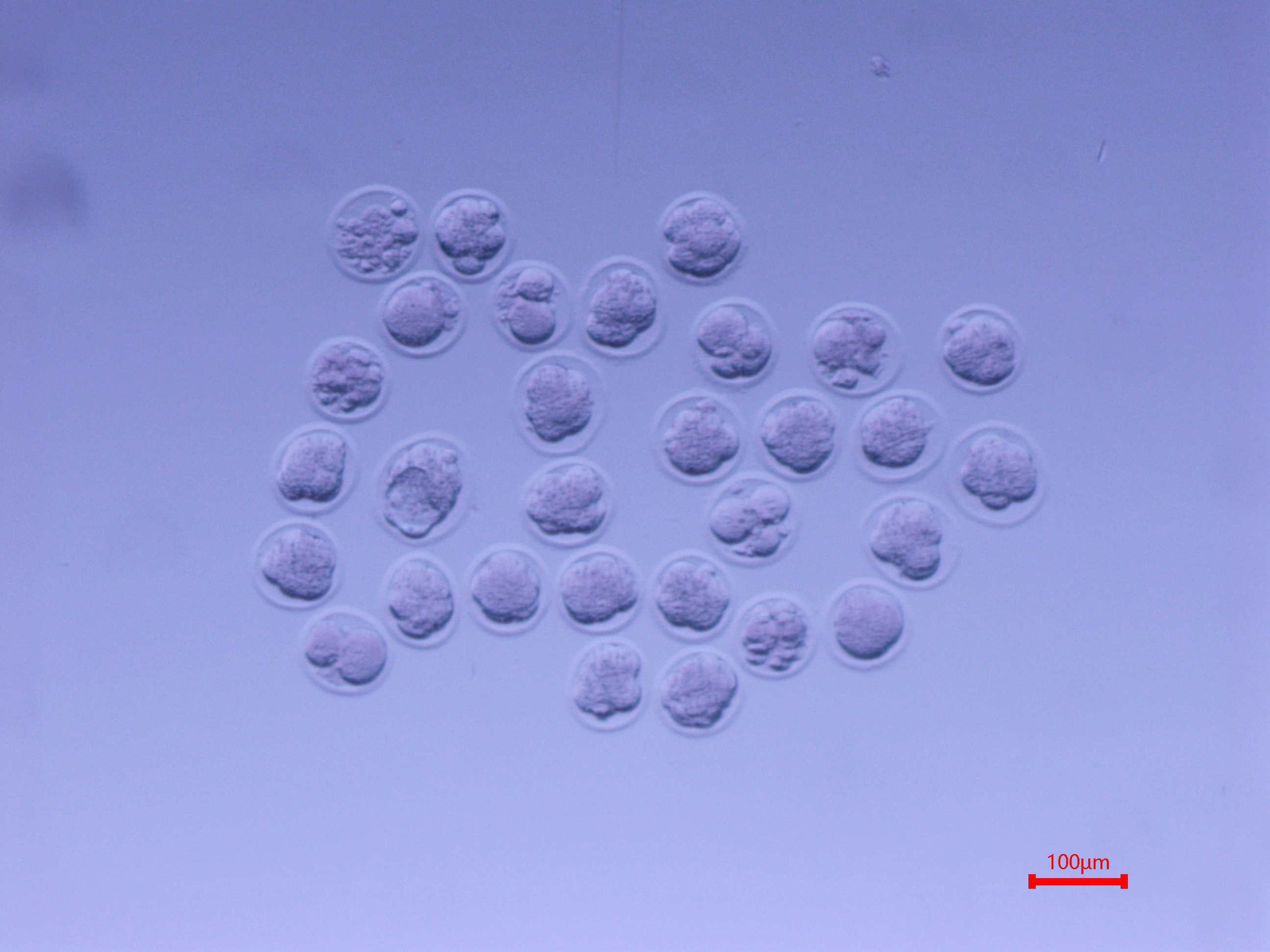

Supplement: Supplementary file 6 — Source data Fig. 5 [file 44319_2026_780_MOESM6_ESM.zip › Figure 5/Fig5C, E/48h/Control.tif]

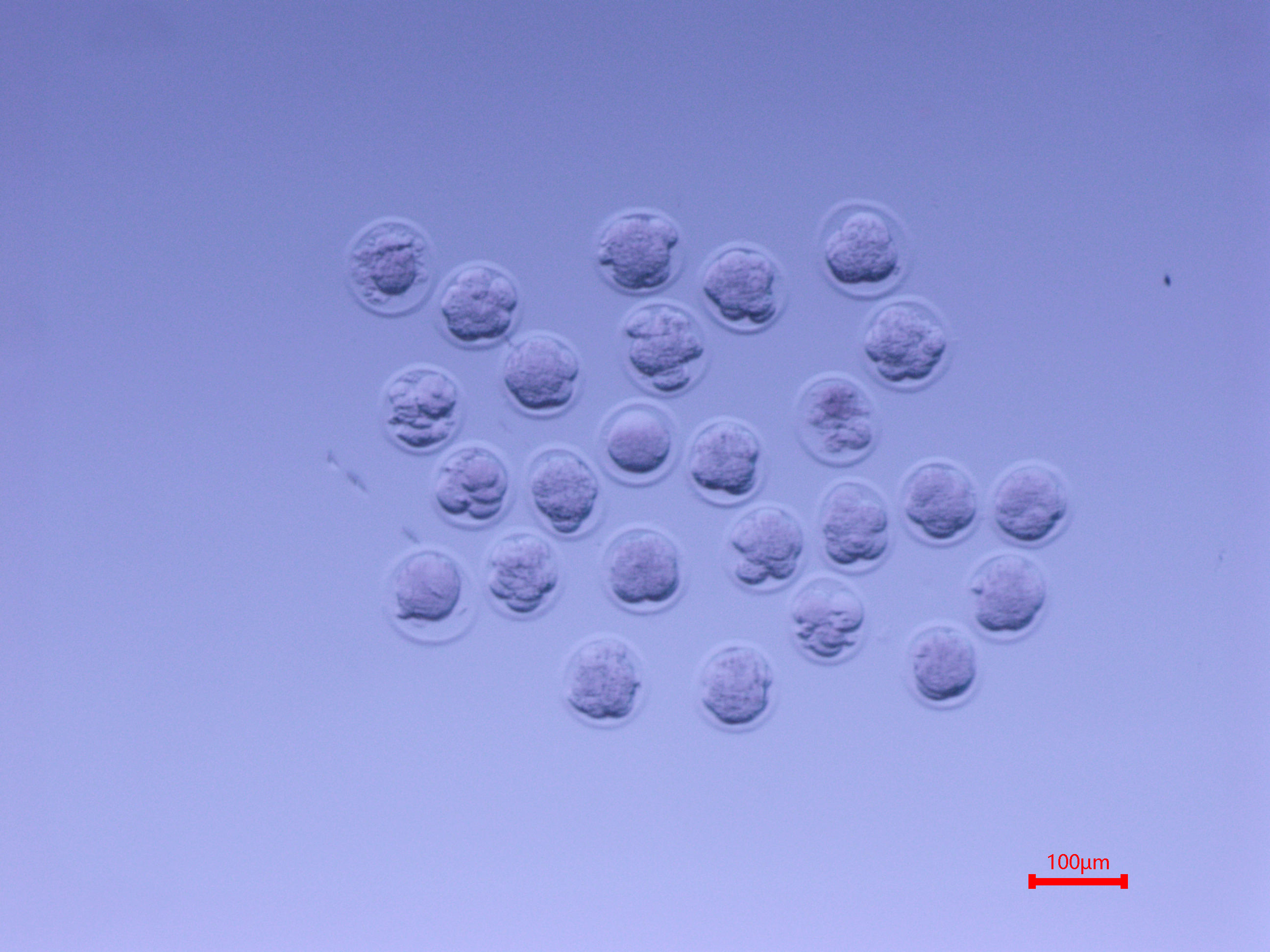

Supplement: Supplementary file 6 — Source data Fig. 5 [file 44319_2026_780_MOESM6_ESM.zip › Figure 5/Fig5C, E/48h/DMSO.tif]

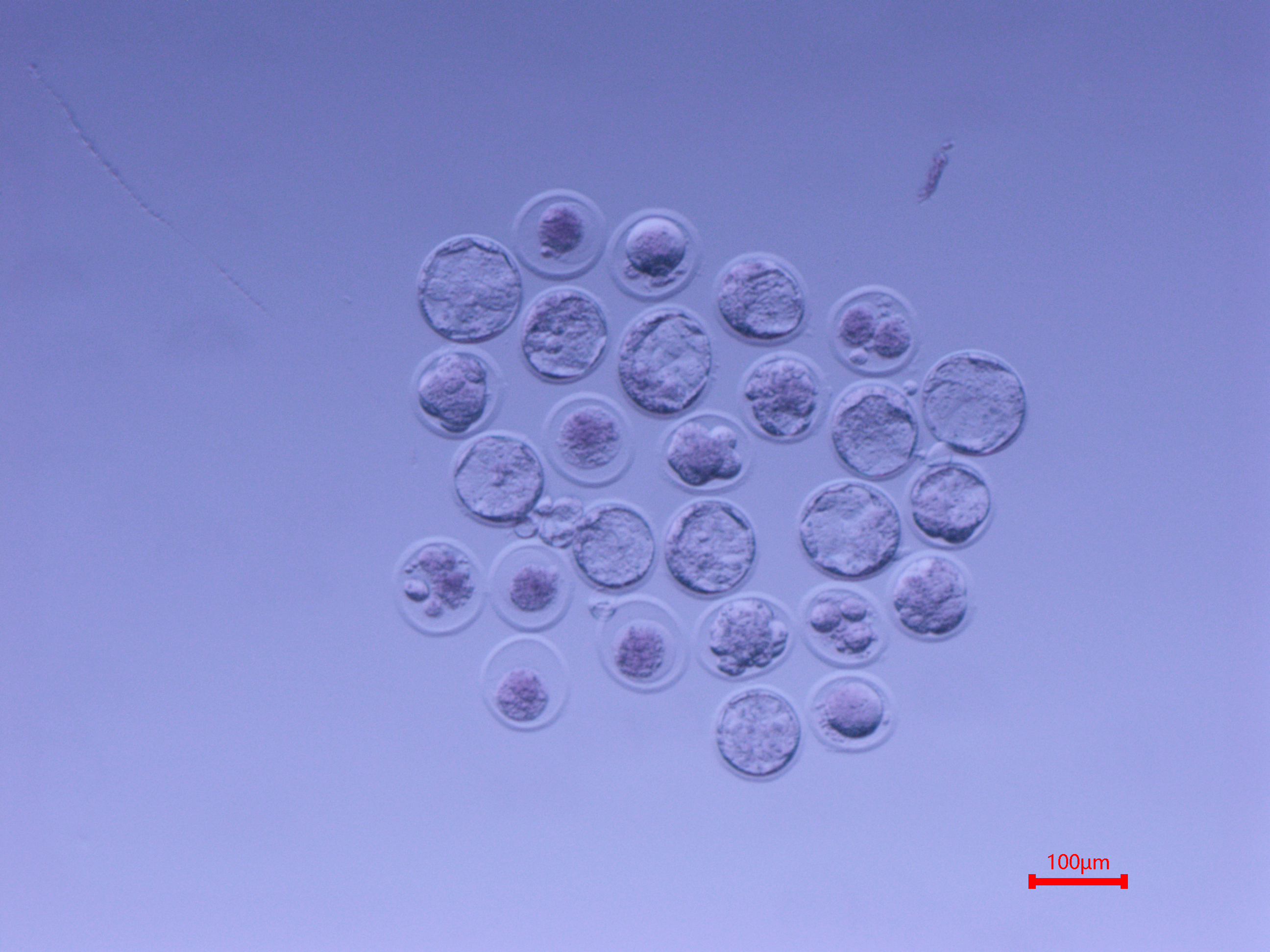

Supplement: Supplementary file 6 — Source data Fig. 5 [file 44319_2026_780_MOESM6_ESM.zip › Figure 5/Fig5C, E/72h/5uM-Rescue.tif]

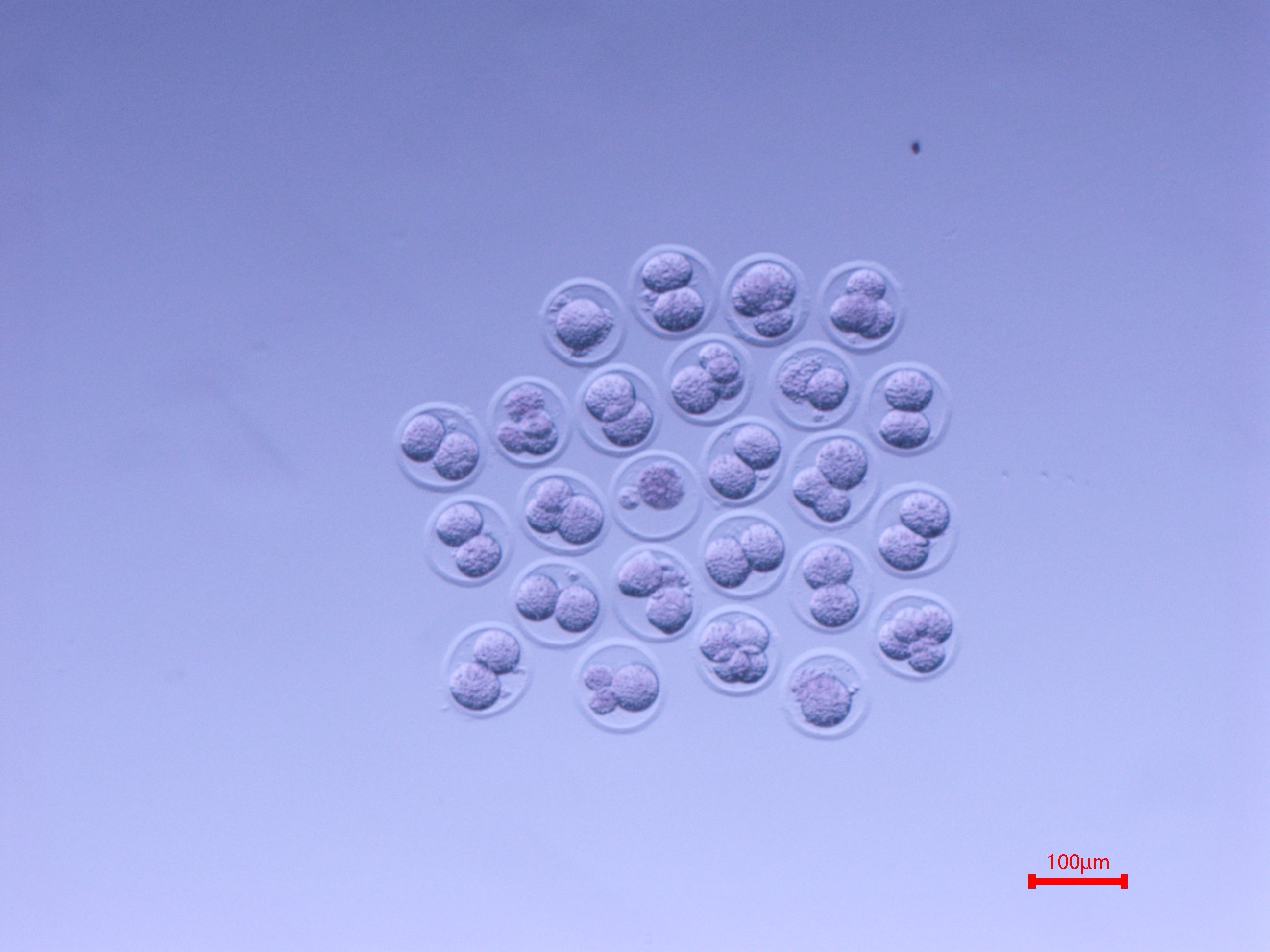

Supplement: Supplementary file 6 — Source data Fig. 5 [file 44319_2026_780_MOESM6_ESM.zip › Figure 5/Fig5C, E/72h/5uM.tif]

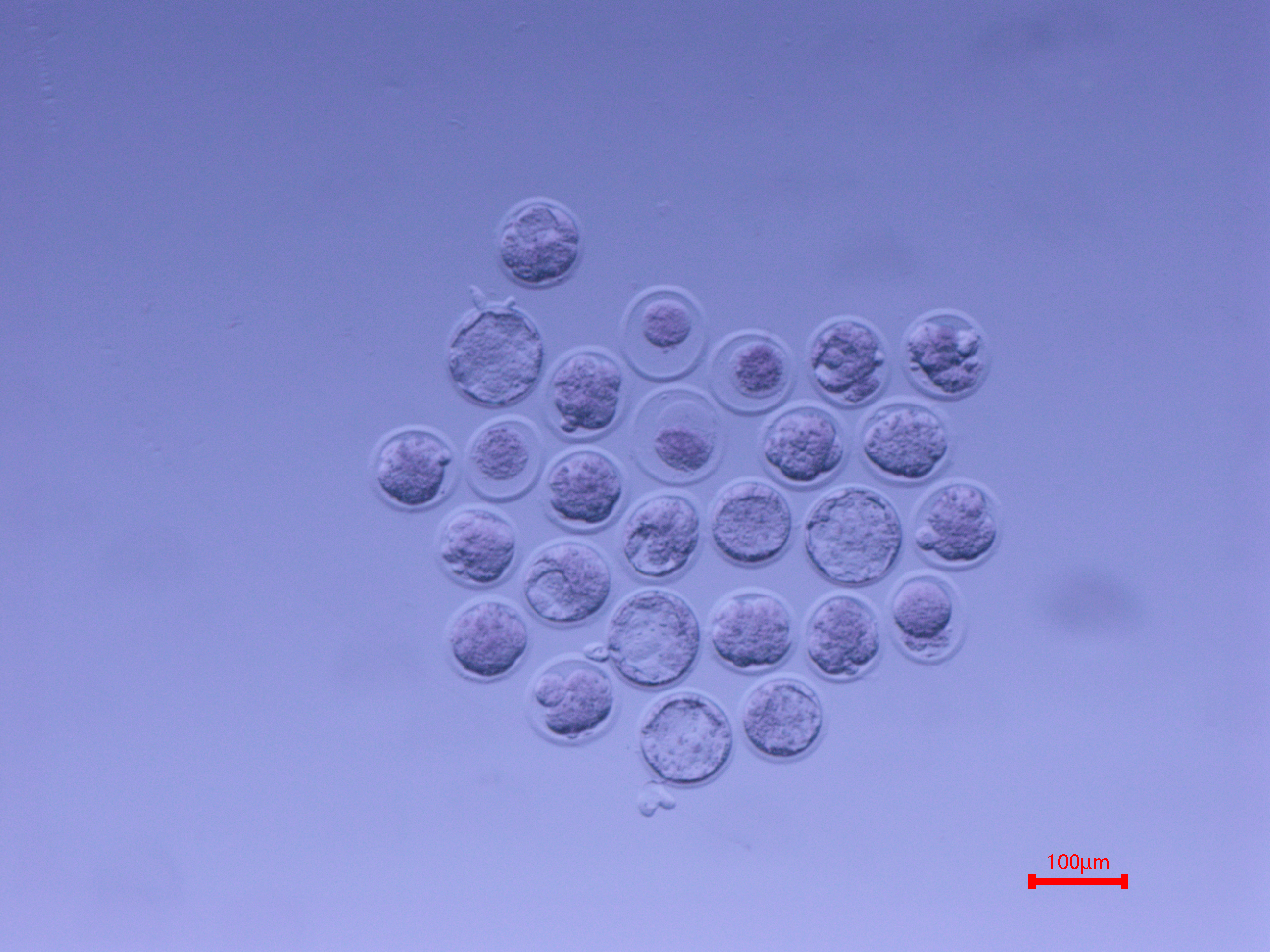

Supplement: Supplementary file 6 — Source data Fig. 5 [file 44319_2026_780_MOESM6_ESM.zip › Figure 5/Fig5C, E/72h/7.5uM-Rescue.tif]

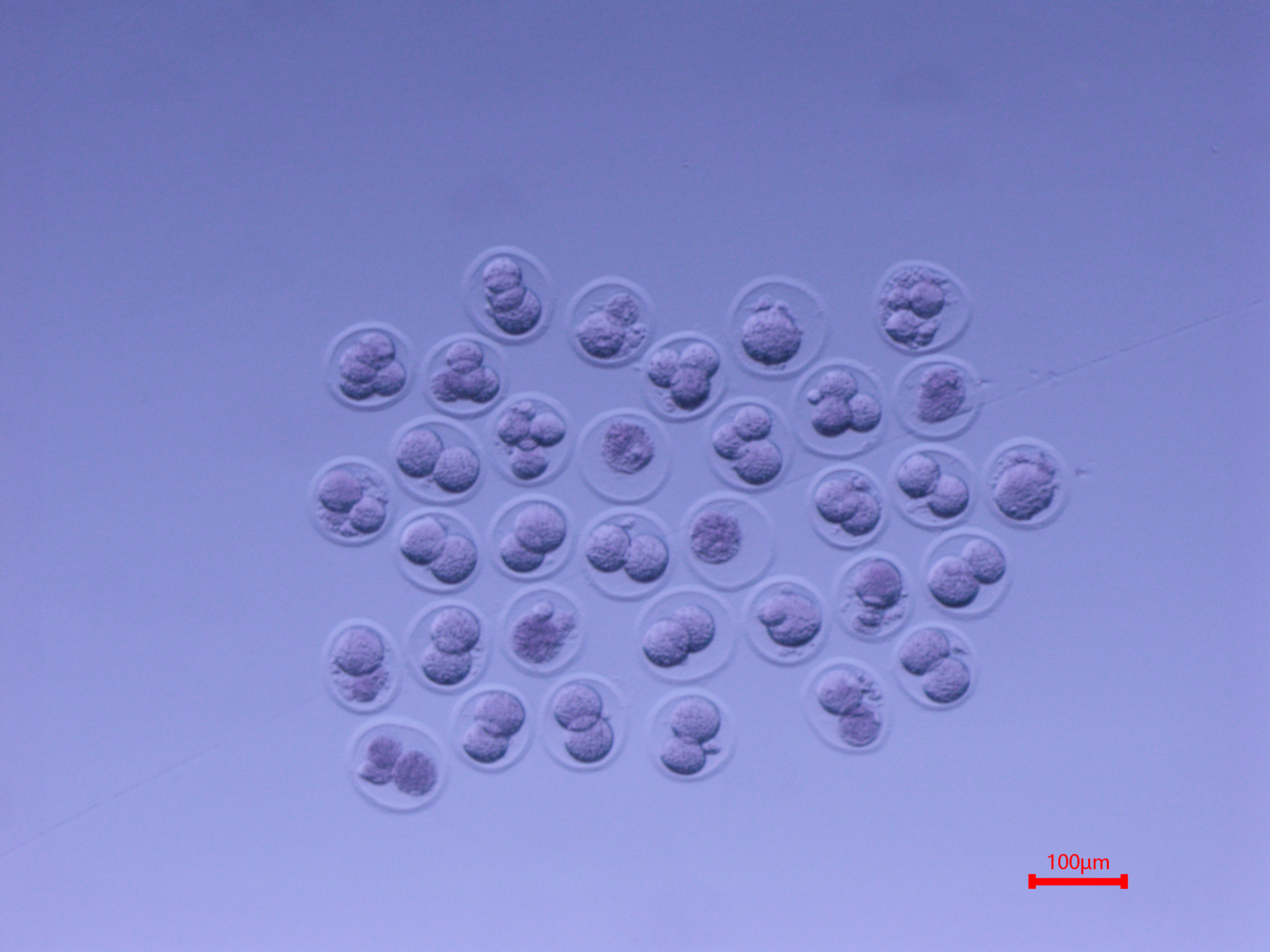

Supplement: Supplementary file 6 — Source data Fig. 5 [file 44319_2026_780_MOESM6_ESM.zip › Figure 5/Fig5C, E/72h/7.5uM.tif]

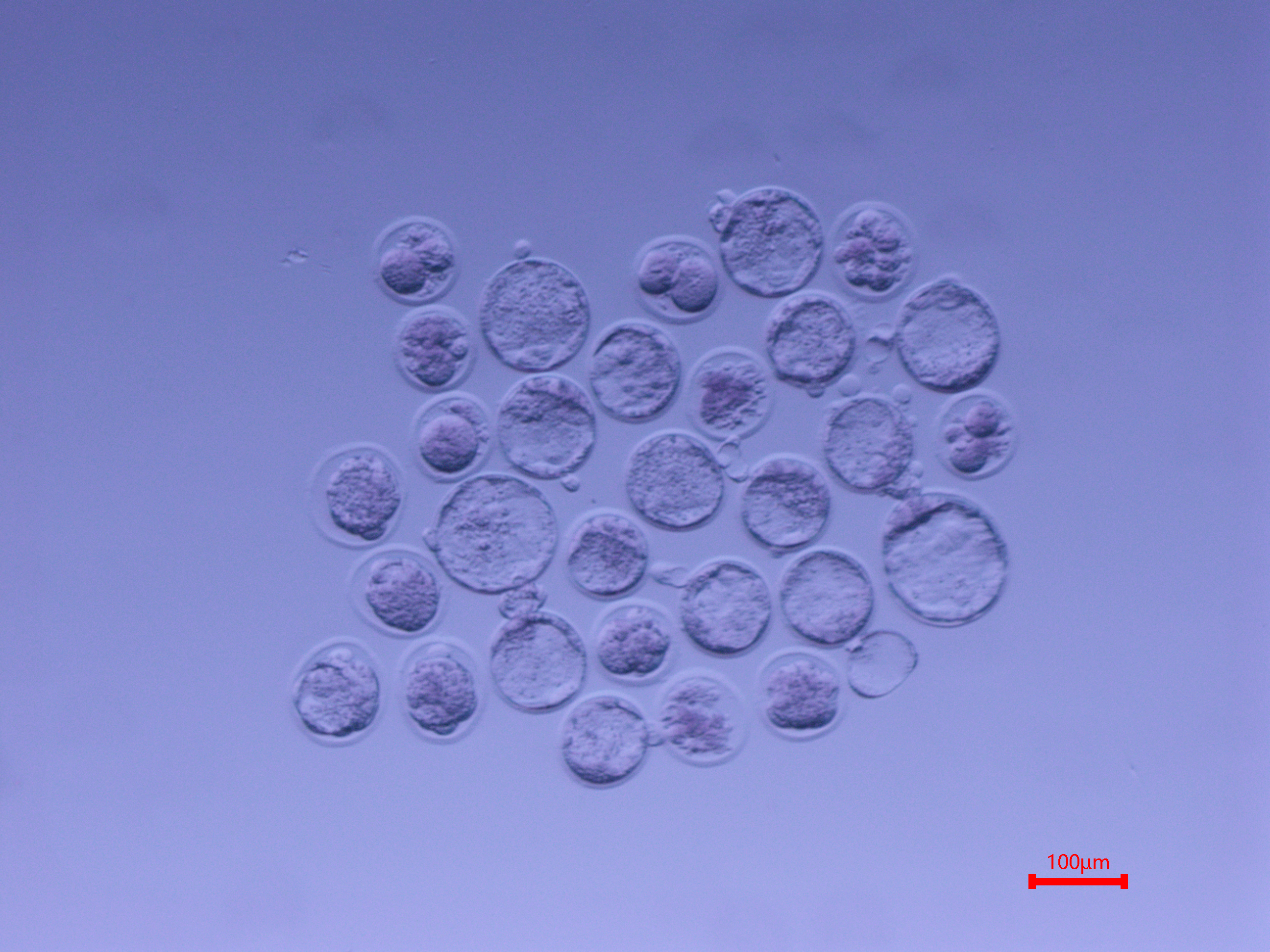

Supplement: Supplementary file 6 — Source data Fig. 5 [file 44319_2026_780_MOESM6_ESM.zip › Figure 5/Fig5C, E/72h/Control.tif]

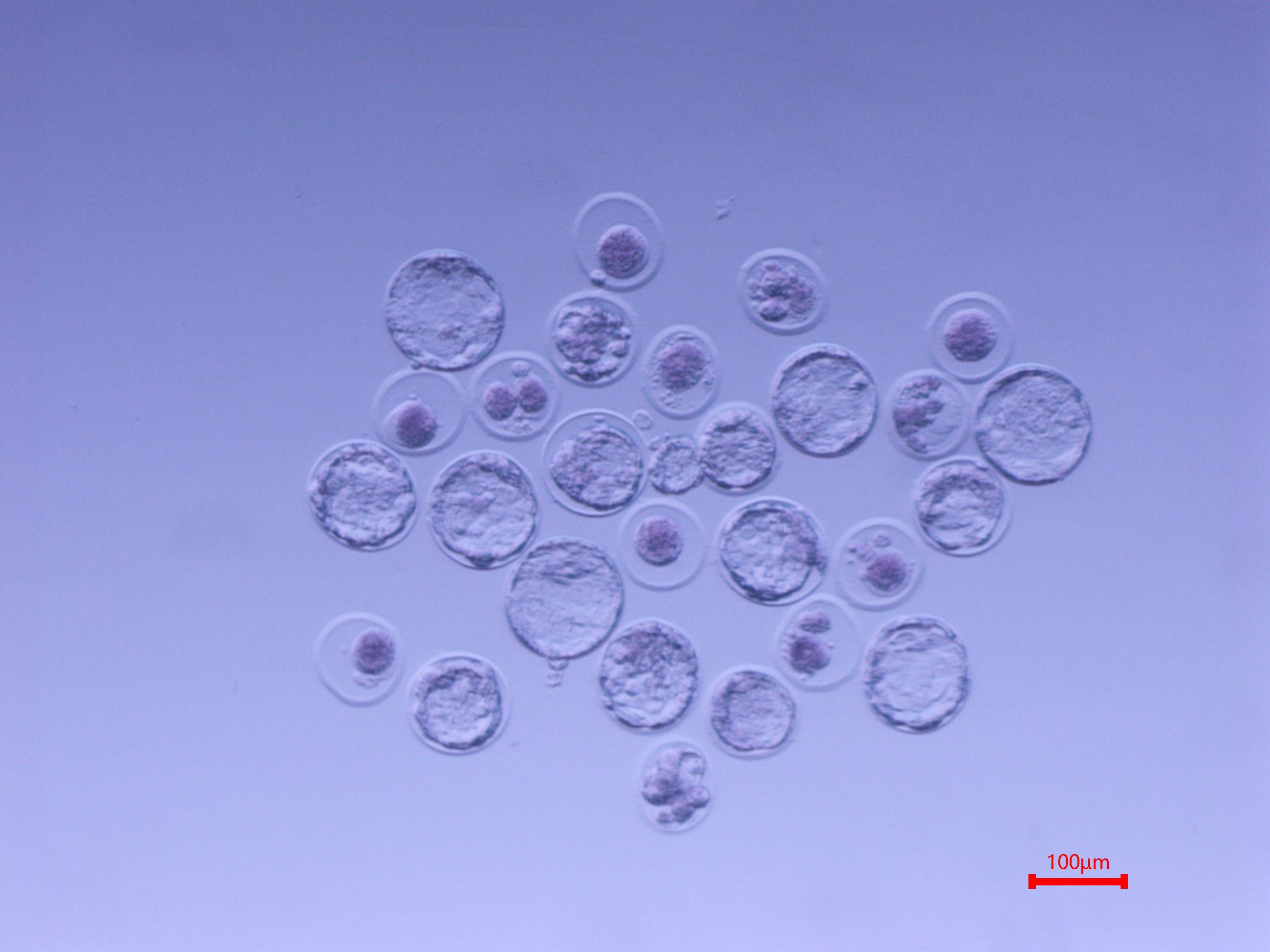

Supplement: Supplementary file 6 — Source data Fig. 5 [file 44319_2026_780_MOESM6_ESM.zip › Figure 5/Fig5C, E/96h/5uM-Rescue.tif]

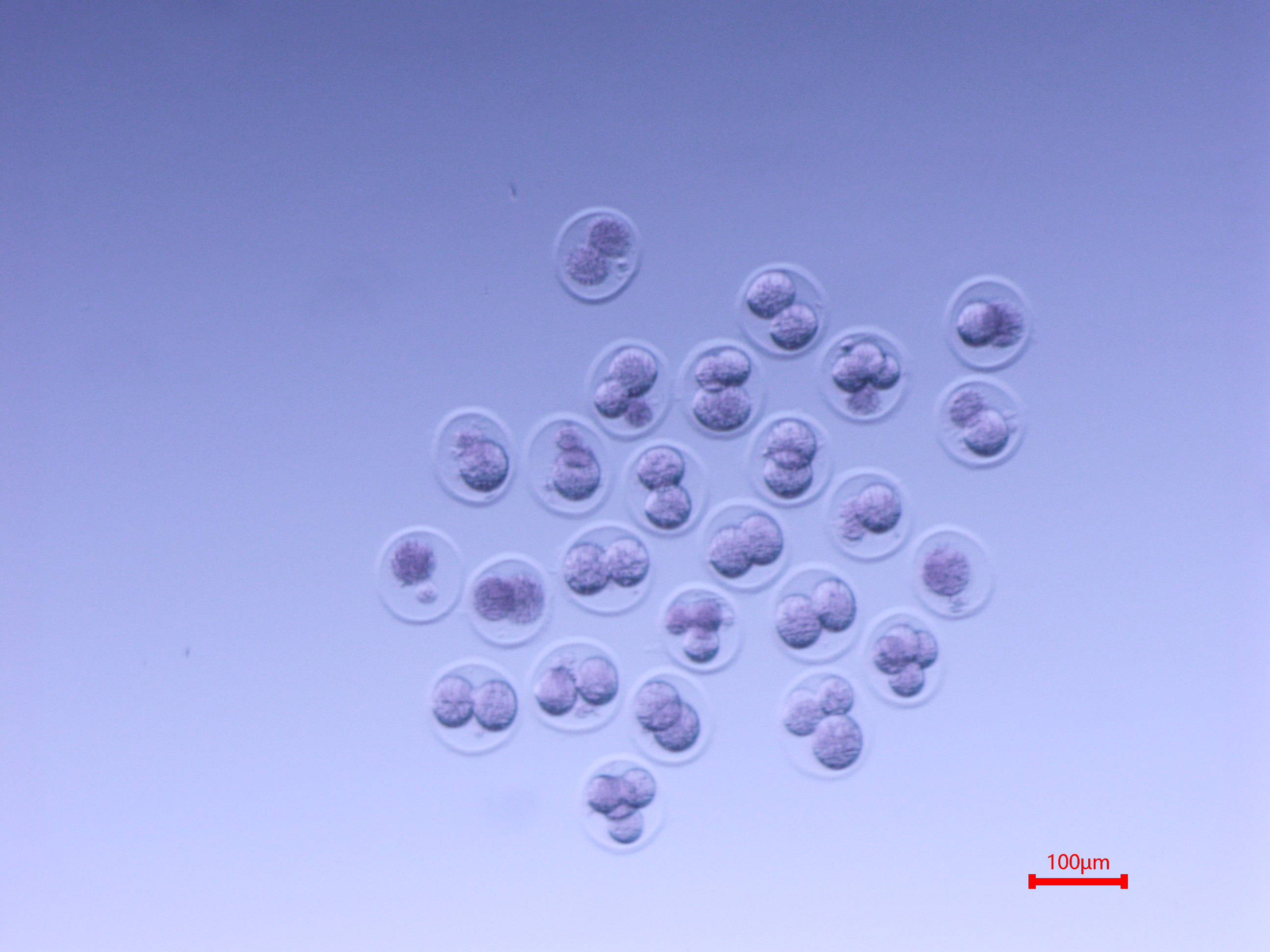

Supplement: Supplementary file 6 — Source data Fig. 5 [file 44319_2026_780_MOESM6_ESM.zip › Figure 5/Fig5C, E/96h/5uM.tif]

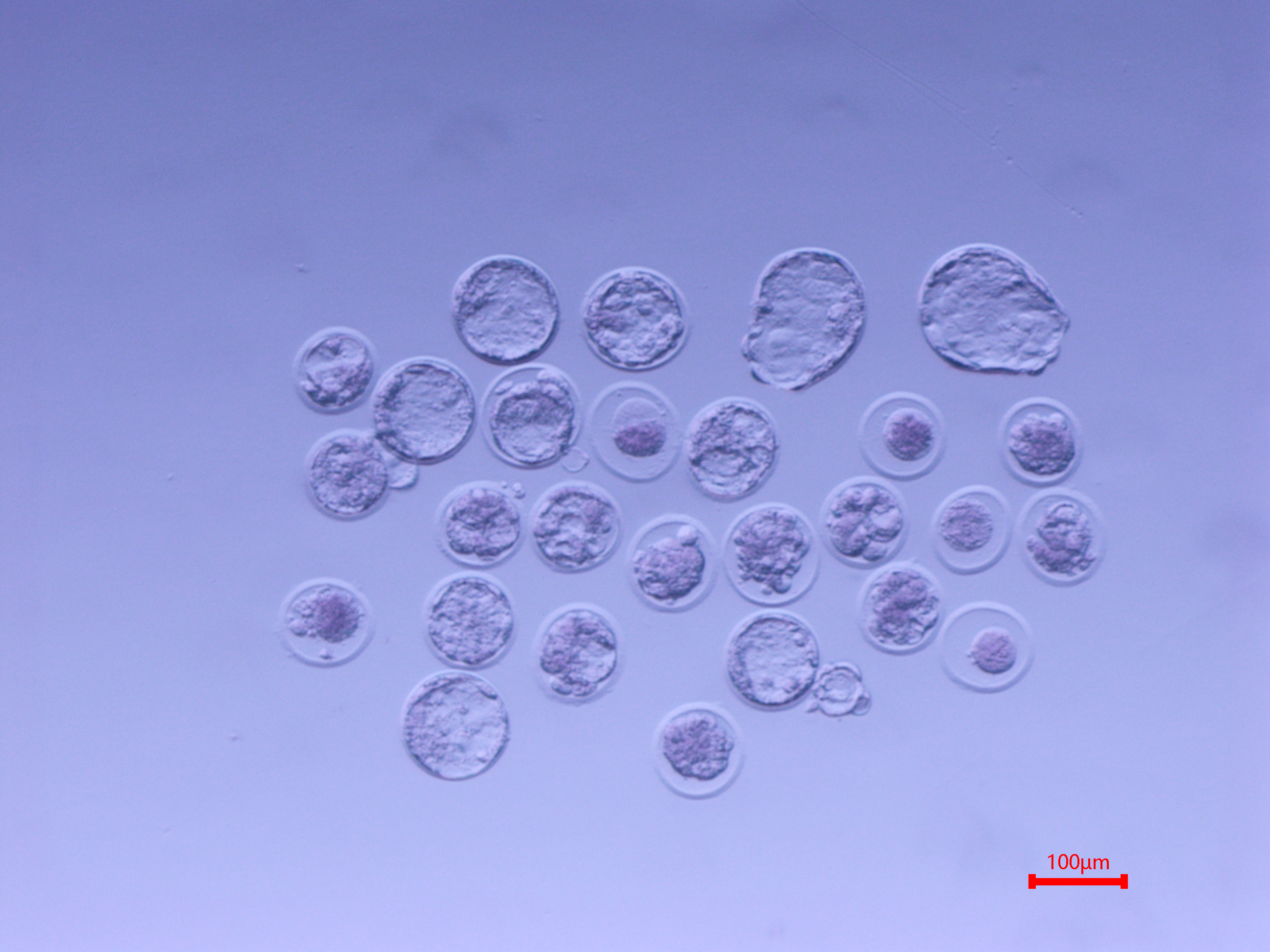

Supplement: Supplementary file 6 — Source data Fig. 5 [file 44319_2026_780_MOESM6_ESM.zip › Figure 5/Fig5C, E/96h/7.5uM-Rescue.tif]

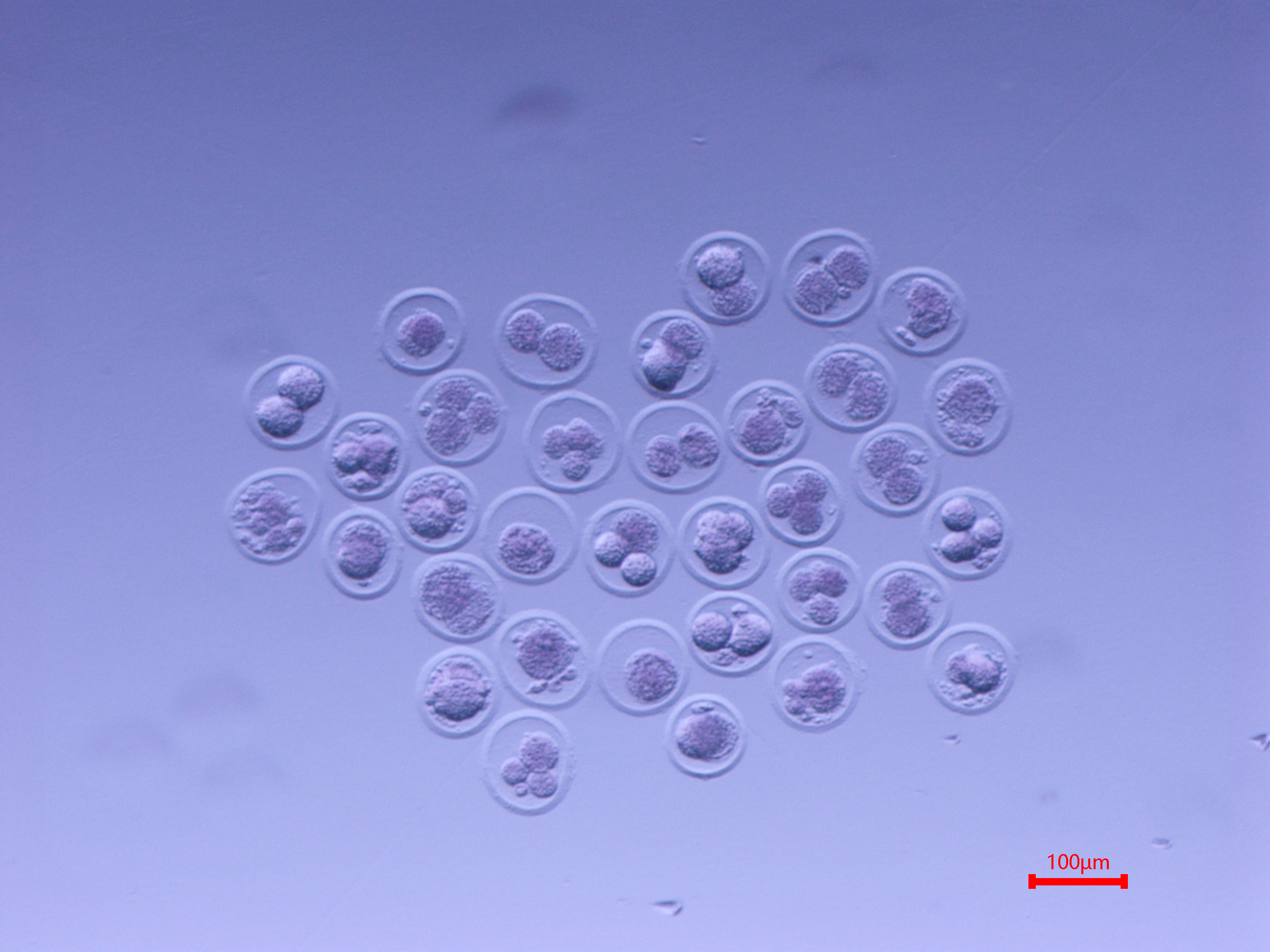

Supplement: Supplementary file 6 — Source data Fig. 5 [file 44319_2026_780_MOESM6_ESM.zip › Figure 5/Fig5C, E/96h/7.5uM.tif]

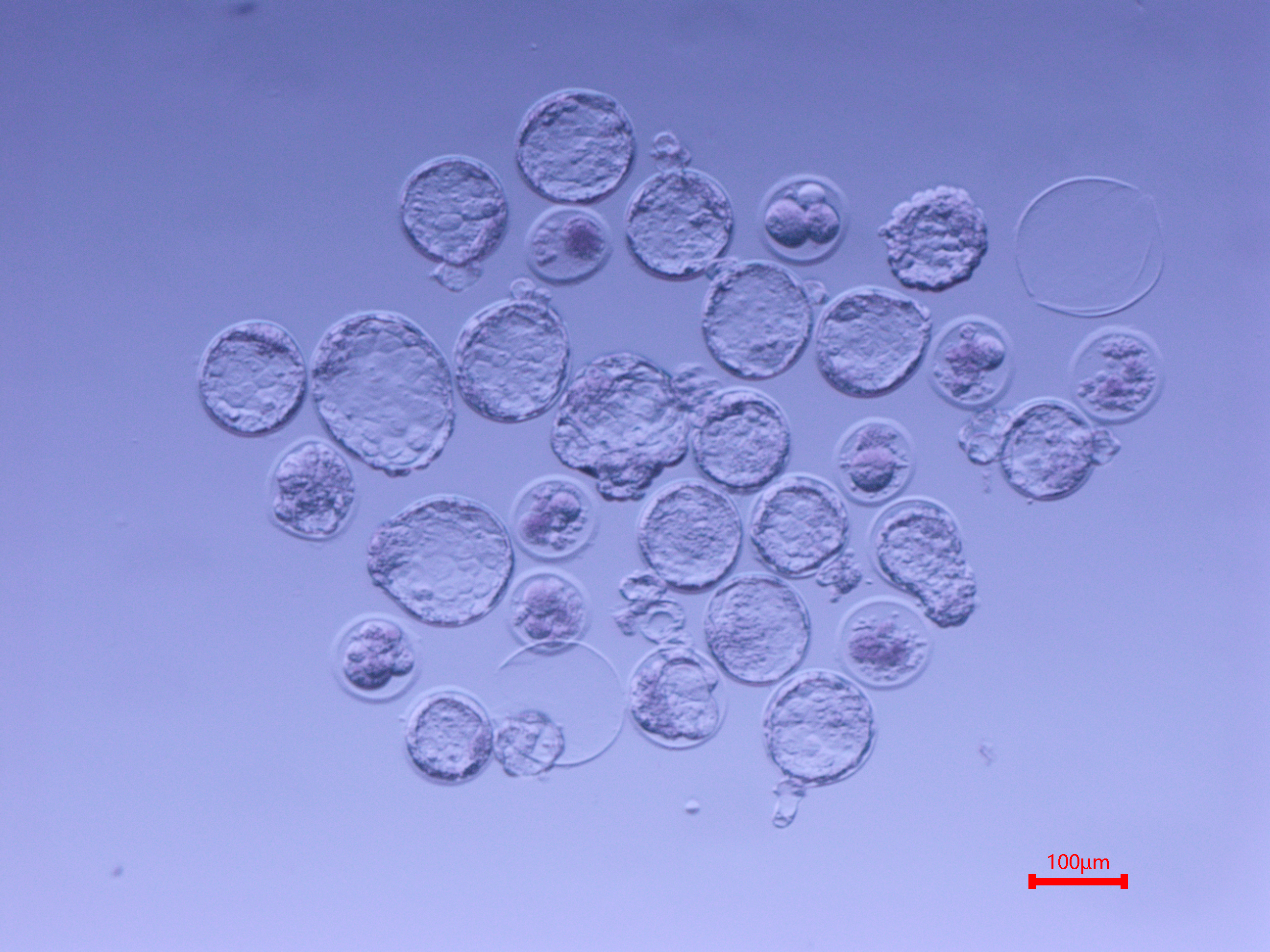

Supplement: Supplementary file 6 — Source data Fig. 5 [file 44319_2026_780_MOESM6_ESM.zip › Figure 5/Fig5C, E/96h/Control.tif]

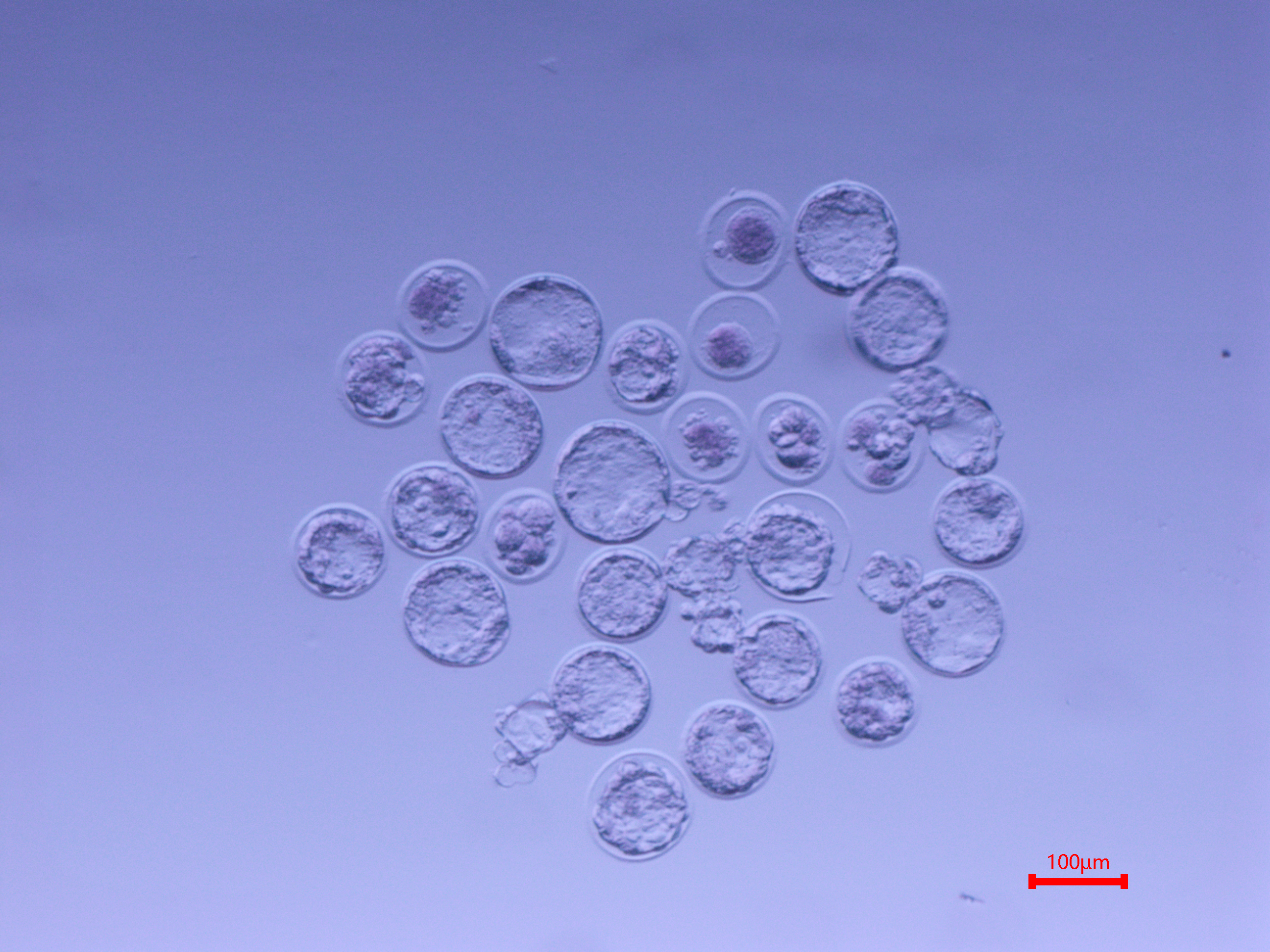

Supplement: Supplementary file 6 — Source data Fig. 5 [file 44319_2026_780_MOESM6_ESM.zip › Figure 5/Fig5C, E/96h/DMSO.tif]

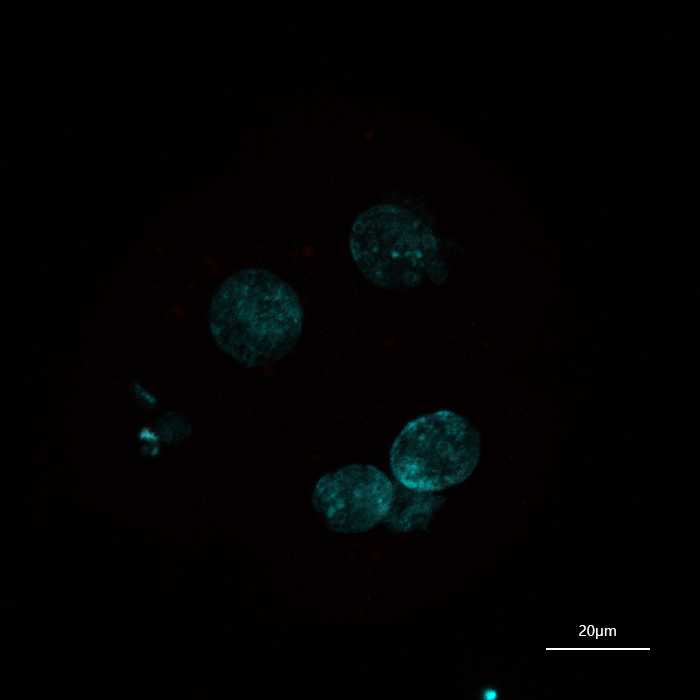

Supplement: Supplementary file 9 — Figure EV3 Source Data [file 44319_2026_780_MOESM9_ESM.zip › Figure EV3/Fig EV3C/4cell-2001-MaxIP_hp_RGB.tif]

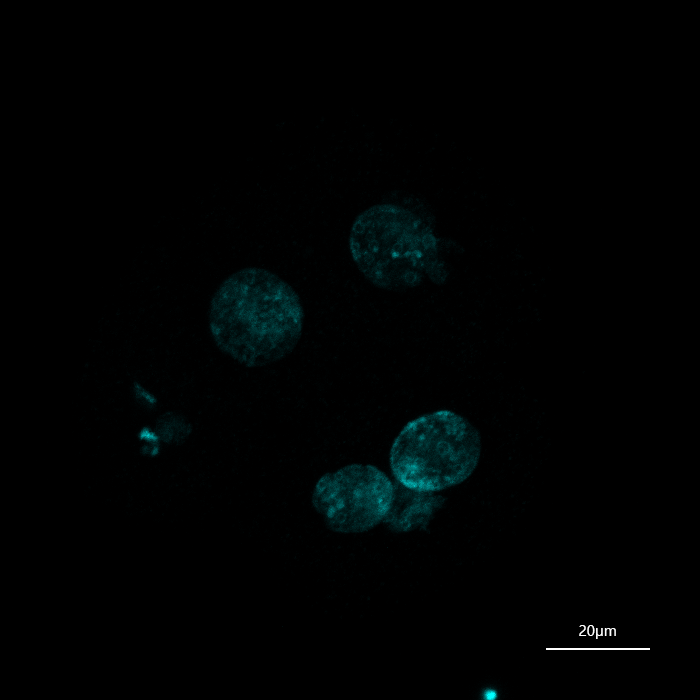

Supplement: Supplementary file 9 — Figure EV3 Source Data [file 44319_2026_780_MOESM9_ESM.zip › Figure EV3/Fig EV3C/4cell-2001-MaxIP_hp_RGB_DAPI.tif]

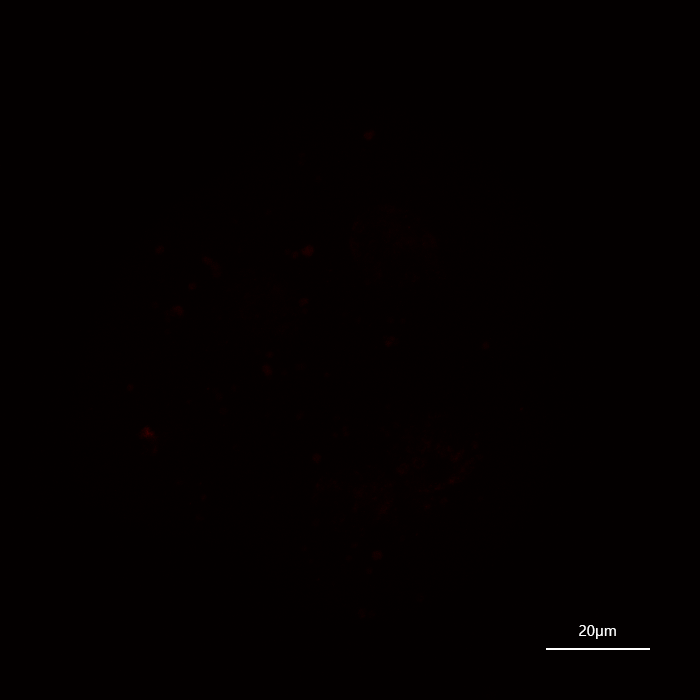

Supplement: Supplementary file 9 — Figure EV3 Source Data [file 44319_2026_780_MOESM9_ESM.zip › Figure EV3/Fig EV3C/4cell-2001-MaxIP_hp_RGB_H4K20me1.tif]

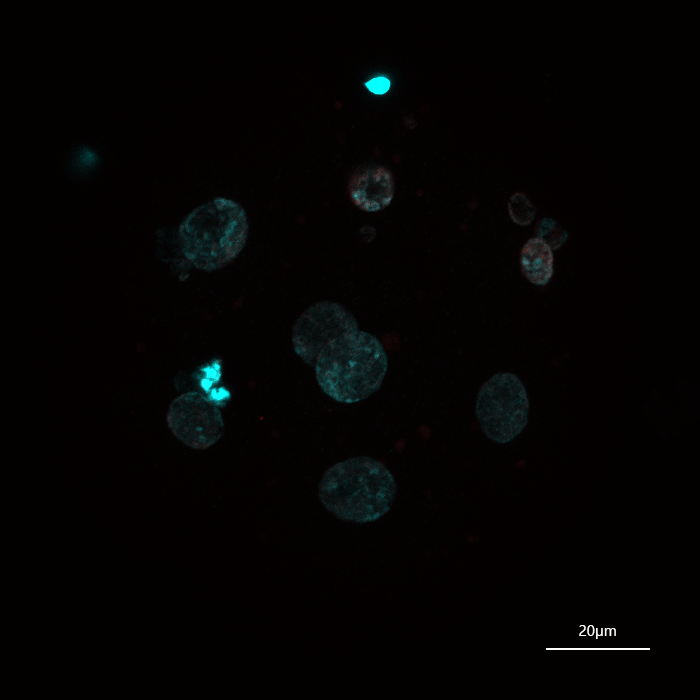

Supplement: Supplementary file 9 — Figure EV3 Source Data [file 44319_2026_780_MOESM9_ESM.zip › Figure EV3/Fig EV3C/8cell-1008-MaxIP_hp_RGB.tif]

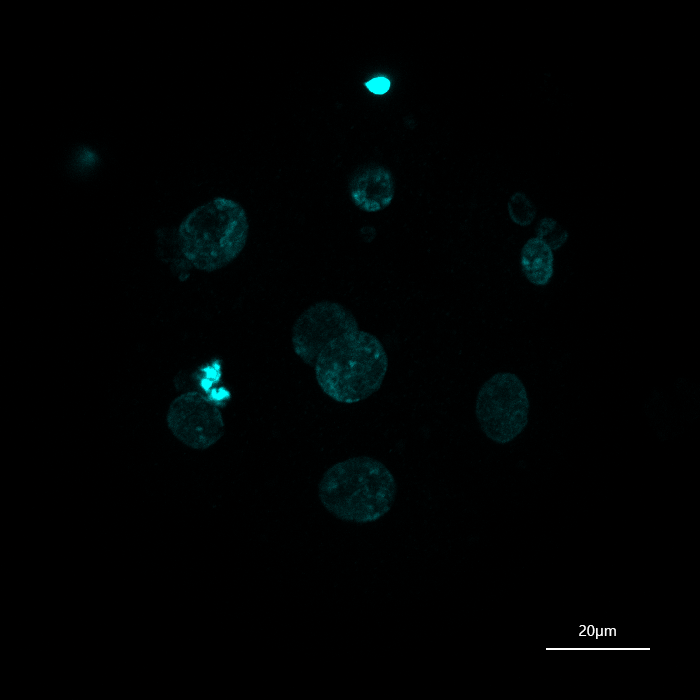

Supplement: Supplementary file 9 — Figure EV3 Source Data [file 44319_2026_780_MOESM9_ESM.zip › Figure EV3/Fig EV3C/8cell-1008-MaxIP_hp_RGB_DAPI.tif]

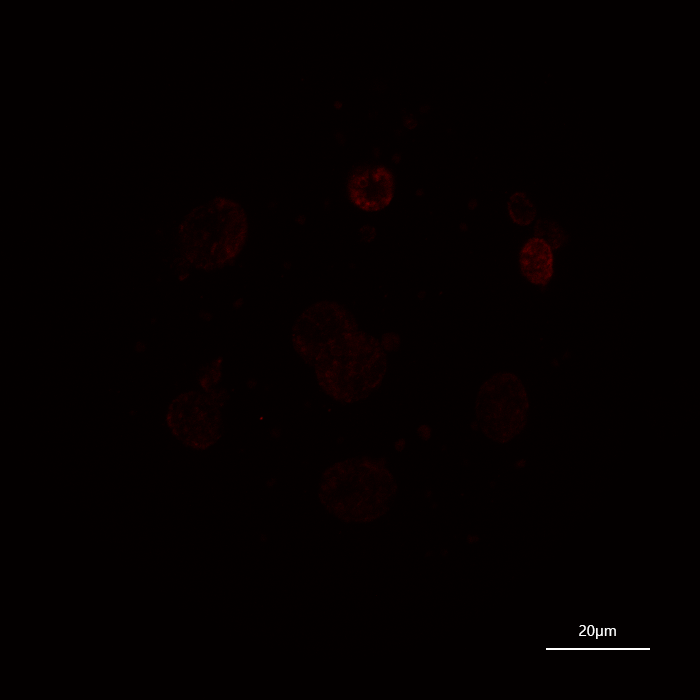

Supplement: Supplementary file 9 — Figure EV3 Source Data [file 44319_2026_780_MOESM9_ESM.zip › Figure EV3/Fig EV3C/8cell-1008-MaxIP_hp_RGB_H4K20me1.tif]

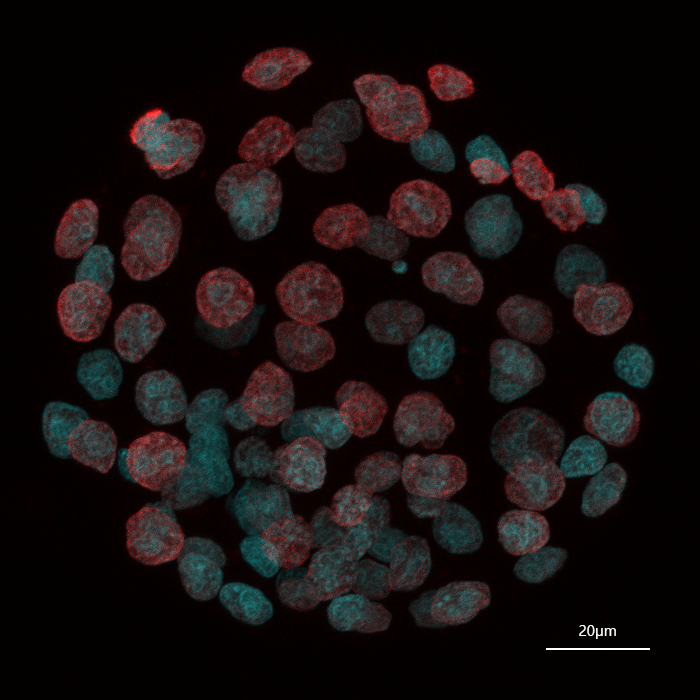

Supplement: Supplementary file 9 — Figure EV3 Source Data [file 44319_2026_780_MOESM9_ESM.zip › Figure EV3/Fig EV3C/blast-1007-MaxIP_hp_RGB.tif]

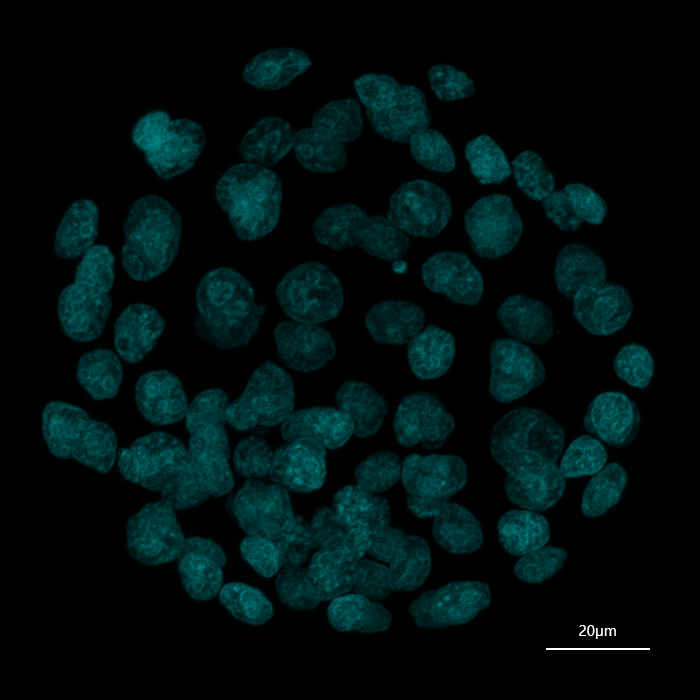

Supplement: Supplementary file 9 — Figure EV3 Source Data [file 44319_2026_780_MOESM9_ESM.zip › Figure EV3/Fig EV3C/blast-1007-MaxIP_hp_RGB_DAPI.tif]

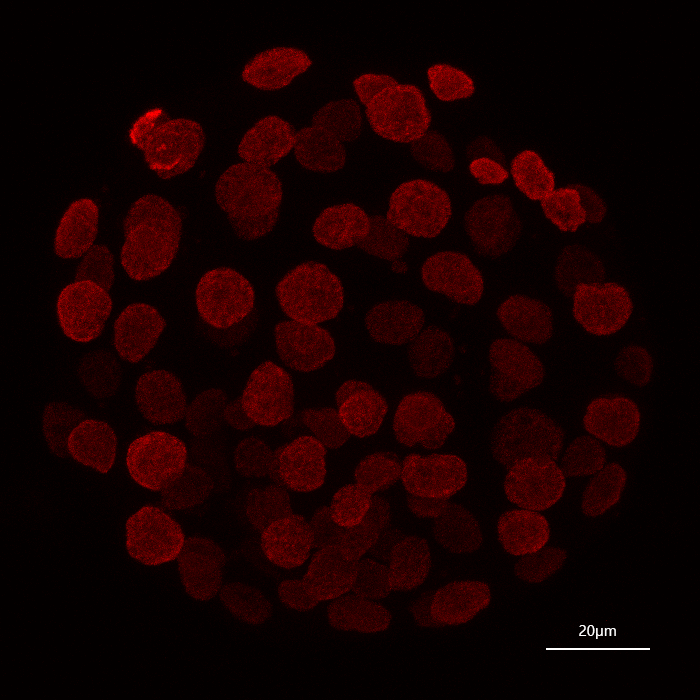

Supplement: Supplementary file 9 — Figure EV3 Source Data [file 44319_2026_780_MOESM9_ESM.zip › Figure EV3/Fig EV3C/blast-1007-MaxIP_hp_RGB_H4K20me1.tif]

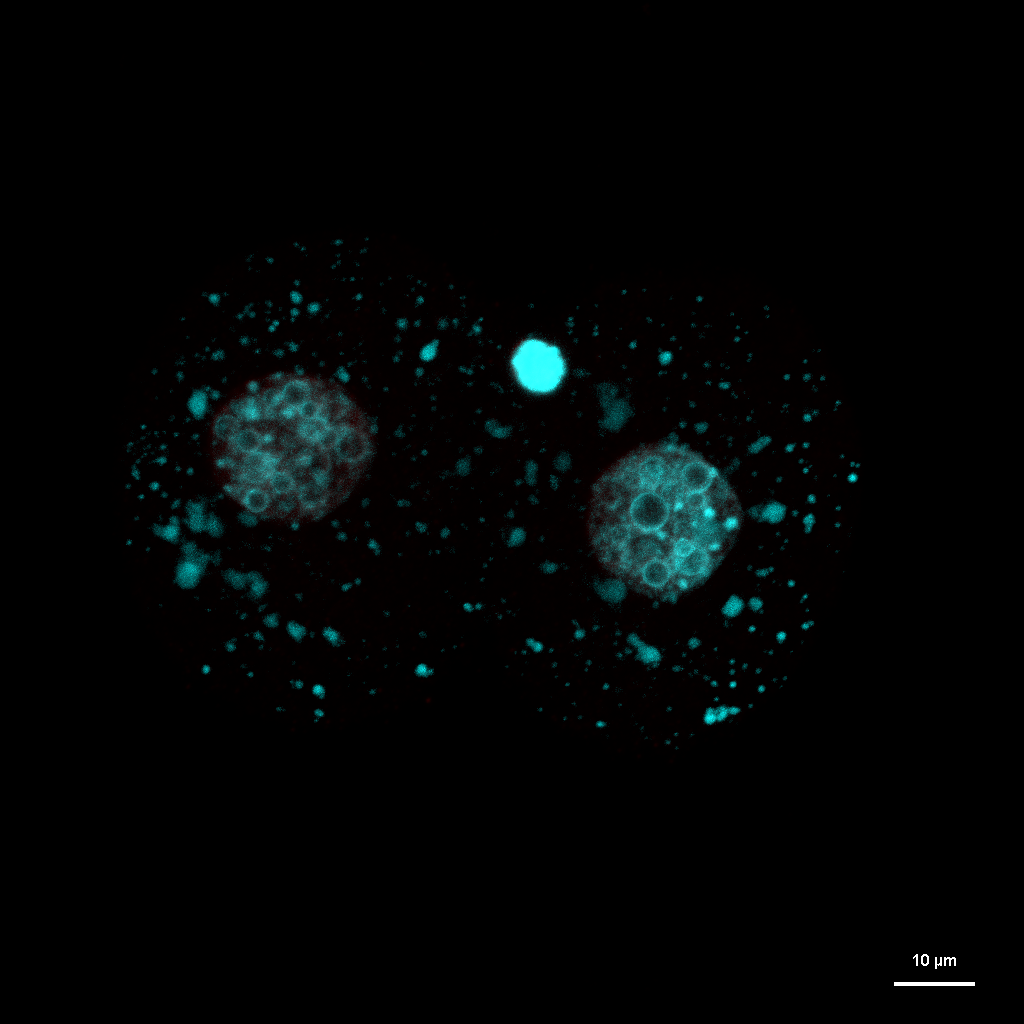

Supplement: Supplementary file 10 — Figure EV5 Source Data [file 44319_2026_780_MOESM10_ESM.zip › Figure EV5/Fig EV5B/5-008-MaxIP_hp_RGB.tif]

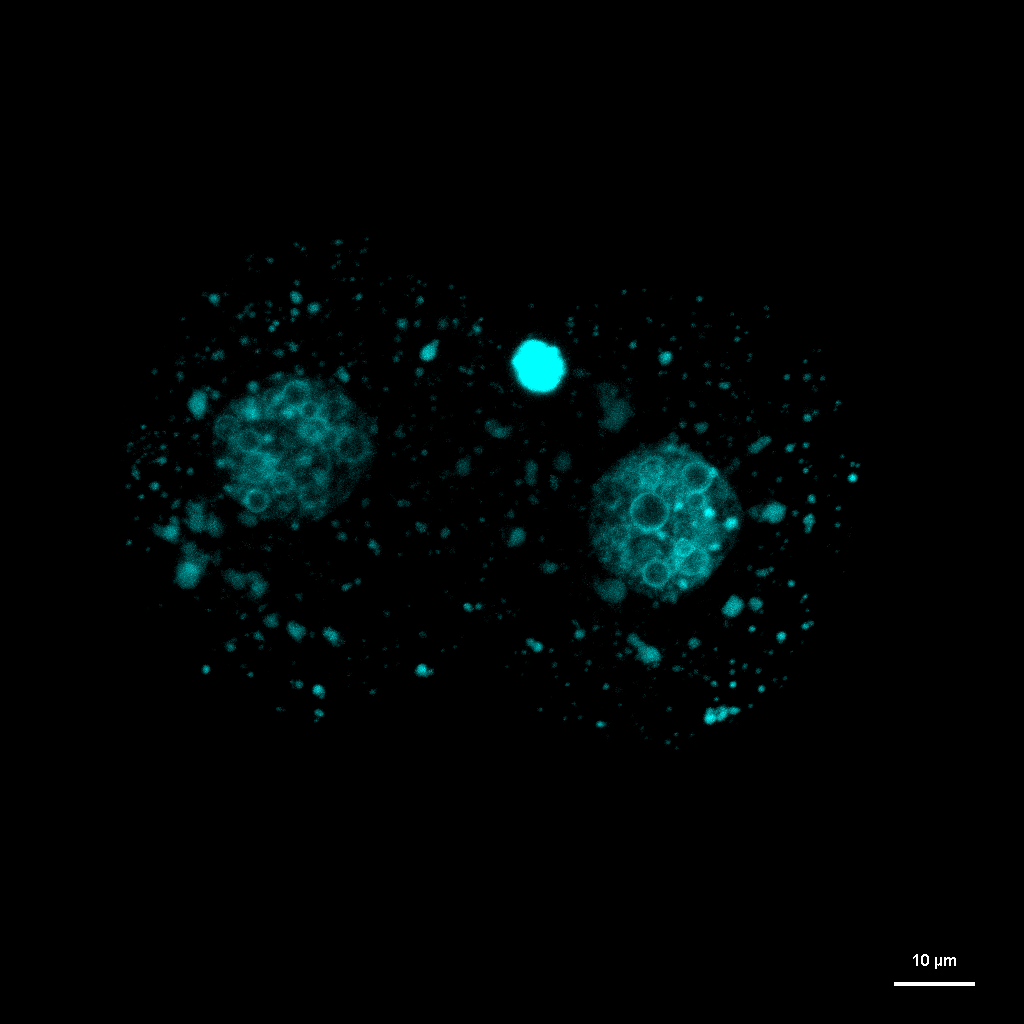

Supplement: Supplementary file 10 — Figure EV5 Source Data [file 44319_2026_780_MOESM10_ESM.zip › Figure EV5/Fig EV5B/5-008-MaxIP_hp_RGB_DAPI.tif]

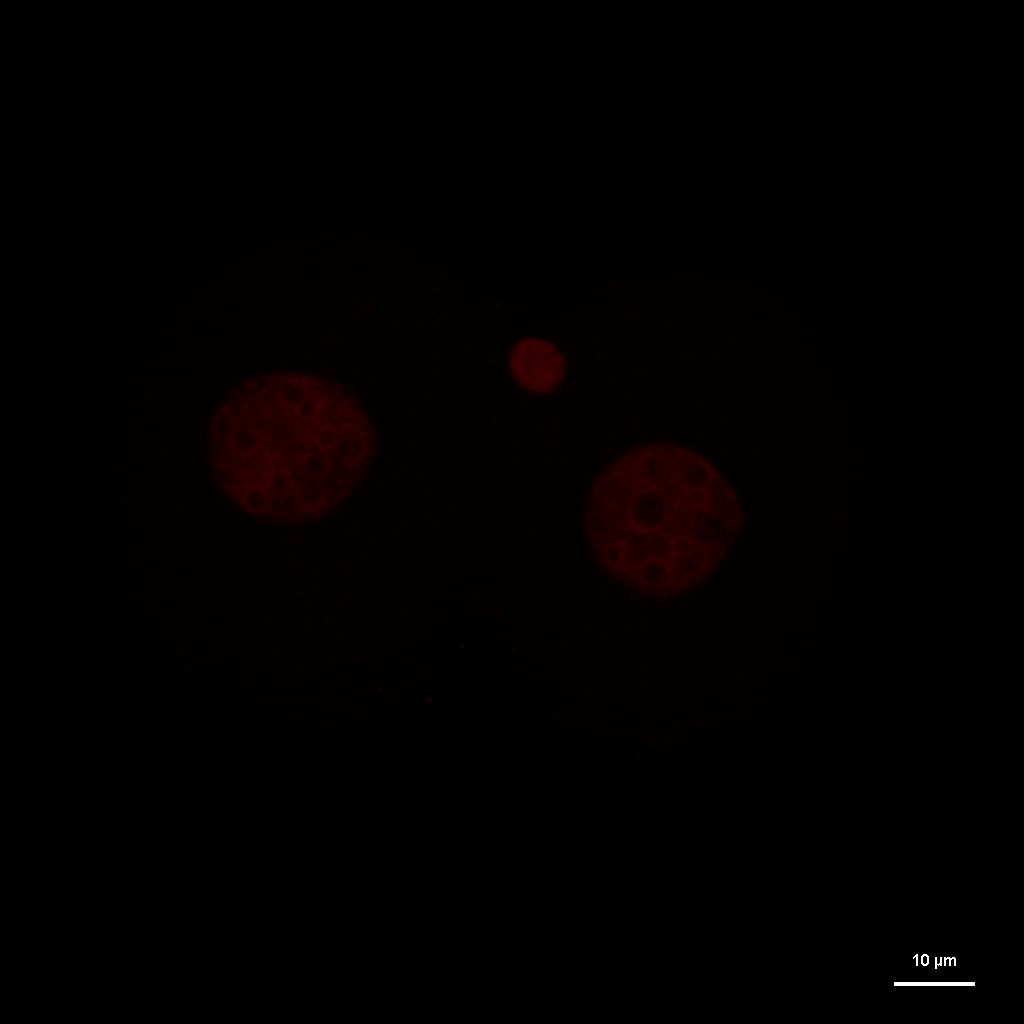

Supplement: Supplementary file 10 — Figure EV5 Source Data [file 44319_2026_780_MOESM10_ESM.zip › Figure EV5/Fig EV5B/5-008-MaxIP_hp_RGB_H4K20me1.tif]

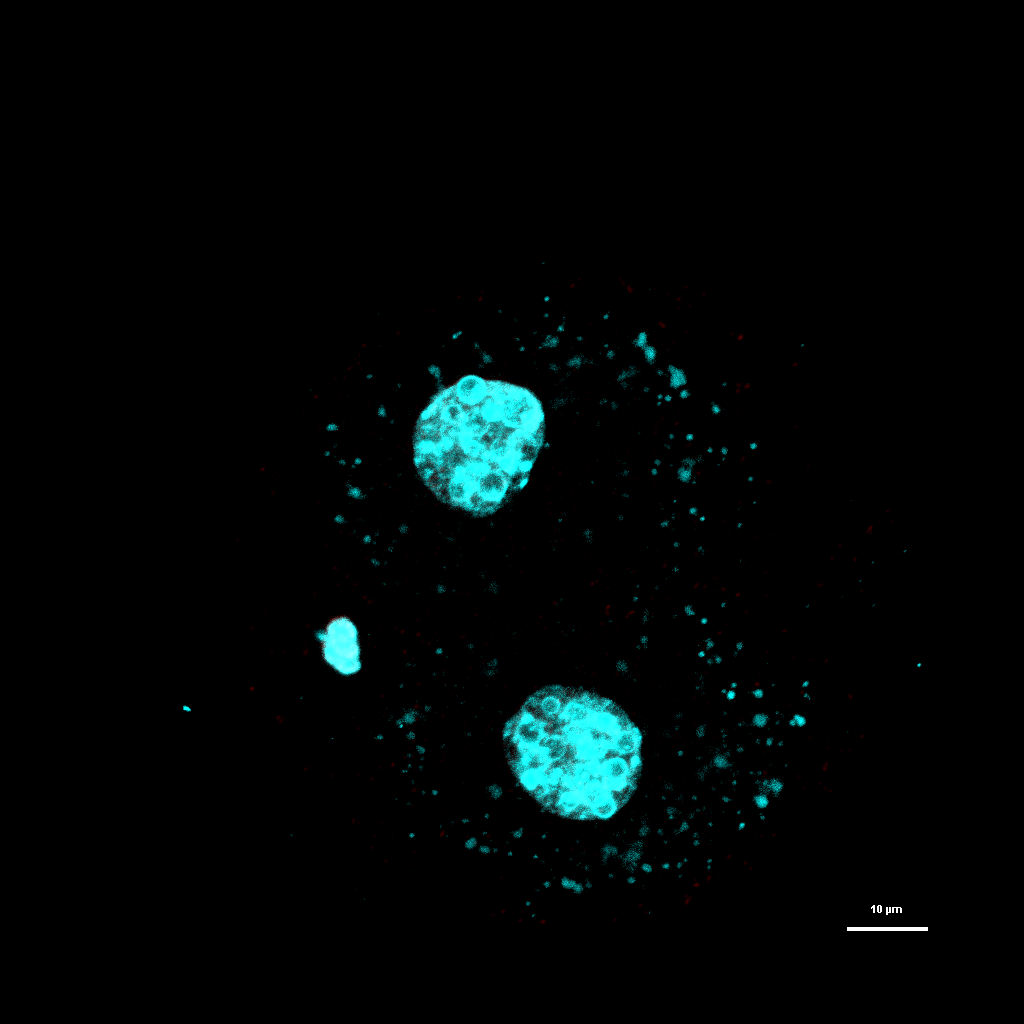

Supplement: Supplementary file 10 — Figure EV5 Source Data [file 44319_2026_780_MOESM10_ESM.zip › Figure EV5/Fig EV5B/7.5-006-MaxIP_hp_RGB.tif]

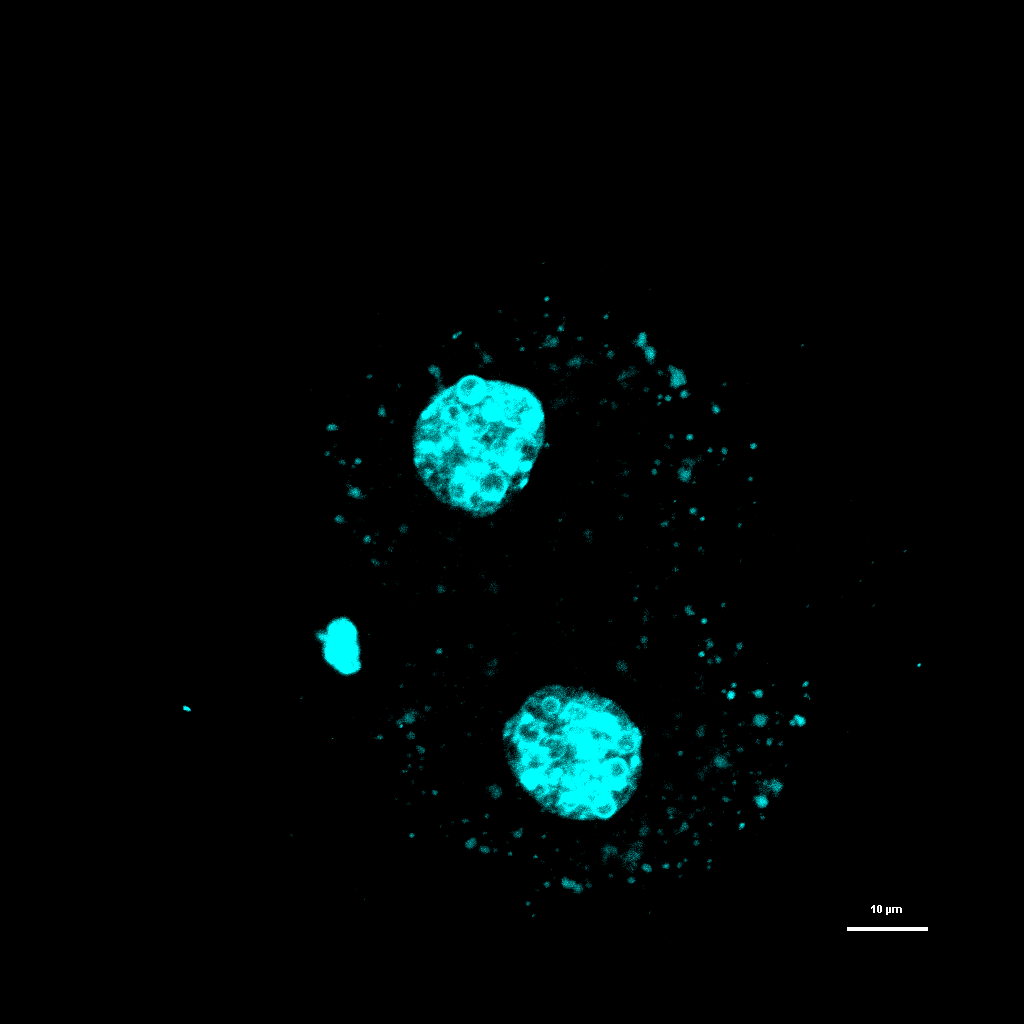

Supplement: Supplementary file 10 — Figure EV5 Source Data [file 44319_2026_780_MOESM10_ESM.zip › Figure EV5/Fig EV5B/7.5-006-MaxIP_hp_RGB_DAPI.tif]

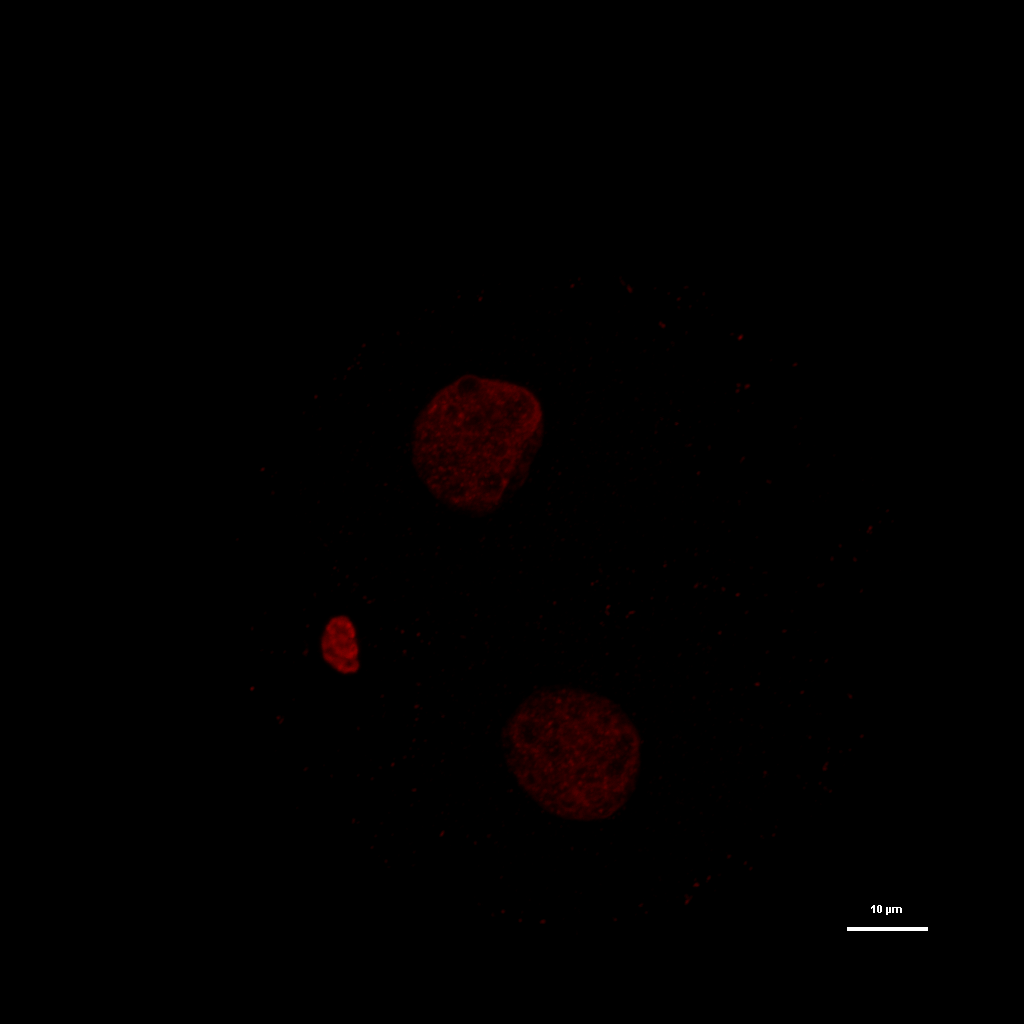

Supplement: Supplementary file 10 — Figure EV5 Source Data [file 44319_2026_780_MOESM10_ESM.zip › Figure EV5/Fig EV5B/7.5-006-MaxIP_hp_RGB_H4K20me1.tif]

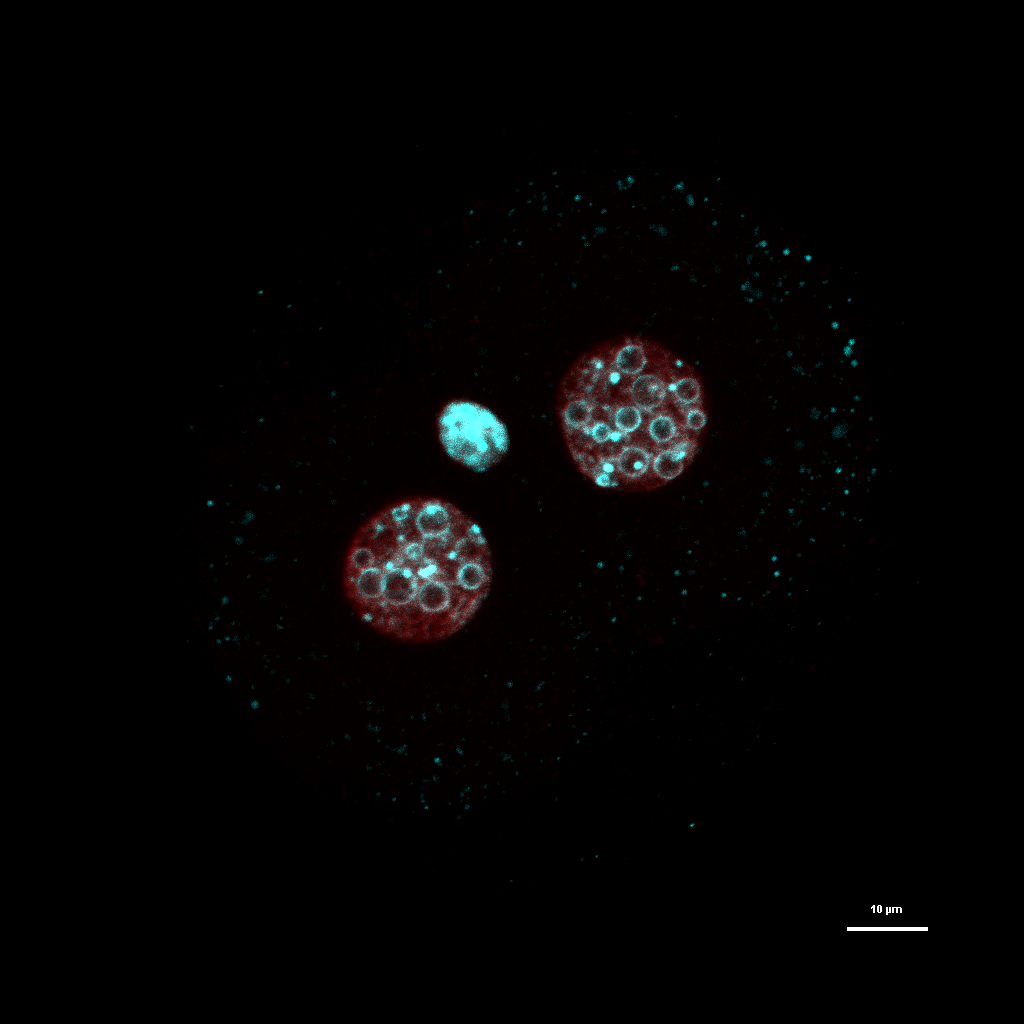

Supplement: Supplementary file 10 — Figure EV5 Source Data [file 44319_2026_780_MOESM10_ESM.zip › Figure EV5/Fig EV5B/CON-004-MaxIP_hp_RGB.tif]

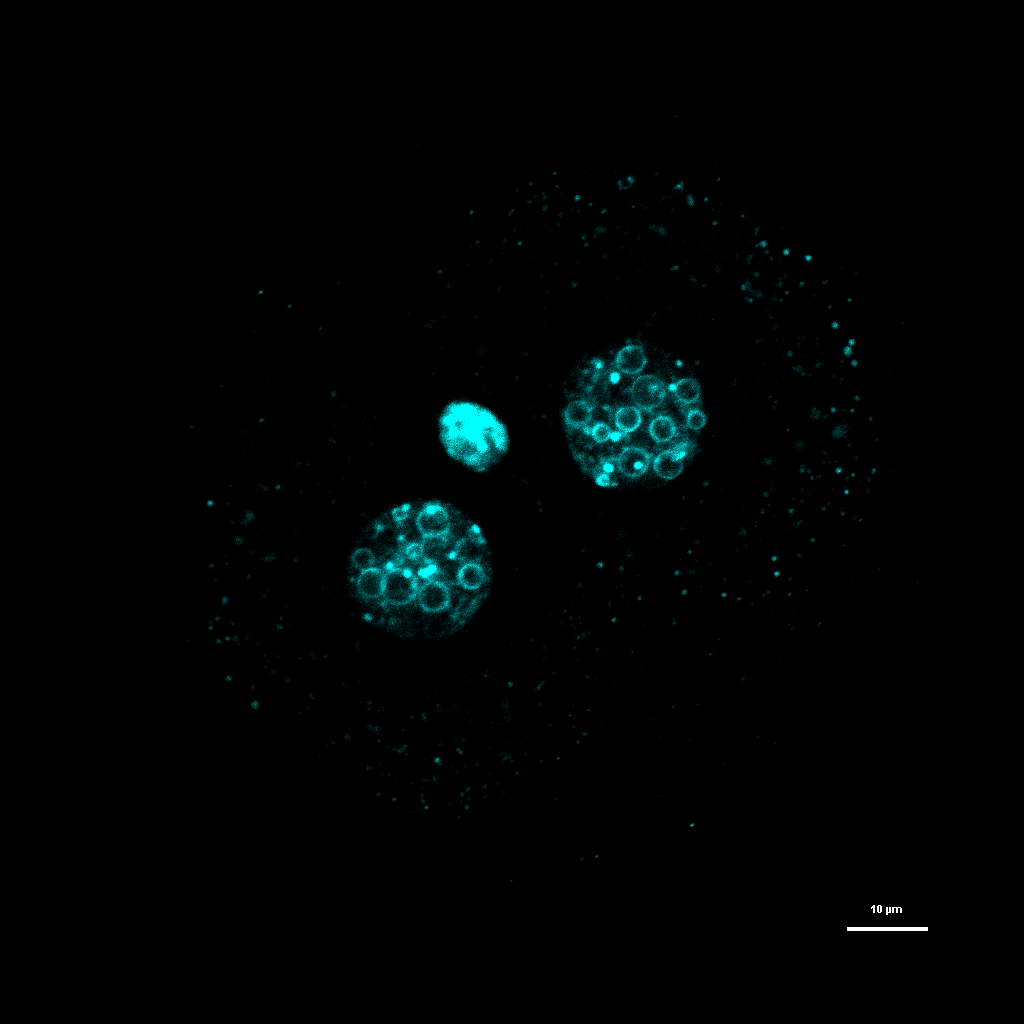

Supplement: Supplementary file 10 — Figure EV5 Source Data [file 44319_2026_780_MOESM10_ESM.zip › Figure EV5/Fig EV5B/CON-004-MaxIP_hp_RGB_DAPI.tif]

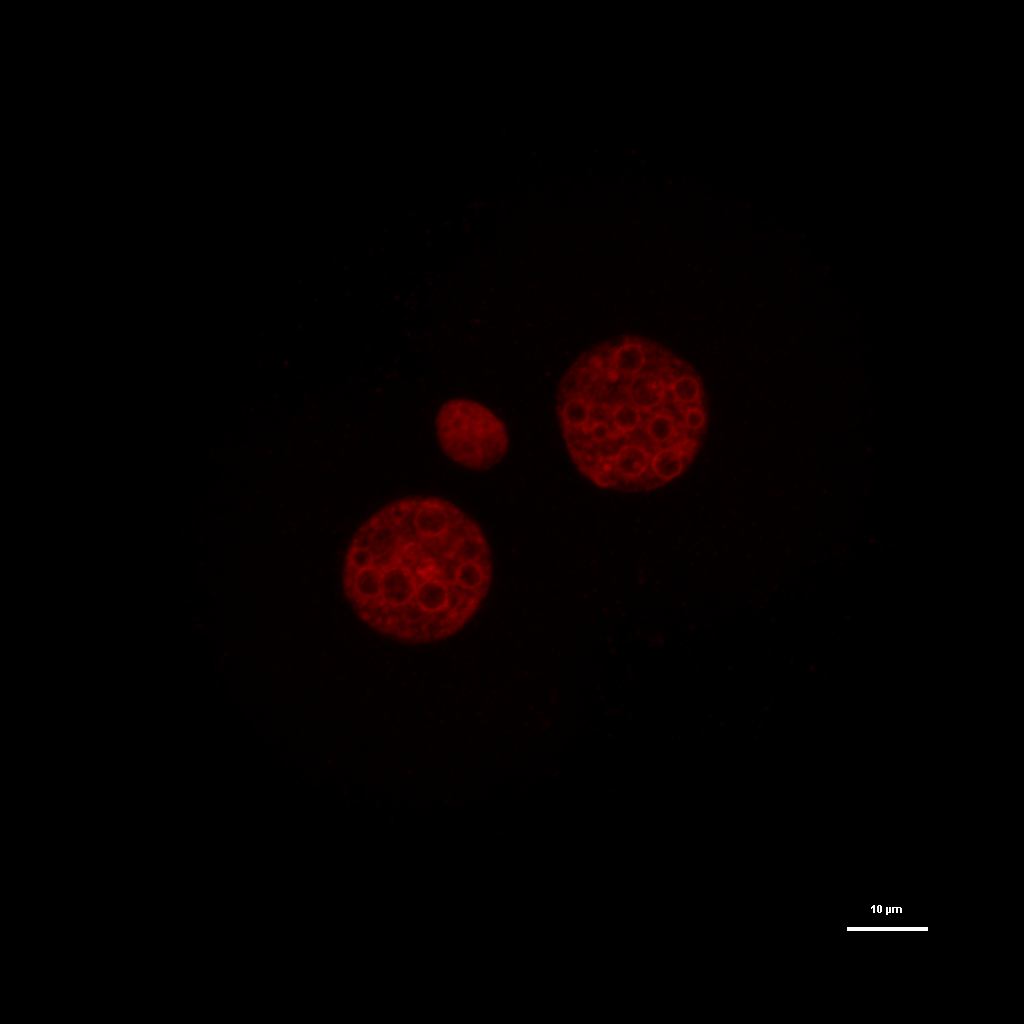

Supplement: Supplementary file 10 — Figure EV5 Source Data [file 44319_2026_780_MOESM10_ESM.zip › Figure EV5/Fig EV5B/CON-004-MaxIP_hp_RGB_H4K20me1.tif]
