# Supplementary material for: The reprogramming and function of H4K20me1 during early embryo development
Source: EMBO Rep. 2026 Apr 22;27(11):3050–77. doi: 10.1038/s44319-026-00780-x (PMC13261155; doi:10.1038/s44319-026-00780-x)
Supplement: Supplementary file 12 — Expanded View Figures [file 44319_2026_780_MOESM12_ESM.pdf]

Expanded View Figures

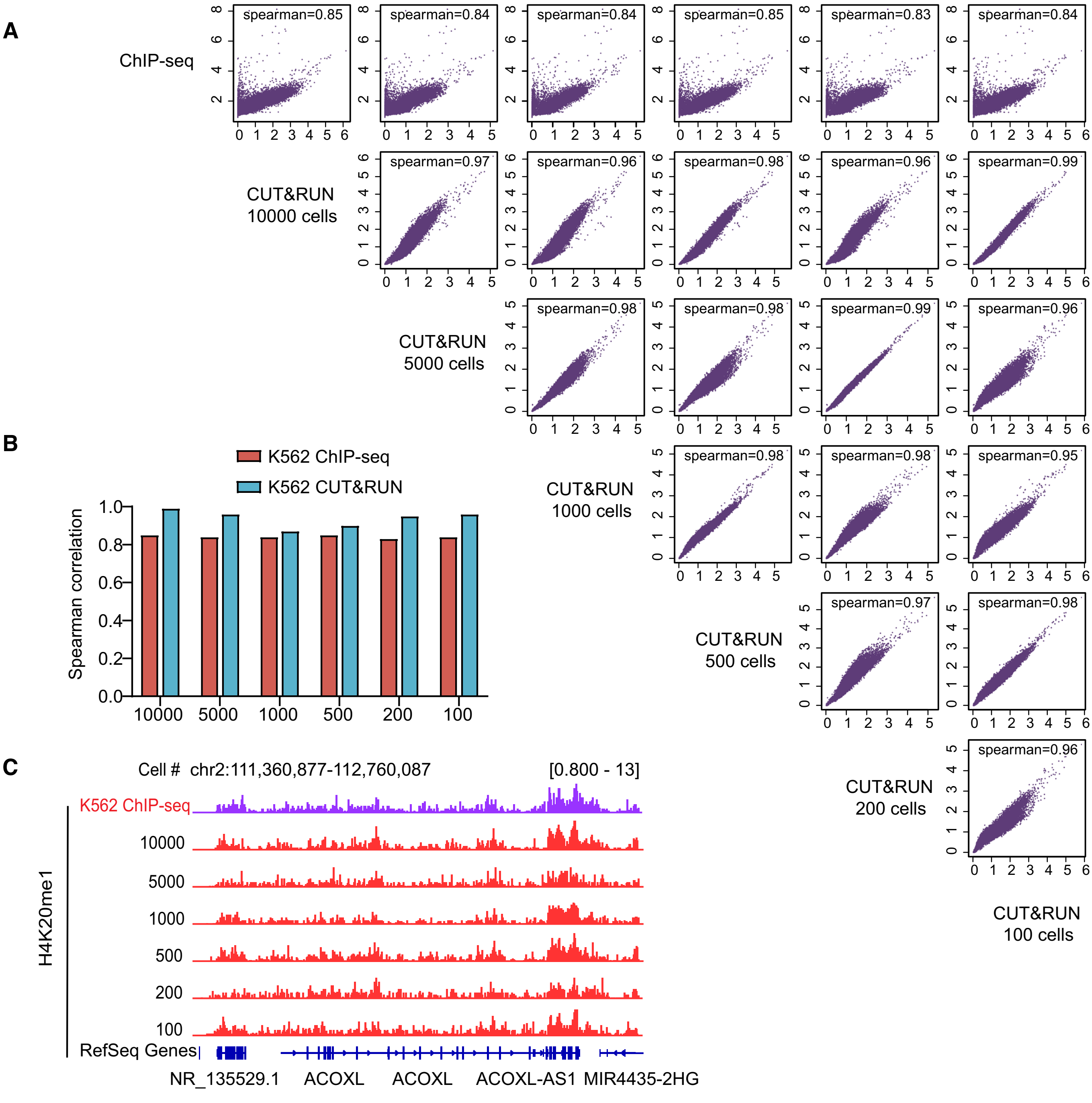

Figure EV1. Validation of the low-input CUT&RUN approach.

(**A**) Spearman correlation analysis showing that high correlations between different cell inputs in K562 cells of CUT&RUN data and ChIP-seq data (obtained from GSE29611) of H4K20me1. The axes in the plots represent log2(normalized signal + 1) values. (**B**) Correlations were assessed between: (i) H4K20me1 CUT&RUN data (from the indicated K562 cell numbers) and the ChIP-seq data (blue bars), and (ii) two biological replicates of the CUT&RUN data at the same cell number (red bars). (**C**) Landscapes of different K562 cell input amounts of H4K20me1 detected with CUT&RUN and a large number of K562 cell amounts of H4K20me1 detected by ChIP-seq.

© The Author(s)

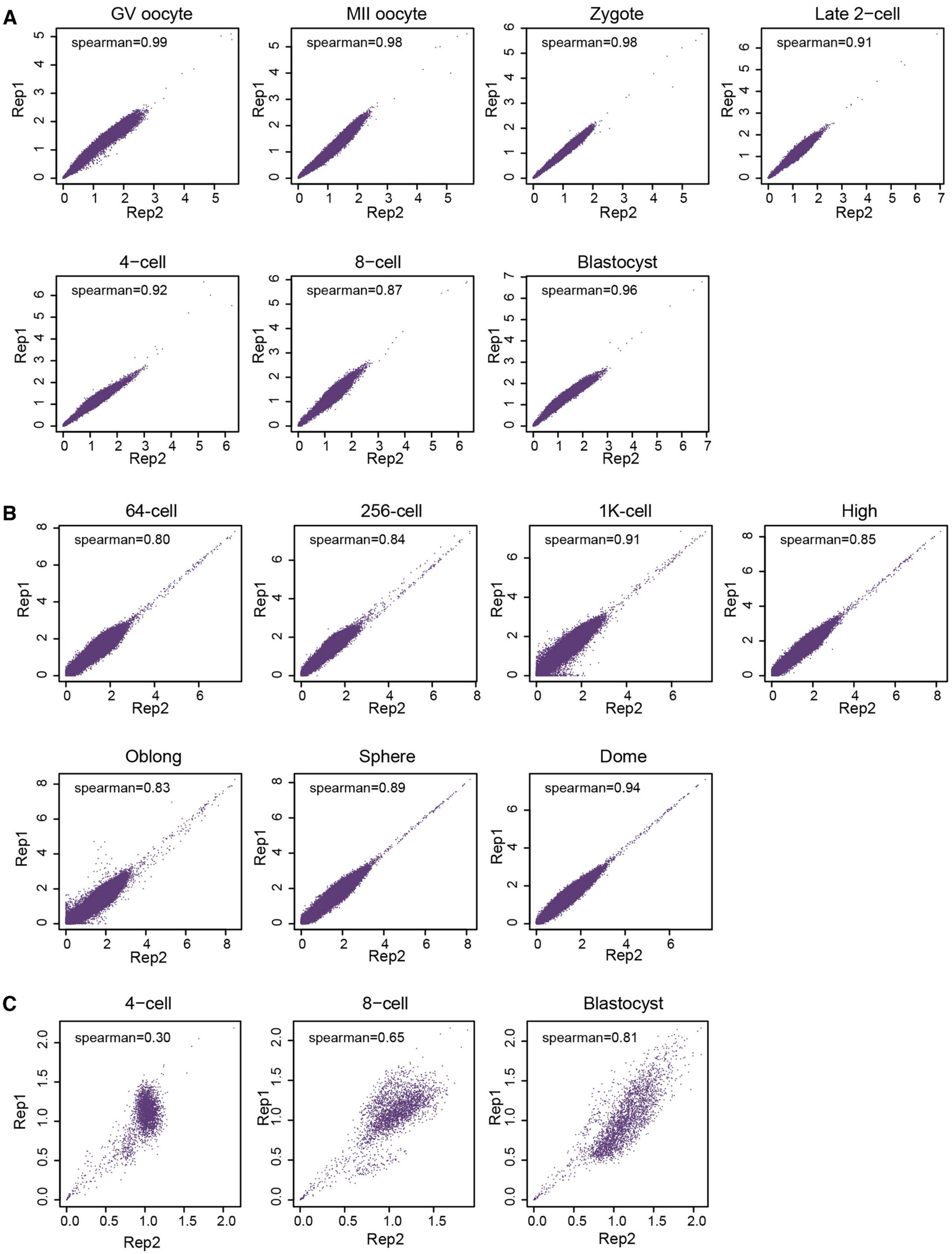
A
GV oocyte
spearman=0.99
MII oocyte
spearman=0.98
Zygote
spearman=0.98
Late 2−cell
spearman=0.91
4−cell
spearman=0.92
8−cell
spearman=0.87
Blastocyst
spearman=0.96
B
64-cell
spearman=0.80
256-cell
spearman=0.84
1K-cell
spearman=0.91
High
spearman=0.85
Oblong
spearman=0.83
Sphere
spearman=0.89
Dome
spearman=0.94
C
4−cell
spearman=0.30
8−cell
spearman=0.65
Blastocyst
spearman=0.81
Rep1
Rep2

 © The Author(s)

◀ **Figure EV2. Correlations between biological replicates of CUT&RUN.**

(**A**) The strong correlations between biological replicates at each stage from GV oocyte to blastocyst in mice demonstrate the high quality of the CUT&RUN data. (**B**) Spearman correlation analysis of CUT&RUN data across zebrafish embryonic stages, demonstrating the high reproducibility between biological replicates. (**C**) Spearman correlations between biological replicates are shown for the 4-cell, 8-cell, and blastocyst stages in human. The lower correlations observed at the earlier stages (particularly the 4-cell stage) are likely attributable to the extremely limited sample availability and the resulting technical variability, including differences in sequencing depth between these rare specimens. The axes in the plots represent log2(normalized signal + 1) values.

© The Author(s)

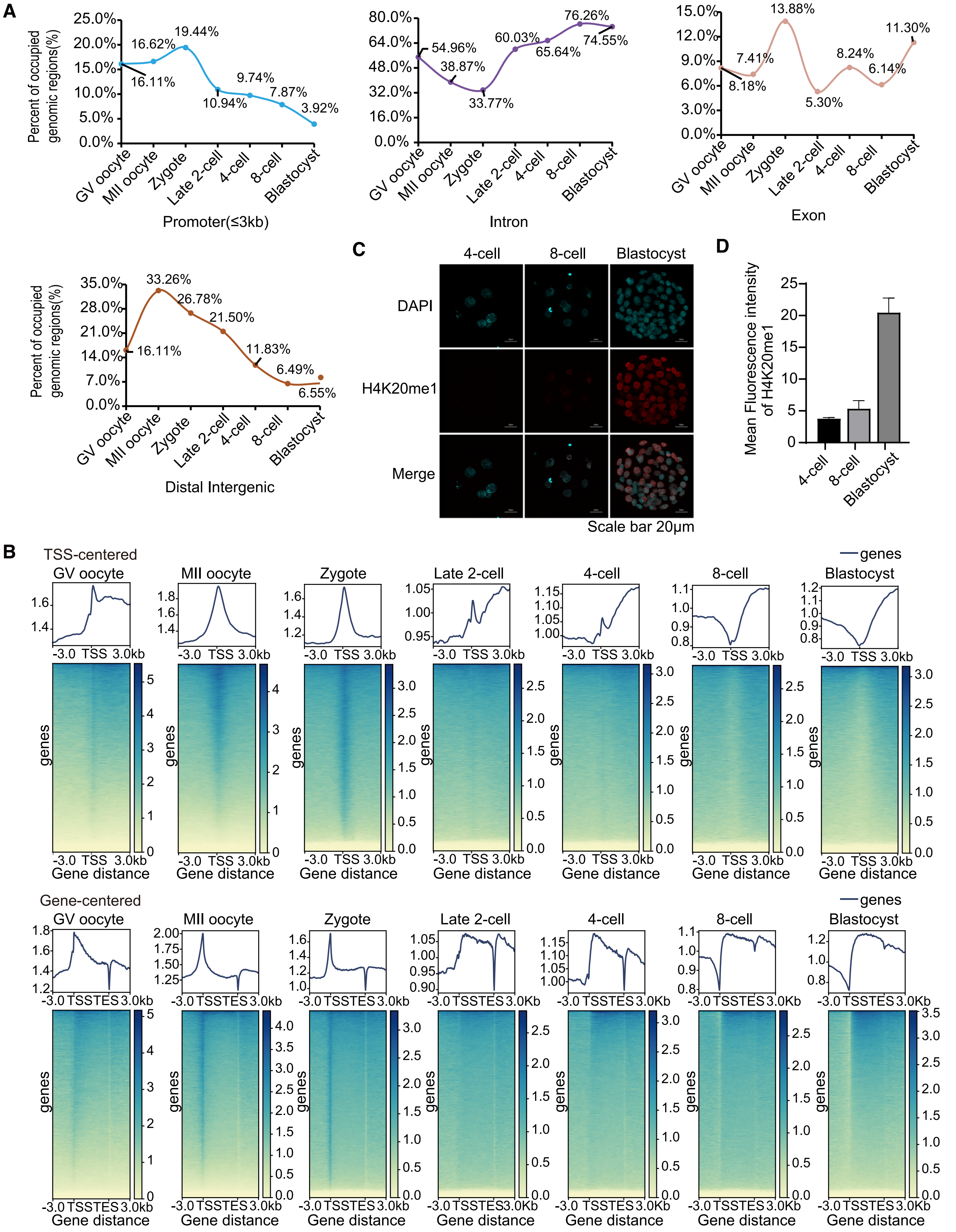
A
Percent of occupied genomic regions(%)
25.0%
20.0%
15.0%
10.0%
5.0%
0.0%
16.62%
19.44%
16.11%
9.74%
10.94%
7.87%
3.92%
GV oocyte
MII oocyte
Zygote
Late 2-cell
4-cell
8-cell
Blastocyst
Promoter(≤3kb)
80.0%
64.0%
48.0%
32.0%
16.0%
0.0%
54.96%
38.87%
33.77%
60.03%
65.64%
76.26%
74.55%
Intron
15.0%
12.0%
9.0%
6.0%
3.0%
0.0%
13.88%
7.41%
8.18%
5.30%
8.24%
6.14%
11.30%
Exon
35.0%
28.0%
21.0%
14.0%
7.0%
0.0%
33.26%
26.78%
21.50%
16.11%
11.83%
6.49%
6.55%
Distal Intergenic
C
4-cell
8-cell
Blastocyst
DAPI
H4K20me1
Merge
Scale bar 20μm
D
Mean Fluorescence intensity of H4K20me1
25
20
15
10
5
0
4-cell
8-cell
Blastocyst
B
TSS-centered
genes
GV oocyte
MII oocyte
Zygote
Late 2-cell
4-cell
8-cell
Blastocyst
-3.0 TSS 3.0kb
genes
Gene distance
Gene-centered
-3.0 TSS TES 3.0kb
-3.0 TSS TES 3.0Kb

 © The Author(s)

Figure EV3. The dynamics of H4K20me1 in mice and humans.

(**A**) Percentage of H4K20me1 peaks assigned to promoters, introns, exons, and intergenic regions at different developmental stages of the mouse. (**B**) Heatmap analysis showing TSS-centered and gene-centered distribution of H4K20me1 CUT&RUN signals in mouse oocytes and early embryos. The *y* axis represents the average H4K20me1 signal density across all genes. (**C**) Immunofluorescence images showing H4K20me1 (red), DAPI-stained nuclei (cyan), and the merged signals in human embryos. Scale bar: 20 µm. (**D**) Mean fluorescence values of H4K20me1 in human embryos. The total number of embryos analyzed per developmental stage was as follows: 4-cell (4), 8-cell (4), and blastocyst (7). Data are presented as mean ± SEM. Source data are available online for this figure.

© The Author(s)

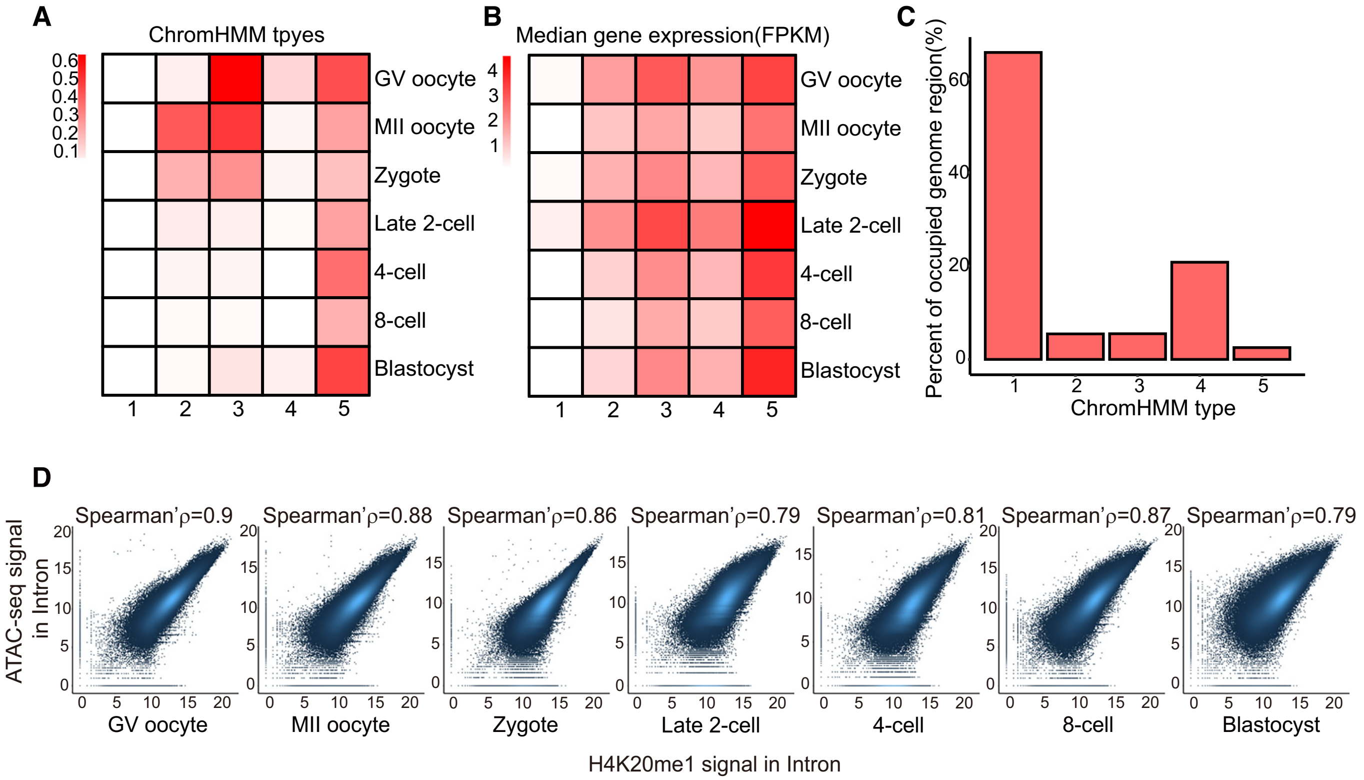

Figure EV4. Reprogramming of H4K20me1 in mouse oocytes and early embryos.

(**A**) Heatmaps of H4K20me1 dynamics for each ChromHMM type in oocytes and early embryos. Red color intensity reflects the level of H4K20me1 signal for each state at respective developmental stages. (**B**) Heatmap of the dynamics of each H4K20me1 ChromHMM type gene at different developmental stages. The intensity in red in each cell indicates the median number of fragments per kb per million reads (FPKM). (**C**) Genomic coverage proportions for each ChromHMM state. (**D**) Scatter plots comparing H4K20me1 signals with ATAC-seq signals in introns across developmental stages. Spearman correlation coefficients are shown. All *P* values < 2.2e–16.

© The Author(s)

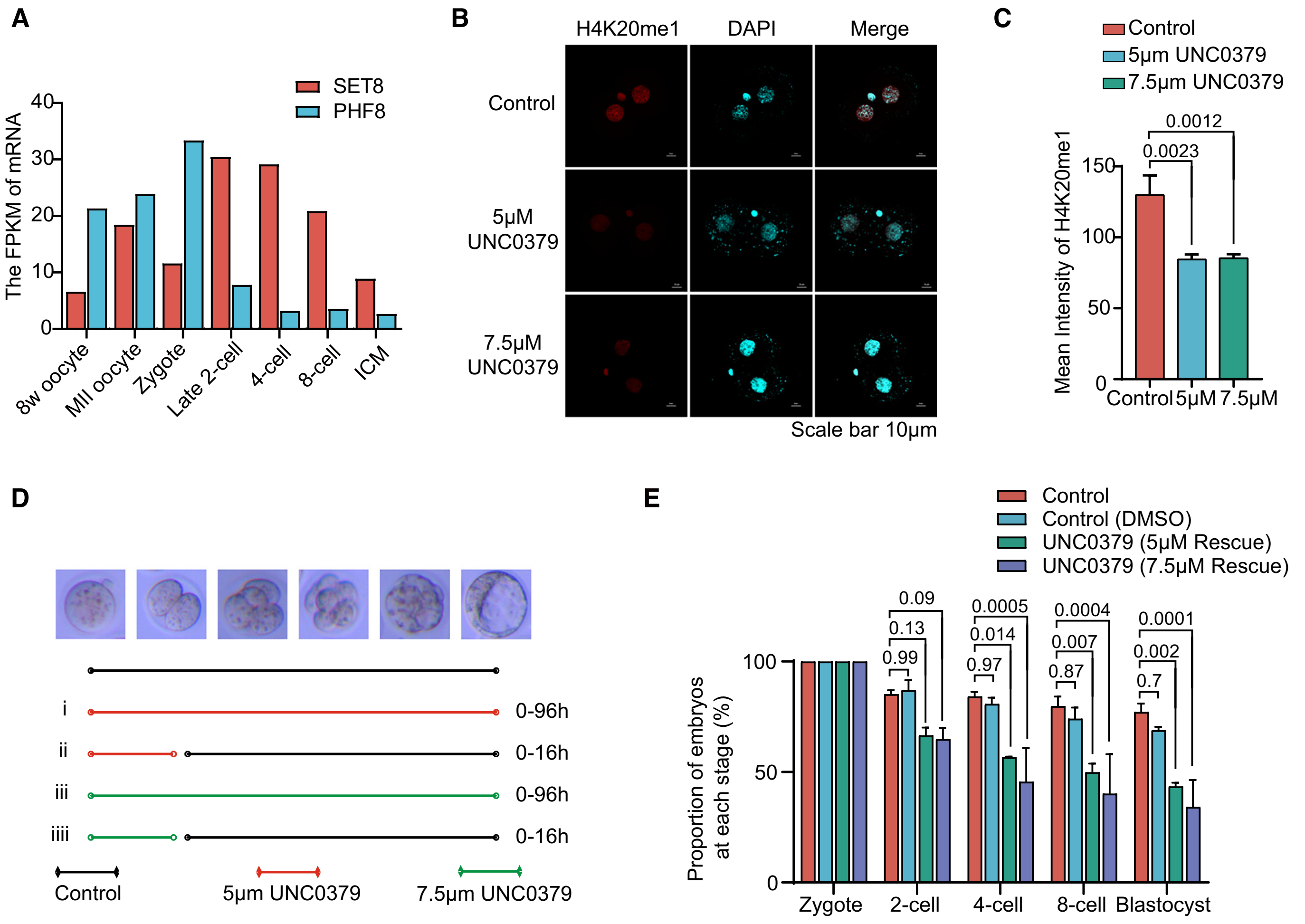

Figure EV5. Inhibition of H4K20me1 in early embryos leads to arrest of embryonic developmental processes.

(**A**) Expression of the H4K20me1-specific methylase SET8 as well as the demethylase PHF8 in mouse oocytes and early embryos. RNA-seq data from GSE71434. (**B**) Representative immunofluorescence images of 2-cell embryos under different conditions (Control, 5 μM UNC0379, 7.5 μM UNC0379). H4K20me1 (red) and DNA stained with DAPI (cyan) are shown individually and in a merge. Scale bar: 10 μm. (**C**) Mean fluorescence values of H4K20me1 levels in 2-cell embryos under different conditions ($n = 7$ embryos per group). Statistical analysis was performed using the Mann–Whitney U test. (**D**) Overview of in vitro experimental design. (**E**) The proportions of embryos at each stage in control, DMSO control, 5 μM UNC0379 rescue, and 7.5 μM UNC0379 rescue groups. Embryos were cultured in vitro and assessed at zygote (0 h), 2-cell (16 h), 4-cell (40 h), 8-cell (48 h), and blastocyst (72–96 h). The experiment was independently repeated three times. Total embryos per group: Control, $n = 89$; DMSO, $n = 72$; 5 μM UNC0379 rescue, $n = 91$; 7.5 μM UNC0379 rescue, $n = 85$. Data are shown as mean ± SEM. Statistical analysis was performed using two-way ANOVA with a multiple comparisons test. Source data are available online for this figure.

© The Author(s)

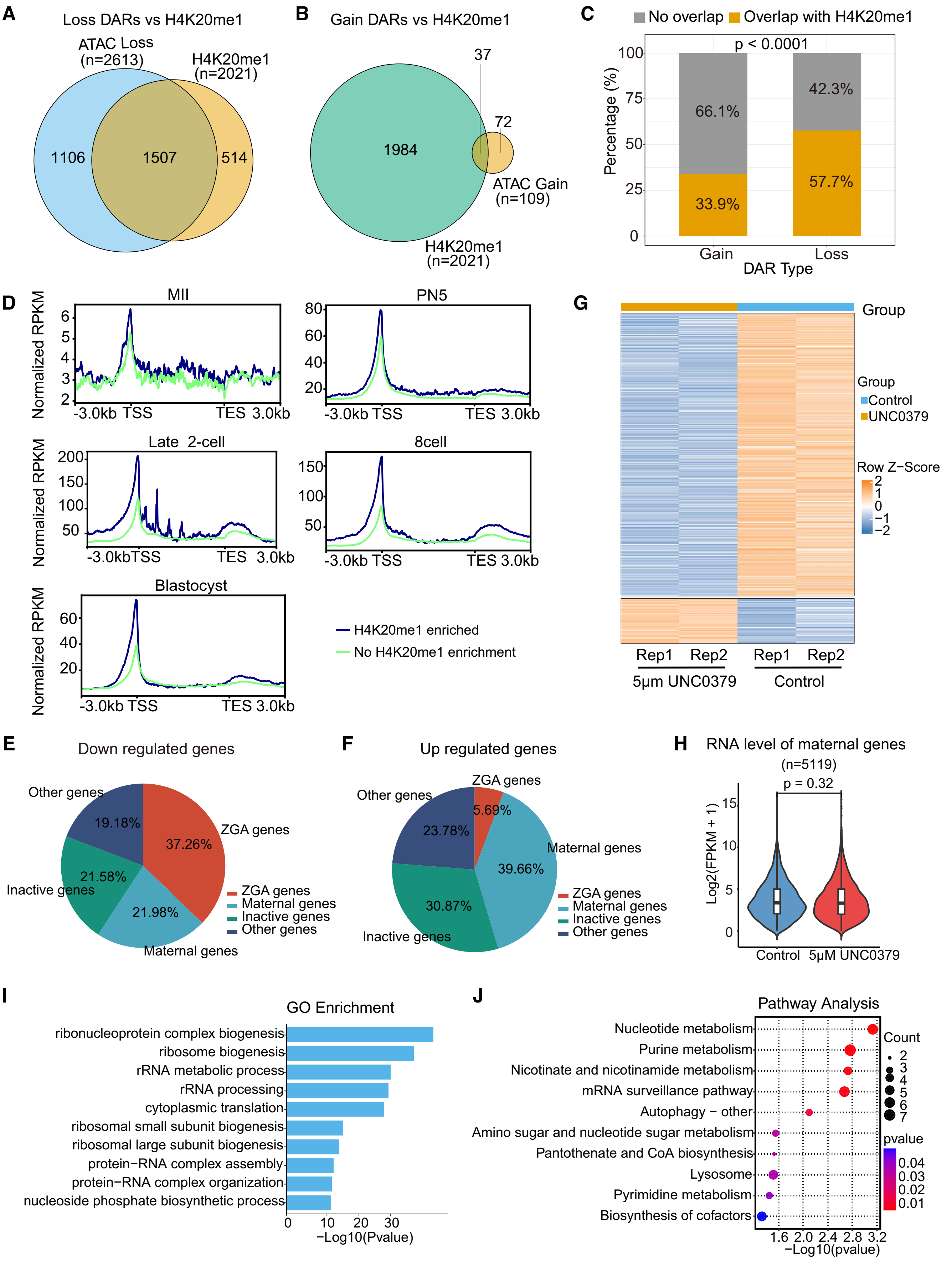
A
Loss DARs vs H4K20me1
ATAC Loss (n=2613)
H4K20me1 (n=2021)
1106
1507
514
B
Gain DARs vs H4K20me1
37
72
1984
ATAC Gain (n=109)
H4K20me1 (n=2021)
C
No overlap
Overlap with H4K20me1
p < 0.0001
Percentage (%)
66.1%
33.9%
42.3%
57.7%
Gain
Loss
DAR Type
D
MII
PN5
Late 2-cell
8cell
Blastocyst
Normalized RPKM
-3.0kb TSS
TES 3.0kb
H4K20me1 enriched
No H4K20me1 enrichment
G
Group
Control
UNC0379
Row Z−Score
Rep1 Rep2
Rep1 Rep2
5μm UNC0379
Control
E
Down regulated genes
Other genes
ZGA genes
Inactive genes
Maternal genes
19.18%
37.26%
21.58%
21.98%
ZGA genes
Maternal genes
Inactive genes
Other genes
F
Up regulated genes
ZGA genes
Other genes
Maternal genes
Inactive genes
5.69%
23.78%
39.66%
30.87%
ZGA genes
Maternal genes
Inactive genes
Other genes
H
RNA level of maternal genes
(n=5119)
p = 0.32
Log2(FPKM + 1)
Control
5μM UNC0379
I
GO Enrichment
ribonucleoprotein complex biogenesis
ribosome biogenesis
rRNA metabolic process
rRNA processing
cytoplasmic translation
ribosomal small subunit biogenesis
ribosomal large subunit biogenesis
protein−RNA complex assembly
protein−RNA complex organization
nucleoside phosphate biosynthetic process
−Log10(Pvalue)
J
Pathway Analysis
Nucleotide metabolism
Purine metabolism
Nicotinate and nicotinamide metabolism
mRNA surveillance pathway
Autophagy − other
Amino sugar and nucleotide sugar metabolism
Pantothenate and CoA biosynthesis
Lysosome
Pyrimidine metabolism
Biosynthesis of cofactors
Count
pvalue
−Log10(pvalue)

 © The Author(s)

Figure EV6. Loss of H4K20me1 in early embryos affects the expression of ZGA genes.

(**A**, **B**) Venn diagrams showing the overlap between H4K20me1 peaks in control 2-cell embryos and DARs with (**A**) lost accessibility or (**B**) gained accessibility upon H4K20me1 inhibition. Numbers indicate the counts of peaks or regions in each category and their intersection. (**C**) Stacked bar plots showing the proportion of Gain DARs (left) and Loss DARs (right) that overlap or do not overlap with H4K20me1 peaks. (**D**) Metaplot shows that Pol II is highly loaded at the locus where H4K20me1 is highly enriched. The data used to assess Pol II loading were obtained from the publicly available dataset GSE135457. The *y* axis represents the average signal density. (**E**, **F**) Pie charts showing the percentage composition of downregulated (**E**) and upregulated (**F**) genes, categorized as ZGA genes, maternal genes, inactive genes, and other gene types. (**G**) Heatmap showing expression levels of differentially expressed ZGA genes ($n = 568$) in control and UNC0379-treated samples. Signal intensity is Z-score normalized, with rows representing individual genes and columns representing samples (DESeq2, FDR < 0.05, FC > 2). (**H**) Violin plot showing no significant change in maternal gene expression between the control and 5 μM UNC0379 group. *n* denotes the number of genes included in each group. Box plots indicate the median (center line), 25th and 75th percentiles (box bounds), and whiskers extending to the minimum and maximum values. Statistical significance was determined using the Wilcoxon rank-sum test. (**I**) GO terms of ZGA genes in downregulated genes. (**J**) Kyoto Encyclopedia of Genes and Genomes (KEGG) analysis for downregulated genes. Source data are available online for this figure.
