# Supplementary material for: The reprogramming and function of H4K20me1 during early embryo development
Source: EMBO Rep. 2026 Apr 22;27(11):3050–77. doi: 10.1038/s44319-026-00780-x (PMC13261155; doi:10.1038/s44319-026-00780-x)
Supplement: Supplementary file 3 — Peer Review File [file 44319_2026_780_MOESM3_ESM.pdf]

EMBO reports

The reprogramming and function of H4K20me1 during early embryo development

Xiangrui Meng, Zhen He, Dong Fang, Wenbo Li, Jia Guo, Jiacheng Lu, Fen Yan, Xin Dong, Lingling Chen, Sarmir khan, Chunling Wang, and Jiawei Xu

Corresponding author(s): Jiawei Xu (fccxujw@zzu.edu.cn) , Zhen He (hezhen@hnsyxkxy.cn)

Review Timeline:

Submission Date:	10th Oct 25
Editorial Decision:	19th Nov 25
Revision Received:	6th Feb 26
Editorial Decision:	13th Mar 26
Revision Received:	29th Mar 26
Accepted:	2nd Apr 26

Editor: Esther Schnapp

Transaction Report:

(Note: With the exception of the correction of typographical or spelling errors that could be a source of ambiguity, letters and reports are not edited. Depending on transfer agreements, referee reports obtained elsewhere may or may not be included in this compilation. Referee reports are anonymous unless the Referee chooses to sign their reports.)

1st Editorial Decision 19th Nov 2025

Dear Prof. Xu,

Thank you for the submission of your manuscript to EMBO reports. We have now received the comments from 2 referees and given that referee 3 will not be able to send the report in the next 10 days, I decided to make a decision now in order to save you from unnecessary loss of time. We had also received comments from an advisor that I also paste below and that should be taken into account as well for the revisions.

As you will see from their reports, the referees acknowledge that the findings are potentially interesting. However, they also have a number of suggestions for how the study should be improved, and most are related to technical issues and data presentation and interpretation. I think all suggestions are good and should be addressed, but please let me know in case you disagree and we can discuss the exact revision requirements further, also in a video chat, if you like.

I would thus like to invite you to revise your manuscript with the understanding that the referee concerns must be fully addressed and their suggestions taken on board. Please address all referee concerns in a complete point-by-point response. Acceptance of the manuscript will depend on a positive outcome of a second round of review. It is EMBO reports policy to allow a single round of major revision only and acceptance or rejection of the manuscript will therefore depend on the completeness of your responses included in the next, final version of the manuscript.

We realize that it is difficult to revise to a specific deadline. In the interest of protecting the conceptual advance provided by the work, we recommend a revision within 3 months (19th Feb 2026). Please discuss the revision progress ahead of this time with the editor if you require more time to complete the revisions.

IMPORTANT NOTE: we perform an initial quality control of all revised manuscripts before re-review. Your manuscript will FAIL this control and the handling will be DELAYED if the following APPLIES:
1) A data availability section providing access to data deposited in public databases is missing. If you have not deposited any data, please add a sentence to the data availability section that explains that.
2) Your manuscript contains statistics and error bars based on n=2. Please use scatter blots in these cases. No statistics should be calculated if n=2.

When submitting your revised manuscript, please carefully review the instructions that follow below. Failure to include requested items will delay the evaluation of your revision.

1) a .docx formatted version of the manuscript text (including legends for main figures, EV figures and tables). Please make sure that the changes are highlighted to be clearly visible.

2) individual production quality figure files as .eps, .tif, .jpg (one file per figure). See https://wol-prod-cdn.literatumonline.com/pb-assets/embo-site/EMBOPress_Figure_Guidelines_061115-1561436025777.pdf for more info on how to prepare your figures.

3) We replaced Supplementary Information with Expanded View (EV) Figures and Tables that are collapsible/expandable online. A maximum of 5 EV Figures can be typeset. EV Figures should be cited as 'Figure EV1, Figure EV2" etc... in the text and their respective legends should be included in the main text after the legends of regular figures.

- For the figures that you do NOT wish to display as Expanded View figures, they should be bundled together with their legends in a single PDF file called *Appendix*, which should start with a short Table of Content. Appendix figures should be referred to in the main text as: "Appendix Figure S1, Appendix Figure S2" etc. See detailed instructions regarding expanded view here: <https://www.embopress.org/page/journal/14693178/authorguide#expandedview>

- Additional Tables/Datasets should be labeled and referred to as Table EV1, Dataset EV1, etc. Legends have to be provided in a separate tab in case of .xls files. Alternatively, the legend can be supplied as a separate text file (README) and zipped together with the Table/Dataset file.

4) a .docx formatted letter INCLUDING the reviewers' reports and your detailed point-by-point responses to their comments. As part of the EMBO Press transparent editorial process, the point-by-point response is part of the Review Process File (RPF), which will be published alongside your paper.

5) a complete author checklist, which you can download from our author guidelines <https://www.embopress.org/page/journal/14693178/authorguide>. Please insert information in the checklist that is also reflected in the manuscript. The completed author checklist will also be part of the RPF.

6) Please note that all corresponding authors are required to supply an ORCID ID for their name upon submission of a revised manuscript (<https://orcid.org/>). Please find instructions on how to link your ORCID ID to your account in our manuscript tracking system in our Author guidelines

<https://www.embopress.org/page/journal/14693178/authorguide#authorshipguidelines>

7) Before submitting your revision, primary datasets produced in this study need to be deposited in an appropriate public database (see https://www.embopress.org/page/journal/14693178/authorguide#datadeposition). Please remember to provide a reviewer password if the datasets are not yet public. The accession numbers and database should be listed in a formal "Data Availability" section placed after Materials & Method (see also https://www.embopress.org/page/journal/14693178/authorguide#datadeposition). Please note that the Data Availability Section is restricted to new primary data that are part of this study. * Note - All links should resolve to a page where the data can be accessed. *
If your study has not produced novel datasets, please mention this fact in the Data Availability Section.

8) At EMBO Press we ask authors to provide source data for the main manuscript figures. You will receive a separate email with instructions for providing source data with your revised manuscript, including how to upload and organize the files.

9) Our journal also encourages inclusion of *data citations in the reference list* to directly cite datasets that were re-used and obtained from public databases. Data citations in the article text are distinct from normal bibliographical citations and should directly link to the database records from which the data can be accessed. In the main text, data citations are formatted as follows: "Data ref: Smith et al, 2001" or "Data ref: NCBI Sequence Read Archive PRJNA342805, 2017". In the Reference list, data citations must be labeled with "[DATASET]". A data reference must provide the database name, accession number/identifiers and a resolvable link to the landing page from which the data can be accessed at the end of the reference. Further instructions are available at https://www.embopress.org/page/journal/14693178/authorguide#referencesformat

10) Regarding data quantification (see Figure Legends: https://www.embopress.org/page/journal/14693178/authorguide#figureformat)

The following points must be specified in each figure legend:

- the name of the statistical test used to generate error bars and P values,

- the number (n) of independent experiments (please specify technical or biological replicates) underlying each data point,

- the nature of the bars and error bars (s.d., s.e.m.),

- If the data are obtained from n Program fragment delivered error ``Can't locate object method "less" via package "than" (perhaps you forgot to load "than"?) at //ejpvfs23/sites23b/embor_www/letters/embor_decision_revise_and_review.txt line 56.' 2, use scatter blots showing the individual data points.

Discussion of statistical methodology can be reported in the materials and methods section, but figure legends should contain a basic description of n, P and the test applied.

- Please also include scale bars in all microscopy images.

11) The journal requires a statement specifying whether or not authors have competing interests (defined as all potential or actual interests that could be perceived to influence the presentation or interpretation of an article). In case of competing interests, this must be specified in your disclosure statement. Further information: https://www.embopress.org/competing-interests

12) All Materials and Methods need to be described in the main text using our 'Structured Methods' format, which is required for all research articles. According to this format, the Methods section includes a separate Reagents and Tools Table file (listing key reagents, experimental models, software and relevant equipment and including their sources and relevant identifiers) and a Methods and Protocols section describing the methods using a step-by-step protocol format. The aim is to facilitate adoption of the methodologies across labs. More information on how to adhere to this format as well as a downloadable template (.docx) for the Reagents and Tools Table can be found in our author guidelines: https://www.embopress.org/page/journal/14693178/authorguide#structuredmethods.

An example of a Method paper with Structured Methods can be found here: https://www.embopress.org/doi/full/10.1038/s44320-024-00037-6#sec-4

We would also welcome the submission of cover suggestions, or motifs to be used by our Graphics Illustrator in designing a cover.

As part of the EMBO publication's Transparent Editorial Process, EMBO reports publishes online a Review Process File (RPF) to accompany accepted manuscripts. This File will be published in conjunction with your paper and will include the referee reports, your point-by-point response and all pertinent correspondence relating to the manuscript.

You are able to opt out of this by letting the editorial office know (emboreports@embo.org). If you do opt out, the Review Process File link will point to the following statement: "No Review Process File is available with this article, as the authors have chosen not to make the review process public in this case."

I look forward to seeing a revised form of your manuscript when it is ready.

Best regards,
Esther

Esther Schnapp, PhD
Senior Editor
EMBO reports

Referee #1:

Meng et al investigate the role of H4K20me1 in early development, focusing on mouse oocytes and embryos. They find broad domains of H4K20me1 predominantly in gene loci, with evidence for wiping and reestablishing this mark during the maternal-to-zygotic transition. Using a pharmacological inhibitor, they show that inhibiting H4K20me1 inhibits development and is correlated with a loss of chromatin accessibility and disruption of zygotic genome activation. Overall, the genomics data are valuable contributions to the field, even though the biological interpretation of how they relate to ZGA remains to be further refined.

Points to address:

1. The H4K20me1 CUT&RUN could be more effectively visualized genome-wide using heatmaps in the style of Fig 4B. It is difficult to see the patterns suggested in Fig 1D on the browser track in Fig 1A. For example, in 1D zygotes have a peak of coverage at the TSS, while for blastocysts there is low coverage at the TSS but higher in the gene body. But the browser tracks in 1A look more like loss of intergenic signal from zygote to blastocyst, rather than a shift from TSS-enrichment to gene body enrichment. Heatmaps over all genes across time would help interpret the metaplots in 1D.

2. But related, it seems like comparing H4K20me1 CUT&RUN levels between stages is challenging because of the lack of a spike-in or other standard. So, in this sense the plot in 1D could be misleading regarding the magnitude of the TSS peak in zygotes. I am not suggesting repeating these experiments, but I do think the analysis and visualizations should carefully take this into account. One suggestion could be to use the fluorescence intensity (Fig 5B) as a stage-specific scaling factor; or to use a computational method to infer scaling factors such as ChIPseqSpikeInFree.

3. The authors use the term "non-canonical pattern" to refer to H4K20me1 genomic distribution in oocytes and embryos, but it's unclear what this is in reference to (i.e., what would canonical look like?). It's particularly problematic to make a claim like (p. 7) "These data indicate that the non-canonical distribution pattern of H4K20me1 in early development is evolutionarily conserved across vertebrates" -- is this meant as, the pattern observed is the same across the three species? Or is it meant as, the pattern is "non canonical" in each of these three species, not necessarily in the same way, but the property of being "non canonical" is shared across the three species? I would argue that neither of these is appropriate. The authors should more precisely describe what the common (or divergent?) pattern of H4K20me1 is between the three species, and also avoid making generalizations about vertebrate-wide conservation based on only three species.

4. The distinction between Promoter and Gene body H4K20me1 coverage (e.g., Fig 3) is potentially imprecise, since there can be continuous signal that overlaps both. This becomes very apparent looking at the ATAC-seq signal in Fig 4B, which shows that the highest concentration of ATAC-seq coverage is at the TSS for both the promoter category and the gene body category (not surprisingly). Are many of the same gene loci represented in both categories? Perhaps the authors could more precisely define these two gene groups for the various analyses (Fig 3, 4) so that they more strongly reflect functionally distinct sets of genes (e.g., poised versus actively transcribed).

It also seems like H4K20me1 profiles would be changing from promoter-centered to gene-body for many of the genes, so unifying the analyses in Fig 3 would be beneficial, and showing heatmaps over genes divided into the different categories of change would be very informative.

5. The micrographs in Fig 5 (both fluorescence and brightfield) are very hard to see. For the fluorescence images, false coloring (e.g., cyan instead of blue) or using inverted grayscale would help. For the brightfield (Fig 5D, I), the figures need to be much larger for the reader to be able to assess the stages/morphologies.

6. Regarding the effect of H4K20me1 on ZGA raises, the authors don't seem to address that the majority of gene expression defects on H4K20me1 inhibition are for genes unrelated to ZGA. Additional analysis is warranted here -- is H4K20me1 inhibition

leading to ectopic expression of some genes? If so, when during development would these genes normally be expressed? And there are 1019 genes not related to ZGA that are downregulated -- what are these? One might suggest they are maternal mRNA that are somehow destabilized, but the graphs in Fig 7B are very confusing in this regard (indeed, please clarify all of Fig 7B). Ultimately, by definition, every mRNA detected in the embryo at these stages must be either maternal or zygotic expressed during minor or major ZGA, so the authors need to clarify what these other genes are. Also, Fig S4 title says "Loss of H4K20me1 in early embryos mainly affects major ZGA" which seems incorrect in light of this.

Additional points:

7. Many of the figures are low-resolution with small text that is difficult to read.
8. Most of the legends are very sparsely written, and not all the figure labeling is intuitive enough to understand. Please ensure that sufficient information is included for each figure panel. Some examples I noticed: what is the significance of the purple highlighting in Fig 1A? Is Fig 1D, 6F, and 7C, 7D showing *average* density across all genes, or sum? Is Fig3E,G *log2* normalized CPM? What does "development rate" mean in Fig 5E (is this proportion of embryos that attain each stage? That is not what I would interpret to mean development rate)?
9. Why is the GO enrichment analysis in Fig 6E showing enrichment score but in Fig 3F,H it is -log10 P? (Also, is this multiple test corrected P?)
10. Fig 6D and E use a non-accessible red-green color scheme, which should be switched to something that is discernable to red-green colorblind readers

Referee #2:

In this manuscript, Meng and colleagues profile the genome-wide distribution of H4K20me1 in mouse, human and zebrafish early embryos. They indicate the presence of a non-canonical distribution of H4K20me1 in all three species, which is reminiscent of other histone modifications at this stage of development. However, they also present species-specific differences in the timing and distribution of the modification across early embryonic development. The majority of the downstream analysis is performed on the mouse data, showing reprogramming of promoter and gene body H4K20me1 and correlation to chromatin accessibility data. Functional experiments in mouse embryos, using an inhibitor of Set8, the only H4K20me1 methyltransferase, show a developmental arrest at the 2-cell stage, with a proportion of major ZGA genes downregulated associated with changes in chromatin accessibility. While Set8 has previously been described to be essential for early embryonic development in mouse, through knockout experiments, H4K20M mutant expression and use of the same inhibitor as in this manuscript (referenced papers: Oda et al., 2009, Shikata et al., 2020), the analysis of transcriptome and chromatin accessibility changes upon perturbation are novel.
Thus, the dataset presented here along with functional experiments have the potential to provide functional insight with physiological relevance that will have an impact on both developmental biology and the chromatin/epigenetics communities. However, I have several concerns over the experimental design, presentation and interpretation of the results that need to be addressed before publication. In particular, I find a number of conclusions not supported by the data and inconsistencies between figures, as detailed below. This could be partly related to the quality of the English language, which hinders the communication of the results and conclusions. In addition, the Figures and legends often lack enough detail, such as the scale, to understand what is shown.
Specific comments
Major
1. Inhibition of Set8 leads to changes in H4K20me2/3 levels in mouse preimplantation embryos (Oda et al, 2009) as H4K20me1 is a substrate for the generation of H4K20me2/3. Therefore, the authors cannot rule out the phenotypes observed are due to altered levels of H4K20me2/3. This should be made clear in the manuscript.
2. It is not clear how the non-canonical vs canonical distribution of H4K20me1 is defined and when it is observed in all three species? Is it defined by the size of the domains or the genomic distribution? Which stages are consistent in terms of domain size or genomic distribution of H4K20me1 with what has been reported in cell lines (of the appropriate matching species), for example, and which are not? How is the non-canonical distribution linked to ZGA as they claim? For example, the clustering analysis in Figure 3 does not demonstrate a non-canonical distribution, just that the localisation of the mark is different before 2-cell to after 2-cell stage.
3. The authors conclude that the H4K20me1 signal 'disappears' (page 5) or is 'rapidly lost' (page 6) at the late 2-cell stage. However, there is still clearly enrichment in the example snapshot in Figure 1A at the late 2-cell stage. They also claim that H4K20me1 'subsequently re-establishes itself, gradually stabilizing during the 4-cell stage'. This conclusion is not supported by the data presented in Figure 1B, in which the number of detected peaks at the 4-cell stage are still very low compared to the oocyte and then decrease even further at the 8-cell stage. Overall, these conclusions are also inconsistent with their immunofluorescence data in Figure 5A and in the referenced paper Shikata et al, which do not show any reduction in H4K20me1 signal at the 2-cell stage. The loss of H4K20me1 signal that they claim at the late 2-cell stage is also not consistent with their conclusion that H4K20me1 plays a critical role in major ZGA. Is the reduction in H4K20me1 peaks that they observed in the genomics data due to either data quality at the 2-cell stage or an artifact of the peak calling strategy? Information about the sequencing data quality should be provided, such as number of reads and mapping statistics should be shown. How was the

peak calling algorithm optimised? Data of % genome covered by peaks and % signal in peaks across stages should be shown.
5. The validation of the data presented in Figure S1 is not clearly presented. It is not clear what is shown in Figures S1A and D as there is no axes labels. In addition, it seems a log scale would be more appropriate here as most of the data points are overlapping. The spearman correlation test should be used rather than the pearson, unless they show that the data is normally distributed. It is not clear what is shown in Figure S1B: correlation between biological replicates of the same sample (e.g. 1,000 cells vs 1,000 cells) or correlation of each sample to bulk (e.g. 100, 200, 500 cells to bulk) or 100 cells vs other samples (100 vs 100, 200, 500 etc)? Please include validation of human and zebrafish H4K20me1 Cut&Run data by correlation of replicates, at least.
6. Immunofluorescence.
The immunofluorescence images including the DAPI channel are very weak throughout. Please improve the images, by presenting them in greyscale and increasing the brightness/contrast (consistently across each panel), if necessary. How was quantification of fluorescence intensity performed? It is not sufficient to write 'using ImageJ'. How was the region of interest selected? Was the mean fluorescence intensity calculated in a single z slice or across multiple slices? Please include the information on replicates (number of independent experiments and number of embryos) throughout.
7. Development experiments.
According to the methods the control group are embryos grown in culture medium. The experiment should be controlled by testing the solvent of the inhibitor (i.e. DMSO) diluted in culture medium to the same concentration as with the inhibitor. Please add the timing information to Figure 5E. The development rate to blastocyst in the control group is less than 40%. Typically, over 90% of control embryos reach the blastocyst in this assay. Please also add an additional later timepoint (e.g. 96h) to determine whether the embryos are arrested or delayed in development to blastocyst in the control groups. Quantification of the results of Figure 5I should also be provided.
8. Chromatin accessibility analysis.
The three datasets mentioned GSE116857, GSE136403, GSE66390 did not analyze 'the association between H4K20me1 and chromatin opening in early mouse embryos' (page 9). None of these datasets include data for H4K20me1.
The data in Figures 4B/C seem to contradict Figure 4D at both promoters and gene bodies. For example, in Figure 4B high H4K20me1 promoters display reduced chromatin accessibility at the late 2-cell stage. However, in Figure 4D all promoters seem to have both high H4K20me1 and ATAC-seq signal. This may be due to a scaling issue as the scale of the axes in Figure 4D is not described. A log scale may be more appropriate here.
The claim that 'the gene body regions of H4K20me1 were consistently and strongly positively correlated with chromatin accessibility' (page 10), which is apparent in Figure 4D, is not clear in Figure 4C. For example, in blastocyst very little ATAC-seq signal is observed in the gene body in either H4K20me1 high or low groups. The signal is almost exclusively focused at the TSS. Do they include the TSS signal as gene body? If so, the TSS should be excluded from gene body analysis.
To support the authors' interpretation that H4K20me1 regulates chromatin accessibility they should test whether DARs correlate to peaks of H4K20me1 genome-wide.
9. English language
The English should be checked and improved throughout. Here are a few examples:
• In Figures 1C, 1G and 2C legends the data does not show 'the proportion of the genome covered by H4K20me1' but rather the proportion of H4K20me1 signal in different genomic features.
• 'We found that there existed embryos that were able to develop backward across the 2-cell stage' (page 11).
• 'All three clusters exhibited erasure of MII oocyte-zygote embryos, followed by re-establishment at late 2-cell embryos, and clusters differed only in H4K20me1 levels' (page 8).
• 'the distribution of H4K20me1 shifted from the TSS to the two sides of the genome in late 2-cell embryos, resulting in a transition to a bimodal pattern' (page 6).
• 'late 2-cell stage embryos exhibited significantly reduced chromatin accessibility' (page 9-10). This analysis is not genome-wide and thus they cannot conclude that there is reduced accessibility at the late 2-cell stage in general.

Minor
The alternative names of Set8 should be described upon its first introduction (KMT5A, Pr-Set7/9)
Please include a table detailing sample and the sequencing information. For example, how many embryos were collected at each stage? How many reads/mapped reads were obtained for each sample etc.
Figure 1A What is the purple shading?
Figure S1C This is not genome-wide.
Figure 2. The time of major ZGA in zebrafish should be noted (it would help to mark it on the figures)
Figures 2B and C are swapped in the legend.
Figure 3F Cluster 1 not cluster 4 genes are associated with voltage-gated channel activity as described in the text (page 8).
Figure 5D. The magnification of the images are too low to see the phenotype of the embryos.
Figure 7C. Is the narrow heatmap to the right of the heatmaps for H4K20me1 showing RNA levels? This should be better described.
Figure 6F and Figure S4D. It is not clear which data are used to assess PolII loading.

Advisor's comments:

The datasets are new and potentially helpful, and this is where I would see the value of the work (no one has reported K20me1 genomics in embryos, which the current paper has). I have, however, two small caveats for this.

First is that the tracks look a little strange, and there is no IgG or input controls. The patterns look suspiciously similar to ATAC-seq, specially at the earliest stages and thus I am not entirely sure that the authors are indeed picking the right K20me1 signal (this could be seen at a formal reviewing step). Also, their interpretations (I believe because of the 'signal' issue) go quite a bit against what is known on K20me1, which oscillates during cell cycle and progressively accumulates during S-phase.

Second, the requirement for early development is not entirely new. The zygotic depletion of PrSet7 results in arrest between the 4-8-cell stage, which has been ascribed to defects in cell cycle progression, in particular S-phase, and signs of DNA damage (and not to transcriptional changes). The authors use inhibitors earlier and report an earlier phenotype, but considering PR-set7 is required for cell viability, it is not clear whether the interpretations of the authors on zygotic genome activation are fully explored and founded.

So, in short, the datasets are definitely potentially useful for the field, but their QC should be properly evaluated. Ideally, the authors should focus on new biology of K20me1 and not the known role of PRSet7 in early embryos, as indeed this angle undermines novelty.

Response Letter

Dear Editors and Reviewers:

We would like to thank you and the reviewers for the extremely helpful comments on our manuscript entitled "The reprogramming and function of H4K20me1 during early development" (Manuscript ID: EMBOR-2025-62939-T). We have carefully read through the highly constructive comments and made lots of revisions, with specific responses provided on the following pages. The manuscript was revised accordingly. The changes are marked in red text in the marked-up manuscript.

We appreciate the Editors' hard work and Reviewers' valuable suggestions earnestly. We believe the manuscript has been greatly improved with the new revised, and these revisions are responsive to the extremely helpful critiques of the Reviewers, and we look forward to your guidance.

Yours Sincerely,
Dr. Jiawei Xu, Dr. Zhen He.

Corresponding author:
Jiawei Xu, Ph.D.
The First Affiliated Hospital of Zhengzhou University & Institute of Reproductive Health, Henan Academy of Innovations in Medical Science; Zhengzhou, 450000, China.
Email: fccxujw@zzu.edu.cn

Zhen He, Ph.D.
Institute of Reproductive Health, Henan Academy of Innovations in Medical Science; Zhengzhou, 450000, China.

Email: hezhen2017@foxmail.com

To Referee #1:

Meng et al investigate the role of H4K20me1 in early development, focusing on mouse oocytes and embryos. They find broad domains of H4K20me1 predominantly in gene loci, with evidence for wiping and reestablishing this mark during the maternal-to-zygotic transition. Using a pharmacological inhibitor, they show that inhibiting H4K20me1 inhibits development and is correlated with a loss of chromatin accessibility and disruption of zygotic genome activation. Overall, the genomics data are valuable contributions to the field, even though the biological interpretation of how they relate to ZGA remains to be further refined.

Response:

Thank you for your valuable comments on our manuscript. We have revised the manuscript one by one according to your valuable comments. Please see below for details.

Points to address:

1. The H4K20me1 CUT&RUN could be more effectively visualized genome-wide using heatmaps in the style of Fig 4B. It is difficult to see the patterns suggested in Fig 1D on the browser track in Fig 1A. For example, in 1D zygotes have a peak of coverage at the TSS, while for blastocysts there is low coverage at the TSS but higher in the gene body. But the browser tracks in 1A look more like loss of intergenic signal from zygote to blastocyst, rather than a shift from TSS-enrichment to gene body enrichment. Heatmaps over all genes across time would help interpret the metaplots in 1D.

Response:

We thank a lot for bringing these issues to our attention. We agree that genome-wide heatmaps would provide a more comprehensive and intuitive visualization of the dynamic redistribution of H4K20me1 compared to individual browser tracks, which only depict the signal distribution at specific genomic loci. As suggested, we have generated two sets of genome-wide heatmaps: one centered around the transcription start site (TSS) and the other spanning regions from 3 kb upstream of the TSS to 3 kb downstream of the transcription end site (TES) for each developmental stage (Response Fig 1). These heatmaps illustrate the shift from a TSS-centric pattern in oocytes and zygotes to a gene body-enriched pattern in blastocysts, thereby confirming the trend indicated by the metaplots in Fig 1D. The new visualizations are presented in the Fig EV1.

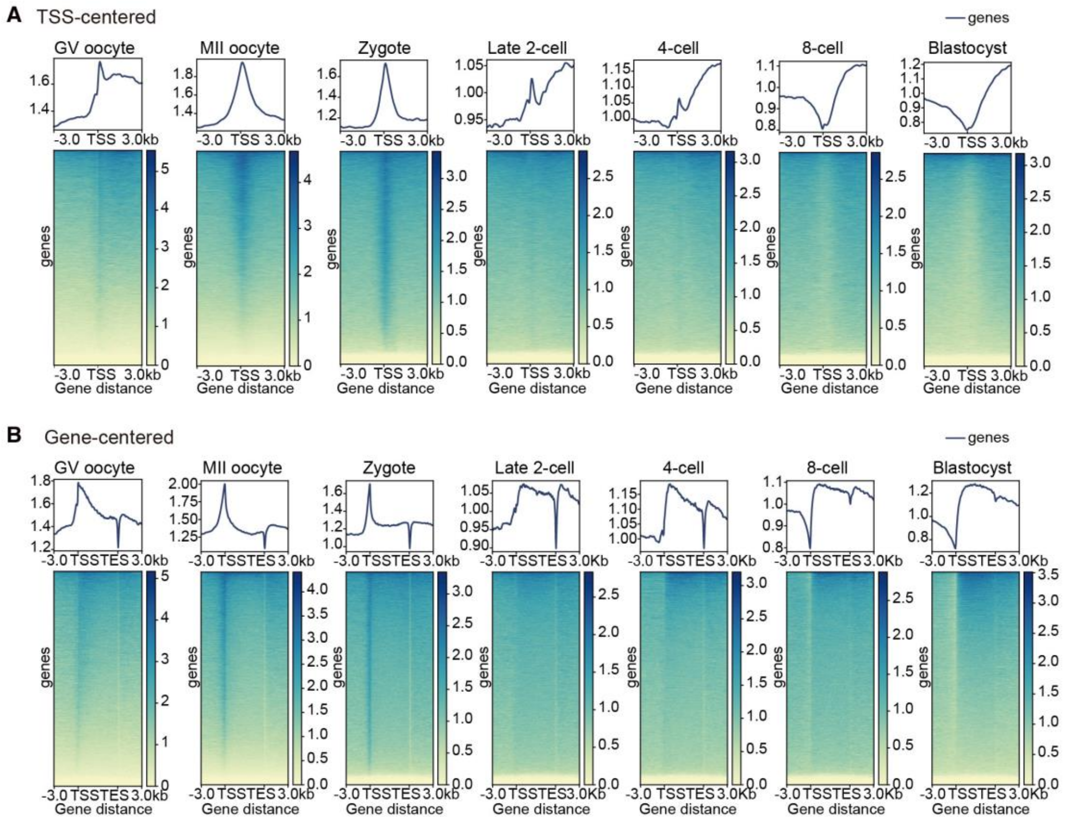

Response Fig 1. Distribution of H4K20me1 across different mouse developmental stages. Heatmaps analysis showing that TSS-centered (A) and gene-centered (B)

distribution of H4K20me1 CUT&RUN signals in mouse oocytes and early embryos. The y-axis represents the average H4K20me1 signal density across genes.

2. But related, it seems like comparing H4K20me1 CUT&RUN levels between stages is challenging because of the lack of a spike-in or other standard. So, in this sense the plot in 1D could be misleading regarding the magnitude of the TSS peak in zygotes. I am not suggesting repeating these experiments, but I do think the analysis and visualizations should carefully take this into account. One suggestion could be to use the fluorescence intensity (Fig 5B) as a stage-specific scaling factor; or to use a computational method to infer scaling factors such as ChIPseqSpikeInFree.

Response:

We appreciate and acknowledge your important point that comparing H4K20me1 signal levels across developmental stages is inherently challenging in the absence of spike-in or other global controls. In our original analysis, we normalized the CUT&RUN data using Counts Per Million mapped reads (CPM) to account for differences across samples. We confirmed the sequencing quality and validated the reliability of our CUT&RUN results. However, we fully agree that CPM normalization alone cannot correct for potential biological differences between stages, which could affect the interpretation of cross-stage signal changes.

To assess whether such global shifts substantially impact our data interpretation, we followed the constructive suggestion and applied the ChIPseqSpikeInFree method, which effectively determines scaling factors across different conditions and treatments. Our results showed that the stages with differential scaling factors were: GV oocyte (1.39), MII oocyte (1.80), and blastocyst (2.21). The rescaled data showed a high Spearman correlation (>0.9) with the original CPM-normalized signals across these stages (see Response Fig 2). This indicates that while global scaling factors exist, the

relative spatial patterns and stage-to-stage trends of H4K20me1 enrichment remain highly consistent before and after adjustment.

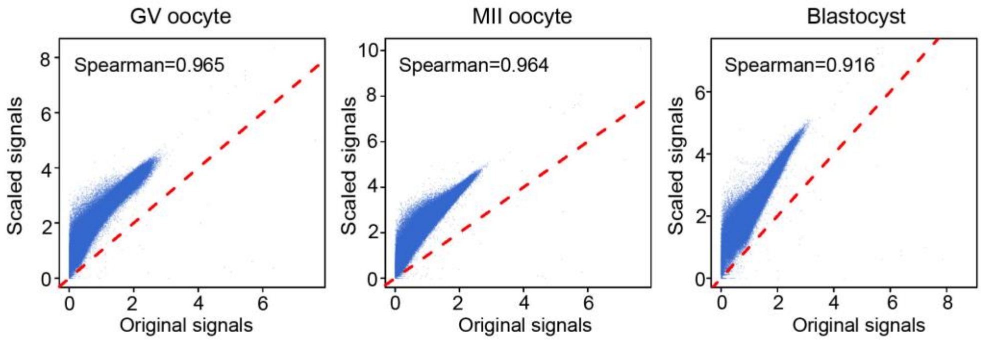

Response Fig 2. Correlation between original CPM-normalized signals and ChIPseqSpikeInFree-scaled signals. Spearman correlation analysis showing that high correlation between original CPM-normalized signals and ChIPseqSpikeInFree-scaled signals in the stages with differential scaling factors (GV oocyte, MII oocyte, and blastocyst). The axis is on a Log2 scale, and this figure was added as Appendix Fig S2C.

Therefore, while the methodological consideration regarding normalization is valid, the strong correlation between original and rescaled profiles supports the robustness of our original CPM-based analyses in capturing the biological dynamics of H4K20me1 redistribution. For consistency and to maintain a direct representation of the sequenced read distribution, the figures in the manuscript continue to present the CPM-normalized data. We have added a note in the methods (page 25) to explain this validation step and to transparently acknowledge the normalization considerations: "Furthermore, we performed an additional validation using the ChIPseqSpikeInFree method(Jin *et al*, 2020) to estimate stage-specific scaling factors. The correlation between original and rescaled signals was used to evaluate the impact of global scaling on our reported patterns. The subsequent analyses and visualizations presented in the main figures are based on the CPM-normalized data to directly reflect the

sequenced read distribution."

3. The authors use the term "non-canonical pattern" to refer to H4K20me1 genomic distribution in oocytes and embryos, but it's unclear what this is in reference to (i.e., what would canonical look like?). It's particularly problematic to make a claim like (p. 7) "These data indicate that the non-canonical distribution pattern of H4K20me1 in early development is evolutionarily conserved across vertebrates" -- is this meant as, the pattern observed is the same across the three species? Or is it meant as, the pattern is "non canonical" in each of these three species, not necessarily in the same way, but the property of being "non canonical" is shared across the three species? I would argue that neither of these is appropriate. The authors should more precisely describe what the common (or divergent?) pattern of H4K20me1 is between the three species, and also avoid making generalizations about vertebrate-wide conservation based on only three species.

Response:

Thank you for raising these crucial points regarding our use of the term "non-canonical pattern" and the suggestion of evolutionary conservation. Your argument and suggestion made us have a more accurate understanding about "non-canonical pattern". We agree that our initial phrasing lacked precision and that the broad claim of vertebrate-wide conservation was not sufficiently supported.

First, regarding the definition of "canonical", we initially use it to refer to the well-established, sharp, and localized enrichment patterns of histone marks (e.g., promoter-focused H3K4me3 or H3K27me3) typically observed in somatic and embryonic stem cells. In contrast, "non-canonical" describes the broad, domain-wide distributions observed for certain marks (including ncH3K4me3 and ncH3K27me3) in oocytes and early embryos(Lu *et al*, 2021; Zhang *et al*, 2016). However, H4K20me1

in somatic cells is also broad and non-canonical form. Therefore, we recognize that labeling it as “non-canonical” based solely on its breadth is indeed problematic and conflates a general chromatin feature with a stage-specific phenomenon.

To address this, we have removed the “non-canonical” description from our manuscript. Instead, we now provide a much more precise, descriptive account of our observations. We describe H4K20me1 in oocytes and embryos as forming “broad, gene body-enriched pattern”. This change eliminates the ambiguous label and focuses the narrative on the dynamic redistribution of the mark.

Second, we have rewritten the relevant sections (page 7) to precisely describe our observations, removed the phrase “evolutionarily conserved across vertebrates” and replaced it with the more accurate and limited statement above, specifying conservation among the three studied species: “These data indicate that, the broad-domain distribution pattern of H4K20me1 in early development is a common feature of embryos in mice, zebrafish, and humans. However, the timing of its redistribution relative to ZGA, as well as its enrichment levels and fine-scale genomic localization, exhibit notable species-specific differences.”

4. The distinction between Promoter and Gene body H4K20me1 coverage (e.g., Fig 3) is potentially imprecise, since there can be continuous signal that overlaps both. This becomes very apparent looking at the ATAC-seq signal in Fig 4B, which shows that the highest concentration of ATAC-seq coverage is at the TSS for both the promoter category and the gene body category (not surprisingly). Are many of the same gene loci represented in both categories? Perhaps the authors could more precisely define these two gene groups for the various analyses (Fig 3, 4) so that they more strongly reflect functionally distinct sets of genes (e.g., poised versus actively transcribed).

<u>It also seems like H4K20me1 profiles would be changing from promoter-centered to gene-body for many of the genes, so unifying the analyses in Fig 3 would be beneficial, and showing heatmaps over genes divided into the different categories of change would be very informative.</u>

Response:

Thanks a lot for your notes that imprecise definitions could lead to ambiguous interpretation. We have now explicitly defined our analysis regions in the revised manuscript: The promoter is defined as the region spanning from 3 kb upstream to the TSS, and the gene body as the region from the TSS to the TES. While individual gene may contribute to signal in both categories, the classification is based on the distribution pattern of H4K20me1 enrichment within these strictly defined intervals, allowing us to compare distinct chromatin modification states.

As suggested, we have adopted a functionally relevant classification based on paused versus non-paused transcriptional states. Following established methods(Day *et al*, 2016), genes were classified into two groups according to their pausing index: genes with a pausing index > 2 were defined as paused, while others were defined as non-paused. We then analyzed H4K20me1 dynamics between these two functional categories. Our analysis revealed that H4K20me1 accumulation before and after the late 2-cell stage is significantly reprogrammed in both paused and non-paused genes (Response Fig 3). Specifically, H4K20me1 signals were categorized into two clusters based on their timing: those predominantly established before ZGA and those newly acquired after ZGA. This also demonstrates that H4K20me1 undergoes stage-specific reprogramming during early development. In the results section (page 9), we added the following description: "To further explore the functional relevance of H4K20me1 dynamics, we classified genes based on their transcriptional pausing state. Following established methods(Day *et al*, 2016), genes were classified into two groups: genes

with a pausing index > 2 were defined as paused, while others were defined as non-paused. Our analysis revealed that H4K20me1 accumulation was significantly reprogrammed before and after the late 2-cell stage in both gene categories, clustering into signals established pre-ZGA and those acquired post-ZGA (Fig EV3D and E)."

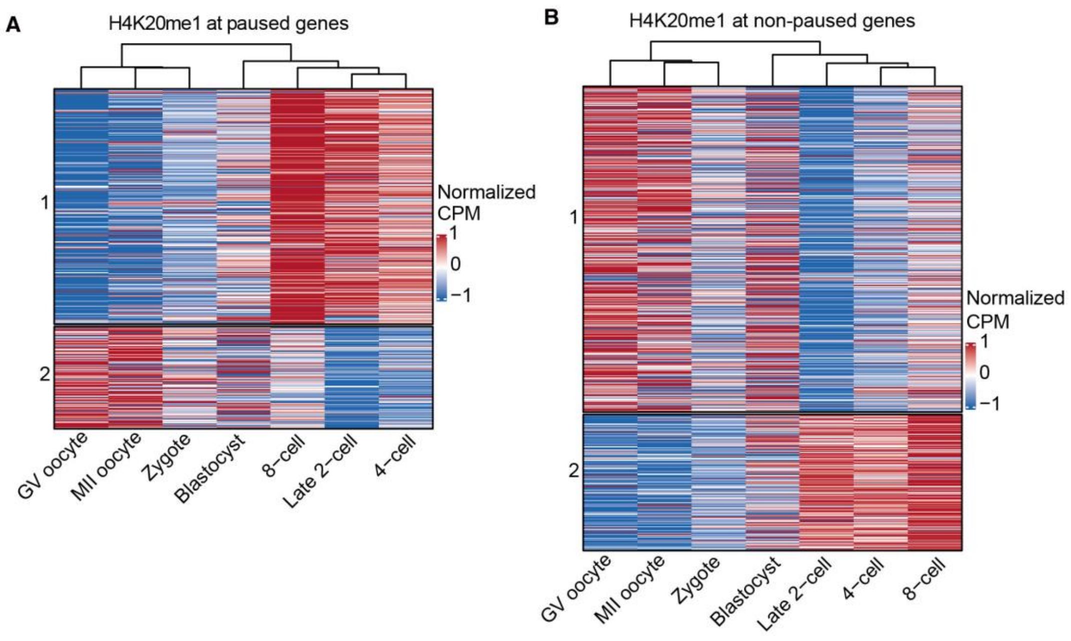

Response Fig 3. Reprogramming of paused and non-paused genes by H4K20me1 in mouse oocytes and early embryos. Hierarchical clustering analysis and dynamics of H4K20me1 enrichment at paused genes (A) or non-paused genes (B) in mouse oocytes and early embryos. Genes are clustered into two groups. CPM, counts per million. These figures were added as Fig EV3D and E.

In addition, we agree that our original Fig 4B and 4C, which displayed metaplots over larger genomic windows for visualization, could have been misinterpreted regarding the exact regions used for downstream quantification. We have revised these figures to eliminate ambiguity: The labels "promoter" and "gene body" above the original panels have been replaced with "TSS-centered" and "Gene-centered", respectively. The figures now explicitly state that while these panels visualize broader regions for

context, the quantitative analyses for the “promoter” and “gene body” categories (e.g., in Fig 4D) use only the strictly defined promoter region (3 kb upstream of TSS) and gene body region (TSS to TES). We have also added a detailed description of these region definitions in the methods section (page 25).

5. The micrographs in Fig 5 (both fluorescence and brightfield) are very hard to see. For the fluorescence images, false coloring (e.g., cyan instead of blue) or using inverted grayscale would help. For the brightfield (Fig 5D, I), the figures need to be much larger for the reader to be able to assess the stages/morphologies.

Response:

Thank you very much for your kind suggestions, we have repeated the experiments and replaced the micrographs in Fig 5 to ensure optimal visibility and assessment of embryo morphology. For the fluorescence images, we reacquired all images under consistent imaging parameters to ensure clear visibility and comparability across different developmental stages (Response Fig 4). For the brightfield, the figures have been significantly enlarged, and the image resolution has been enhanced to allow readers to clearly assess embryonic stage and overall morphology (Response Fig 5). These improvements are reflected in the revised Fig 5A and C.

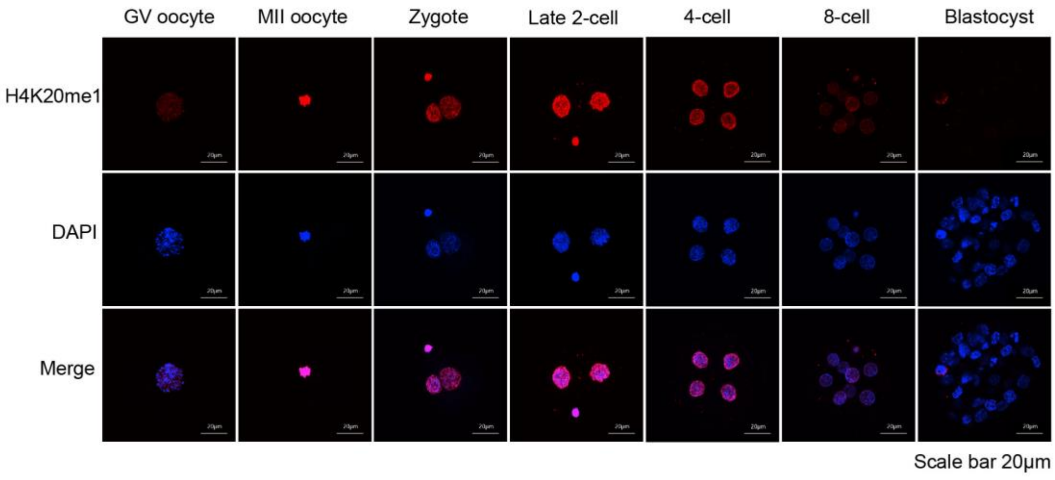

Response Fig 4. H4K20me1 localization during mouse preimplantation development. Immunofluorescence images showing H4K20me1 (red), DAPI-stained nuclei (blue), and the merged signals in oocytes and early embryos. Scale bar, 20 μm.

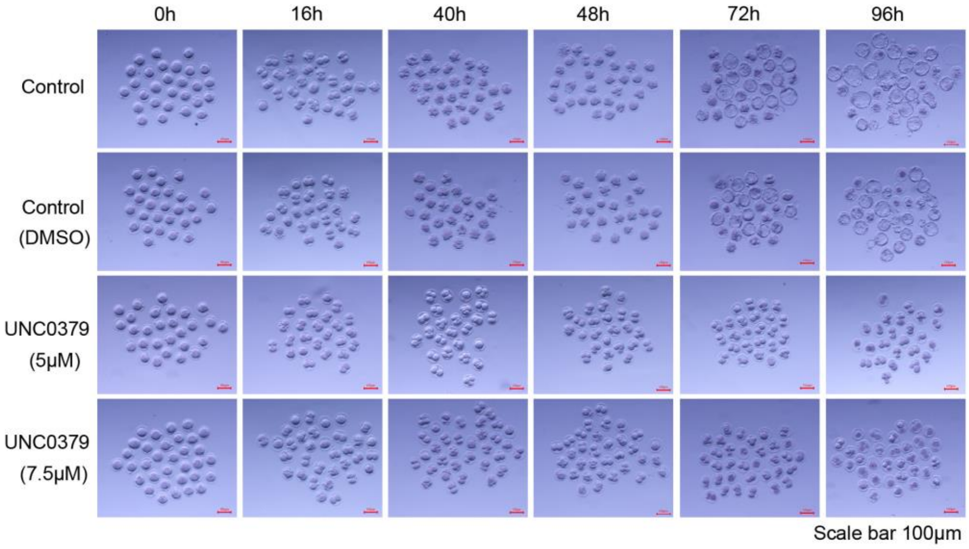

Response Fig 5. Developmental progression of mouse embryos under H4K20me1 inhibition. Representative images of embryos from the Control, DMSO control, 5 μM UNC0379, and 7.5 μM UNC0379 groups are shown at the indicated time points after treatment. The experiment was independently repeated three times. Scale bar, 100 μm.

6. Regarding the effect of H4K20me1 on ZGA raises, the authors don't seem to address that the majority of gene expression defects on H4K20me1 inhibition are for genes unrelated to ZGA. Additional analysis is warranted here -- is H4K20me1 inhibition leading to ectopic expression of some genes? If so, when during development would these genes normally be expressed? And there are 1019 genes not related to ZGA that are downregulated -- what are these? One might suggest they are maternal mRNA that are somehow destabilized, but the graphs in Fig 7B are very confusing in this regard (indeed, please clarify all of Fig 7B). Ultimately, by definition, every mRNA detected in the embryo at these stages must be either maternal or zygotic expressed during minor or major ZGA,

so the authors need to clarify what these other genes are. Also, Fig S4 title says "Loss of H4K20me1 in early embryos mainly affects major ZGA" which seems incorrect in light of this.

Response:

Thank you very much for your questions and suggestions. Actually, we did not address that the majority of gene expression defects on H4K20me1 inhibition.

We have adopted a clear, literature-based criterion for gene classification(Liu *et al*, 2020). ZGA genes are now defined as those with high expression at the late 2-cell stage (FPKM > 5) but low expression in MII oocytes (FPKM < 5), while maternal genes are defined as those with high expression in MII oocytes (FPKM > 5). Applying this revised classification, among the upregulated genes upon H4K20me1 inhibition, we identified 78 ZGA genes (5.7%) and 544 maternal genes (39.7%). Among the downregulated genes, there are 490 ZGA genes (37.3%) and 289 maternal genes (22.0%) (Response Fig 6A and B). ZGA genes are significantly downregulated, indicating that H4K20me1 affects ZGA gene expression (Response Fig 6C).

The remaining differentially expressed genes consist of constantly inactive genes (FPKM < 5 in all stages) and stage-specific genes that are normally activated at other developmental time points. Among the upregulated genes, maternal genes and inactive genes constitute primary categories, accounting for 39.7% and 30.9% respectively (Response Fig 6D and E). However, the overall expression level of maternal genes is not significantly altered (Response Fig 6F).

In addition, the original Fig 7B primarily illustrated the proportions of different gene types among differentially expressed genes, as well as the ratios of up- and down-regulated ZGA genes. However, it indeed contained redundant information and was insufficiently explained, leading to confusion. We have now updated the Fig 7

and legend based on the reclassification results to ensure clarity. The title of original Fig S4 (now Fig EV5) was changed to “Loss of H4K20me1 in early embryos affects the expression of ZGA genes” to more accurately reflect our findings.

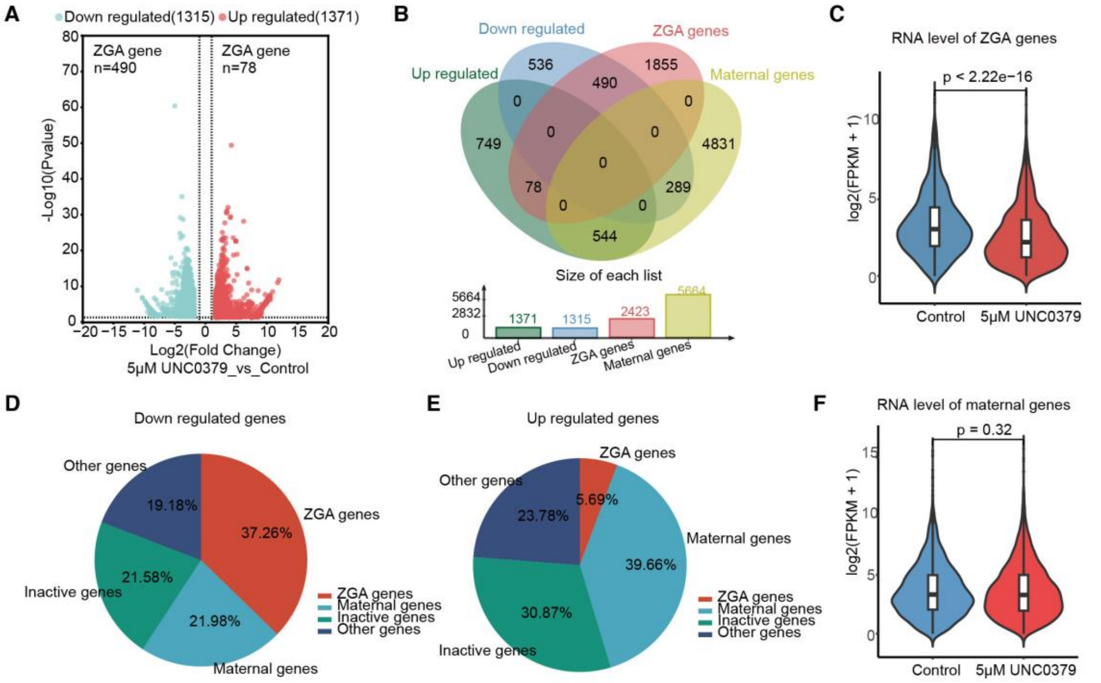

Response Fig 6. Gene expression changes upon H4K20me1 inhibition in early mouse embryos. A) RNA-seq analysis of 2-cell embryos from control and 5 μM UNC0379-treated group. Volcano plot displays changes in gene expression. Red and blue dots indicate significantly upregulated and downregulated genes, respectively. B) Venn diagram illustrating overlaps among ZGA genes, maternal genes, upregulated genes, and downregulated genes following H4K20me1 inhibition. The total number of genes in each category is indicated. C) Violin plot showing significantly downregulated expression of ZGA genes in the 5 μM UNC0379 group compared to control. D-E) Pie charts showing the percentage composition of downregulated (D) and upregulated (E) genes, categorized as ZGA genes, maternal genes, inactive genes, and other gene types. F) Violin plot showing no significant change in maternal gene expression between control and 5 μM UNC0379 group.

Additional points:

7. Many of the figures are low-resolution with small text that is difficult to read.

Response: Thank you very much for your suggestion. We have improved the resolution of the figures and resized the fonts to make them clearer.

8. Most of the legends are very sparsely written, and not all the figure labeling is intuitive enough to understand. Please ensure that sufficient information is included for each figure panel. Some examples I noticed: what is the significance of the purple highlighting in Fig 1A? Is Fig 1D, 6F, and 7C, 7D showing *average* density across all genes, or sum? Is Fig3E,G *log2* normalized CPM? What does "development rate" mean in Fig 5E (is this proportion of embryos that attain each stage? That is not what I would interpret to mean development rate)?

Response: We have revised all figure legends to provide more detailed and comprehensive information. Specifically, the purple highlighting in Fig 1A indicates the representative region shown in the tracks. Fig 1D, 6F (now Fig EV5D), 7C (now Fig 7D), and 7D (now Fig 7E) display the average density across all genes. Fig 3E, G show Z-score normalized expression values (based on CPM). In Fig 5E (now Fig 5D), the label “development rate”, which referred to the proportion of embryos that developed to each stage, has been clarified to “Proportion of embryos at each stage (%)” to accurately reflect the metric shown.

9. Why is the GO enrichment analysis in Fig 6E showing enrichment score but in Fig 3F,H it is -log10 P? (Also, is this multiple test corrected P?)

Response: Regarding the Fig 6E displaying an enrichment score, we apologize for the imprecision noted here. The horizontal axis in this figure actually represents -log10(p-value), and has been corrected to be consistent with Fig 3F and 3H. The

p-values in these analyses were adjusted using the false discovery rate (FDR). We have also updated the relevant figure legends to explicitly state this.

10. Fig 6D and E use a non-accessible red-green color scheme, which should be switched to something that is discernable to red-green colorblind readers

Response: We have modified the figures to avoid the red-green color scheme for better accessibility, and presented in the revised Fig 6D and E.

To Referee #2:

In this manuscript, Meng and colleagues profile the genome-wide distribution of H4K20me1 in mouse, human and zebrafish early embryos. They indicate the presence of a non-canonical distribution of H4K20me1 in all three species, which is reminiscent of other histone modifications at this stage of development. However, they also present species-specific differences in the timing and distribution of the modification across early embryonic development. The majority of the downstream analysis is performed on the mouse data, showing reprogramming of promoter and gene body H4K20me1 and correlation to chromatin accessibility data. Functional experiments in mouse embryos, using an inhibitor of Set8, the only H4K20me1 methyltransferase, show a developmental arrest at the 2-cell stage, with a proportion of major ZGA genes downregulated associated with changes in chromatin accessibility. While Set8 has previously been described to be essential for early embryonic development in mouse, through knockout experiments, H4K20M mutant expression and use of the same inhibitor as in this manuscript (referenced papers: Oda et al., 2009, Shikata et al., 2020), the analysis of transcriptome and chromatin accessibility changes upon perturbation are novel.

Thus, the dataset presented here along with functional experiments have the potential

to provide functional insight with physiological relevance that will have an impact on both developmental biology and the chromatin/epigenetics communities. However, I have several concerns over the experimental design, presentation and interpretation of the results that need to be addressed before publication. In particular, I find a number of conclusions not supported by the data and inconsistencies between figures, as detailed below. This could be partly related to the quality of the English language, which hinders the communication of the results and conclusions. In addition, the Figures and legends often lack enough detail, such as the scale, to understand what is shown.

Response: Thank you very much for the positive assessment of the novelty and potential impact of our study, and for the constructive critiques regarding experimental design and data presentation. We have undertaken a comprehensive revision to address these concerns, as outlined in our point-by-point responses. Based on your suggestions, after we revised the manuscript, this study will be high quality.

Specific comments

Major

1. Inhibition of Set8 leads to changes in H4K20me2/3 levels in mouse preimplantation embryos (Oda et al, 2009) as H4K20me1 is a substrate for the generation of H4K20me2/3. Therefore, the authors cannot rule out the phenotypes observed are due to altered levels of H4K20me2/3. This should be made clear in the manuscript.

Response:

We appreciate and fully agree that because Set8 specifically catalyzes H4K20me1, which itself serves as the substrate for H4K20me2/3 generation, the observed developmental phenotypes upon Set8 inhibition could, in principle, result from a loss of H4K20me1, a consequent reduction in H4K20me2/3, or a combination of both. To contextualize this issue, we now cite and discuss the work by Wongtawan et al., which

systematically quantified H4K20me1/2/3 levels in mouse preimplantation embryos(Wongtawan *et al*, 2011). Their immunofluorescence data show that while H4K20me1 is detected at all stages, H4K20me2 is absent at the one- and two-cell stages and appears only after the four-cell stage, and H4K20me3 is only detected in the maternal pronucleus of one-cell embryos. Therefore, during the key period from zygote to the early cleavage stages, H4K20me1 likely plays the predominant role compared to H4K20me2/3.

We have added a dedicated paragraph in the discussion section (page 17) to explicitly address this point: “It is important to note that SET8 is the sole methyltransferase for H4K20me1, which is a prerequisite for the formation of H4K20me2/3. Thus, the developmental arrest and transcriptional defects we observe upon SET8 inhibition could theoretically stem from the loss of H4K20me1, a secondary decrease in H4K20me2/3, or both. However, a previous study showed that H4K20me2/3 levels are remarkably low or stage-specific during the early cleavage stages in mouse embryos, with H4K20me2 absent at the one- and two-cell stages and H4K20me3 only detectable in the maternal pronucleus of one-cell embryos(Wongtawan *et al*, 2011). Therefore, while a contribution from diminished H4K20me2/3 cannot be formally excluded, the severe phenotype we observed at the early cleavage stages, coincident with major ZGA, strongly suggests that the presence of H4K20me1 itself is critical for this developmental transition.”

2. It is not clear how the non-canonical vs canonical distribution of H4K20me1 is defined and when it is observed in all three species? Is it defined by the size of the domains or the genomic distribution? Which stages are consistent in terms of domain size or genomic distribution of H4K20me1 with what has been reported in cell lines (of the appropriate matching species), for example, and which are not? How is the non-canonical distribution linked to ZGA as they claim? For example,

<u>the clustering analysis in Figure 3 does not demonstrate a non-canonical distribution, just that the localisation of the mark is different before 2-cell to after 2-cell stage.</u>

Response:

Thank you for these critical and professional points regarding our use of the term "non-canonical", which have helped us improve the precision of our manuscript.

First, we used "canonical" in reference to the patterns of histone modifications like H3K4me3 and H3K27me3, whose peaks are sharp and preferentially enriched at promoters in most somatic and embryonic stem cells. Notably, in mouse oocytes, non-canonical broad domains of H3K4me3 and H3K27me3 have also been observed, distributed across partially DNA methylated domains (PMDs)(Lu *et al*, 2021; Zhang *et al*, 2016). In our initial manuscript, we observed that H4K20me1 in oocytes and embryos exists as broad peaks at promoters and a large number of distal regions, distinct from the sharp, promoter-focused peaks of some canonical pattern. However, H4K20me1 is also known to form broad domains and exhibit gene body enrichment in certain cell lines. Given that the broad pattern is not unique to early embryos, we recognized that labeling it as "non-canonical" based solely on its breadth is problematic, as it conflates a general chromatin feature with a stage-specific phenomenon.

To address this, we have removed the "non-canonical" description from our manuscript. Instead, we now provide a much more precise, descriptive account of our observations. We describe H4K20me1 in oocytes and embryos as forming a "broad, gene body-enriched pattern". This change eliminates the ambiguous label and focuses the narrative on the dynamic redistribution of the mark.

Lastly, we would like to clarify that the broad-domain pattern of H4K20me1 is not

directly defined by ZGA. As correctly noted, we observed a clear change in the genomic localization of this mark before versus after ZGA. Therefore, we propose that the redistribution of H4K20me1, rather than its broad pattern, is linked to ZGA. This hypothesis was the basis for our subsequent investigations. We have updated the relevant descriptions in the manuscript (page 7) to avoid ambiguity and to reflect this interpretation more accurately.

3. The authors conclude that the H4K20me1 signal 'disappears' (page 5) or is 'rapidly lost' (page 6) at the late 2-cell stage. However, there is still clearly enrichment in the example snapshot in Figure 1A at the late 2-cell stage. They also claim that H4K20me1 'subsequently re-establishes itself, gradually stabilizing during the 4-cell stage'. This conclusion is not supported by the data presented in Figure 1B, in which the number of detected peaks at the 4-cell stage are still very low compared to the oocyte and then decrease even further at the 8-cell stage. Overall, these conclusions are also inconsistent with their immunofluorescence data in Figure 5A and in the referenced paper Shikata et al, which do not show any reduction in H4K20me1 signal at the 2-cell stage. The loss of H4K20me1 signal that they claim at the late 2-cell stage is also not consistent with their conclusion that H4K20me1 plays a critical role in major ZGA. Is the reduction in H4K20me1 peaks that they observed in the genomics data due to either data quality at the 2-cell stage or an artifact of the peak calling strategy? Information about the sequencing data quality should be provided, such as number of reads and mapping statistics should be shown. How was the peak calling algorithm optimised? Data of % genome covered by peaks and % signal in peaks across stages should be shown.

Response:

Thank you very much for your constructive and valuable suggestions. We agree that our initial descriptions were overstated and have thoroughly revised the manuscript

and deepened our analysis accordingly. We have removed the imprecise terms such as "disappears" and "rapidly lost" from the manuscript (page 5 and 6). The text now accurately describes our findings: "H4K20me1 undergoes extensive reprogramming during the maternal-to-zygotic transition," and "the signals were substantially reduced at the late 2-cell stage."

To better answer the question, we now provide a detailed supplementary table (Appendix table S2) with complete sequencing and mapping statistics for all samples, including total reads, uniquely mapped reads, mapping rate, and Fraction of Reads in Peaks (FRiP) scores. The parameters of the peak calling algorithm are described in the methods section (page 25).

Regarding the apparent discrepancy between the CUT&RUN data (fewer peaks at the 2-cell stage) and the sustained nuclear signal seen by immunofluorescence in Fig. 5A and in Shikata et al., we propose the following explanation: IF measures the global, diffuse abundance of H4K20me1, which remains largely stable. In contrast, CUT&RUN identifies discrete, high-confidence genomic loci where the mark is specifically enriched. The sharp drop in peak numbers at the 2-cell stage indicates a spatial redistribution and focal refinement of H4K20me1—from broad domains to a more targeted, locus-specific pattern. We have elaborated this model in the discussion section (page 15) as follows: "Notably, the apparent nuclear H4K20me1 signal detected by immunofluorescence at the 2-cell stage indicates its retention during this key period. The decrease in locus-specific peaks observed by CUT&RUN may thus reflect not a loss of function, but a transition in its regulatory mode—a spatial redistribution from a broad pattern to a more precisely targeted configuration at select genomic sites, potentially to execute stage-specific functions."

5. The validation of the data presented in Figure S1 is not clearly presented. It is

not clear what is shown in Figures S1A and D as there is no axes labels. In addition, it seems a log scale would be more appropriate here as most of the data points are overlapping. The spearman correlation test should be used rather than the pearson, unless they show that the data is normally distributed. It is not clear what is shown in Figure S1B: correlation between biological replicates of the same sample (e.g. 1,000 cells vs 1,000 cells) or correlation of each sample to bulk (e.g. 100, 200, 500 cells to bulk) or 100 cells vs other samples (100 vs 100, 200, 500 etc)? Please include validation of human and zebrafish H4K20me1 Cut&Run data by correlation of replicates, at least.

Response:

Thank you very much for prompting us to clarify these points, we have addressed each of the points in the revised manuscript, as detailed below:

First, Fig S1A demonstrates the correlation between H4K20me1 ChIP-seq data and CUT&RUN data generated from varying cell numbers (100, 200, 500, 1000, 5000, and 10000 cells) in K562 cells. Fig S1D shows the correlation between two biological replicates of H4K20me1 CUT&RUN data across stages from GV oocyte to blastocyst in mice. As suggested, we have changed both plots to a log scale and employed the Spearman correlation test instead of Pearson correlation (Response Fig 7 and 8). More detailed descriptions have been added to the axis labels, and the updated figures are presented in the Appendix Fig S1 and S3A.

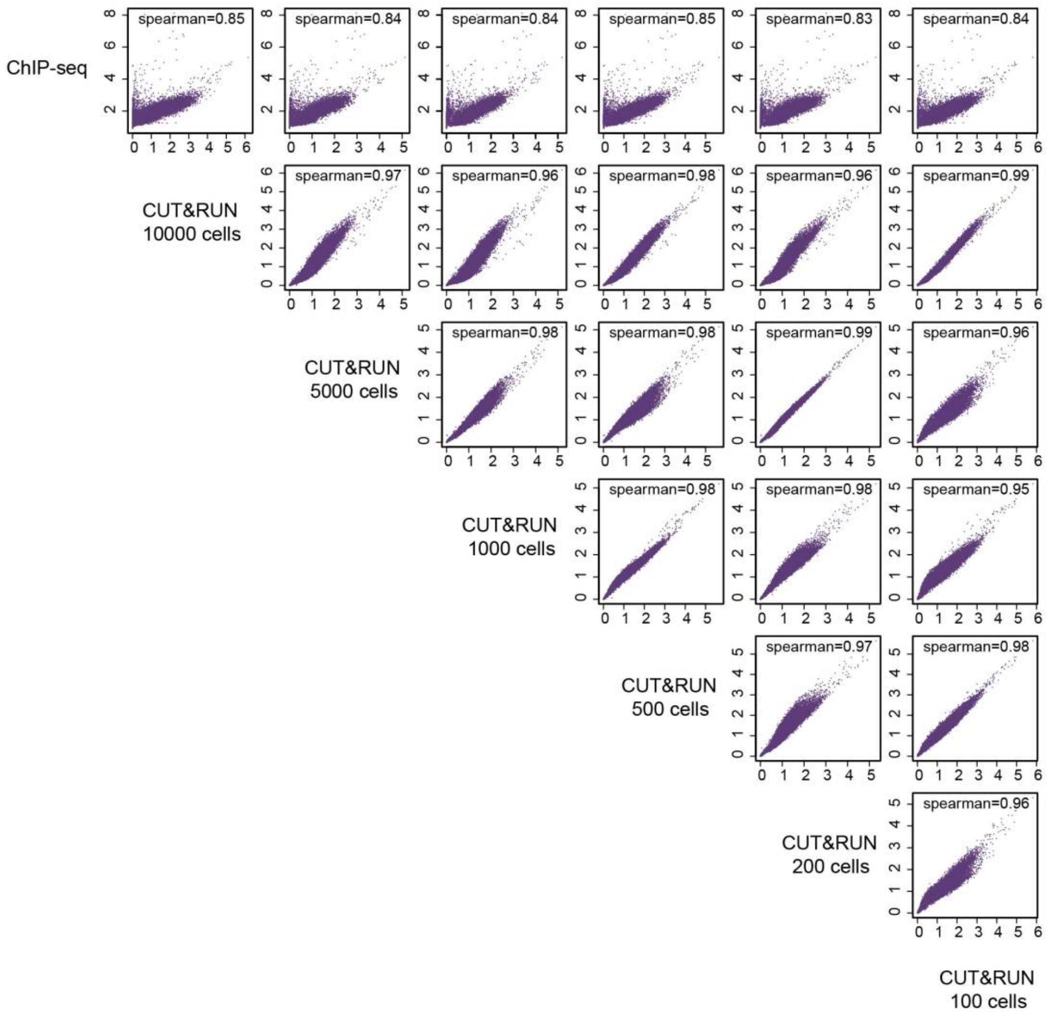

Response Fig 7. Validation of the low-input CUT&RUN approach. Spearman correlation analysis showing that high correlations between different cell inputs in K562 cells of CUT&RUN data and ChIP-seq data of H4K20me1.

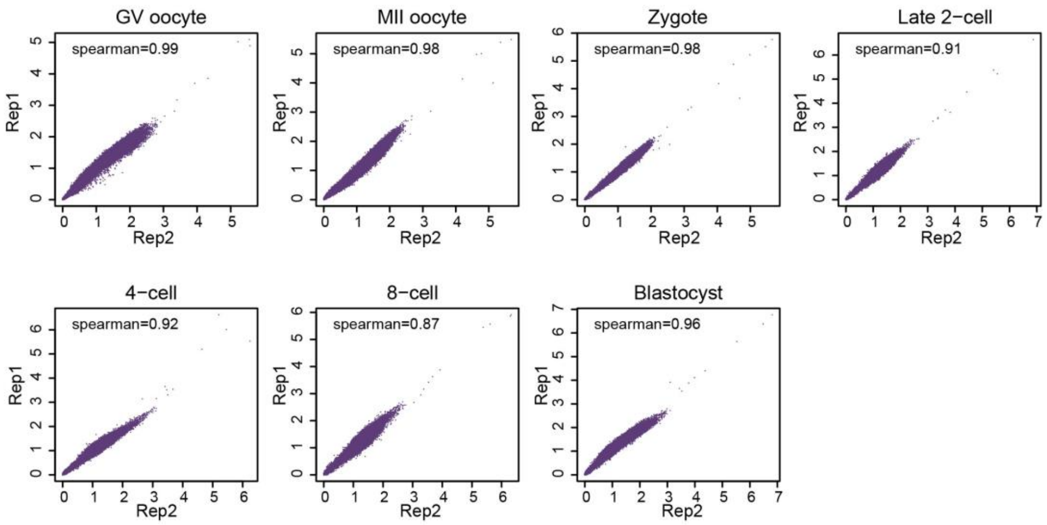

Response Fig 8. Correlations between biological replicates of CUT&RUN in mouse. The strong correlations between biological replicates at each stage from GV oocyte to blastocyst in mice demonstrate the high quality of the CUT&RUN data.

Second, Fig S1B has been revised for clarity. The blue bars represent the correlation between H4K20me1 CUT&RUN data (from the indicated K562 cell numbers) and the ChIP-seq data. The red bars represent the correlation between two biological replicates of the CUT&RUN data at the same cell number. The legend has been updated with a detailed explanation, and the updated figures are presented in the Appendix Fig S2A.

In addition, we have added the validation of zebrafish and human H4K20me1 CUT&RUN data by correlation of biological replicates (Response Fig 9 and 10). These results are presented in Appendix Fig S3B and C. Specifically, we observed relatively lower correlation between the replicates for the human 4-cell and, to a lesser extent, 8-cell stage embryos. This is likely attributable to the extremely limited sample availability and the consequent technical variability, including differences in sequencing depth among these rare specimens. The overall low signal is also reflected in the smaller number of peaks called for these stages, which is consistent with Fig 1F

and the immunofluorescence data presented in Fig EV2B and C. We have addressed these points in the discussion section (page 15-16).

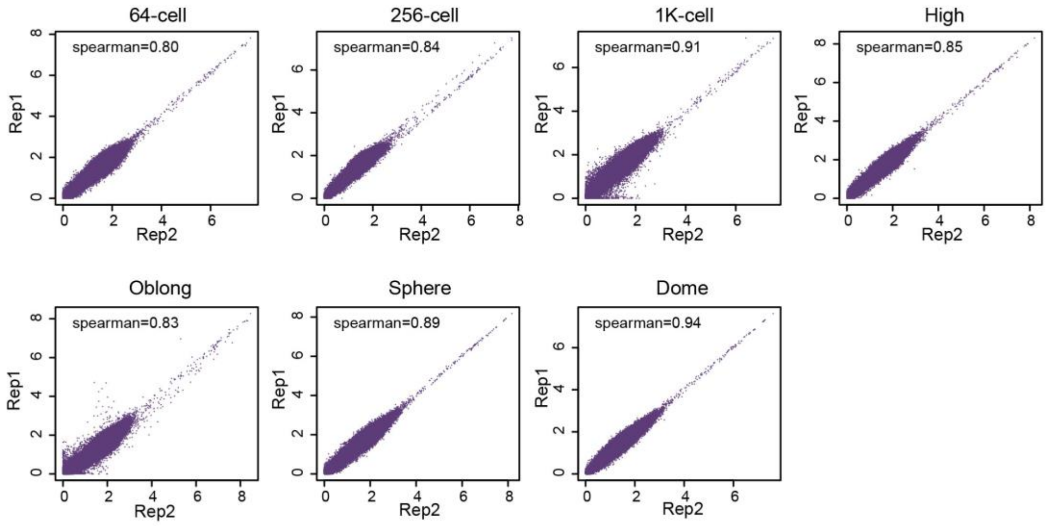

Response Fig 9. Correlation between CUT&RUN biological replicates in zebrafish. Spearman correlation analysis of CUT&RUN data across zebrafish embryonic stages, demonstrating the high reproducibility between biological replicates.

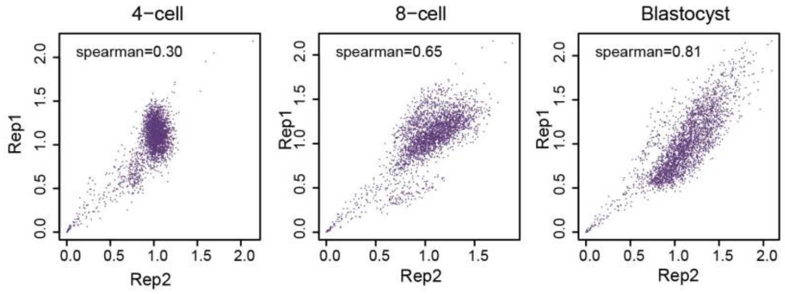

Response Fig 10. Correlation between CUT&RUN biological replicates in human embryos. Spearman correlations between biological replicates are shown for the 4-cell, 8-cell, and blastocyst stages. The lower correlations observed at the earlier stages (particularly the 4-cell stage) are likely attributable to the extremely limited sample availability and the resulting technical variability, including differences in sequencing depth between these rare specimens, along with the low number of peaks

detected.

6. Immunofluorescence.

The immunofluorescence images including the DAPI channel are very weak throughout. Please improve the images, by presenting them in greyscale and increasing the brightness/contrast (consistently across each panel), if necessary. How was quantification of fluorescence intensity performed? It is not sufficient to write 'using ImageJ'. How was the region of interest selected? Was the mean fluorescence intensity calculated in a single z slice or across multiple slices? Please include the information on replicates (number of independent experiments and number of embryos) throughout.

Response:

Thank you very much for your kind suggestions, we have repeated the immunofluorescence experiments and increased the sample size. All IF images throughout the manuscript, including the DAPI channels, have been reprocessed for improved clarity (revised Fig 5A; please see also Response Fig 4). The details for fluorescence intensity quantification have been added to the methods section (page 21-22) as follows:

"Immunofluorescence images were obtained using a Zeiss 700 confocal microscope, maintaining consistent imaging parameters across all samples. For each embryo, a Z-stack consisting of five slices with a fixed step size was collected and merged into a single composite image via maximum intensity projection to capture complete cellular information. Quantification was performed on these projected images using ImageJ: images were converted to 8-bit grayscale and uniformly thresholded with the default algorithm to define regions of interest, and the mean intensity was measured."

The information on replicates has been added in the Figure 5 legend: "The experiments were independently repeated three times, and the total number of

embryos analyzed per developmental stage was as follows: GV oocyte (21), MII oocyte (24), zygote (30), 2-cell (29), 4-cell (28), 8-cell (43), and blastocyst (28)."

7. Development experiments.

According to the methods the control group are embryos grown in culture medium. The experiment should be controlled by testing the solvent of the inhibitor (i.e. DMSO) diluted in culture medium to the same concentration as with the inhibitor. Please add the timing information to Figure 5E. The development rate to blastocyst in the control group is less than 40%. Typically, over 90% of control embryos reach the blastocyst in this assay. Please also add an additional later timepoint (e.g. 96h) to determine whether the embryos are arrested or delayed in development to blastocyst in the control groups. Quantification of the results of Figure 5I should also be provided.

Response:

We appreciate and acknowledge that the omission of a DMSO vehicle control was an oversight in our initial experimental design. In the revised experiments, we have included a control group treated with the same concentration of DMSO used to dissolve UNC0379; we also extended the culture period to 96 hours as suggested. The results show no significant difference in developmental progression at each stage between the standard culture medium control and the DMSO vehicle control, confirming that the solvent itself has no adverse effect. Over 70% of embryos in control groups developed to the blastocyst stage. In contrast, embryos treated with UNC0379 continued to exhibit significant developmental arrest (see updated Fig 5C or Response Fig 5, with quantitative data in Response Fig 11A). The corresponding timing information has been added to the figure legend of the updated Fig 5D. Furthermore, the quantitative analysis for the results originally shown in Fig 5I (now Fig 5E) has been provided in Response Fig 11B and added as Fig EV4E.

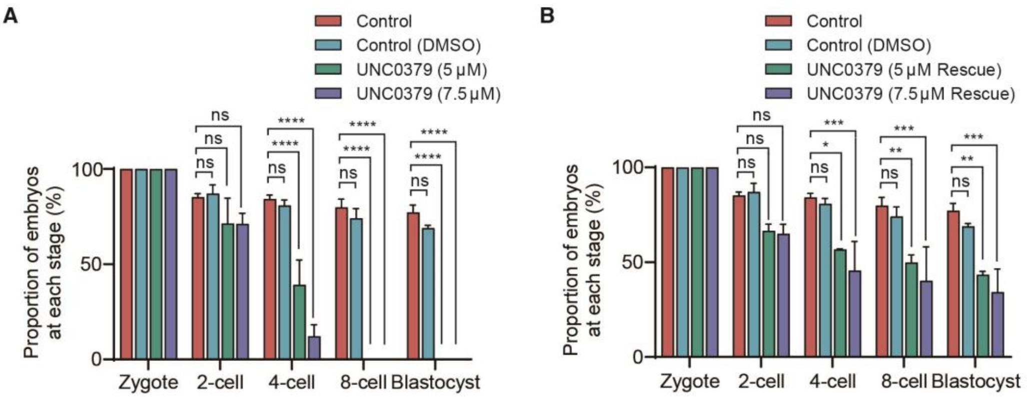

Response Fig 11. Developmental progression of embryos under different treatment conditions. Embryos were cultured in vitro and assessed at the indicated time points: zygote (0 h), 2-cell (16 h), 4-cell (40 h), 8-cell (48 h), and blastocyst (72-96 h). (A) The proportions of embryos at each stage in the standard medium control, DMSO vehicle control, 5 µM UNC0379, and 7.5 µM UNC0379 groups. (B) The proportions of embryos at each stage in the standard medium control, DMSO vehicle control, 5 µM UNC0379 rescue, and 7.5 µM UNC0379 rescue groups. The experiment was independently repeated three times. Embryo numbers per group: Control, n = 89; DMSO, n = 72; 5 µM UNC0379, n = 88; 7.5 µM UNC0379, n = 90; 5 µM UNC0379 rescue, n = 91; 7.5 µM UNC0379 rescue, n = 85. Data are shown as mean ± SEM. Statistical significance: n.s., not significant; *$P < 0.05$, **$P < 0.01$, ***$P < 0.001$, ****$P < 0.0001$.

8. Chromatin accessibility analysis.

The three datasets mentioned GSE116857, GSE136403, GSE66390 did not analyze 'the association between H4K20me1 and chromatin opening in early mouse embryos' (page 9). None of these datasets include data for H4K20me1.

The data in Figures 4B/C seem to contradict Figure 4D at both promoters and gene bodies. For example, in Figure 4B high H4K20me1 promoters display reduced chromatin accessibility at the late 2-cell stage. However, in Figure 4D all promoters seem to have both high H4K20me1 and ATAC-seq signal. This may be

due to a scaling issue as the scale of the axes in Figure 4D is not described. A log scale may be more appropriate here.

The claim that 'the gene body regions of H4K20me1 were consistently and strongly positively correlated with chromatin accessibility' (page 10), which is apparent in Figure 4D, is not clear in Figure 4C. For example, in blastocyst very little ATAC-seq signal is observed in the gene body in either H4K20me1 high or low groups. The signal is almost exclusively focused at the TSS. Do they include the TSS signal as gene body? If so, the TSS should be excluded from gene body analysis.

To support the authors' interpretation that H4K20me1 regulates chromatin accessibility they should test whether DARs correlate to peaks of H4K20me1 genome-wide.

Response:

Thank you for raising these crucial points regarding the chromatin accessibility analysis. We provide the following clarifications and revisions.

First, the datasets (GSE116857, GSE136403, GSE66390) are publicly available ATAC-seq datasets from early mouse embryos. In this study, we performed the joint analysis to examine the association between our generated H4K20me1 CUT&RUN data and chromatin accessibility states from these published datasets. We apologize for the ambiguous phrasing. The text has been revised for clarify to read: "This relationship was further validated through a joint analysis of our H4K20me1 data with published chromatin accessibility datasets (Data ref: GSE116857, 2019; GSE136403, 2020; GSE66390, 2016) in early mouse embryos."

Second, regarding the relationship between Fig 4D and other panels, Fig 4D specifically focuses on the correlation between H4K20me1 and ATAC-seq signals. Both axes in the scatter plots represent log2(FPKM+1) values, and the axis scales

have now been explicitly labeled. For promoters (defined as regions 3 kb upstream of the TSS), a positive correlation is observed from oocytes to the zygote stage ($\rho > 0.5$), which decreases significantly from the late 2-cell stage to blastocyst (ρ values from 0.078 to 0.047). For gene bodies (from TSS to TES), a strong positive correlation ($\rho > 0.9$) is maintained throughout development. While the overall ATAC-seq signal in gene bodies at the blastocyst stage is not low—as reflected by the y-axis scale—we acknowledge that the signal is indeed most concentrated at the TSS.

We recognize that our original definition of “gene body” included the TSS. Given that H4K20me1 is primarily enriched in introns, we performed an additional correlation analysis focusing specifically on intronic regions while excluding the TSS. The correlation between H4K20me1 and ATAC-seq signals within introns remains highly positive ($\rho \geq 0.79$), robustly supporting our conclusion (Response Fig 12). This new analysis was added as Fig EV3F.

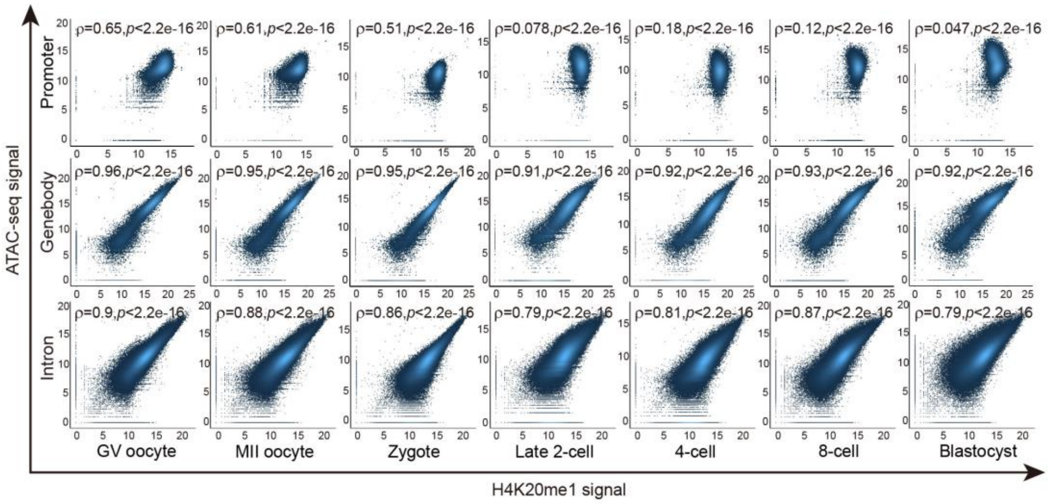

Response Fig 12. Correlation between H4K20me1 and ATAC-seq signals in mouse oocytes and early embryos. Scatter plots comparing H4K20me1 signals with ATAC-seq signals in promoters, gene bodies, and introns across developmental stages. The axes in the plots represent log2(FPKM+1) values. Spearman correlation coefficients and p values are indicated.

Finally, in response to the suggestion to test whether differentially accessible regions (DARs) correlate with H4K20me1 peaks genome-wide, we analyzed the overlap between them. Among the loss-of-accessibility DARs, 1507 regions (57.7%) overlapped with H4K20me1 peaks. Notably, these overlapping peaks constituted 74.6% of all H4K20me1 peaks identified in control embryos. In contrast, only 37 gain-of-accessibility DARs (33.9%) overlapped with H4K20me1 peaks (Response Fig 13). This significant overlap between H4K20me1 peaks and loss DARs indicates a specific role for H4K20me1 in maintaining chromatin accessibility at these loci. These results are now presented in Fig EV5A-C and described in the results section (page 12).

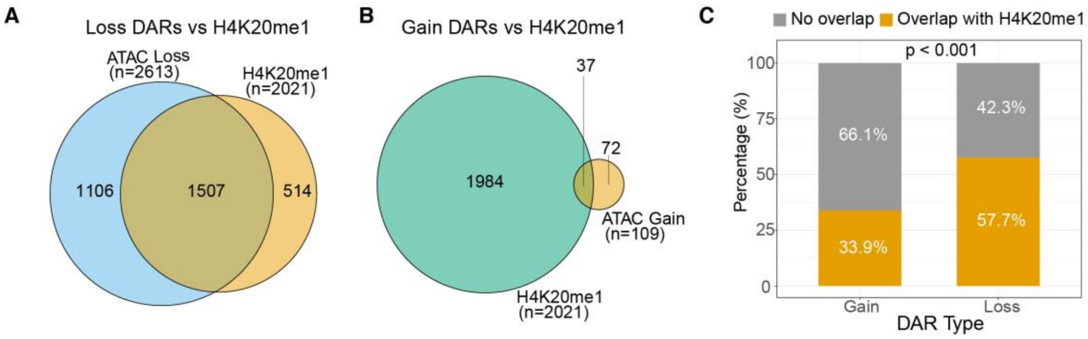

Response Fig 13. Overlap of H4K20me1 peaks with differentially accessible regions. (A-B) Venn diagrams showing the overlap between H4K20me1 peaks in control 2-cell embryos and (A) DARs with lost accessibility or (B) DARs with gained accessibility upon H4K20me1 inhibition. Numbers indicate the counts of peaks or regions in each category and their intersection. (C) Stacked bar plots showing the proportion of Gain DARs (left) and Loss DARs (right) that overlap or do not overlap with H4K20me1 peaks.

9. English language

The English should be checked and improved throughout. Here are a few

examples:

• In Figures 1C, 1G and 2C legends the data does not show 'the proportion of the genome covered by H4K20me1' but rather the proportion of H4K20me1 signal in different genomic features.

• 'We found that there existed embryos that were able to develop backward across the 2-cell stage' (page 11).

• 'All three clusters exhibited erasure of MII oocyte-zygote embryos, followed by re-establishment at late 2-cell embryos, and clusters differed only in H4K20me1 levels' (page 8).

• 'the distribution of H4K20me1 shifted from the TSS to the two sides of the genome in late 2-cell embryos, resulting in a transition to a bimodal pattern' (page 6).

• 'late 2-cell stage embryos exhibited significantly reduced chromatin accessibility' (page 9-10). This analysis is not genome-wide and thus they cannot conclude that there is reduced accessibility at the late 2-cell stage in general.

Response:

Thank you for the detailed corrections regarding English expression. We have carefully reviewed the entire manuscript and made extensive revisions to improve clarity and precision. The specific examples highlighted have been addressed as follows:

• The legends for Fig 1C, 1G, and 2C have been corrected to accurately describe the data, now stating "the proportion of H4K20me1 signal in different genomic features".

• The phrasing on page 11 has been rephrased for clarity: "We found that there existed embryos that were able to develop to the blastocyst stage."

• The sentence on page 8 has been revised as follows: "All three clusters shared a similar pattern, differing primarily in their H4K20me1 signal levels."

• The description on page 6 has been clarified to: "...the distribution of H4K20me1 shifted from the TSS to the flanking regions at the late 2-cell stage, resulting in a

bimodal pattern."

• The sentence on page 10 has been rephrased for clarity: "at the late 2-cell stage, chromatin accessibility in these promoter regions was significantly reduced," avoiding an overgeneralized claim.

Minor

The alternative names of Set8 should be described upon its first introduction (KMT5A, Pr-Set7/9)

Response: Following your helpful suggestion, we have now described the alternative names of SET8 (KMT5A, PR-SET7/9) upon its first introduction in the revised manuscript (page 4).

Please include a table detailing sample and the sequencing information. For example, how many embryos were collected at each stage? How many reads/mapped reads were obtained for each sample etc.

Response: We have added the supplementary tables (Appendix table S1 and 2) detailing the sample and sequencing information.

Figure 1A What is the purple shading?

Response: The purple shading in Fig 1A indicates the representative regions shown in the track plots. We have added a clear explanation in the figure legend.

Figure S1C This is not genome-wide.

Response: We have removed the term "genome-wide" from the description of original Fig S1C (now Appendix Fig S2B).

Figure 2. The time of major ZGA in zebrafish should be noted (it would help to mark it on the figures)

Response: We have now indicated the timing of major ZGA in zebrafish on Fig 2.

Figures 2B and C are swapped in the legend.

Response: We have corrected the figure legend.

Figure 3F Cluster 1 not cluster 4 genes are associated with voltage-gated channel activity as described in the text (page 8).

Response: We have corrected this sentence on page 8.

Figure 5D. The magnification of the images are too low to see the phenotype of the embryos.

Response: We have replaced the images in original Fig 5D (now Fig 5C) with higher-magnification views to make the embryonic phenotypes clearer.

Figure 7C. Is the narrow heatmap to the right of the heatmaps for H4K20me1 showing RNA levels? This should be better described.

Response: The narrow heatmap represents log2 fold-change (log2FC), not RNA levels. We apologize for not describing this clearly. We have removed this potentially ambiguous and non-essential element in the revised version. The figure (now Fig 7D) and its legend have been updated accordingly.

Figure 6F and Figure S4D. It is not clear which data are used to assess PolII loading.

Response: The data used to assess Pol II loading were obtained from the publicly available dataset GSE135457. We have clarified this in the respective figure legends (now Fig EV5D).

To Advisor's comments:

The datasets are new and potentially helpful, and this is where I would see the value of the work (no one has reported K20me1 genomics in embryos, which the current paper has). I have, however, two small caveats for this.

First is that the tracks look a little strange, and there is no IgG or input controls. The patterns look suspiciously similar to ATAC-seq, specially at the earliest stages and thus I am not entirely sure that the authors are indeed picking the right K20me1 signal (this could be seen at a formal reviewing step). Also, their interpretations (I believe because of the 'signal' issue) go quite a bit against what is known on K20me1, which oscillates during cell cycle and progressively accumulates during S-phase.

Second, the requirement for early development is not entirely new. The zygotic depletion of PrSet7 results in arrest between the 4-8-cell stage, which has been ascribed to defects in cell cycle progression, in particular S-phase, and signs of DNA damage (and not to transcriptional changes). The authors use inhibitors earlier and report an earlier phenotype, but considering PR-set7 is required for cell viability, it is not clear whether the interpretations of the authors on zygotic genome activation are fully explored and founded.

So, in short, the datasets are definitely potentially useful for the field, but their QC should be properly evaluated. Ideally, the authors should focus on new biology of K20me1 and not the known role of PRSet7 in early embryos, as indeed this angle undermines novelty.

Response:

We sincerely appreciate your careful reading and valuable suggestions. These

comments have been instrumental in strengthening our study. Our detailed responses and the revisions made to the manuscript are as follows:

First, regarding data quality control and signal specificity:

We thank you for recognizing potential value of the H4K20me1 CUT&RUN dataset and understand the concerns about signal specificity. Our CUT&RUN experiments were conducted following standard protocols using a specific antibody against H4K20me1. To address the concerns regarding the lack of Input/IgG controls and the similarity of our signal patterns to ATAC-seq, we have undertaken additional analyses and clarified our interpretations:

1) Data quality validation: To confirm data reliability, we performed systematic validations. First, we validated the specificity of our H4K20me1 antibody and the sensitivity of our micro-scale CUT&RUN protocol in K562 cells, demonstrating that profiles obtained from low cell numbers correlate well with established ENCODE ChIP-seq data (Appendix Fig S1 and S2A-B). For the embryo datasets, we have provided detailed sequencing and alignment statistics in the Appendix Table S2, and assessed the correlation between biological replicates across each developmental stage (Appendix Fig S3), confirming the data quality. Furthermore, following the reviewer's suggestion, we applied the ChIPseqSpikeInFree method to examine potential global scaling effects. The rescaled data showed high correlation with the original CPM-normalized signals across the affected stages (Appendix Fig S2C). Collectively, these validations support the appropriateness of our dataset for the comparative genomic analyses presented in this study.

2) Signal specificity analysis: H4K20me1 promotes chromatin accessibility and is positively correlated with ATAC-seq signals. Notably, H4K20me1 peaks are generally broad, whereas ATAC-seq peaks tend to be relatively sharp. To our

interesting, our analysis also revealed significant regional differences, such as the varying behaviors in the "Promoter lost" regions. In our study, we found that H4K20me1 is highly positively correlated with ATAC-seq signals in the gene body regions, but this correlation undergoes significant changes in the promoter regions between the two-cell stage (Fig 4).

3) Note on cell cycle oscillation: We acknowledge that H4K20me1 levels oscillate during the somatic cell cycle, peaking at G2/M phase. However, this canonical pattern is not strictly maintained in early embryos, and this seems interesting and helpful to this field. At the one-cell stage, H4K20me1 levels do not change significantly with cell-cycle progression, likely due to the uniquely plastic state of zygotic chromatin and its stage-specific regulatory logic(Shoaib *et al*, 2021; Shikata *et al*, 2020). Our data show that H4K20me1 undergoes significant redistribution during zygotic genome activation (ZGA), while its overall nuclear signal remains relatively stable, as quantified by immunofluorescence (Fig 5A and B). This suggests that during embryo development, the function of H4K20me1 may depend more on qualitative changes in genomic localization rather than quantitative fluctuations.

Second, regarding the novelty and mechanistic insight of our study:

We fully agree that embryonic arrest upon the depletion of PrSet7 is a known phenotype, previously linked to cell cycle and DNA damage defects. While prior studies established this phenotype, the mechanism of H4K20me1 epigenetic reprogramming during early development remained unclear. Our work is specifically focused on defining H4K20me1 reprogramming and its role in regulating zygotic genome. By integrating H4K20me1 CUT&RUN, chromatin accessibility (ATAC-seq), and transcriptome (RNA-seq) data, we establish a functional link between reprogramming of H4K20me1, changes in chromatin accessibility, and ZGA gene

expression in early embryos. The inhibition of H4K20me1 not only downregulates ZGA genes but also specifically reduces chromatin accessibility at its target loci. We uncover a novel function of H4K20me1 in regulating chromatin state and transcriptional programs during ZGA. To clarify the relationship of this work to prior studies, we have revised the discussion section (page 16).

In summary, based on your constructive comments, we have strengthened the validation of data quality, clarified issues related to signal specificity, and more precisely defined the biological novelty of this study—specifically, the depiction of the dynamic genomic landscape of H4K20me1 in early embryos and the elucidation of its functional mechanism in ZGA through the influence on chromatin accessibility and regulation of transcription. We believe these revisions have significantly enhanced the overall quality and persuasiveness of the manuscript.

Response References

Day DS, Zhang B, Stevens SM, Ferrari F, Larschan EN, Park PJ & Pu WT (2016) Comprehensive analysis of promoter-proximal RNA polymerase II pausing across mammalian cell types. *Genome Biol* 17: 120

Jin H, Kasper LH, Larson JD, Wu G, Baker SJ, Zhang J & Fan Y (2020) ChIPseqSpikeInFree: a ChIP-seq normalization approach to reveal global changes in histone modifications without spike-in. *Bioinformatics* 36: 1270–1272

Liu B, Xu Q, Wang Q, Feng S, Lai F, Wang P, Zheng F, Xiang Y, Wu J, Nie J, *et al* (2020) The landscape of RNA Pol II binding reveals a stepwise transition during ZGA. *Nature* 587: 139–144

Lu X, Zhang Y, Wang L, Wang L, Wang H, Xu Q, Xiang Y, Chen C, Kong F, Xia W, *et al* (2021) Evolutionary epigenomic analyses in mammalian early embryos reveal species-specific innovations and conserved principles of imprinting. *Science Advances* 7: eabi6178

Shikata D, Yamamoto T, Honda S, Ikeda S & Minami N (2020) H4K20

monomethylation inhibition causes loss of genomic integrity in mouse preimplantation embryos. *The Journal of Reproduction and Development* 66: 411

Shoaib M, Chen Q, Shi X, Nair N, Prasanna C, Yang R, Walter D, Frederiksen KS, Einarsson H, Svensson JP, *et al* (2021) Histone H4 lysine 20 mono-methylation directly facilitates chromatin openness and promotes transcription of housekeeping genes. *Nature Communications* 12: 4800

Wongtawan T, Taylor JE, Lawson KA, Wilmut I & Pennings S (2011) Histone H4K20me3 and HP1α are late heterochromatin markers in development, but present in undifferentiated embryonic stem cells. *Journal of Cell Science* 124: 1878–1890

Zhang B, Zheng H, Huang B, Li W, Xiang Y, Peng X, Ming J, Wu X, Zhang Y, Xu Q, *et al* (2016) Allelic reprogramming of the histone modification H3K4me3 in early mammalian development. *Nature* 537: 553–557

1st Revision - Editorial Decision 13th Mar 2026

Dear Prof. Xu,

Thank you for the submission of your revised ms. We have now received the comments from referees 1 and 2 and I am happy to say that both referees support the publication of your revised study. Both referees have some more comments, and I would like to invite you to address these. In the cross-comments, referee 2 does not agree with all requests raised by referee 1, and it is fine that you address all final comments along the lines suggested by referee 2. Please submit a point-by-point response with your final ms.

A few editorial requests will also need to be addressed before we can proceed with the official acceptance of your ms:

- Both corr. authors need to have institutional email addresses, please add.

- The author credits need to be removed from the ms file. All credits need to be entered during online ms submission.

- Some FUNDING information is missing in our online submission system: Henan Province excellent innovation group project, Leading medical and health personnel of Central Plains region, Henan Province medical appropriate technology extension project. The funding information in the ms file and in our online system must be congruent. Please correct.

- Please upload one source data folder per ms figure.

- Materials and Methods should be just Methods.

- Please note that weblink for the HRA012004 database is not accessible. It needs to be accessible with the submission of your final ms or at the very latest upon the online publication of your study.

- There are 2 tables provided in zip folders titled Appendix Table S1 and Appendix Table S2. I would like to suggest that you rename the files to Table EV1 and Table EV2. The 3 Appendix figures can also be EV figures, you can have 8 EV figures, this is fine, and the Appendix file can then be deleted. Each legend of the EV tables should be provided in a separate sheet/tab in the same Excel file, and all tables and EV figures need to be called out correctly in the ms text.

*Figure Legends - Comments *

- Please note that the exact p values are not provided in the legends of figures 5D, 7C, EV4 C, E, please provide exact values as reasonable.
- Please indicate the statistical test used for data analysis in the legends of figures 3F, H; 6D, 7A, C, EV5 G
- Please note that the box plots need to be defined in terms of minima, maxima, centre, bounds of box and whiskers, and percentile in the legends of figures 7C, EV5 G
- Please note that information related to n is missing in the legends of figures 7C, EV2 C, EV5 G
- Please note that scale bar and its definition are missing for figure 5E.

I would like to suggest some minor changes to the title and abstract that needs to be written in present tense. Do you agree with :

The reprogramming and function of H4K20me1 during early embryo development

Histone modifications play critical roles in regulating chromatin dynamics and embryonic development. Among these, histone H4 lysine 20 mono-methylation (H4K20me1) is an essential epigenetic mark associated with gene expression and genome stability. However, the reprogramming and functional roles of H4K20me1 in early embryogenesis remain unclear. Here, we map genome-wide distributions of H4K20me1 in mouse, human and zebrafish early embryos, revealing a broad distribution pattern along with species-specific features. H4K20me1 is predominantly enriched in gene bodies and undergoes dynamic erasure and reestablishment following fertilization. Functional perturbation of SET8, the only known H4K20me1 methyltransferase, results in developmental arrest, highlighting its necessity for embryogenesis. Mechanistically, H4K20me1 is crucial for zygotic genome activation (ZGA), where it regulates RNA synthesis and transcription, and promotes chromatin accessibility. Our findings provide insights into the dynamic reprogramming and regulatory functions of H4K20me1 in early developmental processes.

EMBO press papers are accompanied online by A) a short (1-2 sentences) summary of the findings and their significance, B) 2-3 bullet points highlighting key results and C) a synopsis image that is exactly 550 pixels wide and 200-600 pixels high (the height is variable). The synopsis image should provide a sketch of the major findings, like a graphical abstract. Please note that text needs to be readable at the final size. Please send us this information along with the final manuscript.

I look forward to seeing a final version of your manuscript as soon as possible.

Best regards,
Esther

Esther Schnapp, PhD
Senior Editor
EMBO reports

Referee #1:

Overall, the authors have generally addressed my concerns. There are still some issues with rigor and clarity:

- The new heatmaps in EV1 provide valuable insight. It is unclear whether they represent a subset of genes or all genes -- if the latter, that would be very surprising given that the vast majority of genes seem to be marked at zygote stage. To put these trends into context and evaluate their impact, it seems like parallel heatmaps of other chromatin characteristics (accessibility, H3K4 methylation, etc.) would be needed. To some extent, the authors address this in Fig 4, but again chose not to show parallel heatmaps of the different chromatin signals, which would better illustrate the locus-specific trends and overall genome-wide (across all genes) effect. Negative controls (e.g., IgG) would also be useful to ensure the signals are specific.

Additionally, the aggregate profiles shown above the EV1 heatmaps don't seem to exactly match the Fig1D aggregate profiles, suggesting that some locus curation may have occurred.

I want to emphasize that the stage-to-stage variability of H4K20me1 coverage relative to genes is compelling, and seems unlikely to arise from any technical artifact. But I think the genomics analyses could have gone further to establish what those precise patterns are, especially relative to what we already understand about embryonic chromatin dynamics at these early stages.

- Response Fig 2, ChIPseqSpikeInFree should be generating uniform scaling factors, which simply scale linearly in the same way as CPM normalization. So, the non-linear trends shown in the graph don't make sense in this regard, unless scaling was applied in an unconventional way.

The statement in the text "The relative H4K20me1 enrichment patterns across these stages were robust, as confirmed by ChIPseqSpikeInFree normalization assessment" is hard to interpret. What does robust precisely mean here?

- Response Fig 3, it's not clear how pausing is defined relative to stage. It seems like genes are grouped into paused/not paused groups that are assessed for H4K20me1 across all stages, but wouldn't we expect different genes be paused at different stages; and that the pausing dynamics during transcriptional quiescence (prior to zygotic genome activation) be challenging to define? This analysis is too under-explained to be compelling.

- Response Fig 4, the micrographs are easier to interpret now, but it is still challenging to see the DAPI signal. I would again suggest greyscale/false coloring

- The added clarity to gene categories in the RNA-seq analysis helps, but still seems to avoid explicitly addressing whether changes are likely due to transcriptional or post-transcriptional effects relative to the genes' untreated dynamics during the maternal-to-zygotic transition. For example, maternal genes (high in MII) can still be further activated at ZGA, or they can be subject to clearance.

Fig 7C is likely not an appropriate comparison. The test does not appear to be stated anywhere -- at minimum, it should be a paired non-parametric test. However, differential expression analysis combined with heatmaps would be a better way to demonstrate such trends.

- In the intro -- I still disagree that H3K4me3 and H3K27me3 exhibit narrow peaks "canonically" -- broad enrichment is a feature of both across contexts, not just in mouse oocytes. Highly transcribed genes have H3K4me1/2/3 that extends well into the gene body, facultative heterochromatin is marked by broad H3K27me3.

- Throughout, the authors use the word significant or significantly, but do not have statistical tests to support significance.

Referee #2:

The majority of my comments have now been addressed. I have a few outstanding comments listed below:

3. I appreciate the additions the authors have made to address this point, including the discussion regarding the difference between the genomics and the immunofluorescence data and the sequencing control quality statistics. This indicates that the fraction of reads in peaks (FRIP) are very low, particularly in the mouse data. This could indicate poor signal to noise ratio or a broad distribution of the signal, as the authors suggest. Nevertheless, this result should be made clear in the main text to avoid mis-interpretation by the reader regarding the analysis that focuses on peaks. Because the analysis of peaks only represents a minority of the total H4K20me1 signal, it should be made clear which figure panels focus on analysis of peaks (e.g. Figure 1B and 1C) and throughout the manuscript. This should also be mentioned in the main text. For example, when they conclude that the H3K20me1 signal substantially decreases at the late 2-cell stage (page 6), this should be changed to the number of H4K20me1 peaks. Similarly for Figure 1C they write that H4K20me1 was present in promoters etc. it should be made clear that this refers to H4K20me1 peaks.
I noticed that the reference for the H4K20me1 ChIP-seq dataset is incorrect (Pope et al., 2014 did not generate this data). Please cite ENCODE instead (PMID: 22955616) and add the reference to the appropriate Appendix Figure legends.
5. The axis labels are still not included in Appendix Figure S1 and S3 (what is 0, 1, 2 etc?). The other points have been addressed.
6. It should be made clear that maximum intensity projections are shown. Quantification should be performed on either a representative single sections or on 3D reconstructed images, not maximum intensity projections as these are not representative of the true signal.
9. These points have now been addressed. I noticed one other error in English 'H4K20me1 signaling' should be changed to H4K20me1 signal.

Advisor comments
1) The advisor is concerned about the data quality, particularly concerning the lack of an IgG control and the similarity to ATAC-seq data. Low-input Cut&Run methods generally do not include an IgG control as such controls typically fail to generate libraries from low input material (e.g. PMIDs 33821005, 33821003, 36215692, 40273908). The authors now present additional quality control information (statistics in Appendix Tables 1 and 2, and Appendix Figures 1-3) that generally show expected metrices. The correlation of their H4K02me1 datasets to published ChIP-seq H4K20me1 data in K562 cells, to bulk and between replicates suggests reproducibility. However, I do think the very low fraction of reads in peaks is a concern that I have highlighted, although this could reflect a broad distribution pattern of the signal as the authors suggest. I have requested the authors draw attention to this parameter in the main text and to any analysis that focuses on peaks (point 3).
In my view, the advisor's concern over the interpretations regarding the known cell cycle role of H4K20me1 are not valid here as this study does not assess cell cycle differences of H4K20me1 within the same stage and no relationship is claimed by the authors in the text. I do disagree with the author's argumentation in their response that the relationship of H4K20me1 to the cell cycle is different in early embryos, as to my knowledge this not yet been properly assessed in early mouse embryos, in either this manuscript or the cited papers.
While the data shows a general high correlation at early stages to ATAC-seq data (Figure 4D), this is not uncommon for many 'active' histone modifications and is not a concern in itself. In fact the tracks in Figure 4A suggest that the pattern/distribution of signal at all stages between H4K20me1 signal and ATAC-seq signal is different and as the authors argue, in some stages no correlation between H4K20me1 and ATAC-seq is observed (e.g. late 2-cell). Therefore, I think this concern has been addressed as far as possible.

2) Regarding the advisor's concern over the novelty I reference my original comment here:
'While Set8 has previously been described to be essential for early embryonic development in mouse, through knockout experiments, H4K20M mutant expression and use of the same inhibitor as in this manuscript (referenced papers: Oda et al., 2009, Shikata et al., 2020), the analysis of transcriptome and chromatin accessibility changes upon perturbation are novel.'
The authors have pointed out this in the introduction, although the Oda 2009 reference (PMID 19223465) is missing here (it is cited in the discussion). I recommend they add the Oda reference to the appropriate section in the introduction.
I am not sure what the advisor means by the following, 'considering PR-set7 is required for cell viability, it is not clear whether the interpretations of the authors on zygotic genome activation are fully explored and founded' without a specific criticism. In my opinion the analysis of ZGA defects in the inhibitor treated embryos is sound and demonstrates a clear role for H4K20me1 in ZGA in mouse embryos.

Cross-comments from referee 2:

I agree with the Reviewer that the authors should indicate whether all genes are plotted in Fig. EV1 as the enrichment across the majority of genes in the zygote, in particular, would be remarkable.

I do not think the parallel heatmaps analysis would be time-consuming as the requested data for numerous chromatin modifications and accessibility in early mouse embryos are publicly available. However, in my opinion, I do not think this is necessary because the requested analysis are generally present in the corresponding papers. Regarding the negative controls, as I mentioned in my reply to the advisor's comments, IgG controls are generally not performed in low-input Cut&Run with

embryo material as they typically fail to generate libraries.

In my opinion, the gene-centered aggregate profiles in Fig. EV1 do match the profiles shown in Fig 1D.

Response Letter

Dear Editors and Reviewers:

We would like to extend our sincere gratitude to you and the reviewers for the positive feedback and valuable suggestions on our revised manuscript entitled "The reprogramming and function of H4K20me1 during early development" (Manuscript ID: EMBOR-2025-62939V2).

We have carefully addressed the remaining points raised by the editors and reviewers. The following pages contain our point-by-point responses to each comment, along with a description of the corresponding revisions made in the manuscript. All changes are marked in red text in the revised manuscript. We deeply appreciate your time and effort in guiding us through the review process. With these adjustments, we hope the manuscript now meets the standards of *EMBO Reports*.

Yours Sincerely,

Dr. Jiawei Xu, Dr. Zhen He.

Corresponding author:

Jiawei Xu, Ph.D.

The First Affiliated Hospital of Zhengzhou University & Institute of Reproductive Health, Henan Academy of Innovations in Medical Science; Zhengzhou, 450000, China.

Email: fccxujw@zzu.edu.cn

Zhen He, Ph.D.

Institute of Reproductive Health, Henan Academy of Innovations in Medical Science; Zhengzhou, 450000, China.

Email: hezhen@hnsyxkxy.cn

Responses to the Editorial Requests:

- Both corr. authors need to have institutional email addresses, please add.

Response: Thanks for the reminder. Both corresponding authors have added institutional email addresses.

- The author credits need to be removed from the ms file. All credits need to be entered during online ms submission.

Response: The author credits have been removed from the manuscript file.

- Some FUNDING information is missing in our online submission system: Henan Province excellent innovation group project, Leading medical and health personnel of Central Plains region, Henan Province medical appropriate technology extension project. The funding information in the ms file and in our online system must be congruent. Please correct.

Response: We have corrected the funding information in the online submission system. The three funding items mentioned above have now been added.

- Please upload one source data folder per ms figure.

Response: We have uploaded one source data folder for each manuscript figure.

- Materials and Methods should be just Methods.

Response: We have changed “Materials and Methods” to “Methods”.

- Please note that weblink for the HRA012004 database is not accessible. It needs to be accessible with the submission of your final ms or at the very latest upon the online

publication of your study.

Response: We apologize for the oversight. The original link for HRA012004 was missing a hyphen. We have now corrected the weblink (https://ngdc.cncb.ac.cn/gsa-human/s/4a7KDbST), and it should be accessible. We will ensure the data is set to public upon acceptance of the manuscript.

- There are 2 tables provided in zip folders titled Appendix Table S1 and Appendix Table S2. I would like to suggest that you rename the files to Table EV1 and Table EV2. The 3 Appendix figures can also be EV figures, you can have 8 EV figures, this is fine, and the Appendix file can then be deleted. Each legend of the EV tables should be provided in a separate sheet/tab in the same Excel file, and all tables and EV figures need to be called out correctly in the ms text.

Response: Thank you for the suggestion. We have renamed Appendix Table S1 and S2 as Table EV1 and EV2, and converted the three Appendix figures to Fig EV1 and EV2. The original Fig EV1 and EV2 have been merged into Fig EV3, resulting in a total of six EV figures. The legends for EV tables are now provided in separate sheets within the same Excel file, and all citations in the text have been updated accordingly.

*Figure Legends - Comments *

- Please note that the exact p values are not provided in the legends of figures 5D, 7C, EV4 C, E, please provide exact values as reasonable.

Response: Thanks for the reminder. We have included the exact p values in the figures or their legends. Please note that for certain data points, the p values fall outside the precision limits of our statistical software. In Fig 5D, highly significant comparisons are denoted as $p < 0.0001$. In Fig 7C, the p value is so close to zero that it exceeds the calculation capability of the R program, resulting in a reported p value of $< 2.2\times10^{-16}$. The original Fig EV4C and E are now Fig EV5C and E.

- Please indicate the statistical test used for data analysis in the legends of figures 3F, H; 6D, 7A, C, EV5 G

Response: We have included the detailed statistical test in the legends of these figures. The original Fig EV5G are now Fig EV6H.

- Please note that the box plots need to be defined in terms of minima, maxima, centre, bounds of box and whiskers, and percentile in the legends of figures 7C, EV5 G

Response: We have provided clear definitions for the box plots in the legends of Fig 7C and EV6H (original EV5G).

- Please note that information related to n is missing in the legends of figures 7C, EV2 C, EV5 G

Response: The information related to n has been added. The original Fig EV2C and 5G are now Fig EV3D and 6H.

- Please note that scale bar and its definition are missing for figure 5E.

Response: We have added the scale bar and its definition.

I would like to suggest some minor changes to the title and abstract that needs to be written in present tense. Do you agree with:

The reprogramming and function of H4K20me1 during early embryo development

Histone modifications play critical roles in regulating chromatin dynamics and embryonic development. Among these, histone H4 lysine 20 mono-methylation (H4K20me1) is an essential epigenetic mark associated with gene expression and genome stability. However, the reprogramming and functional roles of H4K20me1 in

early embryogenesis remain unclear. Here, we map genome-wide distributions of H4K20me1 in mouse, human and zebrafish early embryos, revealing a broad distribution pattern along with species-specific features. H4K20me1 is predominantly enriched in gene bodies and undergoes dynamic erasure and reestablishment following fertilization. Functional perturbation of SET8, the only known H4K20me1 methyltransferase, results in developmental arrest, highlighting its necessity for embryogenesis. Mechanistically, H4K20me1 is crucial for zygotic genome activation (ZGA), where it regulates RNA synthesis and transcription, and promotes chromatin accessibility. Our findings provide insights into the dynamic reprogramming and regulatory functions of H4K20me1 in early developmental processes.

Response: Thank you very much for your thoughtful suggestion. We appreciate your careful reading and fully agree with your proposed changes, which have made the title and abstract much clearer. We have revised them as suggested.

EMBO press papers are accompanied online by A) a short (1-2 sentences) summary of the findings and their significance, B) 2-3 bullet points highlighting key results and C) a synopsis image that is exactly 550 pixels wide and 200-600 pixels high (the height is variable). The synopsis image should provide a sketch of the major findings, like a graphical abstract. Please note that text needs to be readable at the final size. Please send us this information along with the final manuscript.

Response: We have provided a short summary, three bullet points and a synopsis image as requested.

Responses to Referee #1:

Overall, the authors have generally addressed my concerns. There are still some issues with rigor and clarity:

- The new heatmaps in EV1 provide valuable insight. It is unclear whether they represent a subset of genes or all genes -- if the latter, that would be very surprising given that the vast majority of genes seem to be marked at zygote stage. To put these trends into context and evaluate their impact, it seems like parallel heatmaps of other chromatin characteristics (accessibility, H3K4 methylation, etc.) would be needed. To some extent, the authors address this in Fig 4, but again chose not to show parallel heatmaps of the different chromatin signals, which would better illustrate the locus-specific trends and overall genome-wide (across all genes) effect. Negative controls (e.g., IgG) would also be useful to ensure the signals are specific.

Additionally, the aggregate profiles shown above the EV1 heatmaps don't seem to exactly match the Fig1D aggregate profiles, suggesting that some locus curation may have occurred.

I want to emphasize that the stage-to-stage variability of H4K20me1 coverage relative to genes is compelling, and seems unlikely to arise from any technical artifact. But I think the genomics analyses could have gone further to establish what those precise patterns are, especially relative to what we already understand about embryonic chromatin dynamics at these early stages.

Response:

Thank you for these constructive comments. Regarding the heatmaps in Fig EV1 (now Fig EV3B), we would like to clarify that they represent all genes. We have now added this information to the figure legend. H4K20me1 peaks are most abundant in oocytes and undergo partial erasure following fertilization. In the zygote stage, H4K20me1 is predominantly enriched at promoters, and gradually shifts toward gene bodies in subsequent stages.

We fully agree that parallel heatmaps of other chromatin characteristics would

provide valuable context. Given that comprehensive datasets for these modifications in early mouse embryos are already publicly available and have been extensively characterized in the corresponding publications, and as Referee #2 noted, we respectfully consider that duplicating these analyses here may not be essential for the core findings of our study. We sincerely appreciate this suggestion and will keep it in mind for future investigations.

Regarding the IgG control, as also noted by Referee #2, such controls are generally not performed in low-input CUT&RUN experiments with early embryo materials, as they typically fail to generate informative libraries. Therefore, we have instead provided additional quality control information to show expected metrics (Table EV2).

As for the aggregate profiles, we believe that the trends shown in the gene-centered heatmaps of Fig EV1 (now Fig EV3B) are consistent with those in Fig 1D, and we appreciate your careful examination of these figures.

- Response Fig 2, ChIPseqSpikeInFree should be generating uniform scaling factors, which simply scale linearly in the same way as CPM normalization. So, the non-linear trends shown in the graph don't make sense in this regard, unless scaling was applied in an unconventional way.

The statement in the text "The relative H4K20me1 enrichment patterns across these stages were robust, as confirmed by ChIPseqSpikeInFree normalization assessment" is hard to interpret. What does robust precisely mean here?

Response:

Thank you for raising this important point. We agree that ChIPseqSpikeInFree outputs a single scaling factor per sample and thus performs only linear rescaling, not

non-linear normalization. We applied the method across all developmental stages, but scaling factors were only generated for the three stages: GV oocyte (1.39), MII oocyte (1.80), and blastocyst (2.21). For other stages, no valid scaling factors were returned.

Our original intention was to use the available scaling factors to rescale the data and compare with the original CPM-normalized values, to assess whether the relative H4K20me1 patterns across stages remained consistent. In stages where valid scaling factors were obtained, linear rescaling by ChIPseqSpikeInFree did not substantially alter the relative enrichment trends. For stages where no scaling factors were generated, the method was not applicable, and we therefore relied on CPM-normalized data for presentation. When we described the patterns as “robust,” we meant that the high correlation between the two methods in those stages gave us confidence in our CPM normalization.

We recognize that this analysis did not ultimately add meaningful value to the study. After careful consideration, we have removed the description and analysis related to ChIPseqSpikeInFree from the revised manuscript. Thank you again for your helpful comments.

- Response Fig 3, it's not clear how pausing is defined relative to stage. It seems like genes are grouped into paused/not paused groups that are assessed for H4K20me1 across all stages, but wouldn't we expect different genes be paused at different stages; and that the pausing dynamics during transcriptional quiescence (prior to zygotic genome activation) be challenging to define? This analysis is too under-explained to be compelling.

Response:

We sincerely thank you for this highly insightful comment, and we apologize for our oversight in the previous revision. To further address your concern regarding

stage-specific pausing dynamics, we regenerated the heatmap and performed an additional analysis examining the composition of paused genes across developmental stages. This analysis revealed that H4K20me1 signals at both paused and non-paused genes exhibit distinct patterns before and after ZGA (Response Fig 1A and B). Moreover, both the number and identity of paused genes change dynamically across stages (Response Fig 1C and D), indicating that pausing status is not a fixed property of individual genes throughout development. Nevertheless, substantial overlaps between adjacent stages suggest that pausing features are dynamically remodeled while retaining some continuity.

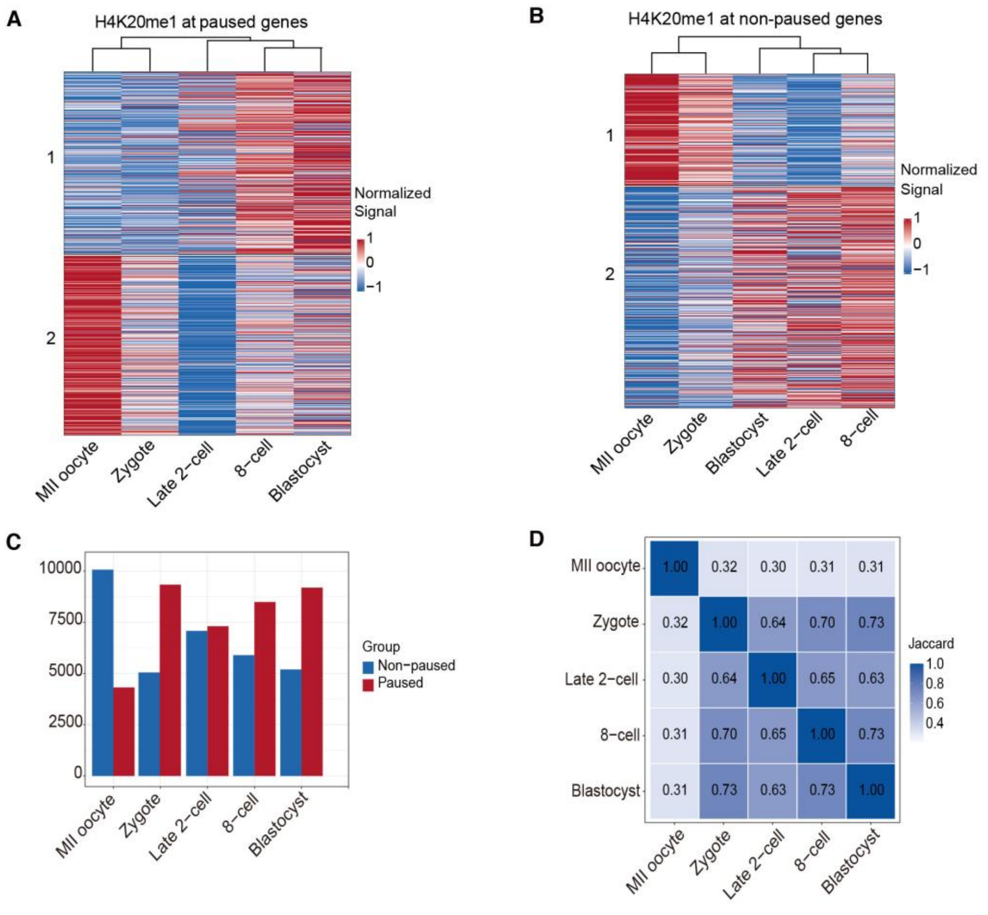

Response Fig 1. Dynamic changes of paused and non-paused genes during early mouse embryogenesis. A-B) H4K20me1 signals at paused and non-paused genes. C) Numbers of paused and non-paused genes per stage. D) Jaccard overlap of paused

gene sets between stages.

Our original intention was to analyze the reprogramming dynamics of H4K20me1 at promoters and gene bodies, as these are the regions where this mark is most prominently enriched. We acknowledge that applying the "transcriptional pausing" classification to early, pre-ZGA stages may be conceptually imprecise. In the interest of rigor, and because removing this analysis does not alter the main conclusions of our study, we have removed the pausing classification analysis and its associated text from the revised manuscript. The additional analysis described above is therefore not included in the manuscript, but we hope it helps clarify the dynamic nature of pausing across stages.

To more directly assess the relationship between H4K20me1 and transcriptional activity, as presented in Fig EV6D, we analyzed Pol II loading relative to H4K20me1 enrichment. This analysis demonstrates that Pol II is highly loaded at loci where H4K20me1 is enriched. We hope this helps clarify the relationship between H4K20me1 and the transcriptional machinery.

- Response Fig 4, the micrographs are easier to interpret now, but it is still challenging to see the DAPI signal. I would again suggest greyscale/false coloring

Response:

Thanks for the suggestion. To improve the visibility of the DAPI signal, we have replaced the original blue color with cyan in the updated micrographs (Response Fig 2).

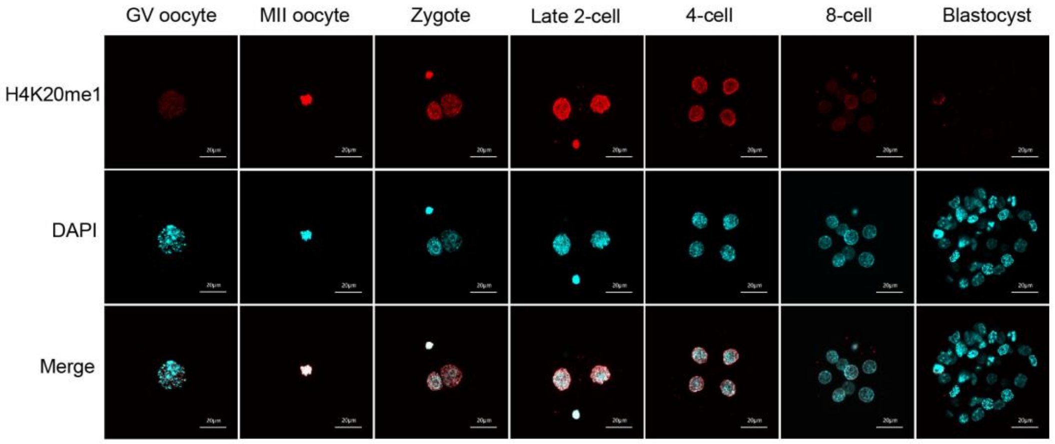

Response Fig 2. H4K20me1 localization during mouse preimplantation development. Immunofluorescence images showing H4K20me1 (red), DAPI-stained nuclei (cyan), and the merged signals in oocytes and early embryos. Scale bar, 20 μm.

- The added clarity to gene categories in the RNA-seq analysis helps, but still seems to avoid explicitly addressing whether changes are likely due to transcriptional or post-transcriptional effects relative to the genes' untreated dynamics during the maternal-to-zygotic transition. For example, maternal genes (high in MII) can still be further activated at ZGA, or they can be subject to clearance.

Fig 7C is likely not an appropriate comparison. The test does not appear to be stated anywhere -- at minimum, it should be a paired non-parametric test. However, differential expression analysis combined with heatmaps would be a better way to demonstrate such trends.

Response:

Thanks for these insightful comments. We agree that our current RNA-seq analysis cannot fully decouple transcriptional from post-transcriptional effects, particularly regarding the complex dynamics of maternal transcripts. Our analysis demonstrates that H4K20me1 inhibition significantly reduces the expression of ZGA genes. Because these specific ZGA genes are virtually absent in the MII stage and require *de*

novo synthesis following fertilization, their downregulation provides evidence that H4K20me1 inhibition impairs their transcriptional activation. We deeply appreciate you pointing out the broader complexities of MZT dynamics, and we will incorporate more specialized methods to address this specific distinction in our future research.

Regarding the statistical test in Fig 7C, we have now used the Wilcoxon signed-rank test and have stated this in the figure legend. Additionally, as suggested, we have included a heatmap (Response Fig 3, new Fig EV6G) to provide a more comprehensive visualization of the expression changes in differentially expressed ZGA genes.

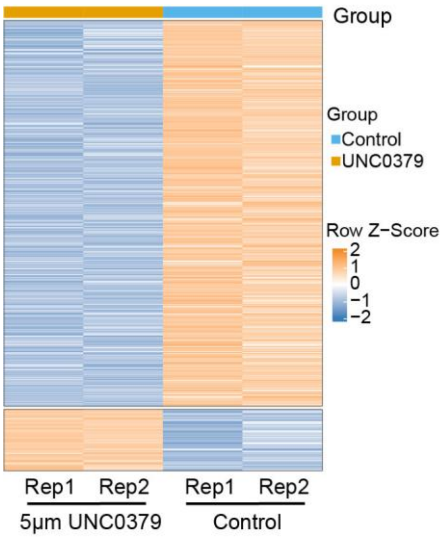

Response Fig 3. Expression changes of differentially expressed ZGA genes upon H4K20me1 inhibition in early mouse embryos. Heatmap showing expression levels of differentially expressed ZGA genes (n = 568) in control and UNC0379-treated samples. Signal intensity is Z-score normalized, with rows representing individual genes and columns representing samples (DESeq2, FDR < 0.05, FC > 2).

- In the intro -- I still disagree that H3K4me3 and H3K27me3 exhibit narrow peaks "canonically" -- broad enrichment is a feature of both across contexts, not just in mouse oocytes. Highly transcribed genes have H3K4me1/2/3 that extends well into

the gene body, facultative heterochromatin is marked by broad H3K27me3.

Response:

Thank you for the correction and for sharing this valuable insight. To avoid any misrepresentation, we have removed this problematic sentence from the introduction in the revised manuscript.

- Throughout, the authors use the word significant or significantly, but do not have statistical tests to support significance.

Response:

Thank you very much for pointing this out. We have carefully reviewed the manuscript and replaced unsupported uses of "significant/significantly" with more appropriate terms such as "substantial," "marked," or "notably" throughout the text.

Responses to Referee #2:

The majority of my comments have now been addressed. I have a few outstanding comments listed below:

3. I appreciate the additions the authors have made to address this point, including the discussion regarding the difference between the genomics and the immunofluorescence data and the sequencing control quality statistics. This indicates that the fraction of reads in peaks (FRIP) are very low, particularly in the mouse data. This could indicate poor signal to noise ratio or a broad distribution of the signal, as the authors suggest. Nevertheless, this result should be made clear in the main text to avoid mis-interpretation by the reader regarding the analysis that focuses on peaks. Because the analysis of peaks only represents a minority of the total H4K20me1 signal, it should be made clear which figure panels focus on analysis of peaks (e.g.

Figure 1B and 1C) and throughout the manuscript. This should also be mentioned in the main text. For example, when they conclude that the H3K20me1 signal substantially decreases at the late 2-cell stage (page 6), this should be changed to the number of H4K20me1 peaks. Similarly for Figure 1C they write that H4K20me1 was present in promoters etc. it should be made clear that this refers to H4K20me1 peaks.

I noticed that the reference for the H4K20me1 ChIP-seq dataset is incorrect (Pope et al., 2014 did not generate this data). Please cite ENCODE instead (PMID: 22955616) and add the reference to the appropriate Appendix Figure legends.

Response:

We sincerely thank you for these highly constructive suggestions. To avoid misinterpretation, we have explicitly addressed the low FRiP values in the main text (page 6), noting that they likely reflect the inherently broad distribution of H4K20me1 or potentially lower signal-to-noise ratio typical of low-input samples. We have also revised the manuscript and figure legends to clearly distinguish analyses based on peaks versus those based on global signal. For example, the statement on page 6 has been corrected from “signal was substantially reduced” to “the number of peaks was substantially reduced.”

Regarding the citation error, we apologize for the oversight. We have corrected the reference to the ENCODE dataset (page 5) and updated the corresponding figure legends (now Fig EV1). We appreciate your careful guidance.

5. The axis labels are still not included in Appendix Figure S1 and S3 (what is 0, 1, 2 etc?). The other points have been addressed.

Response:

Thanks for the reminder. We have now added axis labels to the figure legends of Appendix Figure S1 and S3 (now Fig EV1 and 2), indicating that the values represent log2(normalized signal + 1).

6. It should be made clear that maximum intensity projections are shown. Quantification should be performed on either a representative single sections or on 3D reconstructed images, not maximum intensity projections as these are not representative of the true signal.

Response:

Thank you for pointing out this important technical detail. We have now clarified in the methods (page 21) that the displayed images are maximum intensity projections, and that quantification was performed on representative single sections. The re-quantified data remain consistent with our original conclusions, and have been incorporated into the revised figures.

9. These points have now been addressed. I noticed one other error in English 'H4K20me1 signaling' should be changed to H4K20me1 signal.

Response:

Thanks for the correction. We have changed “H4K20me1 signaling” to “H4K20me1 signal” throughout the manuscript.

Advisor comments

1) The advisor is concerned about the data quality, particularly concerning the lack of an IgG control and the similarity to ATAC-seq data. Low-input Cut&Run methods generally do not include an IgG control as such controls typically fail to generate libraries from low input material (e.g. PMIDs 33821005, 33821003, 36215692, 40273908). The authors now present additional quality control information (statistics in Appendix Tables 1 and 2, and Appendix Figures 1-3) that generally show expected metrices. The correlation of their H4K02me1 datasets to published ChIP-seq H4K20me1 data in K562 cells, to bulk and between replicates suggests reproducibility.

However, I do think the very low fraction of reads in peaks is a concern that I have highlighted, although this could reflect a broad distribution pattern of the signal as the authors suggest. I have requested the authors draw attention to this parameter in the main text and to any analysis that focuses on peaks (point 3).

In my view, the advisor's concern over the interpretations regarding the known cell cycle role of H4K20me1 are not valid here as this study does not assess cell cycle differences of H4K20me1 within the same stage and no relationship is claimed by the authors in the text. I do disagree with the author's argumentation in their response that the relationship of H4K20me1 to the cell cycle is different in early embryos, as to my knowledge this not yet been properly assessed in early mouse embryos, in either this manuscript or the cited papers.

While the data shows a general high correlation at early stages to ATAC-seq data (Figure 4D), this is not uncommon for many 'active' histone modifications and is not a concern in itself. In fact the tracks in Figure 4A suggest that the pattern/distribution of signal at all stages between H4K20me1 signal and ATAC-seq signal is different and as the authors argue, in some stages no correlation between H4K20me1 and ATAC-seq is observed (e.g. late 2-cell). Therefore, I think this concern has been addressed as far as possible.

2) Regarding the advisor's concern over the novelty I reference my original comment here:

'While Set8 has previously been described to be essential for early embryonic development in mouse, through knockout experiments, H4K20M mutant expression and use of the same inhibitor as in this manuscript (referenced papers: Oda et al., 2009, Shikata et al., 2020), the analysis of transcriptome and chromatin accessibility changes upon perturbation are novel.'

The authors have pointed out this in the introduction, although the Oda 2009 reference (PMID 19223465) is missing here (it is cited in the discussion). I recommend they

add the Oda reference to the appropriate section in the introduction.

I am not sure what the advisor means by the following, 'considering PR-set7 is required for cell viability, it is not clear whether the interpretations of the authors on zygotic genome activation are fully explored and founded' without a specific criticism. In my opinion the analysis of ZGA defects in the inhibitor treated embryos is sound and demonstrates a clear role for H4K20me1 in ZGA in mouse embryos.

Response:

We sincerely thank you for taking the time to summarize and address the advisor's concerns, and for providing such constructive guidance throughout the review process. As you suggested, we have discussed the low FRiP values in the main text and distinguished peak-based analyses from global signal analyses to avoid misinterpretation. Regarding the Oda 2009 reference, thank you for the correction. We have now added this citation in the introduction (page 4). We greatly appreciate your efforts in helping us improve the manuscript.

Cross-comments from referee 2:

I agree with the Reviewer that the authors should indicate whether all genes are plotted in Fig. EV1 as the enrichment across the majority of genes in the zygote, in particular, would be remarkable.

I do not think the parallel heatmaps analysis would be time-consuming as the requested data for numerous chromatin modifications and accessibility in early mouse embryos are publicly available. However, in my opinion, I do not think this is necessary because the requested analysis are generally present in the corresponding papers. Regarding the negative controls, as I mentioned in my reply to the advisor's comments, IgG controls are generally not performed in low-input Cut&Run with

embryo material as they typically fail to generate libraries.

In my opinion, the gene-centered aggregate profiles in Fig. EV1 do match the profiles shown in Fig 1D.

Response:

Thank you very much for your helpful clarification regarding the IgG controls, parallel heatmaps analysis, and for your support on the gene-centered aggregate profiles in Fig EV1 (now Fig EV3B). We confirm that the heatmaps include all genes, as now stated in the figure legend. We truly appreciate you taking the time to clarify these points.

Prof. Jiawei Xu
The First Affiliated Hospital of Zhengzhou University
Institute of Reproductive Health
China

Dear Prof. Xu,

I am very pleased to accept your manuscript for publication in the next available issue of EMBO reports. Thank you for your contribution to our journal.

Your manuscript will be processed for publication by EMBO Press. It will be copy edited and you will receive page proofs prior to publication. Please note that you will be contacted by Springer Nature Author Services to complete licensing and payment information.

You may qualify for financial assistance for your publication charges - either via a Springer Nature fully open access agreement or an EMBO initiative. Check your eligibility: https://link.springer.com/journal/44319/how-to-publish-with-us

Should you be planning a Press Release on your article, please get in contact with embo_production@springernature.com as early as possible in order to coordinate publication and release dates.

If you have any questions, please do not hesitate to contact the Editorial Office. Thank you for your contribution to EMBO Reports.

Best regards,
Esther

Esther Schnapp, PhD
Senior Editor
EMBO reports

>>> Please note that it is EMBO Reports policy for the transcript of the editorial process (containing referee reports and your response letter) to be published as an online supplement to each paper. If you do NOT want this, you will need to inform the Editorial Office via email immediately. More information is available here: https://link.springer.com/partners/embo-press/editorial-policies#Peer%20review

EMBO Press Author Checklist

Corresponding Author Name: Jiawei Xu	**USEFUL LINKS FOR COMPLETING THIS FORM**
Journal Submitted to: EMBO Reports	The EMBO Journal - Author Guidelines
Manuscript Number: EMBOR-2025-62939V3	EMBO Reports - Author Guidelines
	Molecular Systems Biology - Author Guidelines
	EMBO Molecular Medicine - Author Guidelines

Reporting Checklist for Life Science Articles (updated January
This checklist is adapted from Materials Design Analysis Reporting (MDAR) Checklist for Authors. MDAR establishes a minimum set of requirements in transparent reporting in the life sciences (see Statement of Task: 10.31222/osf.io/9sm4x). Please follow the journal's guidelines in preparing your
Please note that a copy of this checklist will be published alongside your article.

Abridged guidelines for figures
1. Data
The data shown in figures should satisfy the following conditions:
- the data were obtained and processed according to the field's best practice and are presented to reflect the results of the experiments in an accurate and unbiased manner.
- ideally, figure panels should include only measurements that are directly comparable to each other and obtained with the same assay.
- plots include clearly labeled error bars for independent experiments and sample sizes. Unless justified, error bars should not be shown for technical
- if n<5, the individual data points from each experiment should be plotted. Any statistical test employed should be justified.
- Source Data should be included to report the data underlying figures according to the guidelines set out in the authorship guidelines on Data

2. Captions
Each figure caption should contain the following information, for each panel where they are relevant:
- a specification of the experimental system investigated (eg cell line, species name).
- the assay(s) and method(s) used to carry out the reported observations and measurements.
- an explicit mention of the biological and chemical entity(ies) that are being measured.
- an explicit mention of the biological and chemical entity(ies) that are altered/varied/perturbed in a controlled manner.
- the exact sample size (n) for each experimental group/condition, given as a number, not a range;
- a description of the sample collection allowing the reader to understand whether the samples represent technical or biological replicates (including how many animals, litters, cultures, etc.).
- a statement of how many times the experiment shown was independently replicated in the laboratory.
- definitions of statistical methods and measures:
  - common tests, such as t-test (please specify whether paired vs. unpaired), simple χ2 tests, Wilcoxon and Mann-Whitney tests, can be unambiguously identified by name only, but more complex techniques should be described in the methods section;
  - are tests one-sided or two-sided?
  - are there adjustments for multiple comparisons?
  - exact statistical test results, e.g., P values = x but not P values < x;
  - definition of 'center values' as median or average;
  - definition of error bars as s.d. or s.e.m.

Please complete ALL of the questions below.
Select "Not Applicable" only when the requested information is not relevant for your study.

Materials

Newly Created Materials	Information included in the manuscript?	In which section is the information available? (Reagents and Tools Table, Materials and Methods, Figures, Data Availability Section)
New materials and reagents need to be available; do any restrictions apply?	Not Applicable	

Antibodies	Information included in the manuscript?	In which section is the information available? (Reagents and Tools Table, Materials and Methods, Figures, Data Availability Section)
For **antibodies** provide the following information:<br>- Commercial antibodies: RRID (if possible) or supplier name, catalogue number and or/clone number<br>- Non-commercial: RRID or citation	Yes	Materials and Methods

DNA and RNA sequences	Information included in the manuscript?	In which section is the information available? (Reagents and Tools Table, Materials and Methods, Figures, Data Availability Section)
**Short novel DNA or RNA including primers, probes**: provide the sequences.	Not Applicable	

Cell materials	Information included in the manuscript?	In which section is the information available? (Reagents and Tools Table, Materials and Methods, Figures, Data Availability Section)
**Cell lines:** Provide species information, strain. Provide accession number in repository **OR** supplier name, catalog number, clone number, and/**OR** RRID.	Yes	Materials and Methods
**Primary cultures:** Provide species, strain, sex of origin, genetic modification status.	Not Applicable	
Report if the cell lines were recently **authenticated** (e.g., by STR profiling) and tested for mycoplasma contamination.	Yes	Materials and Methods

Experimental animals	Information included in the manuscript?	In which section is the information available? (Reagents and Tools Table, Materials and Methods, Figures, Data Availability Section)
**Laboratory animals or Model organisms:** Provide species, strain, sex, age, genetic modification status. Provide accession number in repository **OR** supplier name, catalog number, clone number, **OR** RRID.	Yes	Materials and Methods
**Animal observed in or captured from the field:** Provide species, sex, and age where possible.	Not Applicable	
Please detail **housing and husbandry conditions**.	Yes	Materials and Methods

Plants and microbes	Information included in the manuscript?	In which section is the information available? (Reagents and Tools Table, Materials and Methods, Figures, Data Availability Section)
**Plants:** provide species and strain, ecotype and cultivar where relevant, unique accession number if available, and source (including location for collected wild specimens).	Not Applicable	
**Microbes:** provide species and strain, unique accession number if available, and source.	Not Applicable	

Human research participants	Information included in the manuscript?	In which section is the information available? (Reagents and Tools Table, Materials and Methods, Figures, Data Availability Section)
If collected and within the bounds of privacy constraints report on age, sex and gender or ethnicity for all study participants.	Yes	Materials and Methods

Core facilities	Information included in the manuscript?	In which section is the information available? (Reagents and Tools Table, Materials and Methods, Figures, Data Availability Section)
If your work benefited from core facilities, was their service mentioned in the acknowledgments section?	Yes	Acknowledgments

Design

Study protocol	Information included in the manuscript?	In which section is the information available? (Reagents and Tools Table, Materials and Methods, Figures, Data Availability Section)
If study protocol has been **pre-registered, provide DOI in the manuscript**. For clinical trials, provide the trial registration number **OR** cite DOI.	Not Applicable	
Report the **clinical trial registration number** (at ClinicalTrials.gov or equivalent), where applicable.	Not Applicable	

Laboratory protocol	Information included in the manuscript?	In which section is the information available? (Reagents and Tools Table, Materials and Methods, Figures, Data Availability Section)
Provide DOI OR other citation details if **external detailed step-by-step protocols** are available.	Not Applicable	

Experimental study design and statistics	Information included in the manuscript?	In which section is the information available? (Reagents and Tools Table, Materials and Methods, Figures, Data Availability Section)
Include a statement about **sample size** estimate even if no statistical methods were used.	Yes	Figures
Were any steps taken to minimize the effects of subjective bias when allocating animals/samples to treatment (e.g. **randomization procedure**)? If yes, have they been described?	Yes	Materials and Methods
Include a statement about **blinding** even if no blinding was done.	Not Applicable	
Describe **inclusion/exclusion criteria** if samples or animals were excluded from the analysis. Were the criteria pre-established?<br><br>If sample or data points were omitted from analysis, report if this was due to attrition or intentional exclusion and provide justification.	Not Applicable	
For every figure, are **statistical tests** justified as appropriate? Do the data meet the assumptions of the tests (e.g., normal distribution)? Describe any methods used to assess it. Is there an estimate of variation within each group of data? Is the variance similar between the groups that are being statistically compared?	Yes	Figures

Sample definition and in-laboratory replication	Information included in the manuscript?	In which section is the information available? (Reagents and Tools Table, Materials and Methods, Figures, Data Availability Section)
In the figure legends: state number of times the experiment was **replicated** in laboratory.	Yes	Figures
In the figure legends: define whether data describe **technical or biological replicates**.	Yes	Figures

Ethics

Ethics	Information included in the manuscript?	In which section is the information available? (Reagents and Tools Table, Materials and Methods, Figures, Data Availability Section)
Studies involving **human participants**: State details of **authority granting ethics approval** (IRB or equivalent committee(s), provide reference number for approval.	Yes	Materials and Methods
Studies involving **human participants**: Include a statement confirming that **informed consent** was obtained from all subjects and that the experiments conformed to the principles set out in the WMA Declaration of Helsinki and the Department of Health and Human Services Belmont Report.	Yes	Materials and Methods
Studies involving **human participants:** For publication of **patient photos**, include a statement confirming that consent to publish was obtained.	Not Applicable	
Studies involving experimental **animals**: State details of **authority granting ethics approval** (IRB or equivalent committee(s), provide reference number for approval. Include a statement of compliance with ethical regulations.	Yes	Materials and Methods
Studies involving **specimen and field samples:** State if relevant **permits** obtained, provide details of authority approving study; if none were required, explain why.	Not Applicable	

Dual Use Research of Concern (DURC)	Information included in the manuscript?	In which section is the information available? (Reagents and Tools Table, Materials and Methods, Figures, Data Availability Section)
Could your study fall under dual use research restrictions? Please check biosecurity documents and list of **select agents and toxins** (CDC): https://www.selectagents.gov/sat/list.htm	Not Applicable	
If you used a select agent, is the security level of the lab appropriate and reported in the manuscript?	Not Applicable	
If a study is subject to dual use research of concern regulations, is the name of the **authority granting approval and reference number** for the regulatory approval provided in the manuscript?	Not Applicable	

Reporting

The MDAR framework recommends adoption of discipline-specific guidelines, established and endorsed through community initiatives. Journals have their own policy about requiring specific guidelines and recommendations to complement MDAR.

Adherence to community standards	Information included in the manuscript?	In which section is the information available? (Reagents and Tools Table, Materials and Methods, Figures, Data Availability Section)
State if relevant guidelines or checklists (e.g., **ICMJE, MIBBI, ARRIVE, PRISMA**) have been followed or provided.	Not Applicable	
For **tumor marker prognostic studies,** we recommend that you follow the **REMARK** reporting guidelines (see link list at top right). See author guidelines, under 'Reporting Guidelines'. Please confirm you have followed these guidelines.	Not Applicable	
For **phase II and III randomized controlled trials**, please refer to the **CONSORT** flow diagram (see link list at top right) and submit the CONSORT checklist (see link list at top right) with your submission. See author guidelines, under 'Reporting Guidelines'. Please confirm you have submitted this list.	Not Applicable	

Data Availability

Data availability	Information included in the manuscript?	In which section is the information available? (Reagents and Tools Table, Materials and Methods, Figures, Data Availability Section)
Have **primary datasets** been deposited according to the journal's guidelines (see 'Data Deposition' section) and the respective accession numbers provided in the Data Availability Section?	Yes	Data Availability
Were **human clinical and genomic datasets** deposited in a public access-controlled repository in accordance to ethical obligations to the patients and to the applicable consent agreement?	Yes	Data Availability
Are **computational models** that are central and integral to a study available without restrictions in a machine-readable form? Were the relevant accession numbers or links provided?	Not Applicable	
If publicly available data were reused, provide the respective **data citations in the reference list**.	Yes	Reference
